# A Continental-Wide Perspective: The Genepool of Nuclear Encoded Ribosomal DNA and Single-Copy Gene Sequences in North American *Boechera* (Brassicaceae)

**DOI:** 10.1371/journal.pone.0036491

**Published:** 2012-05-14

**Authors:** Christiane Kiefer, Marcus A. Koch

**Affiliations:** 1 Department of Plant Developmental Biology, Max Planck Institute for Plant Breeding Research, Cologne, Germany; 2 Department of Biodiversity and Plant Systematics, Heidelberg University, Centre for Organismal Studies Heidelberg, Heidelberg, Germany; American University in Cairo, Egypt

## Abstract

74 of the currently accepted 111 taxa of the North American genus *Boechera* (Brassicaceae) were subject to pyhlogenetic reconstruction and network analysis. The dataset comprised 911 accessions for which ITS sequences were analyzed. Phylogenetic analyses yielded largely unresolved trees. Together with the network analysis confirming this result this can be interpreted as an indication for multiple, independent, and rapid diversification events. Network analyses were superimposed with datasets describing i) geographical distribution, ii) taxonomy, iii) reproductive mode, and iv) distribution history based on phylogeographic evidence. Our results provide first direct evidence for enormous reticulate evolution in the entire genus and give further insights into the evolutionary history of this complex genus on a continental scale. In addition two novel single-copy gene markers, orthologues of the *Arabidopsis thaliana* genes *At2g25920* and *At3g18900*, were analyzed for subsets of taxa and confirmed the findings obtained through the ITS data.

## Introduction

The North American genus *Boechera* Á. Löve & D Löve (Brassicaceae) is notorious for its complex taxonomy, various modes of reproduction and hybridisation patterns. *Boechera* belongs to the tribe Boechereae along with six closely related genera, *Anelsonia* J.F. Macbr. & Payson, *Cusickiella* Rollins, *Nevada* N.H. Holmgren, *Phoenicaulis* Nutt., *Polyctenium* Greene, and *Sandbergia* Greene [1], [2], [3], [4]. It was shown recently, that Eastern North American *Boechera* species are most closely related to *Borodinia* [4] and the taxonomical decision was left open to either integrate those *Boechera* species into *Borodinia*, or to revise the genus *Borodinia*.

Species of *Boechera* grow in diverse habitats ranging from alpine regions to meadows and deserts making it a suitable model system for evolutionary genomics and molecular ecology [5], also for a growing scientific community [6]. A phylogeographic-evolutionary scenario (chloroplast DNA (cpDNA) sequence variation) has been established for the genus in two studies [7], [8]. In these studies a significant amount of cpDNA haplotype sharing was found, presumably indicating the reticulate relationships but also significant sharing of ancestral DNA polymorphisms [7], [8]. A first case-study, which focused on a small number of taxa (*B. stricta*, *B. holboellii* (as *Arabis* sensu Rollins [9] and *B divaricarpa* (as *Arabis* sensu Rollins [9]) investigated DNA sequence variation of the Internally Transcribed Spacers 1 and 2 and was able to assign various ITS types to few distinct groups (referred to as ITS in the following) [10]. It was also shown that the resolution among ITS sequence types, considering a small number of taxa with a limited number of ITS types, is low and biased by sample size and geographic coverage. Still a study of a comprehensive dataset of nuclear encoded biparentally inherited genes is missing.

*Boechera* has a base chromosome number of x = 7 and the various species occur as diploids, triploids, very rarely tetraploids, and a few aneuploids with 15 chromosomes [11], [12], [13]. Since sexual reproduction is dependent on an even chromosome number, triploidy mostly precludes this reproductive pathway and hence triploids are facultative apomicts. However, also diploid/aneuploid apomictic species and populations are known [14], [13].

Apomictic reproduction in *Boechera* has been studied extensively e.g. [15], [16]. These studies showed that apomictic *Boechera* are not strictly asexually reproducing, but some percentage of offspring may be produced by sexual reproduction leading to the introgression of mostly apomictic individuals into sexual populations [17]. Apomicts in *Boechera* are thought to be of hybrid origin [14]. Hybridisation in *Boechera* occurred many times independently throughout the Pleistocene involving the better part of the diploid species [7], [10], [14].

The best studied diploid species within *Boechera* is probably *Boechera stricta*, which represents an early separated lineage in respect to cpDNA [8]. Populations of this taxon have been studied in respect to population structure and linkage disequilibrium [18] but also natural selection on flowering time has been investigated leading to the identification of several QTLs related to flowering time in wild populations or in growth chamber experiments [19].

Taxonomically, *Boechera* used to be treated as member of the highly polyphyletic genus *Arabis* L. [20]. *Arabis holboellii*, was described by Hornemann in 1827 [21] and was designated by Löve and Löve [22] as the type of their newly segregated *Boechera.* However, Rollins [9] reduced *Boechera* to synonymy of *Arabis,* and the genus was not firmly established until 2003 (see [23]). *Arabis holboellii* (sensu Rollins [9] was a highly polymorphic taxon that included several varieties with diploids, triploids, and aneuploids [24], [25]. A series of molecular studies showed *Arabis* to be highly polyphyletic [20], [26], [27], [28], [29], [30], and most of its species have since been transferred into *Boechera* [23], [31], [32], [33].

In their recent Flora of North America (FNA) account, Al-Shehbaz and Windham [14] recognized 73 sexual diploid and 38 apomictic diploid or triploid taxa, and some of Rollins [9] *Arabis* varieties (e.g., the current *B. retrofracta*) were recognized as distinct species, whereas the ranges of others (e.g., the current *B. holboellii*) have become restricted. Extensive molecular studies using microsatellites and several single-copy nuclear genes (Windham et al., in progress; Alexander et al., submitted; Beck et al., submitted) should provide a better understanding of the taxonomic complexity of the genus (see also Table S1).

When faced with the task of reconstructing the phylogeny of a species-rich, taxonomically difficult, and hyper-diverse genus, the question arises which marker system should be employed. The most frequently applied nuclear encoded marker system in plant systematics is the Internally Transcribed Spacer region (ITS1 and ITS2 together referred to as ITS) [34]. The ITS is found in thousands of copies arranged in tandem arrays in the nuclear genome and is located between the highly conserved rRNA encoding genes [35]. Fluorescence in situ hybridisation in *Boechera* revealed that rDNA is arranged in two arrays, one consisting of overlapping 45 S (including the 18 S, 5.8 S and 25 S rRNA genes which are separated by ITS1 and ITS2) and 5 S rDNA and one consisting only of 5 S rDNA in sexual *Boechera “holboellii”* and *Boechera stricta*. However the size of the overlapping 45 S and 5S rDNA region was different. Apomictic accessions also showed a repeat size polymorphism [13]. The ITS region is claimed to have several advantages like the high degree of conservation of rRNA encoding genes which enables the design of common primers that can be used across large groups of taxa [36], [37], [38]. Amplification is also unproblematic due to the high copy number and can be achieved easily from DNA obtained from herbarium specimens. Successful large-scale phylogenetic reconstructions using the ITS have for example been achieved for the Brassicaceae [3], [30], [39], [40]. However, despite all these claimed benefits the ITS may also cause several problems which have been summarized by [41]: among them are the presence of multiple rDNA arrays and orthologues and paralogues, concerted evolution leading to chimeric ITS types (for specific examples see [42], [43], [44], for examples of non-concerted evolution see [45]), presence of pseudogenes (see also [43]), compensatory base changes due to functional constraints (secondary structure), problems with alignment, accuracy and rooting of trees due to the presence of large numbers of indels and high GC content, contamination due to the universality of the used primers, abundant homoplasy and technical problems such as selective amplification of certain ITS versions by the applied PCR conditions. Despite all these complications we decided to make use of this commonly used marker system in order to extend previously generated datasets which were based on ITS data and to use the complexity of the sequence as possible benefit in respect to the identification of hybridisation and reticulation events [44]. Generally, we followed certain precautions and based our work on data published previously examining various aspects of ITS evolution in *Boechera* [44]. With the availability of cheaper and faster sequencing methods, it is possible to make use of other sequencing-based marker systems [46]. One such system is the nuclear single-copy genes. For example several studies focused on the 1^st^ or 2^nd^ intron of the developmentally important gene *LEAFY* and its orthologues in taxa other than *Arabidopsis*. Successful analyses have been carried out in the genus *Ophrys* [47], the Gnetales [48], and Rosaceae [49]. Also intronic regions of *PISTILLATA* [50], [51] were successfully used. Exonic regions of AGAMOUS1 and PHYTOCHROME B were applied in Arecaceae [52]. The orthologues of the *Arabidopsis* loci *At2g32520*, *At2g13360*, and *At5g23290* were suggested as useful markers within the Brassicaceae [53]. Comparing different studies dealing with single-copy genes, the outcome varies from finding less informative characters than in the ITS to far more information [34]. The amount of genomic regions usable for phylogenetic reconstructions is immense. Theoretically, any type of single-copy gene with suitable amount of variation can be used.

Combining and comparing the results of studies of ITS and single-copy genes may help in forming hypotheses on relatedness and evolution of the taxa investigated. Single-copy genes may provide better resolution due to possible higher variation while the ITS may offer insight into reticulate processes.

With the combination of ITS and two novel single-copy gene markers (orthologues of *At3g18900* and *At2g25920*), in comparison to previous chloroplast DNA analyses, we aim to shed some light on hyper-diverse *Boechera* and we want to (a) recognize evolutionary lineages comprising defined taxa or species groups, (b) describe reticulate evolutionary patterns taking also data on ploidy level and reproductive mode into account, and (c) identify ancestral and basal lineages in putative Pleistocene refuge areas. We believe that the obtained knowledge is of help to other studies currently focused on *B. stricta* (e.g. [18], [19]) as sexual, diploid taxon if these should be extended into other lineages as well.

## Results

### *ITS* Type Definition and Alignment

In total, 289 ITS types were detected among the 911 *Boechera* accessions representing 74 taxa (sensu Al-Shehbaz and Windham [14]). 39 ITS types were shared by several species, 30 were specific to one species and 220 were singletons. Of the 39 ITS-types shared by several species, 21 were mainly found in one taxon. Of the 74 taxa included, 54 were described as sexuals while 13 were described as apomictic hybrids and seven as sexual or apomictic (sensu Al-Shebaz and Windham [14]. The 39 taxa which were not sampled in this study include those known from the type specimen, very rare, or recently described as apomictic hybrids [14].

Sixty nine ITS sequences (also defined as different ITS types, see materials and methods) contained ambiguous sites in forward and reverse sequence and were therefore assumed to be the result of the presence of multiple ITS types in the same individual indicating a hybrid origin; only three of them occurred twice, all others were singletons (Table S4 and grey shaded accessions in Figure S1). ITS types with ambiguous sites (indicating hybridization) were found in the apomictic or sexually and apomictic reproducing taxa *B. microphylla*, *B. pauciflora*, *B. inyoensis*, *B. lemmonii*, *B. inyoensis*, *B. lyallii*, *B. pendulina*, *B. “divaricarpa”* and *B. lignifera*. However, also taxa described as only sexually reproducing carried ITS types with ambiguous sites.

A comprehensive table (taxa, their ITS types, corresponding Genebank accession numbers, voucher information, as well as cpDNA haplotypes if present) can be found in the supplement (Table S2).

The ITS alignment including all sequence types detected in this study as well as the outgroup had a total length of 725 bp. Of these, 362 characters were constant, 154 characters were variable but parsimony-uninformative, and 209 characters were parsimony informative (Table S3).

### Phylogenetic Reconstructions Based on ITS

The Bayesian analysis of all ITS types identified in this and previous studies was stopped when the standard deviation of split frequencies had reached 0.023 which is not the generally accepted standard deviation of split frequencies at which a Bayesian analysis may be stopped. However, [54] recognized difficulties which arise from datasets which contain very closely related individuals, such as phylogeographic datasets. Often these datasets contain polytomies which mislead the Bayesian analysis. By running the program AWTY [55], it was confirmed that the runs had converged sufficiently (graphs can be obtained upon request) although the commonly accepted standard deviation of 0.01 had not been achieved. The likelihoods of the individual runs were –7317.13, –7317.43, and –7317.39, respectively.

The Bayesian analysis separated genera of the tribe Boechereae as well as *Boechera* species into several major lineages (Figure S1). *Cusickiella*, *Pennellia* (*Pennellia* a member of the tribe Halimolobeae), *and Anelsonia* were sister to an unresolved group containing *Boechera repanda*, *Sandbergia/Polyctenium*, *B. canadensis,* and the large group of mainly western North American *Boechera species* and the eastern *B. laevigata*/*B. missouriensis* group. The *laevigata*/*missouriensis* group was sister to the western North American *Boechera* species. Like the other relationships, this was supported by a posterior probability greater than 0.5.

Relationships among the mainly western North American species remained unresolved. 38 lineages containing more than one ITS type were identified, most of them without significant posterior support. 132 ITS types were not assigned to any lineage and remained unresolved on a polytomy together with the 38 lineages. Well-supported lineages had a tendency to be specific to one or or a small number of species (upper part of the tree, Figure S1).

When cpDNA haplotypes (data taken from [8]) are plotted onto the ITS phylogeny, no significant congruent patterns were observed (for cpDNA haplotype identity see table S2).

### *At2g25920* Alignment and Phylogenetic Reconstruction

The orthologue of *At2g25920* (referred to *At2g25920* as in the following) was successfully amplified and sequenced for 210 accessions representing 40 *Boechera* taxa and five accessions from *Sandbergia* and *Polyctenium*. The total length of the *At2g25920* alignment was 564 bp. Six indels were coded separately and added as an 0/1-matrix to the alignment giving it a new total length of 570 bp (Table S5). In all, 331 characters were constant, 79 variable characters were parsimony-uninformative, and 154 characters were parsimony-informative. All six additional gap characters were also parsimony informative.

The Bayesian analysis of *At2g25920* had a final standard deviation of split frequencies of 0.024399. We did not proceed further with the analysis for the same reasons as described above for the ITS analysis. The best likelihoods of the four runs reached –3471.12, –3471.65, –3472.6, and –3471.65. *Boechera. laevigata* was sister to all other taxa including *Sandbergia*. *Boechera perstellata*, *B. repanda*, and *B. canadensis* were placed on a polytomy together with a lineage comprising the remaining *Boechera* specie as well as their (in this analysis) sister *Sandbergia* (Figure S2). A group of admixed taxa was sister to a large group of *Boechera* species. Relationships within this large group were unresolved. We found 25 lineages containing more than one accession. In total, 38 sequence types were not assigned to any lineage. As in the phylogenetic reconstruction based on the ITS, some species-specific lineages were recovered. Taxa representing own lineages were *Boechera microphylla, B. lemmonii, B. cobrensis, B. glaucovalvula, B. schistacea, B. stricta, B. suffrutescens* (shared with one individual of *B. constancei* and *B. koehleri*), *B. rectissima* (together with one *B. arcuata* accession) and *B. crandallii.* Other lineages contained not only one taxon but represented several taxa.

### *At3g18900* Alignment and Phylogenetic Reconstruction

The orthologue of *At3g18900* (referred to *At3g18900* as in the following) was successfully amplified and for 111 accessions representing 34 *Boechera* taxa and three accessions from *Sandbergia* and *Cusickiella*. The *At3g18900* alignment had a total length of 631 characters. Four gaps were coded as a 0/1 matrix increasing the alignment length to 635 bp (Table S6). A total of 494 characters were constant, 55 were variable and parsimony-uninformative, and 82 were parsimony informative. All four additional gap characters were also parsimony informative.

The Bayesian analysis of *At3g18900* reached a final standard deviation of split frequencies of 0.012445. The best likelihoods for run 1 to 3 were –2241.58, –2241.77, and –2242.72, respectively. The resolution of the tree based on *At3g18900* was higher than in the analysis of the ITS. The phylogenetic reconstruction based on *At3g18900* placed *Boechera repanda* as sister to the remaining *Boechera* accessions and *Sandbergia*. The *Boechera* accessions were split into two groups, *Sandbergia* being sister to one of the groups (Figure S3). Relationships in the group being sister to *Sandbergia* remained unresolved. On the polytomy a specific lineage for *B. lemmonii*, *B. lyallii* and *B. stricta* was found. Another lineage contained accessions representing *B. koehleri, B. breweri, B. sparsiflora* but also *B. microphylla* and *B. macounii*. *At3g18900* sequence types specific to *B. crandallii* and *B. cobrensis* were exclusively found within the *Boechera* lineage being sister to *Sandbergia*. However they were found to be unresolved on the polytomy among accessions representing *B. perennans, B. pallidifolia, B. williamsii, B. rectissima, B. gracilenta, B. lignifera, B. pallidifolia,* and *B. schistacea*.

*Boechera missouriensis* and *Boechera laevigata* were sister to the second clade recovered by the *At3g18900* based phylogenetic reconstruction. *Boechera canadensis* was sister to the remaining *Boechera* accessions in the second clade. *Boechera rigidissima, B. constancei* and *B. suffrutescens* formed a sister clade to a polytomy from which five lineages arose. The first lineage was a single *B. macounii* accession, the second lineage contained *B. pendulina* (six accessions), *B. fendleri* (five) and *B. perennans* (two accessions). The third lineage contained six accessions representing *B. formosa*, two *B. pulchra* accessions and one *B. lincolnensis* accession. The fourth lineage contained one accession of each *B. inyoensis*, *B. fendleri*, *B. shockleyi*, and *B. sparsiflora*. The fifth lineage contained two *B. pygmaea* accessions.

### *ITS* Network Analysis of the Alignment Comprising 24 Ancestral ITS Types

For a better understanding, the most simplified network obtained for the reduced dataset of 24 ITS types is described first although it was a consequence of the more complicated analysis containing 149 ITS types.

**Figure 1:**
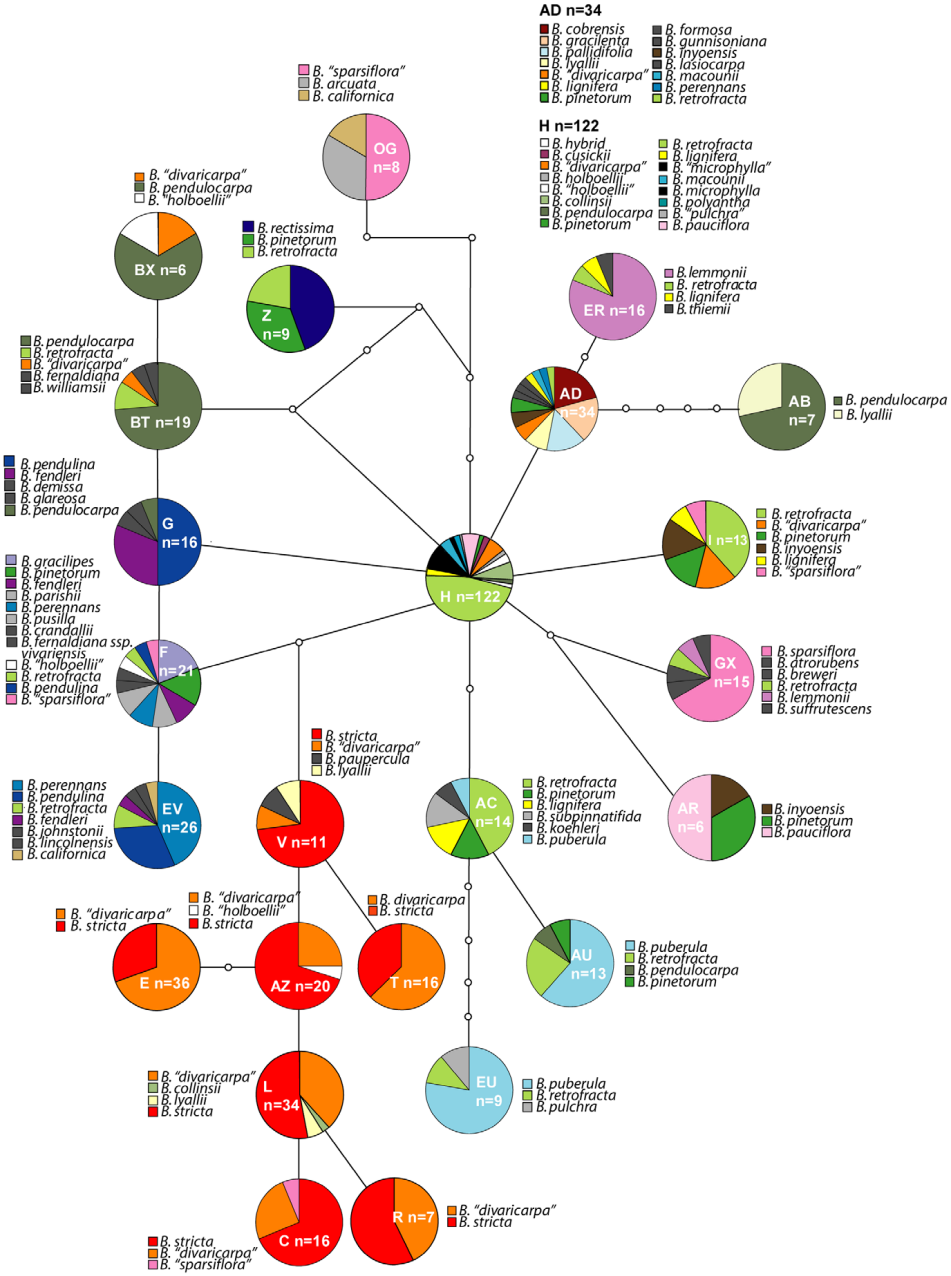
Statistical Parsimony network based on the 24 most frequent ITS types. The nodes of the network have been replaced by pie diagrams depicting ITS type sharing by different taxa; taxa which occurred only once or twice all over the network are coloured in grey (light gray 1, dark grey 2); the overall frequency of the ITS types is given within the pie charts.

The 24 ITS types, on which the most simplified network was based, were selected according to their frequency (>5), their position in the Bayesian analysis (on the large polytomy or basal in a lineage containing several ITS types) and their central position in the network based on 149 ITS types. Those selection criteria were applied in order to get an easier insight into the relationship only of ancestral ITS types in a form which also allowed the presentation of ITS type sharing among taxa. The network based on those 24 ITS types contained resolved as well as reticulate connections (Figure 1). ITS type H (most frequent and most shared in the dataset) was found to be central to the network. Clockwise the following lineages (in the sense of connections) directly derived from ITS type H could be identified: a lineage comprising the ITS types AD (n = 34), ER (n = 16) and AB (n = 7); a lineage represented by ITS type I only (n = 13); a lineage comprising ITS types GX (n = 15) and AR (n = 6); a lineage comprising ITS types AC (n = 14), AU (n = 13) and EU (n = 9). The relationships between the other lineages were more complex. ITS types F (n = 21), G (n = 16), BT (n = 19) and Z (n = 9) were derived from H but had also reticulate connections among each other. ITS type F was connected to H by one missing ITS type and to G (n = 16). G was also connected to BT (n = 19) and BT to Z (n = 9) by three missing ITS types. Z in turn was connected to H and the lineage of ITS type OG by four mutational steps which closed the circle connection of these ITS types. From the missing ITS type connecting H and F, a lineage comprising ITS types V (n = 11), T (n = 16), AZ (n = 20), E (n = 36), L (n = 34), C (n = 16) and R (n = 7) was derived. This lineage was unique because it was almost specific to *Boechera stricta*, a well defined sexual species, and its putative hybrid *Boechera “divaricarpa*”. The aforementioned ITS type F (n = 21) was ancestor to ITS type EV (n = 26). ITS type BX (n = 6) was derived from ITS type BT.

ITS type sharing was very evident and is shown as pie charts which replace the nodes of the networks. ITS type H was found to be shared by 15 taxa, ITS type AD was shared by 14 taxa and ITS type F was shared by 12 taxa.

In the network reduced to 24 ITS types, *Boechera retrofracta* shared most of the frequent ITS types with decreasing frequency towards the tips of the network. *Boechera stricta* was found in a discrete lineage together with what had previously been identified as *B. divaricarpa* (sensu Rollins [9]) and several *B. lyallii* accessions. *B. pendulocarpa* appeared in two distinct sections of the network as did *B. sparsiflora*. *B. puberula* shared the three connected ITS types AC, AU and EU with increasing frequency towards the tips of the network. *B. lemmonii* dominated ITS type ER. *B. cobrensis*, *B. gracilenta* and *B. pallidifolia* shared ITS type AD in high frequency. *B. lignifera* was sharing five different ITS types. *B. pinetorum* shared 8 ITS types across the network. *B. pendulina*, *B. perennans* and *B. fendleri* mainly shared ITS types F, G and EV of which ITS types F and G had reticulate connections to H and/or BT.

Remarkably taxa like *B. stricta*, *B. lemmonii* or *B. cobrensis* were restricted to one lineage with some outliers which may be unrecognized hybrids or backcrosses of past introgressions. On the other hand, there are taxa like *B. retrofracta*, *B. pendulocarpa, B. lyallii, B. lignifera* and *B. pinetorum* which appear scattered all over the network.

### Network Analysis of the Alignment Comprising 149 Representative ITS Types

**Figure 2:**
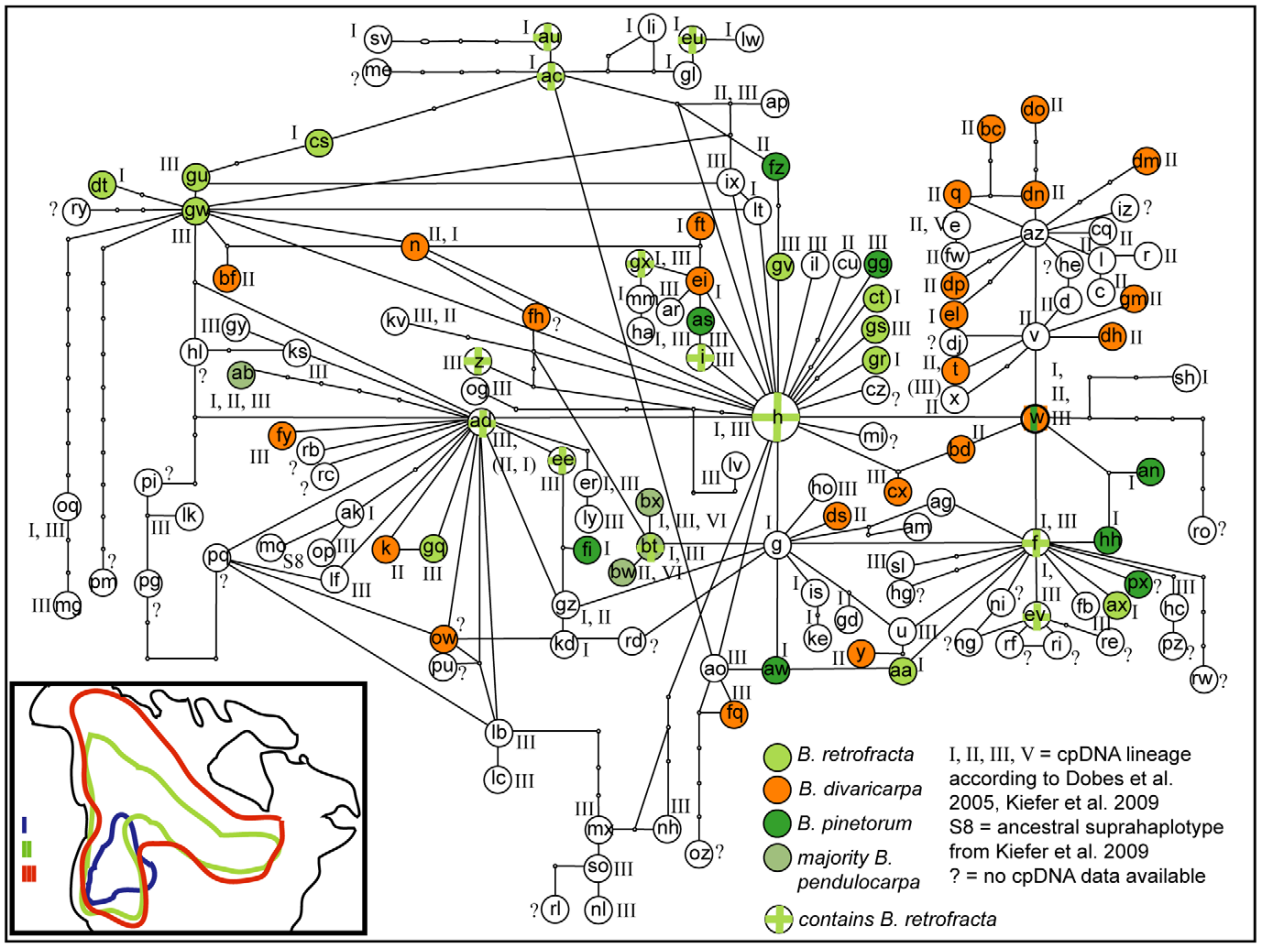
cpDNA haplotype plotted on statistical parsimony network based on 149 ITS types. Roman numbers (I, II, III, V) indicate the lineage of the cpDNA types associated with accessions sharing the ITS type in question: cpDNA lineages are numbered according to [7], [8]; S8 =  ancestral suprahaplotype from [8]; ?  =  no cpDNA data available. It can be concluded that cpDNA lineages and ITS lineages are not congruent in most cases. However, some ITS sequence groups are dominated by a particular cpDNA lineage.

**Figure 3 pone-0036491-g003:**
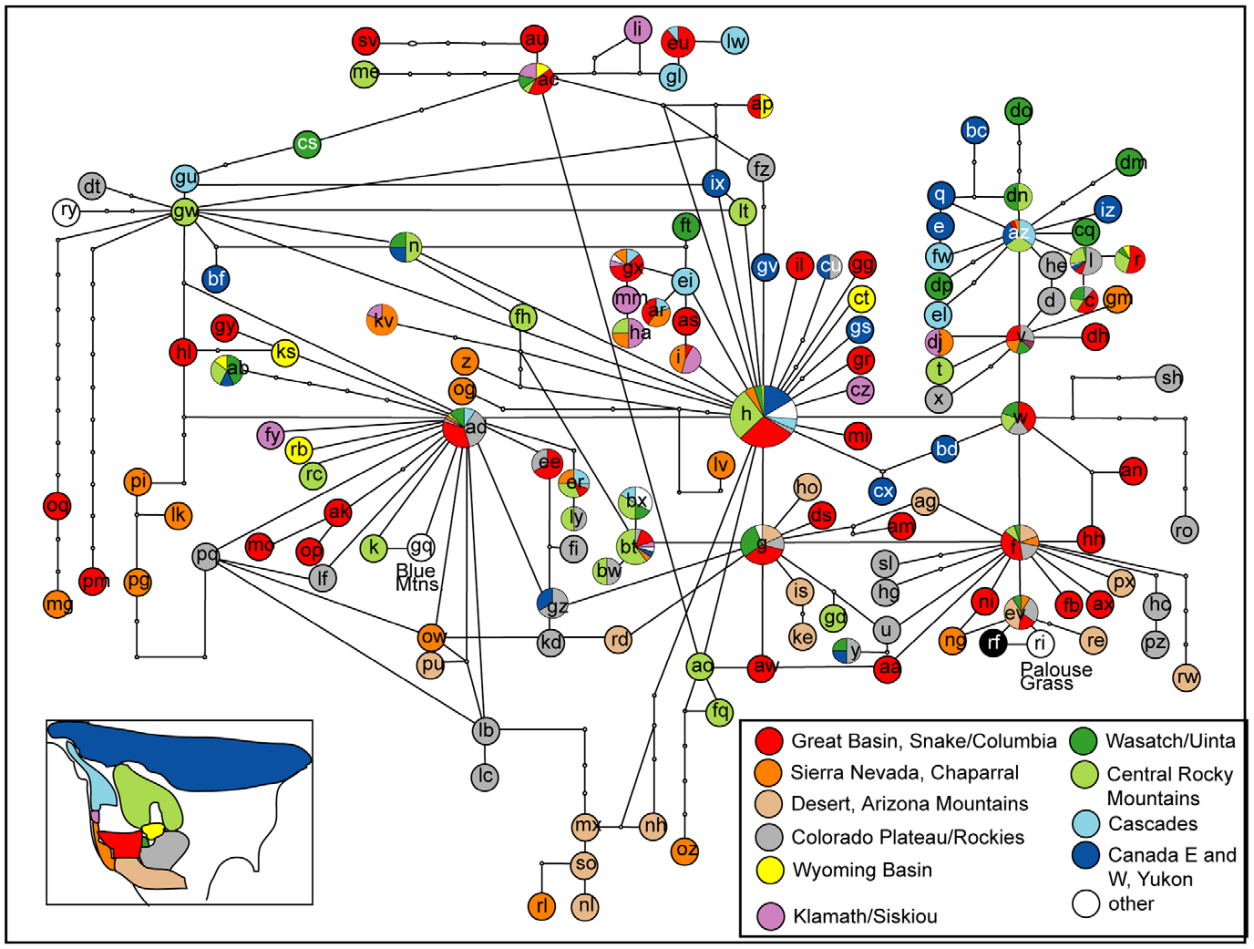
Taxon identity plotted on statistical parsimony network based on 149 ITS types. Sharing of ITS types by taxa was calculated for every ITS type in the network. Pie Charts indicate percentage of taxa sharing the ITS type in question. It is obvious that some taxa are restricted to portions of the network while others share several of the ancestral ITS types (e.g. *Boechera retrofracta*).

**Figure 4 pone-0036491-g004:**
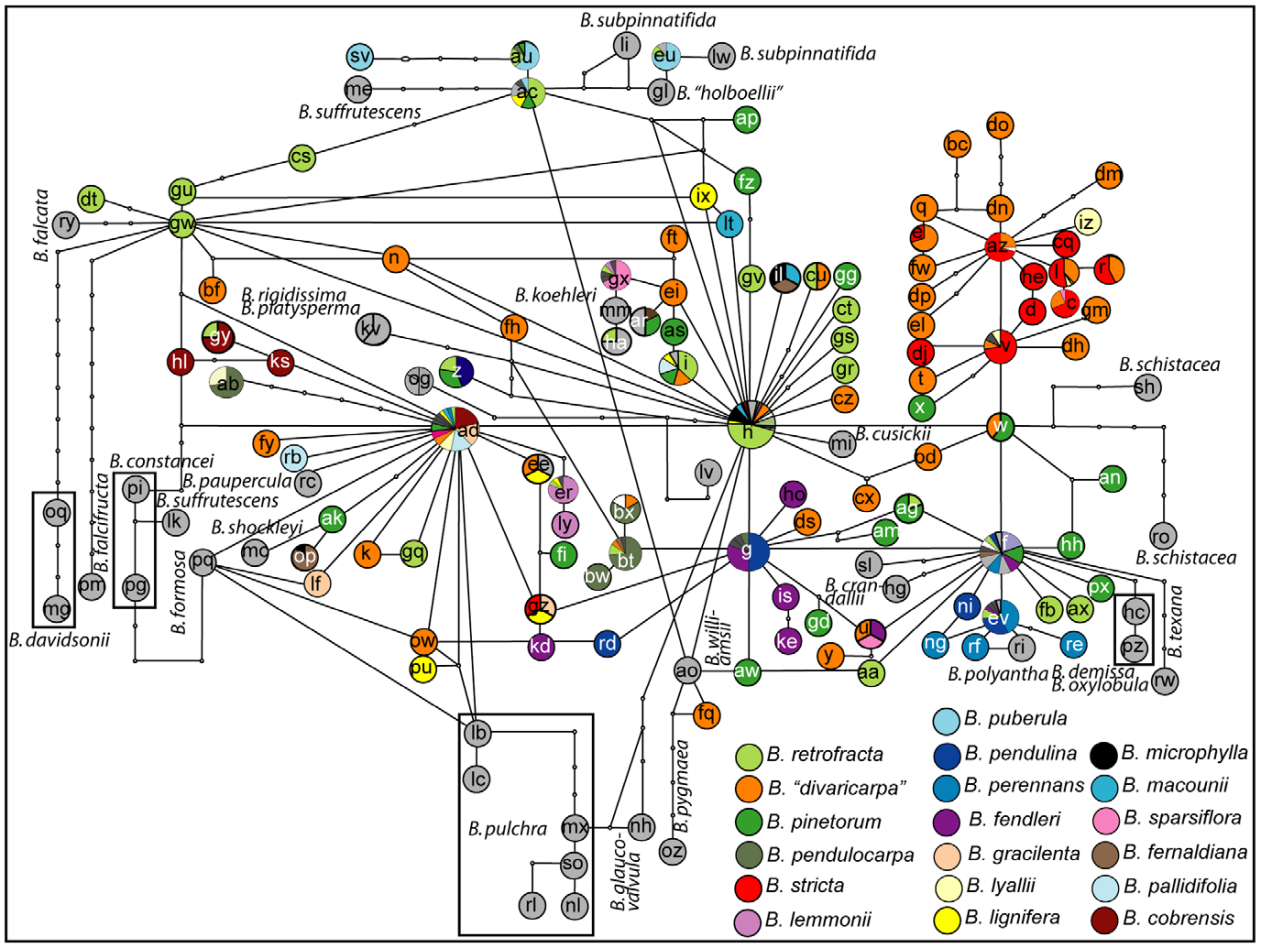
Geographical distribution of accessions plotted on statistical parsimony network based on 149 ITS types. Accessions were sorted into geographical groups (see colour legend of Figure) and distribution of accessions sharing an ITS type over geographical regions was calculated and is displayed as pie charts. There is a trend for ITS type lineages to occur more in particular geographical regions than in others indicating (at least past) gene flow throughout that region.

**Figure 5:**
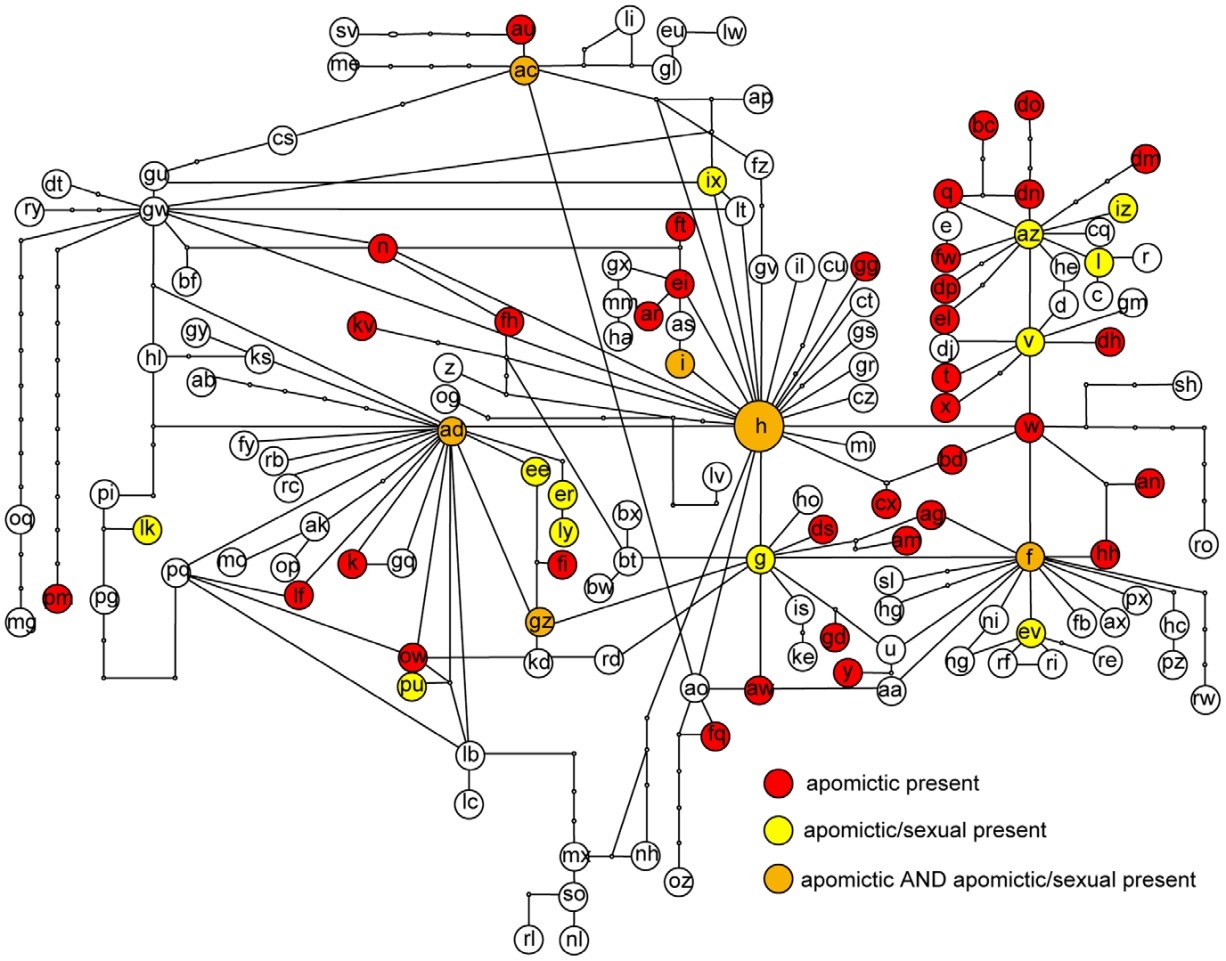
Occurrence of apomictic taxa plotted on statistical parsimony network based on 149 ITS types. There are apomictic, sexual and apomic taxa as well as only sexual taxa reported for Boechera [14]. The graph indicates if the there is an apomictic taxon (red), an apomictic and a sexual/apomictic taxon (orange) or a sexual/apomictic taxon (yellow) among the group of taxa sharing the ITS type of a particular node.

The network reconstruction based on 149 representative ITS types yielded a reticulate network. ITS type H was found to have the highest probability to be at the centre of the network. Other ancestral ITS types were AD, G, F, V, AZ and the previously not included ITS type W. They were all centres for new lineages being derived from them. In order to facilitate interpretation of the network information on cpDNA lineage as defined in [7] and [8], as well as geographic origin, taxon identity and reproductive mode were plotted on the network in three independent Figures (Figures 2, 3, 4, 5).

**Figure 6:**
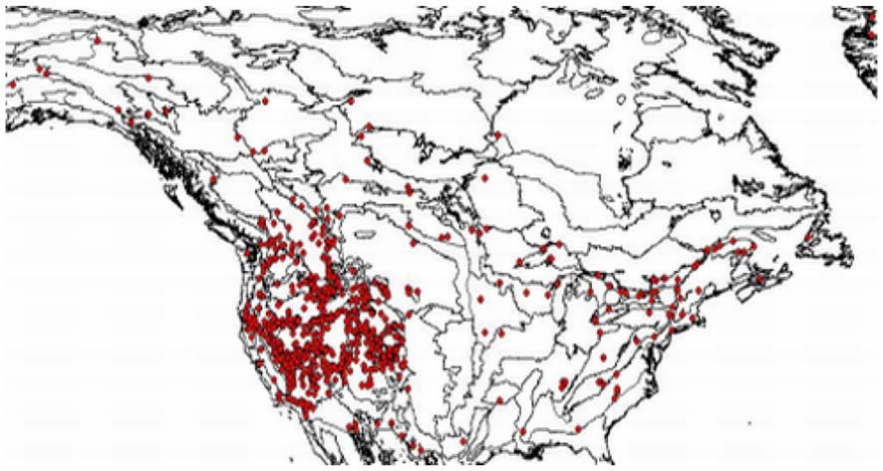
Geographic distribution of accession included in this study. Sampling density is highest in the west of North America where species diversity is highest as well [8].

#### ITS network analysis (149 ITS types) and cpDNA

Plotting cpDNA lineage identity on the network revealed some patterns (opposed to the phylogenetic analysis where due to the large degree of unresolved relationships no obvious pattern could be detected) and indicated possible hybrids which typically had reticulate connections to different ITS types (Figure 2). The ITS lineage comprising ancestral ITS types V and AZ typically carried cpDNA haplotypes belonging to the previously defined lineage II. Individuals in which ITS type H or a derived rare ITS type were found typically carried cpDNA types belonging to lineages I or III. Individuals carrying ITS type AD or derived ITS types typically were found to share cpDNA haplotypes belonging to lineage III (from the individuals for which cpDNA types were present 11 shared cpDNA typed belonging to lineage I, 7 shared cpDNA types from lineage II and 42 individuals carried cpDNA types belonging to lineage III) while individuals carrying ITS type G or its derived ITS types typically carried cpDNA haplotypes from lineage I (from the individuals for which cpDNA types were present 27 shared cpDNA typed belonging to lineage I, 12 shared cpDNA types from lineage II, 12 individuals carried cpDNA types belonging to lineage III and 2 individuals shared cpDNA types from lineage VII). ITS type F was found in individuals carrying cpDNA types belonging either to lineage I or lineage III.

ITS types with reticulate connections had a tendency to carry “non-typical” cpDNA haplotypes. This means accessions with reticulate connections to ITS types carrying cpDNA haplotypes from lineages I or III were found to carry cpDNA haplotypes from lineage II for example or vice versa. Examples for this are ITS types N, AP, BF, K, GZ and Y.

#### ITS network analysis (149 ITS types) and taxon identity

Overlay of the extended network based on 149 ITS types (Figure 3) with taxon identity showed that if a taxon shared an ITS type with a high frequency then it would also be found in derived ITS types (e.g., *B. retrofracta* in ITS type H with derived ITS types CT, GS, GR, GV and I or *B. lemmonii* with ITS type ER and the derived ITS type LY). Furthermore it was evident that some taxa (e.g. *B. pulchra*, *B. davidsonii*, *B. schistacea, B. lemmonii, B. fendleri, B. cobrensis*) were found in particular sections of the network representing small groups (the same as identified in the phylogenetic analysis) while other taxa appeared to be spread across the network (*B. retrofracta, B. “divaricarpa”, B. lignifera*).

#### ITS network analysis (149 ITS types) and geography

For an insight into geographic distribution of ancestral ITS types and their derived lineages, every accessions was assigned to an ecoregion defined by [72]. This approach allowed us to visualize putative geographical patterns in the network (Figure 4).

The lineage containing ancestral ITS types V and AZ showed a south-north split. Accessions carrying ITS type V were collected in the Great Basin and surrounding mountain ranges while accessions which carried ITS type AZ or derived ITS types came from the South Central Rocky Mountains as well as the Cascades and Eastern Canada.

ITS type H was mainly found in the South Central Rocky Mountains and the Great Basin. Other occurrences included the Sierra Nevada, Wasatch/Uinta, the Cascades and Canada. Derived ITS types occurred in the Great Basin/Snake/Columbia and Canada but also in Klamath/Siskiyou, the Wyoming Basin and on the Colorado Plateau. ITS type EI, also derived from H, occurred in the Cascades and was basal to a lineage which reached from the Cascades into Klamath/Siskiyou, the Sierra Nevada and the Great Basin/Snake/Columbia and rarely in the Central Rocky Mountains.

ITS type AD, derived from H, occurred to almost equal proportions in the Great Basin, the Colorado Plateau, and the Colorado Rocky Mountains. However it was also found in Wasatch/Uinta and the Cascades and to a minor extent in the South Central Rocky Mountains, the Sierra Nevada, and Klamath/Siskiyou. Derived ITS types occurred in the Wyoming Basin, the South Central Rocky Mountains, the Great Basin/Snake/Columbia and the Colorado Rocky Mountains/Colorado Plateau. ITS type GZ, which was found in a reticulate position connected to ITS type G as well, occurred in Canada and the Colorado Rocky Mountains/Colorado Plateau. ITS type ER and its derived ITS types were found in the Sierra Nevada, Cascades, the South Central Rocky Mountains and the Colorado Rocky Mountains and Colorado Plateau. To a minor extent ITS type ER also occurred in the Great Basin and Snake/Columbia. ITS type AK and its derived ITS types were restricted to the Great Basin and Snake Columbia. Reticulate ITS types with a relation to ITS type ad occurred on the Colorado Plateau/in the Colorado Rocky Mountains.

ITS types G and F and their derived ITS types can be considered as lineages of dryer habitats. Accessions sharing these ITS types occur in the Sonora, Mojave and Chihuahua Deserts besides the Great Basin/Snake/Columbia, the Colorado Rocky Mountains/Colorado Plateau but also Wasatch/Uinta.

#### ITS network analysis, phylogeny and apomixes

For identifying if apomixis was restricted to certain lineages within the network, apomictic taxa were identified according to [14]. The 20 apomictic or apomictic and sexual taxa included in this study and their corresponding ITS types are listed in table S10. Apomictic taxa were found sharing all ancestral ITS types in the network as well as sharing ITS types derived from them (Figure 5). Also in the phylogeny it is apparent that they occur in all major lineages.

### Comparison of ITS Network Analysis, Geographic Distribution of ITS Types and Lineages Identified in the Phylogenies Based on Single-copy Genes

All three phylogenetic reconstructions yielded phylogenetic trees which were largely unresolved. However, if sufficiently differentiated, accessions belonging to the same taxon were found in discrete lineages arising from the polytomies. Lineages recovered by ITS and both single-copy genes included *Boechera pulchra*, *B. formosa*, *B. breweri-B. koehleri* (also *B. sparsiflora* for ITS and *At3g18900*), *B. suffrutescens-B. constancei* (by the single-copy genes also *B. rigidissima*, *B. lemmonii*, *B. shockleyi-B. inyoensis*, and *B. stricta*. Supported by two out of the three markers were taxon-specific lineages comprising *B. pulchra*, *B. platysperma* (with *B. rigidissima* in the ITS based phylogeny and *B. pygmaea* in the *At2g25920* based phylogeny), *B. cobrensis*, *B. rectissima* (in respect to ITS together with *B. arcuata* and *B. californica*), and *B. oxylobula*.

In some cases an otherwise taxon-specific lineage harboured also an individual belonging to a different taxon. This “misplaced” taxon was then also misplaced by one or both of the two other marker systems applied. This could be indicative of original misidentifications.

The information obtained from network and phylogenetic as well as geographic analyses was compiled in a table and used for interpretation of status of lineage sorting and origin of a taxon (e.g., hybrid origin) (Table S8). For 56 out of the 74 taxa included in this study, some conclusions in respect to lineage sorting or hybrid status could be drawn. Only for a minority of taxa lineage sorting was complete in respect to ITS data (*B. davidsonii*, *B. glaucovalvula*, *B. lemmonii*, *B. platysperma*, *B. pulchra* and its subspecies sensu Rollins [9] (which are now distinct species), *B. repanda*, *B. stricta*). Other taxa were in a lineage separate from the ancestral ITS types as well. However, in the derived lineages a secondary diversification had happened, so lineage sorting within the sublineage was incomplete (*B. breweri*, *B. breweri* ssp. *shastaensis*, *B. koehleri*, *B. sparsiflora* sharing ITS type GX; *B. suffrutescens*, *B. constancei*, *B. formosa*). A number of taxa shared the ancestral ITS types and sometimes showed signs of beginning differentiation by displaying specific ITS types derived from their ancestral ITS type (*B. cobrensis*, *B. crandallii*, *B. fendleri*, *B. collinsii*, *B. lasiocarpa*, *B. pendulocarpa*, *B. microphylla*, *B. pallidifolia*, *B. pendulina*, *B. perennans*, *B. puberula*, *B. gracilenta*, *B. subpinnatifida*). A vast group of taxa appeared in separate independent ITS or single-copy gene lineages or carried several ITS types with ambiguous sites or ITS types with reticulate connections indicating either a putative hybrid origin or present day frequent hybridization (*B. crandallii*, *B. cusickii*, *B. demissa*, “*B. divaricarpa*,” *B. spatifolia*, *B. fernaldiana*, *B. formosa*, *B. gunnisoniana*, *B. pinetorum*, *B. inyoensis*, *B. lignifera*, *B. lyallii*, *B. oxylobula*, *B. paupercula*, *B. polyantha*, *B. pusilla*, *B. rectissima*, *B. schistacea*, *B. texana*, *B. williamsii*).

## Discussion

The taxonomic complexity of *Boechera* has resulted from different modes of reproduction (sexual vs. apomictic), different ploidy levels, and multiple cycles of hybridisation throughout Pleistocene glaciation and deglaciation cycles on a continental scale, all of which caused a vast number of ways for differentiation and diversification, temporally and spatially. The difficulty in understanding relationships within this genus is reflected by its taxonomic history (see (Rollins [9] vs. Al-Shehbaz and Windham [14]). It is obvious that based on comprehensive and continental-wide samplings and utilizing different molecular marker systems, we are now much closer to unravel the evolutionary history of the hyper-diverse *Boechera* clade (e.g. [8], [6]).

### Use of ITS Data in this Study

The ITS is one of the most widely used marker systems. However, it is also critically seen for good reasons when actually applied as only marker system for unravelling a “true” phylogeny. [41] compiled a review pointing out a wide range of putative problems connected to the use of ITS data. In order to add to the understanding of how we analysed the data used in this paper and which precautions we took in order to avoid common problems connected to the use of the ITS, the complications listed in the aforementioned review shall be addressed one by one in the following. The first complication mentioned by [41] is the presence of several rDNA arrays in the genome which may cause comparison of loci which do not share the same evolutionary history which is a basic assumption vital for pyhlogenetic reconstruction. Kantama et al. [13] could show that there is one rDNA array encoding 45S rRNA (this is the array including the 18 S, 5.8 S, 25 S rRNA genes) so the mentioned complication does not apply to our situation though one can critically add that the rDNA arrays have different lengths in different accessions [13]. The second complication listed by [41] is concerted evolution. The different degrees to which concerted evolution can occur may indeed cause difficulties in a true phylogenetic reconstruction where true distances between related groups are important and when chimeric ITS types are formed by recombination of different ITS types. Previous studies in *Boechera* showed that concerted evolution occurs [44]. However, we think that the occurence of chimeric ITS types can also be a benefit because it will shift hybridogenous individuals into reticulate positions in a network analysis. Furthermore pseudogenes which are amplified along with functional copes of the ITS ay be a source of problems [41]. Pseudogenes are often characterized by the presence of large or a big number of indels [41] and we found neither one in our dataset. Typically they would also cause long branches in phylogenetic reconstructions [41] which are also not present in our analyses; on the contrary - our branches are very short. Due to the low number of indels we are also not faced with the problem of difficult alignments which in worst case can make alignments with the outgroup impossible [41]. Contamination of our dataset by for example fungus ITS types was not detected. All ITS types identified for the ingroup yield *Boechera* hits when blasted against genebank. Last but not least homoplasy was mentioned as complication in phylogenetic reconstruction which was attributed to alignment and sequencing problems, compensatory base changes to keep the econdary structure of ITS, paralogy, presence of pseudogenes and incomplete concerted evolution [41]. Since almost every sequence in this dataset was almost entirely covered by two sequence reads which were checked by eye we do not think that we have homoplasy caused by sequencing errors. As stated above the alignments were simple and we can also exclude the presence of paralogues and pseudogenes according to the low sequence divergence and lack of indels. However, we are left with incomplete concerted evolution which we found to be beneficial in our dataset. Also we were not aiming at reconstructing a solid phylogeny based on ITS data which due to the lack of sequence variation (young age of the genus) is almost impossible. Hence homoplasy is not deeply relevant to this dataset as it would be to a true phylogenetic dataset.

Alvarez and Wendel [41] suggest that as a minimum precaution ITS PCR products should be cloned and subsequently several clones should be sequenced per accession. This approach was followed by [44], however in a study with widely different aims (identification of parents of a hybrid) than the current study. If in the current study sequencing revealed ambiguous sites indicating the presence of more than one ITS type within an individual, the resulting sequence including ambiguous codes got designated as a separate ITS type, making it a “collective” ITS type for the individual in question. Analyses were run including and excluding these ITS types with no difference in the results (data not shown).

### Phylogenetic Reconstructions

In this study, 74 out of the currently accepted 113 *Boechera* taxa were analyzed in respect to ITS along with five out of the seven (including *Borodinia*) other genera of the Boechereae. Single-copy gene data (the orthologues of *At2g25920* and *At3g18900*) were obtained for as many accessions as possible and phylogenetic trees were reconstructed by Bayesian analysis for all three datasets (Figures S1, S2, and S3).

Common features of the three phylogenetic reconstructions were that (a) eastern and western North American *Boechera* taxa were separated (as in [6]) apart from one taxon (see point b), (b) *B. repanda* was placed outside the western North American *Boechera* among the other taxa of the Boechereae and suggests the taxonomic status of this species has to be revised and it should perhaps be placed in an independent genus, (c) trees were largely unresolved within the western North American *Boechera*, and (d) for a number of taxa ITS/single copy-gene types specific to one taxon/a group of taxa clustered together.

Unresolved trees, as we found them (see point c and Figures S1, S2, and S3), are a common phenomenon, usually regarded as experimental failure [57]. However, the lack of resolution indicates that the time span between branching events was extremely small, and that is useful information. If a polytomy is caused by truly simultaneous cladogenesis, it is referred to as a hard polytomy, whereas a polytomy caused by too few characters and superimposed substitutions it is called a soft polytomy [58]. Since the polytomy was recovered for ITS and *At2g25920* in parallel and also in the phylogenetic reconstruction based on *At3g18900* internal branches are shorter than terminal branches the polytomy detected in our dataset can be regarded as a hard polytomy showing the rapid early cladogenesis in *Boechera*. Many of the lineages we recovered were species-specific. Therefore, the shape of the trees cannot only be interpreted as rapid cladogenesis but indeed reflects radiation with increased speciation rates.

“Species specific” lineages on the polytomy recovered in all three datasets were found for *Boechera pulchra*, *B. formosa*, *B. breweri-B. koehleri* (also *B. sparsiflora* for ITS and *At3g18900*), *B. suffrutescens-B. constancei* (by the single-copy genes also *B. rigidissima*), *B. lemmonii*, *B. shockleyi-B. inyoensis*, and *B. stricta*; *B. platysperma* (with *B. rigidissima* in the ITS based phylogeny and *B. pygmaea* in the *At2g25920* based phylogeny), *B. cobrensis*, *B. rectissima* (in respect to ITS together with *B. arcuata* and *B. californica*), and *B. oxylobula* were found to form lineages supported by two out of three markers. Since these lineages are recovered by several markers they can be regarded as well supported. In the light of the ITS type sharing that we found otherwise, these lineages seem to represent well differentiated taxa/groups of taxa.

Relationships among the Boechereae differed among the phylogenetic reconstructions but the application of different marker systems may yield different trees. Also our previous studies as well as other phylogenies [59] have shown that the branches within the Boechereae are short reflecting late Tertiary and Pleistocene differentiation processes.

### Network Analyses of the ITS

It is a common phenomenon, that in intraspecific phylogenies branches with a length of zero are found and being caused by the coexistence of ancestral and derived alleles [60]. In these cases a network analysis should be preferred over a phylogenetic reconstruction [60], [61]. In our phylogeny, the most frequent ITS types with the widest distribution ranges had been found on the polytomy with almost zero branch length. In the network analysis, these ITS types were placed at central positions (making them ancestral sequence types according to coalescence theory [62]) clearly indicating that in *Boechera* ancestral and derived ITS types both are present. Hence, a network reconstruction is the suitable approach in the case of the ITS dataset.

The structure of the network (Figures 2–5) showed furthermore that distances between ITS types were short (often one mutation step only) and some conflicting information was present (that we can interpret as signals of hybridisation) also giving an explanation for the recovered topology of the ITS based phylogeny.

The polytomies in the phylogenetic analyses had already indicated rapid cladogenesis. This finding was further supported by the overall structure of the statistical parsimony networks. From ancestral ITS type H the frequent ITS types G, AD, F (though W), I, AC (through AO) and GX/AR (through EI) were derived and that from them more ITS types had differentiated. Since ITS type H is the most ancestral ITS type, differentiation into these frequent ITS types presents a primary diversification. Differentiation from them can be seen as a secondary round of diversification. In the cases of ITS types EV and GX a tertiary diversification seems to be evident.

### Overlay of ITS Network and cpDNA Data

Previous studies of cpDNA data had revealed at least five (if counting very small basal lineages 7) evolutionary lineages (I to V or VII) and showed that (a) cpDNA haplotype sharing among taxa was a common phenomenon, (b) cpDNA haplotype differentiation predated speciation in many cases, and (c) reticulation and incomplete lineage sorting resulted in complex and rarely species-specific cpDNA haplotype variation [6], [8], [17]. Lineage I had the smallest distribution range and occurred mainly south of the last glaciation. Lineage II was specific to *Boechera stricta* but also contained cpDNA haplotypes shared by *B.* “*divaricarpa*” or *B. lyallii*. Its distribution covered all western North America south of the last glaciation and reached into the north and northeast. Lineage III covered the same range as lineage II, but its distribution reached even further north [8], [7]. The cpDNA network cannot be simply superimposed with the ITS network. It is true that the ITS network also revealed a lineage specific to *B. stricta*, *B. “divaricarpa”* and some *B. lyallii* accessions. But not all of the *B.* “*divaricarpa*” and *B. lyallii* accessions carried cpDNA types from lineage II. This suggests a hybrid origin for these two taxa, which in the case of *B.* “*divaricarpa*” has been shown before [7] and was suggested for *B. lyallii* [13].

ITS type H was found in accessions previously determined to carry cpDNA haplotypes of lineages I and III. However, also some accessions carrying cpDNA types from lineage II were found. The latter can be explained by introgression into the “*B. stricta* ITS lineage” and subsequent backcrossing. The presence of cpDNA lineage I and III in individuals sharing ITS type H can be explained by the cpDNA lineage separation predating speciation. When differentiation proceeded in the direction of ITS type G, cpDNA types of lineage I were sorted into this ITS lineage. A long-term stable population for cpDNA lineage I was inferred for the Great Basin, Snake Columbia, and the Colorado Plateau [8]. This overlaps with the distribution range of ITS type G which is found in the eastern Great Basin and the adjacent Wasatch/Uinta range as well as the Colorado Plateau and deserts towards the south.

Differentiation from ITS type H to ITS type AD carried cpDNA types of lineages I and III with it (but mainly III) but here no clear overlap with the cpDNA network was possible.

ITS type F which showed reticulate connections to ITS types G and H (through W) was shared by individuals carrying cpDNA haplotypes of lineages I and III. The reticulate position of ITS type F indicates an ancestral hybridisation event. Diversification from ITS type F happened in the Great Basin/Snake Columbia and on the Colorado Plateau/the Colorado Rocky Mountains. From cpDNA data an admixture of lineages (or long-term stable population) had been inferred for the Colorado Plateau and the Colorado Rocky Mountains [8]. The reticulate positioning of ITS type F may be an indicator of this admixture (hybridisation).

ITS types in reticulate positions in the network like N, BF, FZ or GZ belonged to individuals carrying cpDNA haplotypes being part of cpDNA lineage II. This indicates introgression of *B. stricta* or another cpDNA lineage II carrier like *B. lyallii* or *B. divaricarpa* sensu Rollins [9] into other taxa and underlines that accessions in conflicting positions in the network can indeed by hybrids.

### Overlay of ITS Type Network and Taxon Identity

Both network analyses performed on ITS datasets with 24 and 149 ITS types, respectively, were overlaid with taxon identity. ITS type sharing was found to be common especially in the centre of the network. Sharing of cpDNA types has been reported to be caused by incomplete lineage sorting especially in young species groups [60]. Another reason can be hybridisation [61]. Based on cpDNA, the split between *Arabidopsis* and *Boechera* has been estimated to have happened 6 to 15 million years ago [7]. The split between the most divergent cpDNA lineages was estimated to have occurred 1 to 3 million years ago [7]. Hence *Boechera* can be seen as an evolutionary young group of taxa which explains the large amount of ITS type sharing. However, there were also cases when hybridisation was the more likely source for ITS type sharing. We assumed this to be the case when all but one or few accessions of a taxon grouped together. The outliers were interpreted to be hybrids (or misidentifications). This information has been summarized in table 1 and Table S8.

**Table 1:** Summary of taxa included and conclusions drawn.

number of taxa	74		
number of apomictic and sexual/apomictic taxa according to the taxonomy of (14)	apomictic: 13sexual/apomictic: 7	number of putative apomictic or sexual/apomicticaccessions with indication for hybridisation(ambiguous sites in ITS)	30 (345) [only apomictic 170 (345) sexual/apomictic 175 (345)] 13 of the onlyapomictic have ITS types with ambiguoussites
number of sexual taxa accordingto the taxonomy of (14)	54	number of sexual accessions with indicationfor hybridisation (ambiguous sites in ITS)	39 out of 599
number of taxa where lineagesorting is assumed to be complete(assumptions made for 25 taxa)	9		
number of taxa where lineagesorting is incomplete (assumptionsmade for 25 taxa)	16		
indication for hybridisation basedon ambiguous ITS sites andplacement in network orphylogenies (assumptions made for54 taxa, Table S8)	31		

Information summarized in numbers; for details see Table S8.

It was remarkable that *B. retrofracta* was found to be sharing every frequent ITS type apart from the ones dominated by *B. stricta*. This omnipresence of *B. retrofracta* may indicate that it probably represents the closest taxon to the ancestor of the genus by which the derived ITS types were carried into new geographic regions where differentiation into new taxa took place. There is also the possibility that misidentification of some rare or not yet described hybrids as *B. retrofracta* may artificially increase the occurrence of *B. retrofracta* across the network. However, in most cases more than one *B. retrofracta* individual is found in the major nodes of the network making it unlikely that these are all misidentifications.

### Overlay of ITS-type Network with (phylo-)Geography and Diversification

The quaternary ice ages have shaped distribution and diversity of biota in North America until the present day [63]. The repeated cycles of warm periods with retreating glaciers and cold periods with glacial expansion offered changing environments in which species could either migrate into new habitats or were forced into glacial refugia [64]. Retreating into a glacial refuge and differentiation within the refuge accumulated diversity [65] while expansion into newly available regions was marked by decreased diversity [66], [67]. However, also a secondary mixture of lineages may lead to an increased diversity [68], [69]. Our analyses of *Boechera* reflect these complex patterns.

#### A well defined early lineage - *Boechera stricta*

The ITS lineage dominated by *B. stricta* (ITS types V, AZ and derived ITS types) showed the origin of the taxon in more southern regions like the Great Basin, Snake/Columbia, the Sierra Nevada and Colorado Rocky Mountains and Colorado Plateau (ITS type V) and the subsequent differentiation into northern regions like the Central Rocky Mountains, the Cascades and also other areas covered or much affected by the ice shields of the quaternary ice ages (for extent of glaciations refer to [70]). With this pattern it was the clearest lineage found in the entire dataset.

#### First differentiation in the great basin and central rocky mountains and source for going north

The current distribution of the ancestral ITS type H and its derived singleton ITS types suggests a long-term persistence in the Great Basin/Snake-Columbia, but possibly also the Sierra Nevada and the Cascades. ITS type H is mostly found in *B. retrofracta*, *B. microphylla* and its varieties defined by Rollins [9] as well as *B. sparsiflora*. Based on the number of differentiated ITS types assigned to these three taxa *B. retrofracta* appears to be the most ancestral taxon while *B. microphylla* seems to be a more recent development.

ITS type H is also the ITS type which was carried North in post-glacial colonization.

#### From the great basin into the desert

Differentiation from ITS type H into ITS type F and derived ITS types was accompanied by a shift in habitats. While a large proportion of individuals carrying ITS type H were found in the Great Basin/Snake/Columbia and the Central Rocky Mountains, individuals carrying ITS type G diversified into *B. pendulina* in the Great Basin and reached into the deserts and with *B. fendleri*. ITS type G also appears in Wasatch/Uinta in different taxa and could indicate a refuge in these mountains.

#### Meeting in the colorado plateau resulting into speciation

ITS type F is in a reticulate position between ITS types H (through W) and G. It occurs in the Great Basin, Snake/Columbia, the Colorado Plateau, the deserts and the Sierra Nevada as well as the Central Rocky Mountains and Wasatch/Uinta. The number of derived ITS types suggests diversification in the Great Basin/Snake/Columbia and on the Colorado Plateau. ITS type W is at the base of the lineage comprising mainly *B. stricta*. cpDNA haplotypes belong to lineage I, II and III. The position of ITS type F suggests a hybrid origin. As discussed above, the admixture of individuals carrying ITS type G or W may have happened on the Colorado Plateau/in the Colorado Rocky Mountains. The admixture resulted into further differentiation into *B. gracilipes* and *B. perennans*. Further migration of individuals carrying ITS type F was also accompanied by speciation like *B. parishii* in the Sierra Nevada.

#### Migrating west and north from the colorado plateau

ITS type AD is mainly found in the Great Basin/Snake/Columbia and on the Colorado Plateau/in the Colorado Rocky Mountains. The considerable number of derived ITS types suggests long-term persistence and diversification in these regions. Differentiation into ITS type AD was accompanied by speciation into at least *B. gracilenta*, *B. pallidifolia* and *B. cobrensis*. Differentiation on the Colorado Plateau/in the Colorado Rocky Mountains happened into *B. formosa* and *B. pulchra*. ITS type ER and its related ITS types are derived from ITS type AD. They stand for speciation into *B. lemmonii* and migrating further North.

#### Diversification in klamath/Siskiyou

*B. sparsiflora* has its own differentiated lineage with the frequent basal ITS type GX. ITS type GX was derived from ITS type H through ITS type EI. The frequent ITS type GX was found to be the ancestral ITS type in a secondary diversification event which seems to have taken place in Klamath/Siskiyou, a region known to be a glacial refuge [63]. Also ITS type EI was found in the Cascades, a region which is known to have harboured further refugia [63]. It is possible that ITS type H arrived with *B. sparsiflora* to the Klamath/Siskiyou refuge where it gave rise to ITS type GX which was the most common ITS type in the gene pool which underwent a secondary diversification into other taxa such as *B. breweri* or *B. koehleri*.

### Apomixis as a Consequence of Reticulation?

*Boechera* is unusual because it shows apomixis also at the diploid level [13], [14]. Hence while superimposing the ITS networks and the phylogeny with taxon identity we also recorded if a taxon had been reported as (partially) apomictic (data taken from [14]). This revealed that apomictically or sexually and apomictically reproducing taxa are found sharing all ancestral ITS types. In *Ranunculus* it was suggested that apomictic lineages arise either through hybridisation or by facultative sexual reproduction in apomicts [71]. For *Boechera* a hybrid origin of the apomicts has been assumed [31]–[33]. However, it was also shown that apomixis in *Boechera* is facultative [17]. The high number of triploid hybrids in *Boechera* suggests the involvement of hybridisation processes in the establishment of apomictic reproduction while the data of [17] support the possibility that apomixis once having been established spread into sexual populations by the introgression of occasionally reproducing apomictic individuals. Our data suggest that there is not an occurrence of a single apomictic lineage but that apomixis in *Boechera* is a general old feature of the genus as has been suggested earlier [15].

### Summary

Our extensive analyses of the ITS and the analyses of two novel single-copy gene markers reveal that phylogenetic analyses remain largely unresolved. However, network analyses support that the polytomies detected in the phylogenetic reconstructions indicate rapid diversification. ITS type sharing among taxa shows the young age of some taxa while other taxa seem to be (almost) completely differentiated in respect to the applied marker systems. Superimposing geographical origin of accessions and the ITS network showed a South-North pattern for *Boechera stricta*. Patterns in the remaining part of the network were less obvious but indicate diversification of the most ancestral ITS type in the Great Basin and subsequent diversification into desert/dry habitats on the one hand and in the Great Basin/Snake Columbia Plateau and the Colorado Rocky Mountains and Plateau on the other hand. Northern territories affected by the quaternary ice ages were recolonized mainly from individuals that carried ITS type H or ITS types derived from H. Comparison with cpDNA lineage identity [8] indicated that ITS type lineages and cpDNA lineages partially overlap.

Analyzing the distribution of apomictic taxa across the ITS based network indicates that apomixis is an ancestral feature of the genus *Boechera*.

Although it was not possible to reconstruct a resolved phylogeny application of different marker systems assisted in enhancing the understanding of the complex history of this Brassicaceae genus.

## Materials and Methods

### Plant Material

Leaf material was obtained from herbarium specimens from GH, MO, and DAO, as well as from Eric Schranz (University of Amsterdam, NL) and Thomas Mitchell Olds (Duke University, USA). The corresponding accession details are listed in table S2. In total, we analysed 964 vouchers including outgroup specimens, but we were not able to obtain DNA sequence information for all accessions and all loci (see table S2). This sampling comprises 911 *Boechera* accessions that were analyzed for ITS, and 182 and 107 accessions that were analysed for *At2g25920* and *At3g18900*, respectively. Some of the accessions were used in two earlier comprehensive case studies and had been analysed for ITS sequence variation [44], [10].

A comprehensive chloroplast DNA dataset of the same set of accessions was also utilized herein [8].

The geographic distribution of the samples is shown in Figure 6. Every accession was also assigned to an ecoregion defined by [72] to facilitate geographical comparisons.

### Taxonomic Treatment of Plant Material

Out of the 985 analyzed accessions (this is the total of accessions analyzed for ITS or one of the single-copy genes) 141 had been revised by Ihsan Al-Shebaz (St. Louis, Missouri, USA) and Michael Windham (Duke, Durham, USA). The other vouchers had been determined according to the taxonomy of Rollins [9]. For 73 taxa, it was possible to transfer them to the taxon described in a thorough revision of the genus prepared by [14]. Therefore taxa are labelled with the current name in Figures and tables. *Boechera divaricarpa* was a different case because its current circumscription is much narrower than Rollins [9] concept, and it was not possible for us to determine that. Therefore we write the name as *B.* “*divaricarpa*” to indicate that it is a putative apomictic hybrid but without clear species identity. *Boechera holboellii* is restricted to Greenland [14], whereas Rollins [9] very broadly delimited it to include four varieties (*retrofracta*, *collinsii*, *pendulocarpa* and *pinetorum*) distributed throughout much of North America. If accessions were determined to species level only it was not possible for us to assign them to any of the current taxa. Therefore these individuals are labelled as *B.* “*holboellii*”, and the same approach was applied to all species with varieties according to Rollins [9].

We understand that it is likely that our sexual diploid species may include apomictic forms that had not been recognized as such when the specimen was determined.

### DNA Extraction

Total DNA was obtained from a 0.5×0.5 cm^2^ piece of dried leaf tissue from single individuals. Extraction followed the CTAB method of [73], but modified by grinding of only a 0.5×0.5 cm^2^ piece of dry leaf tissue in 2ml tubes using a Retsch swing mill (MM 200), addition of two units of ribonuclease (RNAse A) to the dissolved DNA, and washing of the DNA pellet twice with 70% ethanol. DNA was finally dissolved in 50–70 µl deionised water or low TE-buffer (Tris-EDTA) for long-term storage.

### PCR Conditions

PCR reactions were performed in a volume of 25 µl containing 1x GoTaq buffer (Promega, Madison, USA), 2 mM MgCl_2_, 5 pmol of each primer, 5 nmol dNTPs (1.25 nmol of each dNTP) and one unit Taq DNA polymerase (GoTaq, Promega), and variable concentrations of template (50 to 400 ng) using a PTC-200 thermal cycler (MJ-Research). Thermal cycling started with a denaturation step at 95°C lasting three min followed by 30 cycles each comprising 30 s denaturation at 95°C, 30 s annealing at 48°C and 1 min elongation at 72°C. Amplification ended with an elongation phase at 72°C lasting 10 min, and a final hold at 4°C.

ITS1 and ITS2 were amplified as described in [10] with PCR products spanning the complete ITS1 and ITS2 as well as the intervening 5.8S rRNA gene in order to prevent the assembly of chimeric ITS types resulting from preferential amplification of different copies of ITS1 and ITS2. PCR products were checked on agarose gels (1% agarose in TAE).

Two genomic sequences from genes known to be single copy in *Arabidopsis thaliana* were chosen as additional markers, the orthologues of *At2g25920* and *At3g18900* respectively both encoding proteins with unknown molecular function.

Primers used for the amplification of *At3g18900* and *At2g25920* were 5′-GCACTTGACCATCTCTTCAGATAA-3′/5′-AGTCCTTCGACGCAAACTG-3′ and 5′-TTTGTTGTTGCATATGGTTGT-3′/5′-TGCTTTACATGACTTGCTCTTA-3′, respectively. Primer sequence information was kindly provided by T. Mitchell-Olds and E. Schranz. PCR products were checked on agarose gels (1% agarose in TAE). PCR products were all purified with the NucleoFastKit (Macherey-Nagel, Germany).

### Cycle Sequencing

Cycle sequencing was done with the DYEnamic ET Terminator Cycle Sequencing Kit (Amersham Biosciences) using the same PCR primers as used for the primary PCR reaction. Samples were resolved in 10 µl Loading Solution and then run on a MegaBace 500 Sequencer. All sequencing was done directly without cloning.

### Alignments and ITS Type Definition

Forward and reverse sequences were assembled, edited by hand and trimmed to a common length. New ITS-types were named following the nomenclature of [44] or were assigned to ITS-types previously recognized (see [44], [10]). An ITS type was defined as any sequence differing from the others by at least one mutation. Sequences with ambiguous sites were also named as an own ITS type since parents could not be determined in most cases by comparison to sequences without ambiguous sites and no prior cloning. Alignments for the phylogenetic analyses were done manually by using the program GeneDoc [74] and followed a previously published alignment [44]. However, new gaps were introduced when needed. ITS type and species identity as given on the herbarium voucher as well as species identity according to the new taxonomy and origin of the individual are listed in Table S2.

The alignments for the *At2g25920* and *At3g18900* sequences were done by eye using the program GeneDoc [74] since sequence similarity was very high and no doubtful positions were found.

### ITS, *At2g25920* and *At3g18900* Phylogenetic Tree Reconstructions

For phylogenetic reconstructions based on the ITS dataset, the alignment was supplemented with sequences of closely related genera obtained from Genebank as well as from our own dataset to determine the relationship of *Boechera* to other genera of the tribe Boechereae (AF146515, AF146514 =  *Cusickiella douglasii*, DQ452059 =  *Anelsonia eurycarpa*, DQ452061 =  *Nevada holmgrenii*, DQ452066 =  *Cusickiella quadricostata* and AJ232927, AJ232926 =  *Halimolobus perplexa* var. *lemhiensis* (hereafter *Sandbergia*), AY230615 =  *Polyctenium williamsiae*, and AF183109 =  *Polyctenium fremontii*). *Capsella rubella* (AJ232913) served as appropriate outgroup.

In a first step, an appropriate model was selected for the Bayesian analysis for each of the ITS and single-copy gene datasets using the program Model Test [75]. Subsequently Bayesian analyses were run using the program MrBayes [76], [77] for all three marker sets under the assumption of the most likely model (nst  = 6, rates equal). For the ITS dataset three runs with four chains each were performed with 40 million generations and a sample frequency of 40,000. For the reconstruction based on the alignment of the *At3g18900* orthologues, four runs were performed and the number of generations was set to 40 million. Trees were sampled every 40,000 generations. The analysis of the *At2g25920* dataset was performed in 4 runs with four chains each for 20 million generations, and trees were sampled every 20,000 generations. For all analyses, the temperature determining the “erosion” of the likelihood landscape was set to 0.01, and 25% of the trees were excluded from the calculation of the consensus (burnin). The success of the analyses was tested by running the program AWTY [55].

### ITS Network Reconstructions

Statistical parsimony network reconstruction was performed by running the program TCS1.21 [56]. Gaps were included as 5^th^ state, and the connection limit was set to 95%. In a first step, a network analysis was run on all identified ITS types. This network proved to be very reticulate, and therefore the analysis needed to be simplified. The phylogenetic analysis had shown that the most frequent ITS type H was unresolved on a polytomy along with 131 other ITS types (among them the other most frequent ITS types F, AD, EV, AC, G, V, AU, AR) and 38 lineages containing two or more ITS types. The frequent ITS types were suspected to be among the most ancestral ITS types since they also had the broadest distribution ranges. Hence, we were interested in their relationship among each other and their relationship to other ITS types on the polytomy and the basal and/or frequent ITS types in the lineages containing more than one ITS type. Following this aim, we simplified the dataset in the following way: (1) we omitted sequences which had larger stretches of missing data at the ends (OM, PA, HK, SB, PP, PR, RH, RX, PW), (2) we omitted 69 additional sequences containing ambiguous sites since we were interested in non-reticulate connections or old hybridisation events (omitted sequences are listed in Table S4), (3) we selected the most basal and most frequent ITS types in the lineages containing more than one ITS type and ITS types representing sublineages if present, and (4) in a final step, all single sequences from the polytomy that had not been excluded by any of the criteria above were added to the alignment. Running the analysis revealed that ITS types CV and PM did not connect to the network with the chosen settings and consequently they were omitted as well. The final resulting alignment included 149 ITS types (Table S7).

The analysis was re-run on this reduced dataset and yielded a reticulate but informative network. The network was edited using the program Adobe Illustrator CS and for a more informative display chloroplast DNA lineages [7], [8], geographical origin (pie charts) and taxon identity (pie charts) were added in the Figures in case of the two latter replacing the nodes of the network.

For visualizing the core network in a maximum simplified form, we picked the central ITS types of the network which were found more than 5 times in the dataset. This yielded an alignment with 24 ITS types in total (Table S9). ITS type sharing among taxa was displayed as pie charts (e.g., an ITS type occurred 12 times in the data set and was found in four taxa; three individuals per taxon shared the ITS type in question; from this it followed that each taxon got a share of 25% of the pie chart representing this ITS type) which were placed on their corresponding nodes in the original network using Adobe Illustrator CS. The number of accessions in which the ITS type was found was also included in the pie charts. Frequent taxa were colour coded, while the ones that occurred only once or twice in the network were coded in two different grey shades (n = 1 dark grey, n = 2 light grey).

Also the occurrence of diploids and apomictic taxa across the network and especially at the ancestral nodes was examined.

## Supporting Information

Table S1 Comparative list of taxonomic classifications of *Boechera* species according to Rollins [9] and [14] along with other synonyms of taxa as recognized by the two authors as well as comments and chromosome numbers as given in the two respective floras.
(PDF)

Table S2 Details of the accessions used in this study; given are the taxon name according to the taxonomy of [14] if possible, geographical information, collector, source herbarium and herbarium accession number as given on the herbarium voucher as well as ITS type, cpDNA type and single-copy gene type with their respective Genebank accession numbers; furthermore it is indicated in which ecoregion [72] the accessions were placed (individuals included in statistical analysis).
(PDF)

Table S3 Alignment of *Boechera* ITS types and sequences representing the outgroup; the alignment is given in nexus format.
(TXT)

Table S4 ITS types which were found to have ambiguous sites and were hence assumed to be found in hybrid individuals; given are the accession number, taxon as on herbarium voucher, ITS type, chloroplast DNA lineage according to [8] and [7] and the main representative taxon which shared this cpDNA type; also it is indicated if hybrids of this taxon have been identified previously according to [14]; ITS types from Table S4 are shaded gray in the ITS phylogeny (Figure S1).
(DOC)

Table S5 Alignment of *At2g25920* orthologue types and sequences representing the outgroup; the alignment is given in nexus format.
(TXT)

Table S6 Alignment of *At3g18900* orthologue types and sequences representing the outgroup; the alignment is given in nexus format.
(TXT)

Table S7 Alignment used for statistical parsimony network reconstruction by TCS based on 149 ITS sequence types given in nexus format.
(TXT)

Table S8 Summary of the phylogenetic tree and network reconstructions for all taxa included in the study; based on the data conclusions were drawn on lineage sorting and hybridisation which are stated in the last column.
(DOC)

Table S9 Alignment used for statistical parsimony network reconstruction by TCS based on 24 ITS sequence types given in nexus format.
(TXT)

Table S10 List of apomictic taxa according to [14] included in this study and ITS types shared by these accessions.
(DOC)

Figure S1 Phylogenetic reconstruction by Bayesian analysis based on ITS types of *Boechera* and several Boechereae taxa and *Capsella rubella* as outgroup; ITS types are labelled with their letter code; if they occur only once the accession number of the taxon carrying the ITS type is given; if the ITS type is shared among several accessions only the taxa are given including the frequency of the ITS type; geographical origin is indicated according to the names of the ecoregions given in [72]; for abbreviations of ecoregion names refer to the table given in the Figure; ITS types shaded in grey include ambiguous sites; ITS types shaded in red were used in the reduced network analysis (Figure 1).
(PDF)

Figure S2 Bayesian analysis of the *At2g25920* dataset; number, taxon identity and geographic origin of the accessions are given.
(PDF)

Figure S3 Bayesian analysis of the *At3g18900* dataset; number, taxon identity and geographic origin of the accessions are given; in this phylogeny it is also indicated in which cpDNA lineage the accessions occurred in a previous study ([6]; blue  =  lineage 1, yellow  =  lineage 2, red  =  lineage 3, pink/lilac  =  lineage 5 and 6, grey  =  central cpDNA type).
(PDF)
