# Supplementary material for: A Continental-Wide Perspective: The Genepool of Nuclear Encoded Ribosomal DNA and Single-Copy Gene Sequences in North American Boechera (Brassicaceae)
Source: PLoS One. 2012 May 14;7(5):e36491. doi: 10.1371/journal.pone.0036491 (PMC3351400; doi:10.1371/journal.pone.0036491)
Supplement: Table S1 — Comparative list of taxonomic classifications of Boechera species according to Rollins [9] and [14] along with other synonyms of taxa as recognized by the two authors as well as comments and chromosome numbers as given in the two respective floras. (PDF) [file pone.0036491.s001.pdf]

**Taxonomie Rollins, 1993**	**Synonym given in Rollins 1993**	**Comment Rollins**	**chromosome number**	**Taxonomie Al-Shehbaz**	**synonyms**	**comment**
Arabis bodiensis Rollins		hybrid involving sparsiflora and fernaldiana var. stylosa according to Rollins but was made a separate taxon as fern. styl does not co-occur		***Boechera bodiensis (Rollins) Al-Shehbaz***	*Arabis bodiensis* Rollins, Contr. Gray Herb. 212: 113. 1982	*B. bodiensis* and *B. falcifructa* are apomictic hybrids containing one or more genomes derived from *B. cobrensis*
Arabis breweri S. Wats. var. austinae (E. L. Green) Rollins	*Arabis austinae E. L. Green*			***Boechera breweri subsp. shastaensis Windham & Al-Shehbaz***	*Arabis austinae* Greene; *A. breweri* var. *austinae* (Greene) Rollins; *A. rostellata* Greene	
Arabis breweri S. Wats. var. breweri	*Arabis epiloboides E. L. Greene; Arabis rostellata E. L. Greene, Arabis breweri var. figularis Jepson*		2n=14	***Boechera breweri (S. Waton) Al-Shehbaz***	*Arabis breweri* S. Watson, Proc. Amer. Acad. Arts 11: 123. 1875; *A. breweri* var. *figularis* Jepson; *A. epilobioides* Greene	*B. rubicundula* (= *B. arcuata* □ *B. breweri*; see Windham and Al-Shehbaz, 2007a
Arabis breweri S. Wats. var. pecuniaria Rollins		shares features with lemmonii but grows in separate range; may hybridize with A. sparsiflora var. subvillosa		***Boechera peirsonii Windham & Al-Shehbaz***	*Arabis breweri* S. Watson var. *pecuniaria* Rollins	
				Boechera calderi (G. A. Mulligan) Windham & Al-Shehbaz	*Arabis calderi* G. A. Mulligan, Rhodora 97: 144. 1996	
Arabis canadensis L.	*Arabis fecunda Michx.; Arabis mollis Raf.*		n=7 2n=14	***Boechera canadensis (Linnaeus) Al-Shehbaz***	*Arabis canadensis* Linnaeus, Sp. Pl. 2: 665. 1753; *A. falcata* Michaux (1803), not A. Richard (1847), nor (Turczaninow) Berkutenko (1976); *A. hirsuta* (Linnaeus) Scopoli var. *ovata* (Pursh) Torrey & A. Gray; *A. mollis* Rafinesque (1817), not *A. mollis* Stevens (1812); *A. ovata* (Pursh) Poiret	
Arabis cobrensis M. E. Jones	*Arabis canescens Nutt.*	closely related to Arabis	n=7	***Boechera cobrensis (M.***	*Arabis cobrensis* M. E.	

	ex Torrey & A. Gray; NOT Arabis canescens Brocchi; Arabis crypta A. Nels.;	bodiensis and Arabis falcifructa		**E. Jones) Dorn**	Jones, Contr. W. Bot. 12: 1. 1908; *A. canescens* Nuttall (1838), not Brocchi (1823)	
Arabis constancei Rollins	*Arabis suffrutescens S. Wats. var. perstylosa Rollins*		n=7 2n=14	**Boechera constancei (Rollins) Al-Shehbaz**	*Arabis constancei* Rollins, Contr. Gray Herb. 201: 5. 1971; *A. suffrutescens* S. Watson var. *perstylosa* Rollins	
Arabis crandallii Robinson	*Arabis stenoloba E. L. Greene, Boechera crandallii (Robinson) W. A. Weber*	may hybridize with Arabis holboellii var. retrofracta in Colorado	n=7 2n=14 2n=21 in hybrid	**Boechera crandallii (B. L. Robinson) W. A. Weber**	*Arabis crandallii* B. L. Robinson, Bot. Gaz. 28: 135. 1899; *A. stenoloba* Greene	
Arabis cusickii S. Wats.		introgression of Arabis sparsiflora var. subvillosa in Washington		**Boechera cusickii (S. Watson) Al-Shehbaz**	*Arabis cusickii* S. Watson, Proc. Amer. Acad. Arts 17: 363. 1882	
Arabis davidsonii E. L. Greene var. davidsonii	*Arabis brucae M. E. Jones; Arabis cognata Jepson; Arabis lyallii S. Watson var. davidsonii (E. L. Greene) Smiley*			**Boechera davidsonii (Greene) N. H. Holmgren**	*Arabis davidsonii* Greene, Leafl. Bot. Observ. Crit. 2: 159. 1911; *A. brucae* M. E. Jones; *A. cognata* Jepson; *A. davidsonii* var. *parva* Rollins; *A. lyallii* S. Watson var. *davidsonii* (Greene) Smiley	
Arabis davidsonii E. L. Greene var. parva Rollins		miniature edition of var. davidsonii				
Arabis demissa E. L. Greene var. demissa	*Arabis rugocarpa Osterh.; Arabis aprica Osterh. es A. Nels.; Boechera demissa*		2n=21	**Boechera oxylobula (Greene) W. A. Weber**	*Arabis oxylobula* Greene, Pittonia 4: 195. 1900; *A. aprica* Osterhout ex A. Nelson; *A. demissa* Greene; *A. rugocarpa* Osterhout; *Boechera demissa* (Greene) W. A. Weber	
Arabis oxylobula E. L. Greene	*Boechera oxylobula (E. L. Greene) W. A. Weber*	related to Arabis demissa				
Arabis demissa E. L. Greene var. languida Rollins				**Boechera languida (Rollins) Windham & Al-Shehbaz**	*Arabis demissa* Greene var. *languida* Rollins, Rhodora 43: 388. 1941; *Boechera demissa* (Greene) W. A. Weber	closer relationship to *B. spatifolia*

					var. *languida* (Rollins) Dorn	
Arabis dispar M. E. Jones	*Arabis nardina E. L. Greene; Arabis salubris M. E. Jones; Arabis juniperina M. E. Jones*			***Boechera dispar (M. E. Jones) Al-Shehbaz***	*Arabis dispar* M. E. Jones, Contr. W. Bot. 8: 41. 1898; *A. juniperina* M. E. Jones; *A. nardina* Greene; *A. salubris* M. E. Jones	
Arabis xdivaricarpa A. Nels. (pro. sp.)	*Arabis pratincola E. L. Greene; Arabis nemophila E. L. Greene; Arabis drummondii var. pratincola (E. L. Greene) M. Hopkins;*	is of hybrid origin; Arabis drummondii is one parent, the other parent is one of the Arabis holboellii but also Arabis sparsiflora, Arabis pendulina and Arabis perennans seem to have been involved; many varieties of divaricarpa have been described		***Boechera pratincola (Greene) Windham & Al-Shehbaz***	*Arabis pratincola* Greene, Repert. Sp. Nov. Regni Veg. 5: 244. 1908; *A. drummondii* A. Gray var. *pratincola* (Greene) M. Hopkins; *A. nemophila* Greene	arose through hybridization between *B. stricta* and *B. paupercula*

	Turritis brachycarpa Torrey & Grey; Arabis brachycarpa Ruprecht; Arabis oblanceolata Rydb.; Arabis brevisiliqua Rydb.;		n=8,15 2n=14 + 2B, 21, 20 + 2B, 22, 28			

	Arabis divaricarpa A. Nels.; Arabis stokesiae Rydb.; Boechera divaricarpa (A. Nels.) Löve & Löve			**Boechera divaricarpa (A. Nelson) Á. Löve & D. Löve**	*Arabis divaricarpa* A. Nelson, Bot. Gaz. 30: 193. 1900; *A. stokesiae* Rydberg	
	Arabis acutina E. L. Greene; Arabis drummondii var. interposita (E. L. Greene) Rollins; Arabis divaricarpa var. interposita (E. L. Greene) Rollins; Arabis interposita E. L. Greene;			**Boechera acutina (Greene) Windham & Al-Shehbaz**	*Arabis acutina* Greene, Leafl. Bot. Observ. Crit. 2: 82. 1910; *A. drummondii* A. Gray var. *interposita* (Greene) Rollins; *A. divaricarpa* A. Nelson var. *interposita* (Greene) Rollins; *A. interposita* Greene	contains a genome derived from *B. stricta*; the other parent remains uncertain
	A. drummondii A. Gray var. brachycarpa (Torrey & A. Gray) A. Gray; Arabis confinis S. Wats. var. brachycarpa			**Boechera grahamii (Lehmann) Windham & Al-Shehbaz**	*Turritis grahamii* Lehmann, Del. Sem. Hort. Hamb. 7. 1831 and Linnaea 6: Litt. 74. 1831; based on *T.*	arose through hybridization between *B. stricta* and *B. collinsii*

	(Torrey & and A. Gray) S. Wats. & Coulter; Arabis brachycarpa (Torrey & A. Gray) Farw.; Arabis divaricarpa var. stenocarpa M. Hopkins; Arabis divaricarpa var. hemicylindrica Boivin; Arabis patula (Graham) Torrey var. stenocarpa (Hopkins) Farw.;				*patula* Graham, Edinburgh New Philos. J. [7]: 350. 1829, not *T. patula* Ehrhart; *Arabis boivinii* G. A. Mulligan; *A. bourgovii* Rydberg; A. brachycarpa (Torrey & A. Gray) Britton (1894), not Ruprecht (1869); *A. confinis* S. Watson; A. confinis var. brachycarpa (Torrey & A. Gray) S. Watson & J. M. Coulter; A. dacotica Greene; A. divaricarpa A. Nelson var. dacotica (Greene) B. Boivin; A. divaricarpa var. hemicylindrica B. Boivin; A. divaricarpa A. Nelson var. stenocarpa M. Hopkins; A. drummondii A. Gray var. brachycarpa (Torrey & A. Gray) A. Gray; *A. holboellii* Hornemann var. *brachycarpa* (Torrey & A. Gray) S. L. Welsh; *A. patula* (Graham) Torrey, not *A. patula* Weinmann, nor *A. patula* Host; *Boechera brachycarpa* (Torrey & A. Gray) Dorn	
Arabis holboellii Hornem var. collinsii (Fern.) Rollins	*Arabis dacotica E. L. Greene, Arabis divaricarpa A. nels. var. dacotica (E. L. Greene) Boivin*					
	Arabis collinsii fern; Boechera collinsii (Fern.) Löve & Löve;		2n=14	***Boechera collinsii (Fernald) Á. Löve & D. Löve***	*Arabis collinsii* Fernald, Rhodora 7: 32. 1905; *A. holboellii* Hornemann var. *collinsii* (Fernald) Rollins; *A. retrofracta* Graham var. *collinsii* (Fernald) B. Boivin	
	Arabis retrofracta	Arabis holboellii in the	n=7, 9, 14	***Boechera retrofracta***	Arabis retrofracta	

Arabis holboellii Hornem var. retrofracta (Graham) Rydb.	*Graham var. multicaulis Boivin;*<br>*Arabis retrofracta (Graham); Turritis retrofracta (Graham) Hook; Strepthantus virgatus Nutt. ex Torrey & A. Gray; Arabis secunda T. J. Howell; Arabis arcuata (Nutt ex Torrey & A. Gray) A. Gray var. secunda (T. J. Howell) Robinson; Arabis holboellii Hornem var. secunda (T. J. Howell) Jepson; Arabis rhodantha E. L. Greene; Arabis exilis A. nels.; Arabis lignipes A. Nels.; Arabis tenuis E. L. Greene; Arabis kochii Blank; Arabis caduca A. Nels.; Boechera retrofracta (Graham) Löve & Löve*	broad sense consists of a range of biological entities, var. retrofracta being at one end (collinsii is nearby)	2n=14, 21 +1B	***(Graham) A. Löve & D. Löve***	Graham, Edinburgh New Philos. J. 1829: 344. 1829; A. arcuata (Nuttall) A. Gray var. secunda (Howell) B. L. Robinson; A. exilis A. Nelson; A. holboellii (Hornemann) A. Löve & D. Löve var. retrofracta (Graham) Rydberg; A. holboellii var. secunda (Howell) Jepson; A. kochii Blankinship; A. lignipes A. Nelson; A. retrofracta var. multicaulis B. Boivin; A. secunda Howell; A. sparsiflora Nuttall subsp. secunda (Howell) Piper; A. tenuis Greene; Boechera exilis (A. Nelson) Dorn; B. holboellii (Graham) A. Löve & D. Löve var. secunda (Howell) Dorn	
	Arabis polyantha E. L. Greene;<br>*Arabis macdougallii Rydb.;*			***Boechera polyantha (Greene) Al-Shehbaz***	*Arabis polyantha* Greene, Leafl. Bot. Observ. Crit. 2: 80. 1910; *A. macdougalii* Rydberg	
	Arabis consanguinea E. L. Greene;			***Boechera consanguinea (Greene) Windham & Al-Shehbaz***	*Arabis consanguinea* Greene, Pittonia 4: 190. 1900; *A. holboellii* Hornemann var. *consanguinea* (Greene) G. A. Mulligan	arose through hybridization between *B. retrofracta* and *B. fendleri*
Arabis drummondii A. Gray	*Turritis stricta Graham; Arabis stricta Hudson; Strepthantus angustifolius Nutt. ex Torrey & A. Gray; NOT Arabis angustifolia*	in eastern ranges biennial; in western ranges perennial; hybridizes with Arabis pendulian, Arabis holboellii, Arabis laevigata, Arabis		***Boechera stricta (Graham) Al-Shehbaz***	*Turritis stricta* Graham, Edinburgh New Philos. J. 7: 350. 1829; *A. connexa* Greene; *A. drummondii* A. Gray; *A. drummondii* var.	

	Lam.; Arabis confinis S. Wats.; Erysimum drummondii (A. Gray) Kuntze; Arabis connexa E. L. Greene; Arabis oxyphylla E. L. Greene; Arabis albertina E. L. Greene; Arabis drummondii var. connexa (E. L. Greene) fern.; Turritis drummondii (A. Gray) Lunell; Arabis drummondii var. oxypphylla (E. L. Greene) M. Hopkins; Boechera drummondii (A. Gray) Löve & Löve	lignifera, Arabis sparsiflora			*connexa* (Greene) Fernald; *A. drummondii* var. *oxyphylla* (Greene) M. Hopkins; *A. oxyphylla* Greene; *Boechera angustifolia* (Nuttall) Dorn; *B. drummondii* (A. Gray) A. Löve & D. Löve; *Erysimum drummondii* (A. Gray) Kuntze; *Streptanthus angustifolius* Nuttall, not *A. angustifolia* Lamarck; *T. drummondii* (A. Gray) Lunell	
				Boechera elkoensis Windham & Al-Shehbaz		arose through hybridization between *B. platysperma* and *B. stricta*
				Boechera evadens Windham & Al-Shehbaz		
Arabis falcatoria Rollins				***Boechera falcatoria (Rollins) Dorn***	*Arabis falcatoria* Rollins, Contr. Gray Herb. 212: 106. 1982	it is virtually certain that *B. cusickii* contributed at least one genome
Arabis falcifructa Rollins		basla leaves as in Arabis cobrensis		***Boechera falcifructa (Rollins) Al-Shehbaz***	*Arabis falcifructa* Rollins, Contr. Gray Herb. 212: 106. 1982	arose through hybridization between *B. cobrensis* and *B. fernaldiana*
Arabis fecunda Rollins		habit somewhat like that of Arabis fernaliana; indument more similar to Arabis puberula		***Boechera fecunda (Rollins) Dorn***	*Arabis fecunda* Rollins, Contr. Gray Herb. 214: 1. 1984	
Arabis fendleri (S. Wats.) E. L. Greene var. fendleri	*Arabis holboellii Hornem. var. fendleri (S. Wats.); Boechera fendleri (S. wats.) W. A. Weber;*		n=7 2n=14	***Boechera texana Windham & Al-Shehbaz***		
				Boechera fendleri (S. Watson) W. A. Weber	*Arabis holboellii* Hornemann var. *fendleri* S. Watson in A. Gray et al., Synop. Fl. N. Amer. 1: 164. 1895; *A. fendleri*	

					(S. Watson) Greene	
	Arabis porphyrea Wooton & Standley;			***Boechera porphyrea (Wooton & Standley) Windham, Al-Shehbaz & P. Alexander***	*Arabis porphyrea* Wooton & Standley, Contr. U.S. Natl. Herb. 16: 123. 1913	
Arabis fendleri (S. Wats.) E. L. Greene var. spatifolia (Rydb.) Rollins	*Arabis spatifolia Rydb.; Sysimbrium pauciflorum Nutt. ex Torrey & A. Gray; Arabis pauciflora (Grimm) Garke; Boechera fendleri (S. Wats) W. A. Weber subsp. spatifolia (Rydb.) W. A. Weber*	grows in the same habitat as perennans	n=7 2n=14	***Boechera spatifolia (Rydberg) Windham & Al-Shehbaz***	*Arabis spatifolia* Rydberg, Fl. Rocky Mts. 361. 1917; *A. fendleri* (S. Watson) Greene var. *spatifolia* (Rydberg) Rollins; *Boechera fendleri* (S. Watson) W. A. Weber subsp. *spatifolia* (Rydberg) W. A. Weber; *B. fendleri* var. *spatifolia* (Rydberg) Dorn	
Arabis fernaldiana Rollins var. fernaldiana	*Boechera fernaldiana (Rollins) W. A. Weber;*			***Boechera fernaldiana (Rollins) W. A. Weber var. fernaldiana***	*Arabis fernaldiana* Rollins, Rhodora 43: 430. 1941; *A. canescens* Nuttall var. *stylosa* S. Watson; *A. fernaldiana* var. *stylosa* (S. Watson) Rollins	
Arabis fernaldiana Rollins var. stylosa (S. Wats.) Rollins		may be merely a high elevation form of the species				
Arabis fernaldiana Rollins var. fernaldiana	*Arabis vivariensis Welsh; Boechera vivariensis (Welsh) W. A. Weber*			***Boechera fernaldiana (Rollins) W. A. Weber subsp. vivariensis (S. L. Welsh) Windham & Al-Shehbaz***	*Arabis vivariensis* S. L. Welsh, Great Basin Naturalist 46: 263. 1986; *Boechera vivariensis* (S. L. Welsh) W. A. Weber	
Arabis fructicosa A. Nelson		may belong to Arabis xdivaricarpa complex		***Boechera fructicosa (A. Nelson) Al-Shehbaz***	*Arabis fructicosa* A. Nelson, Bot. Gaz. 30: 190. 1900	it is virtually certain that *B. microphylla* contributed at least one genome
				Boechera glareosa Dorn		Specimens of *B. glareosa* originally were identified as *Arabis microphylla* var. *macounii* (= *B. macounii*) by Rollins

Arabis glaucovalvula M. E. Jones			n=7	***Boechera glaucovalvula (M. E. Jones) Al-Shehbaz***	*Arabis glaucovalvula* M. E. Jones, Contr. W. Bot. 8: 40. 1898	
				Boechera goodrichii (S. L. Welsh) N. H. Holmgren	*Arabis goodrichii* S. L. Welsh in S. L. Welsh et al., Utah Fl., ed. 3. 255. 2005	arose through hybridization between *B. retrofracta* and *B. gracilipes*
Arabis gracilipes E. L. Greene	*Arabis arcuata (Nutt. ex Torrey & A. Gray) A. Gray var. longipes S. Wats.; Arabis perennans S. Wats. var. longipes (S. Wats.) Jepson*			***Boechera gracilipes (Greene) Dorn***	*Arabis gracilipes* Greene, Pittonia 4: 193. 1900; *A. arcuata* (Nuttall) A. Gray var. *longipes* S. Watson; *A. perennans* S. Watson var. *longipes* (S. Watson) Jepson	
Arabis gunnisoniana Rollins	*Boechera gunnisoniana (Rollins) W. A. Weber*		n=7	***Boechera gunnisoniana (Rollins) W. A. Weber***	*Arabis gunnisoniana* Rollins, Rhodora 43: 434. 1941	
				Boechera harrisonii (S. L. Welsh) Windham & Al-Shehbaz	*Arabis harrisonii* S. L. Welsh in S. L. Welsh et al., Utah Fl., ed. 3. 256. 2003; *Boechera microphylla* (Nuttall) Dorn var. *harrisonii* (S. L. Welsh) N. H. Holmgren	arose through hybridization between *B. microphylla* and *B. perennans*
Arabis hastatula E. L. Greene				***Boechera hastatula (Greene) Al-Shehbaz***	*Arabis hastatula* Greene, Leafl. Bot. Observ. Crit. 2: 79. 1910	
Arabis hoffmannii (Munz) Rollins	*Arabis maxima E. L. Greene var. hoffmannii Munz*			***Boechera hoffmannii (Munz) Al-Shehbaz***	*Arabis maxima* Greene var. *hoffmannii* Munz, Bull. S. Calif. Acad. Sci. 31: 63. 1932; *A. hoffmannii* (Munz) Rollins	
Arabis holboellii Hornem var. holboellii	*Arabis holboellii var. tenuis f. glabra Böcher; Boechera holboellii*		2n=13 + 2B, 14, 20 + 2B, 21, 21 + 1B			

	(Hornem.) Löve & Löve;					
	Arabis holboellii var. tenuis Böcher; Boechera tenuis (Böcher) Löve & Löve			***Boechera holboellii (Hornemann) Á. Löve & D. Löve***	*Arabis holboellii* Hornemann in Oeder, Fl. Danica 11(Heft 32): 5, pl. 1879. 1827; *A. holboellii* var. *tenuis* Böcher; *Boechera tenuis* (Böcher) Á. Löve & D. Löve	
Arabis holboellii Hornem var. pendulocarpa (A. Nels.) Rollins	*Arabis pendulocarpa A. Nels.*		n=7 2n=14	***Boechera pendulocarpa (A. Nelson) Windham & Al-Shehbaz***	*Arabis pendulocarpa* A. Nelson, Bot. Gaz. 30: 192. 1900; *A. holboellii* Hornemann var. *pendulocarpa* (A. Nelson) Rollins; *Boechera holboellii* (Hornemann) Á. Löve & D. Löve var. *pedulocarpa* (A. Nelson) N. Snow	
Arabis holboellii Hornem var. pinetorum (Tidestrom) Rollins	*Arabis pinetorum Tidestrom*		n=14, 21, 2n=21	***Boechera pinetorum (Tidestrom) Windham & Al-Shehbaz***	*Arabis pinetorum* Tidestrom, Proc. Biol. Soc. Wash. 36: 182. 1923; *A. divaricarpa* A. Nelson var. *pinetorum* (Tidestrom) B. Boivin; *A. holboellii* Hornemann var. *pinetorum* (Tidestrom) Rollins; *Boechera holboellii* (Hornemann) Á. Löve & D. Löve var. *pinetorum* (Tidestrom) Dorn	derived from *B. retrofracta, B. rectissima*, and *B. sparsiflora*
Arabis inyoensis Rollins			2n=21, 23	***Boechera inyoensis (Rollins) Al-Shehbaz***	*Arabis inyoensis* Rollins, Rhodora 43: 457. 1941; *A. holboellii* Hornemann var. *derensis* S. L. Welsh; *Boechera selbyi* (Rydberg) W. A. Weber var. *inyoensis* (Rollins) N. H. Holmgren	contains at least one genome derived from *B. shockleyi*

Arabis johnstonii Munz				***Boechera johnstonii (Munz) Al-Shehbaz***	*Arabis johnstonii* Munz, Bull. S. Calif. Acad. Sci. 31: 63. 1932; *A. hirshbergiae* S. Boyd; *Boechera hirshbergiae* (S. Boyd) Al-Shehbaz	
Arabis koehleri T. J. Howell var. koehleri				***Boechera koehleri (T. J. Howell) Al-Shehbaz***	*Arabis koehleri* T. J. Howell, Fl. Northw. Amer. 1: 44. 1897; *A. arbuscula* Greene; *A. koehleri* var. *stipitata* Rollins	
Arabis koehleri T. J. Howell var. stipitata (Muhlenb. ex Willd.)						
Arabis laevigata (Muhlenb. ex Willd.) Poiret var. burkii Porter	*Arabis burkii (Porter) Small*			***Boechera burkii (Porter) Windham & Al-Shehbaz***	*Arabis laevigata* Muhlenberg ex Willdenow var. *burkii* Porter, Bull. Torrey Bot. Club 17: 15. 1890; *A. burkii* (Porter) Small	
Arabis laevigata (Muhlenb. ex Willd.) Poiret var. laevigata	*Turritis laevigata (Muhlenb. ex Willd.; Arabis lyraefolia DC.; Arabis heterophylla Nutt. ex Torrey & A. Gray; Arabis hastata Eaton; Arabis laevigata var. heterophylla (Nutt. ex Torrey & A. Gray) Farw.*		n=7 2n=14	***Boechera laevigata (Muhlenberg ex Willdenow) Al-Shehbaz***	*Turritis laevigata* Muhlenberg ex Willdenow, Sp. Pl. 3(2): 543. 1801; *Arabis hastata* Eaton; *A. heterophylla* Nuttall; *A. laevigata* (Muhlenberg ex Willdenow) Poiret; *A. lyraefolia* de Candolle	
Arabis lasiocarpa Rollins				***Boechera lasiocarpa (Rollins) Dorn***	*Arabis lasiocarpa* Rollins, Syst. Bot. 6: 58. 1981	
Arabis lemmonii S. wats. var. depauperata (A. Nels. & Kenn.)	*Arabis depauperata A. Nels. & Kenn.*			***Boechera depauperata (A. Nelson & P. B. Kennedy) Windham & Al-Shehbaz***	*Arabis depauperata* A. Nelson & P. B. Kennedy, Proc. Biol. Soc. Wash. 19: 36. 1906; *A. lemmonii* S. Watson var. *depauperata* (A. Nelson & P. B. Kennedy) Rollins	arose through hybridization between *B. lemmonii* and *B. paupercula*
Arabis lemmonii S. wats. var. lemmonii	*Arabis canescens Nutt. ex Torrey & A. Gray*		2n=14	***Boechera lemmonii (S. Watson) W. A. Weber***	*Arabis lemmonii* S. Watson, Proc. Amer.	

	var. latifolia S. Wats.; Arabis latifolia (S. Wats) Piper; Arabis bracteolata E. L. Greene; Arabis kennedyi E. L. Greene; Arabis oreocallis E. L. Greene; Arabis polyclada E. L. Greene; Arabis semisepulta E. L. Greene; Arabis egglestonii Rydb.; Boechera lemmonii (S. wats.) W. A. Weber				Acad. Arts 22: 467. 1887; *A. bracteolata* Greene; *A. canescens* Nuttall var. *latifolia* S. Watson; *A. codyi* G. A. Mulligan; *A. egglestonii* Rydberg; *A. kennedyi* Greene; *A. latifolia* (S. Watson) Piper; *A. oreocallis* Greene; *A. polyclada* Greene; *A. semisepulta* Greene	
Arabis lemmonii S. wats. var. paddoensis Rollins				***Boechera paddoensis (Rollins) Windham & Al-Shehbaz***	*Arabis lemmonii* S. Watson var. *paddoensis* Rollins, Rhodora 43: 384. 1941	
Arabis lemmonii S. wats. var. drepanoloba (E. L. Greene) Rollins	*Arabis drepanoloba E. L. Greene*			***Boechera drepanoloba (Greene) Windham & Al-Shehbaz***	*Arabis drepanoloba* Greene, Pittonia 3: 306. 1898; *A. drummondii* A. Gray var. *oreophila* (Rydberg) M. Hopkins; *A. lemmonii* S. Watson var. *drepanoloba* (Greene) Rollins; *A. oreophila* Rydberg	arose through hybridization between *B. lemmonii* and *B. stricta*
Arabis lyallii S. Wats. var. lyallii	*Arabis oreophila Rydb.; Arabis drummondii var. oreophila (Rydb.) M. Hopkins*					
	Arabis drummondii A. Gray var. alpina S. wats.; Arabis armerifolia E. L. Greene; Arabis densa E. L. Greene; Arabis multiceps E. L. Greene; Arabis drummondii var. lyallii (S. Wats.) Jepson;		2n=ca. 20, 21	***Boechera lyallii (S. Watson) Dorn***	*Arabis lyallii* S. Watson, Proc. Amer. Acad. Arts 11: 122. 1876; *A. armerifolia* Greene; *A. densa* Greene; *A. drummondii* A. Gray var. *alpina* S. Watson; *A. drummondii* var. *lyallii* (S. Watson) Jepson; *A. multiceps* Greene; *A. murrayi* G. A. Mulligan	
Arabis lyallii S. Wats. var. nubigena (J. F. Maybr. & Payson) Rollins	*Arabis nubigena J. F. Macbr. & Payson; Arabis paupercula E. L. Greene; Arabis*			***Boechera paupercula (Greene) Windham & Al-Shehbaz***	*Arabis paupercula* Greene, Leafl. Bot. Observ. Crit. 2: 77. 1910; *A. lyallii* S.	

	microphylla Nutt. ex Torrey & A. Gray var. nubigena (J. F. Macbr. & Payson) Rollins				Watson var. *nubigena* (J. F. Macbride & Payson) Rollins; *A. microphylla* Nuttall var. *nubigena* (J. F. Macbride & Payson) Rollins; *A. nubigena* J. F. Macbride & Payson	
Arabis lignifera A. Nels.	*Boechera lignifera (A. Nels.) W. A. Weber*		n=7, 14	***Boechera lignifera (A. Nelson) W. A. Weber***	*Arabis lignifera* A. Nelson, Bull. Torrey Bot. Club 26: 123. 1899	
Arabis microphylla Nutt. ex Torrey & A. Gray var. macounii (S. Wats.) Rollins	*Arabis macounii S. Wats.; Arabis densicaulis A. Nels.*		2n=15	***Boechera macounii (S. Watson) Windham & Al-Shehbaz***	*Arabis macounii* S. Watson, Proc. Amer. Acad. Arts 26: 124. 1891; *A. densicaulis* A. Nelson; *A. microphylla* Nuttall var. *macounii* (S. Watson) Rollins	arose through hybridization between *B. microphylla* and *B. collinsii*
Arabis microphylla Nutt. ex Torrey & A. Gray var. microphylla	*Arabis tenuicula E. L. Greene*		2n=14	***Boechera microphylla (Nuttall) Dorn***	*Arabis microphylla* Nuttall in Torrey & A. Gray, Fl. N. Amer. 1: 82. 1838; *A. tenuicula* Greene	
Arabis microphylla Nutt. ex Torrey & A. Gray var. thompsonii Rollins		known only on the basis of a single collection		***Boechera cascadensis Windham & Al-Shehbaz***	*Arabis microphylla* Nuttall var. *thompsonii* Rollins, Rhodora 43: 429. 1941, not *Boechera thompsonii* (S. L. Welsh) N. H. Holmgren (2005)	arose through hybridization between *B. microphylla* and *B. paupercula*
Arabis missouriensis E. L. Greene	*Arabis viridis Harger; Arabis viridis var. deamii M. Hopkins; Arabis missouriensis var. deamii (M. Hopkins) M. Hopkins; Arabis laevigata (Muhlenb.) Poiret var. missouriensis (E. L. Greene) Ahles, Arabis viridis var. heterophylla (Farw.) Farw.*			***Boechera missouriensis (Greene) Al-Shehbaz***	*Arabis missouriensis* Greene, Repert. Sp. Nov. Regni Veg. 5: 244. 1908; *A. laevigata* var. *heterophylla* (Nuttall) Farwell; *A. laevigata* var. *missouriensis* (Greene) Ahles; *A. missouriensis* var. *deamii* (M. Hopkins) M. Hopkins; *A. viridis* Harger	

Arabis ophira Rollins		habitally similar to fernaldiana var. stylosa		***Boechera ophira (Rollins) Al-Shehbaz***	*Arabis ophira* Rollins, Syst. Bot. 6: 56. 1981	
Arabis pallidifolia Rollins		related to Arabis crandallii		***Boechera pallidifolia (Rollins) W. A. Weber***	*Arabis pallidifolia* Rollins, Cruciferae of Continental North America 181. 1993; *A. thompsonii* S. L. Welsh; *Boechera thompsonii* (S. L. Welsh) N. H. Holmgren	
Arabisb parishii S. Wats.			n=7	***Boechera parishii (S. Watson) Al-Shehbaz***	*Arabis parishii* S. Watson, Proc. Amer. Acad. Arts 22: 468. 1887	
Arabis patens Sullivant						
Arabis pendulina E. L. Greene var. pendulina	*Arabis nevadensis Tidestrom;*		2n=14	***Boechera nevadensis (Tidestrom) Windham & Al-Shehbaz***	*Arabis nevadensis* Tidestrom, Proc. Biol. Soc. Wash. 36: 182. 1923	
	Arabis diehlii M. E. Jones; Boechera pendulina (E. L. Greene) W. A. Weber			***Boechera pendulina (Greene) W. A. Weber***	*Arabis pendulina* Greene, Leafl. Bot. Observ. Crit. 2: 81. 1910; *A. demissa* Greene var. *russeola* Rollins; *A. diehlii* M. E. Jones; *A. pendulina* Greene var. *russeola* (Rollins) Rollins; *A. setulosa* Greene; *Boechera demissa* (Greene) W. A. Weber var. *pendulina* (Greene) N. H. Holmgren; *B. demissa* var. *russeola* (Rollins) N. H. Holmgren; *B. pendulina* (Greene) W. A. Weber var. *russeola* (Rollins) Dorn	
Arabis pendulina E. L. Greene var. russeola (Rollins) Rollins	*Arabis demissa E. L. Greene var. russeola Rollins; Arabis setulosa E. L. Greene*					
Arabis perennans S. Wats.	*Arabis arcuata (Nutt. ex Torrey & A. Gray) A. Gray var. perennans (S. Wats.) Jones; Arabis*		n=7 2n=14	***Boechera perennans (S. Watson) W. A. Weber***	*Arabis perennans* S. Watson, Proc. Amer. Acad. Arts 22: 467. 1887; *A. angulata*	

	eremophila E. L. Greene, Arabis recondita E. L. Greene; Arabis angulata E. L. Greene ex Wooton & Standley; Boechera perennans (S. wats.) W. A. Weber				Greene ex Wooton & Standley; *A. arcuata* (Nuttall) A. Gray var. *perennans* (S. Watson) M. E. Jones; *A. eremophila* Greene; *A. recondita* Greene	
	Arabis gracilenta E. L. Greene;			***Boechera gracilenta (Greene) Windham & Al-Shehbaz***	*Arabis gracilenta* Greene, Pittonia 4: 194. 1900; *A. selbyi* Rydb.; *Boechera selbyi* (Rydberg) W.A. Weber	arose through hybridization between *B. fendleri* and *B. pallidifolia*
Arabis selbyi Rydb.	*Boechera selbyi (Rydb.) W. A. Weber*					
Arabis perstellata E. Braun.	*Arabis perstellata E. Braun var. ampla Rollins*		n=7	***Boechera perstellata (E. Braun) Al-Shehbaz***	*Arabis perstellata* E. Braun, Rhodora 42: 47. 1940; *A. perstellata* var. *ampla* Rollins	
Arabis petiolaris (A. Gray) A. Gray	*Streptanthus petiolaris A. Gray; Streptanthus brazoensis Buckley; Erysimum petiolare (A. Gray) Kuntze*		n=14 2n=ca. 28			
Arabis pinzliae Rollins				***Boechera pinzliae (Rollins) Al-Shehbaz***	*Arabis pinzliae* Rollins, Contr. Gray Herb. 212: 110. 1982	at least one genome derived from *B. platysperma*
Arabis platysperma A. Gray var. platysperma	*Erysimum platyspermum (A. Gray) Kuntze; Arabis inamoena E. L. Greene; Arabis oligantha E. L. Greene*	hybridisation with rigidissima Rollins var. demota Rollins		***Boechera platysperma (A. Gray) Al-Shehbaz***	*Arabis platysperma* A. Gray, Proc. Amer. Acad. Arts 6: 519. 1865; *A. chionophila* Greene; *A. inamoena* Greene (1908), not Greene (1911); A. *oligantha* Greene	
Arabis platysperma A. Gray var. howellii (S. Wats. Jepson	*Arabis covillei E. L. Greene; Arabis leibergii E. L. Greene;*		n=7	***Boechera covillei (Greene) Windham & Al-Shehbaz***	*Arabis covillei* Greene, Repert. Sp. Nov. Regni Veg. 5: 243. 1908; *A. leibergii* Greene	arose through hybridization between *B. howellii* and *B. lyallii*
	Arabis howellii S. wats.; Arabis platyloba E. L. Greene; Arabis conferta E. L. Greene; Arabis platyperma A. Gray var. imparata Jepson; Arabis			***Boechera howellii (S. Watson) Windham & Al-Shehbaz***	*Arabis howellii* S. Watson, Proc. Amer. Acad. Arts 25: 124. 1890; *A. conferta* Greene; *A. inamoena* Greene var. *acutatus*	

	inamoena E. L. Greene var. arcuata Jepson				Jepson; *A. platyloba* Greene; *A. platysperma* A. Gray var. *howellii* (S. Watson) Jepson; *A. platysperma* var. *imparata* Jepson	
Arabis puberula Nutt. ex Torrey & A. Gray	*Arabis arida E. L. Greene; Arabis lignipes A. nels var. impar (A. Nels.) Rollins; Arabis sabulosa M. E. Jones; Arabis sabulosa var. frigida M. E. Jones; Arabis sabulosa var. colorata M. E. Jones; Erysimum puberulum (Nutt.) Kuntze*	may be mistaken as holboellii sensu lato		**Boechera puberula (Nuttall) Dorn**	*Arabis puberula* Nuttall in Torrey & A. Gray, Fl. N. Amer. 1: 82. 1838; *A. arida* Greene; *A. beckwithii* S. Watson; *A. lignipes* A. Nelson var. *impar* A. Nelson; *A. sabulosa* M. E. Jones; *A. subpinnatifida* S. Watson var. *beckwithii* (S. Watson) Jepson; *A. subpinnatifida* var. *impar* (A. Nelson) Rollins; *Boechera beckwithii* (S. Watson) Dorn	
Arabis beckwithii S. Wats.	*Arabis subpinnatifida S. Wats. var. beckwithii (S. Wats.) Jepson*	used to be treated as synonym of A. puberula by Rollins				
Arabis pulchra M. E. Jones ex S. Wats. var. duchesnensis Rollins				**Boechera duchesnensis (Rollins) Windham, Al-Shehbaz & Allphin**	*Arabis pulchra* M. E. Jones ex S. Watson var. *duchesnensis* Rollins, Syst. Bot. 6: 59. 1981; *Boechera pulchra* (M. E. Jones ex S. Watson) W. A. Weber var. *duchesnensis* (Rollins) Dorn	arose through hybridization between *B. formosa* and *B. pallidifolia*
Arabis pulchra M. E. Jones ex S. Wats. var. gracilis M. E. Jones	*Arabis trichopoda E. L. Greene; Arabis pulchra var. glabrescens Wiggins; Arabis pulchra var. viridis Jepson*		n=7 2n=14			
Arabis pulchra M. E. Jones ex S. Wats. var. munciensis M. E. Jones				**Boechera lincolnensis Windham & Al-Shehbaz**	*Arabis pulchra* M. E. Jones ex S. Watson var. *munciensis* M. E. Jones; *B. pulchra* (M. E. Jones ex S. Watson) W. A. Weber var. *munciensis*	

					(M. E. Jones) Dorn	
Arabis pulchra M. E. Jones ex S. Wats. var. pallens M. E. Jones	*Arabis formosa E. L. Greene; Boechera pulchra (M. E. Jones es S. Watson) W. A. Weber subsp. pallens*			***Boechera formosa (Greene) Windham & Al-Shehbaz***	*Arabis formosa* Greene, Pittonia 4: 198. 1900; *A. pulchra* M. E. Jones ex S. Watson var. *pallens* M. E. Jones; *Boechera pulchra* (M. E. Jones ex S. Watson) W. A. Weber subsp. *pallens* (M. E. Jones) W. A. Weber; *B. pulchra* var. *pallens* (M. E. Jones) Dorn	
Arabis pulchra M. E. Jones ex S. Wats. var. pulchra	*Boechera pulchra (M. E. Jones ex S. Wats.)*		2n=14	***Boechera pulchra (M. E. Jones ex S. Watson) W. A. Weber***	*Arabis pulchra* M. E. Jones ex S. Watson, Proc. Amer. Acad. Arts 22: 468. 1887	
Arabis pusilla Rollins		closely related to Arabis oxylobula		***Boechera pusilla (Rollins) Dorn***	*Arabis pusilla* Rollins, Contr. Gray Herb. 212: 107. 1982	arose through hybridization between *B. lemmonii* and *B. pendulina*
Arabis pygmaea Rollins				***Boechera pygmaea (Rollins) Al-Shehbaz***	*Arabis pygmaea* Rollins, Rhodora 43: 476. 1941	
				Boechera quebecensis Windham & Al-Shehbaz	*Arabis divaricarpa* A. Nelson var. *dechamplainii* B. Boivin	arose through hybridization between *B. holboellii* and *B. stricta*
Arabis rectissima E. L. Greene var. rectissima	*Arabis setigera E. L. Greene; Arabis wyndii Henderson*			***Boechera rectissima (Greene) Al-Shehbaz***	*Arabis rectissima* Greene, Pittonia 4: 191. 1900; *A. setigera* Greene; *A. wyndii* Henderson	
Arabis rectissima E. L. Greene var. simulans Rollins						
Arabis repanda S. Wats. var. repanda			n=7	***Boechera repanda (S. Watson) Al-Shehbaz***	*Arabis repanda* S. Watson, Proc. Amer. Acad. Arts 11: 122. 1875; *A. inamoena* Greene (1911) not Greene (1908); *A. repanda* var. *greenei* Jepson	
Arabis repanda S. Wats. var. greenei Jepson	*Arabis inamoena E. L. Greene (1911); NOT Arabis inamoena E. L. Greene (1908)*					
Arabis rigidissima Rollins var.				***Boechera rigidissima***	*Arabis rigidissima*	

rigidissima				***(Rollins) Al-Shehbaz***	Rollins, Rhodora 43: 380. 1941; *A. rigidissima* Rollins var. *demota* Rollins	
Arabis rigidissima Rollins var. demota Rollins		closely related to Arabis platysperma				
Arabis rollei Rollins				***Boechera rollei (Rollins) Al-Shehbaz***	*Arabis rollei* Rollins, Harvard Pap. Bot. 4: 43. 1993	
				Boechera rollinsiorum Windham & Al-Shehbaz		
Arabis schistacea Rollibs			2n=14	***Boechera schistacea (Rollins) Dorn***	*Arabis schistacea* Rollins, Contr. Dudley Herb. 3: 370. 1946	
Arabos serotina Steele			2n=14	***Boechera serotina (Steele) Windham & Al-Shehbaz***	*Arabis serotina* Steele, Contr. U.S. Natl. Herb. 13: 365. 1911	
				Boechera serpenticola Windham & Al-Shehbaz		
				Boechera shevockii Windham & Al-Shehbaz		
Arabis shockleyi Munz				***Boechera shockleyi (Munz) Dorn***	*Arabis shockleyi* Munz, Bull. S. Calif. Acad. Sci. 31: 62. 1932	
Arabis shortii (Fern.) Gleason	*Sisymbrium dentatum Torrey; Arabis dentata (Torrey) Torrey & A. Gray; NOT Arabis dentata Allioni; Iodanthus dentatus (Torrey) E. L. Greene; Arabis perstellata E. Braun Var. shortii Fern.; Arabis dentata var. phalacrocarpa M. Hopkins; Arabis shortii var. phalacrocarpa (M. Hopkins) Steyerm.*		2n=12	***Boechera shortii (Fernald) Al-Shehbaz***	*Arabis perstellata* E. Braun var. *shortii* Fernald, Rhodora 48: 208. 1946; *A. dentata* (Torrey) Torrey & A. Gray (1838), not Allioni (1785); *A. dentata* var. *phalacrocarpa* M. Hopkins; *A. shortii* (Fernald) Gleason; *A. shortii* var. *phalacrocarpa* (M. Hopkins) Steyermark; *Iodanthus dentatus* (Torrey) Greene; *Sisymbrium dentatum* Torrey (1833), not Allioni (1785)	
Arabis sparsiflora Nutt. ex Torrey & A. Gray var. arcuata (Nutt. ex Torrey & A. Gray) Rollins	*Streptanthus arcuatus Nutt. ex Torrey & A. Gray; Arabis arcuata*			***Boechera arcuata (Nuttall) Windham & Al-Shehbaz, Harvard Pap.***	*Streptanthus arcuatus* Nuttall in Torrey & A. Gray, Fl. N. Amer. 1:	

	(Nutt.) A. Gray; Arabis holboellii Hornem. var. arcuata; Arabis maxima E. L. Greene;			**Bot. 11: 64. 2006**	77. 1838; *Arabis arcuata* (Nuttall) A. Gray, non Shuttelworth ex Godet; *A. holboellii* Hornemann var. *arcuata* (Nuttall) Jepson; *A. maxima* Greene; *A. sparsiflora* Nuttall var. *arcuata* (Nuttall) Rollins	
	Arabis arcuata var. rubicundula Jepson			***Boechera rubicundula (Jepson) Windham & Al-Shehbaz***	*Arabis arcuata* (Nuttall) A. Gray var. *rubicundula* Jepson, Fl. Calif. 2: 69. 1936	arose through hybridization between *B. breweri* and *B. arcuata*
Arabis sparsiflora Nutt. ex Torrey & A. Gray var. atrorubens (Suksd. ex E. L. Greene)	*Arabis atrorubens Suksd.; Arabis atriflora Suksd.*			**Boechera atrorubens (Suksdorf ex Greene) Windham & Al-Shehbaz**	*Arabis atrorubens* Suksdorf ex Greene, Erythea 1: 223. 1893; *A. atriflora* Suksdorf; *A. sparsiflora* Nuttall var. *atrorubens* (Suksdorf ex Greene) Rollins	
Arabis sparsiflora Nutt. ex Torrey & A. Gray var. californica Rollins			2n=22, 23	***Boechera californica (Rollins) Windham & Al-Shehbaz***	*Arabis sparsiflora* Nutt. var. *californica* Rollins, Rhodora 43: 402. 1941	species arose through hybridization between *B. arcuata* and *B. pulchra*
Arabis sparsiflora Nutt. ex Torrey & A. Gray var. columbiana (Macoun) Rollins	*Arabis columbiana Macoun*		2n=32			
Arabis sparsiflora Nutt. ex Torrey & A. Gray var. sparsiflora	*Arabis peramoena E. l. Greene; Arabis arcoidea A. Nels.; Arabis sparsiflora var. peramoena (E. L. Greene) Rollins*			***Boechera sparsiflora (Nuttall) Dorn***	Arabis sparsiflora Nuttall in Torrey & A. Gray, Fl. N. Amer. 1: 81. 1838; A. campyloloba Greene; A. peramoena Greene; A. polytricha Greene; A. arcoidea A. Nelson, A. sparsiflora var. peramoena (Greene) Rollins	
Arabis sparsiflora Nutt. ex Torrey & A. Gray var. subvillosa (S. Wats.) Rollins	*Arabis poyltricha E. L. Greene; Arabis campylolobula E. L. Greene;*					
	Arabis arcuata (Nutt ex Torrey & A. Gray) A. Gray var. subvillosa S. Wats.; Arabis elegans A. Nels.; Arabis perelegans	hybridizes with Arabis holboellii var. retrofracta in southern Idaho; hybridizes with A. breweri in California	2n=21, 22 +1B	***Boechera pauciflora (Nuttall) Windham & Al-Shehbaz***	*Sisymbrium pauciflorum* Nuttall in Torrey & A. Gray, Fl. N. Amer. 1: 93. 1838; *Arabis arcuata* (Nuttall) A.	arose through hybridization between *B. sparsiflora* and *B. retrofracta*

	A. Nels.; Arabis subserata E. L. Greene				Gray var. *subvillosa* S. Watson; *A. elegans* A. Nelson (1900), not Tineo ex Lojacono (1886); *A. perelegans* A. Nelson; *A. sparsiflora* Nuttall var. *subvillosa* (S. Watson) Rollins; *Boechera sparsiflora* (Nuttall) Dorn var. *subvillosa* (S. Watson) Dorn	
Arabis subpinnatifida S. Wats.			n=7 2n=14	***Boechera subpinnatifida (S. Watson) Al-Shehbaz***	*Arabis subpinnatifida* S. Watson, Proc. Amer. Acad. Arts 20: 353. 1885	
Arabis suffrutescens S. Wats. var. suffrutescens	*Arabis duriuscula E. L. Greene; Arabis dianthifolia E. L. Greene;*			***Boechera suffrutescens (S. Watson) Dorn***	*Arabis suffrutescens* S. Watson, Proc. Amer. Acad. Arts 17: 362. 1882; *A. dianthifolia* Greene; *A. duriscula* Greene	
Arabis suffrutescens S. Wats. var. horizontalis (E. L. Greene) Rollins	*Arabis horizontalis E. L. Greene*			***Boechera horizontalis (Greene) Windham & Al-Shehbaz***	*Arabis horizontalis* Greene, Leafl. Bot. Observ. Crit. 2: 74. 1910; *A. suffrutescens* S. Watson var. *horizontalis* (Greene) Rollins	arose through hybridization between *B. suffrutescens* and *B. lemmonii*
Arabis tiehmii Rollins		most closely related to Arabis davidsonii var. davidsonii		***Boechera tiehmii (Rollins) Al-Shehbaz***	*Arabis tiehmii* Rollins, J. Arnold Arbor. 64: 496. 1983	
Arabis williamsii Rollins var. saximontana (Rollins) Rollins	*Arabis pendulocarpa A. Nels. var. saximontana (Rollins) Dorn*	based on Arabis microphylla Nutt. ex Torrey & A. Gray var. saximontana Rollins	2n=14	***Boechera saximontana (Rollins) Windham & Al-Shehbaz***	*Arabis microphylla* Nuttall var. *saximontana* Rollins, Rhodora 43: 429. 1941; *A. pendulocarpa* A. Nelson var. *saximontana* (Rollins) Dorn; *A. williamsii* Rollins var. *saximontana* (Rollins) Rollins; *Boechera*	microphylla and williamsii may be involved in hybridization

					williamsii (Rollins) Dorn var. *saximontana* (Rollins) Dorn	
				***Boechera tularensis* Windham & Al-Shehbaz**		contains three different genomes, one each from *B. stricta, B. rectissima*, and *B. retrofracta*
				***Boechera ultraalsa* Windham & Al-Shehbaz**		originally identified as platysperma
				***Boechera villosa* Windham & Al-Shehbaz**		originally determined as perennans
Arabis williamsii Rollins var. williamsii		hybrids with Arabis holboellii var. pendulocarpa		***Boechera williamsii* (Rollins) Dorn**	*Arabis williamsii* Rollins, Syst. Bot. 6: 62. 1981	
