# Supplementary material for: A Continental-Wide Perspective: The Genepool of Nuclear Encoded Ribosomal DNA and Single-Copy Gene Sequences in North American Boechera (Brassicaceae)
Source: PLoS One. 2012 May 14;7(5):e36491. doi: 10.1371/journal.pone.0036491 (PMC3351400; doi:10.1371/journal.pone.0036491)
Supplement: Table S2 — Details of the accessions used in this study; given are the taxon name according to the taxonomy of [14] if possible, geographical information, collector, source herbarium and herbarium accession number as given on the herbarium voucher as well as ITS type, cpDNA type and single-copy gene type with their respective Genebank accession numbers; furthermore it is indicated in which ecoregion [72] the accessions were placed (individuals included in statistical analysis). (PDF) [file pone.0036491.s002.pdf]

Accession	Taxon	wwf_ecoregion	ITS-type	ITS accession number	trnL/F type	trnLF accession numbers	*At2g25920 orthologue*	*At3g18900 orthologue*
Arab0002	*Boechera stricta*	Wasatch/Uinta Montane Forest	b	AY165313	AS	AY257736		
Arab0003	*Boechera stricta*	Colorado Plateau Shrub Steppe	c	AY165314	AS	AY257736		
Arab0005	*Boechera stricta*	South Central Rockies Forest West	c	AY165314	AH	AY257725		
Arab0007	*Boechera stricta*	Colorado Rockies Forest	d	AY165315	AI			
Arab0008	*Boechera laevigata*	Southern Great Lakes Forest	e	AY165316	AS	AY257736		
Arab0013	*Boechera pinetorum*	Great Basin Shrub Steppe central East	f	AY165346	C	AY257694		
Arab0016	*Boechera pinetorum*	Sierra Nevada North	i	AY165317	CQ	AY257788		
Arab0021	*Boechera "divaricarpa"*	Great Basin Shrub Steppe West	k	AY165318	AH	AY257725		
Arab0023	*Boechera stricta*	Great Basin Shrub Steppe West	l	AY165319	AH	AY257725		
Arab0025	*Boechera stricta*	Colorado Rockies Forest	m	AY165320	AI	AY257726		
Arab0027	*Boechera stricta*	Snake/Columbia Shrub Steppe	c	AY165314	AS	AY257736		
Arab0029	*Boechera laevigata*	Allegheny Highlands	e	AY165316	AS	AY257736		
Arab0033	*Boechera "divaricarpa"*	South Central Rockies Forest East	n	AY165348	B	AY257693		
Arab0035	*Boechera "divaricarpa"*	Western Great Lakes Forest	e	AY165316	AS	AY257736		
Arab0036	*Boechera "divaricarpa"*	Eastern Great Lakes Forest	o, p, q	AY165350, AY165351, AY165352	AS	AY257736		
Arab0037	*Boechera "divaricarpa"*	Eastern Great Lakes Forest	e	AY165316	AS	AY257736		
Arab0040	*Boechera "divaricarpa"*	Great Basin Shrub Steppe East	r	AY165321	B	AY257693		
Arab0041	*Boechera "divaricarpa"*	North Central Rockies Forest	s	AY165322				
Arab0042	*Boechera "divaricarpa"*	South Central Rockies Forest West	t	AY165323	AH	AY257725		
Arab0043	*Boechera "divaricarpa"*	Colorado Rockies Forest	u	AY165324	AH	AY257725		
Arab0045	*Boechera "divaricarpa" x B. holboellii var. retrofracta*	Great Basin Shrub Steppe East	h	AY165348	X	AY257715		
Arab0046	*Boechera pinetorum*	Colorado Rockies Forest	x, w	AY165353, AY165347	AT	AY257737		
Arab0047	*Boechera pinetorum*	California Montane Chaparral and Woodlands	z	AY165325	CH	AY257777		
Arab0049	*Boechera "holboellii"*	Great Basin Shrub Steppe central West	h	AY165348				
Arab0054	*Boechera pendulocarpa*	Wasatch/Uinta Montane Forest	ab	AY165326				
Arab0057	*Boechera pendulocarpa*	Wasatch/Uinta Montane Forest	ab	AY165326				

Accession	Country	State/ Province	County	Herbarium	Herbarium number	Collector	Coll. number	X coordi-nate	Y coordi-nate
Arab0002	USA	Utah	Carbon	MO	3472784	R.C. & K.W. Rollins	83119	-110.80	39.80
Arab0003	USA	Utah	Kane						
Arab0005	USA	Idaho	Custer	MO	3446183	R.C. & K.W. Rollins	83268	-114.33	44.51
Arab0007	USA	Colorado	Rio Grande						
Arab0008	USA	Ohio	Ottawa	MO	5017293	A.W. Cusick	32435	-82.80	41.68
Arab0013	USA	Nevada	White Pine	MO	3464767	R.C. & K.W. Rollins	83190	-114.25	39.03
Arab0016	USA	California	Plumas	MO	3391157	R.C. & K.W. Rollins	81159	-120.41	39.86
Arab0021	USA	California	Mono						
Arab0023	USA	Nevada	Nye	MO	3397059	R.C. & K.W. Rollins	79235	-117.35	38.96
Arab0025	USA	Colorado	San Juan	MO	3660437	G. Rhinehalt	36	-107.71	37.85
Arab0027	USA	Idaho	Custer	MO	1212063	A. Cronquist	3138	-113.85	44.16
Arab0029	USA	New York	Tompkins	MO	978554	F.P. Metcalf		-76.50	42.45
Arab0033	USA	Wyoming	Sublette	MO	927159	E.B. Payson, L.B. Payson			
Arab0035	USA	Michigan	Keweenaw	MO	1149887	N.C. Fassett	20102	-87.96	47.45
Arab0036	Canada	Ontario	Northumberland	MO	1642190	J.H. Soper, J.K. Shields	4638		
Arab0037	Canada	Ontario	Bruce	MO	1244554	N.C. Fassett	21020	-81.18	44.96
Arab0040	USA	Idaho	Bannock	MO	3389509	L.C. Higgins	1629	-112.35	42.69
Arab0041	USA	Montana	Glacier	MO	3390713	L.C. Higgins	1677		
Arab0042	USA	Montana	Ravalli	MO	3464766	R.C. & K.W. Rollins	83297	-113.93	45.70
Arab0043	USA	Colorado	Park	MO	3391087	R.C. Rollins	51290	-106.05	39.33
Arab0045	USA	Utah	Box Elder	MO	3391159	R.C. & K.W. Rollins	81257		
Arab0046	USA	Colorado	Boulder	MO	1132474	F. Ramaley	15986		
Arab0047	USA	California	San Bernardino	MO	142280	S.B. Parish	3011		
Arab0049	USA	Nevada	Humboldt						
Arab0054	USA	Utah	Rich	MO	3391146	R.C. & K.W. Rollins	81333	-111.46	41.91
Arab0057	USA	Utah	Rich	MO	3398768	R.C. & K.W. Rollins	79310	-111.46	41.93

Accession	Taxon	wwf_ecoregion	ITS-type	ITS accession number	trnL/F type	trnLF accession numbers	*At2g25920 orthologue*	*At3g18900 orthologue*
Arab0061	*Boechera collinsii*	Western Canadian Forests	h	AY165348	BY	AY257768		
Arab0064	*Boechera retrofracta*	South Central Rockies Forest East	h	AY165348				
Arab0065	*Boechera retrofracta*	Wyoming Basin Shrub Steppe	ac	AY165327	U	AY257712		
Arab0069	*Boechera retrofracta*	Okanogan Forest	h	AY165348	CC	AY257772		
Arab0070	*Boechera retrofracta*	Great Basin Shrub Steppe East	h	AY165348	U	AY257712		
Arab0071	*Boechera retrofracta*	South Central Rockies Forest West	h	AY165348	CN	AY257783		
Arab0072	*Boechera retrofracta*	Klamath Siskiyou Forests	i	AY165317	CB	AY257771		
Arab0073	*Boechera retrofracta*	South Central Rockies Forest West	h	AY165348	CI	AY257778		
Arab0074	*Boechera retrofracta*	Montana Valley Foothill Grasslands	h	AY165348	CI	AY257778		
Arab0077	*Boechera retrofracta*	Sierra Nevada South	h	AY165348	CM	AY257782		
Arab0079	*Boechera retrofracta*	Sierra Nevada South	ad	AY165328	CI	AY257778		
Arab0082	*Boechera collinsii*	Montana Valley Foothill Grasslands	h	AY165348	CI	AY257778		
Arab0083	*Boechera collinsii*	NW Mixed Grasslands	h	AY165348	CG	AY257776		
Arab0088b	*Boechera stricta*	South Central Rockies Forest East	l	AY165319	AS	AY257736		
Arab0089	*Boechera collinsii*	South Central Rockies Forest East	r	AY165321	BJ	AY257753		
Arab0096	*Boechera pinetorum*	Great Basin Shrub Steppe central West	ad	AY165328	U	AY257712		
Arab0097	*Boechera pinetorum*	Mojave Desert	ak, ag, ai, ah	AY165358, AY165362, AY165359, AY165360	U	AY257712		
Arab0100	*Boechera pinetorum*	Great Basin Shrub Steppe central East	w	AY165347	U	AY257712		
Arab0105	*Boechera pinetorum*	Great Basin Shrub Steppe central East	hi, an, ap, am, ag	AY165367, AY165364, AY165366, AY165363, AY165358	C	AY257694		
Arab0106	*Boechera "holboellii"*	Great Basin Shrub Steppe East	f	AY165346				

Accession	Country	State/ Province	County	Herbarium	Herbarium number	Collector	Coll. number	X coordi-nate	Y coordi-nate
Arab0061	Canada	Saskatchewan		MO	2819807	W.K.W. Baldwin	10992	-104.63	54.05
Arab0064	USA	Wyoming	Lincoln	MO	914442	E.B. Payson, G.M. Armstrong		-110.83	42.71
Arab0065	USA	Wyoming	Sweetwater	MO	1791237	R.C. Rollins 5139, C.L. Porter		-110.03	41.08
Arab0069	USA	Washington	Stevens	MO	1198589	L. Constance	1841	-118.15	48.36
Arab0070	USA	Utah	Cache	MO	1067373	W.C. Muenscher, B. Maguire		-111.80	41.70
Arab0071	USA	Idaho	Valley	MO	1303871	C.L. Hitchcock & C.V. Mublick	14046	-115.66	44.65
Arab0072	USA	California	Siskiyou	MO	1289310	L. Constance, R.C. Reeds		-123.11	41.66
Arab0073	USA	Idaho	Blaine	MO	1212105	A. Cronquist	2612	-114.86	43.91
Arab0074	USA	Montana	Beaverhead						
Arab0077	USA	California	Tulare	MO	2610650	E.C. Twisselmann, D.J. Bedell	18744	-118.18	36.20
Arab0079	USA	California	Inyo	MO	2575166	A.R. & H.N. Moldenke	25918	-118.74	37.45
Arab0082	USA	Montana	Missoula	GH		C.L. Hitchcock	1686	-113.85	46.88
Arab0083	USA	Montana		GH		E.L. Larsen		-104.05	48.86
Arab0088b	USA	Wyoming	Fremont						
Arab0089	USA	Wyoming	Fremont						
Arab0096	USA	Nevada	White Pine						
Arab0097	USA	Nevada	Clark	GH		I.W. Clokey, R.C. Anderson			
Arab0100	USA	Nevada	Lincoln	GH		P. Train	2676		
Arab0105	USA	Nevada	White Pine	GH		R.C. & K.W. Rollins	83190		
Arab0106	USA	Nevada	Lincoln	GH		R.C. & K.W. Rollins	81117	-114.10	37.73

Accession	Taxon	wwf_ecoregion	ITS-type	ITS accession number	trnL/F type	trnLF accession numbers	*At2g25920 orthologue*	*At3g18900 orthologue*
Arab0107	*Boechera pinetorum*	Great Basin Shrub Steppe central West	as	AY165368	CI	AY257778		
Arab0108	*Boechera pinetorum*	Great Basin Shrub Steppe West	ac	AY165327	N	AY257705		
Arab0109	*Boechera pinetorum*	Great Basin Shrub Steppe West	aw, an, ax, hh, ag	AY165371, AY165364, AY165372, AY165373, AY165358	S	AY257710		
Arab0110	*Boechera pinetorum*	Great Basin Shrub Steppe West	au	AY165370	N	AY257705		
Arab0113	*Boechera "divaricarpa"*	New England/Acadian Forest	ay	GQ166419	AS	AY257736		
Arab0115	*Boechera "divaricarpa"*	Muskwa/Slave Lakes Forest	n	AY165319	AS	AY257736		
Arab0116	*Boechera "divaricarpa"*	Northern Mixed Grasslands	l	AY165319	CG	AY257776		
Arab0118	*Boechera "divaricarpa"*	Western Canadian Forests	h	AY165348	AH	AY257725		
Arab0119	*Boechera "divaricarpa"*	Canadian Aspen Forest and Parklands	be, bd, bc	AY165377, AY165376, AY165375	AZ	AY257743		
Arab0120	*Boechera "divaricarpa"*	Western Canadian Forests	bf, bg	AY165378, AY165379	AS	AY257736		
Arab0121	*Boechera "divaricarpa"*	Montana Valley Foothill Grasslands	s	AY165322	AH	AY257725		
Arab0122	*Boechera "divaricarpa"*	North Central Rockies Forest	bh, bi	AY165380, AY165381	BR	AY257761		
Arab0123	*Boechera "divaricarpa"*	North Central Rockies Forest	bj	AY165329	B	AY257693		
Arab0124	*Boechera "divaricarpa"*	Canadian Aspen Forest and Parklands	bl	AY165374	AS	AY257736		
Arab0124			bk	AY165382				
Arab0125	*Boechera "divaricarpa"*	North Central Rockies Forest	h	AY165348	AH	AY257725		
Arab0126	*Boechera "divaricarpa"*	New England/Acadian Forest	e	AY165316	AS	AY257736		
Arab0128	*Boechera "divaricarpa"*	Eastern Great Lakes Forest	e	AY165316	AS	AY257736		
Arab0129	*Boechera "divaricarpa"*	Eastern Forest/Boreal Transition	e	AY165316	AS	AY257736		
Arab0130	*Boechera "divaricarpa"*	Central Canadian Shield Forest	e	AY165316	AS	AY257736		
Arab0131	*Boechera "divaricarpa"*	Eastern Great Lakes Forest	e	AY165316	AS	AY257736		
Arab0132	*Boechera "divaricarpa"*	Eastern Forest/Boreal Transition	h	AY165348	AS	AY257736		

Accession	Country	State/ Province	County	Herbarium	Herbarium number	Collector	Coll. number	X coordi-nate	Y coordi-nate
Arab0107	USA	Nevada	Humboldt						
Arab0108	USA	Nevada	Mineral	GH		A. Pinzl	9951	-118.21	38.61
Arab0109	USA	Nevada	Mineral	GH		A. Pinzl	9957		
Arab0110	USA	Nevada	Mineral						
Arab0113	Canada	Quebec		GH		J. Rousseau		-68.23	48.61
Arab0115	Canada	Northwest Territories	Mackenzie	GH		W.J. Cody, J.M. Matte	8388		
Arab0116	Canada	Saskatchewan	Regina	GH		B. Boivin, W.G. Dore	7523	-104.61	50.43
Arab0118	Canada	Saskatchewan		GH		H.M. Raup		-109.21	59.60
Arab0119	Canada	Saskatchewan		GH		W. Krivda			
Arab0120	Canada	Saskatchewan							
Arab0121	Canada	Alberta		GH		E.H. Moss	1138	-113.28	49.18
Arab0122	Canada	Alberta		GH		A.J. Breitung	16749		
Arab0123	Canada	Alberta		GH		A.J. Breitung	16141	-114.05	49.07
Arab0124	Canada	Alberta		GH		W.J. Cody, R.L. Gutteridge	6938		
Arab0124									
Arab0125	Canada	Alberta		GH		F.J. Hermann	12647		
Arab0126	Canada	Quebec	Bonaventure	GH		E.F. Williams, M.L. Fernald		-65.33	48.01
Arab0128	Canada	Quebec	Argenteuil	GH		F. Marie-Victorin et al.			
Arab0129	Canada	Quebec		GH		F. Marie-Victorin			
Arab0130	Canada	Ontario	Thunder Bay	GH		A.S. Pease, R.C. Bean			
Arab0131	Canada	Ontario		GH		A.S. Pease, E.C. Ogden		-82.45	45.91
Arab0132	Canada	Quebec		GH		F. Marie-Victorin et al.		-70.93	46.93

Accession	Taxon	wwf_ecoregion	ITS-type	ITS accession number	trnL/F type	trnLF accession numbers	*At2g25920 orthologue*	*At3g18900 orthologue*
Arab0135	*Boechera "divaricarpa"*	New England/Acadian Forest	e	AY165316	AS	AY257736		
Arab0136	*Boechera "divaricarpa"*	Eastern Great Lakes Forest	e	AY165316	AS	AY257736		
Arab0137	*Boechera "divaricarpa"*	Eastern Canadian Forests	e	AY165316	AS	AY257736		
Arab0139	*Boechera "divaricarpa"*	Eastern Great Lakes Forest	bn	AY165330	AS	AY257736		
Arab0140	*Boechera "divaricarpa"*	Western Great Lakes Forest	e	AY165316	AS	AY257736		
Arab0141	*Boechera "divaricarpa"*	Southern Hudson Bay Taiga	e	AY165316	AS	AY257736		
Arab0142	*Boechera "divaricarpa"*	Midwestern Canadian Shield Forest	bo, bp, br	AY165384, AY165385, AY165386	AS	AY257736		
Arab0143	*Boechera "divaricarpa"*	Northern Tall Grassland	e	AY165316	AS	AY257736		
Arab0144	*Boechera "divaricarpa"*	Eastern Canadian Forests	e	AY165316	AS	AY257736		
Arab0146	*Boechera stricta*	Colorado Plateau Shrub Steppe	l	AY165319	AH	AY257725		
Arab0152	*Boechera stricta*	Wasatch/Uinta Montane Forest	c	AY165314	BD	AY257747		
Arab0156	*Boechera stricta*	New England/Acadian Forest	e	AY165316	AS	AY257736		
Arab0158	*Boechera stricta*	North Cascade Forests	az	AY165374	AE	AY257722		
Arab0160	*Boechera stricta*	North Cascade Forests	az	AY165374	AH	AY257725		
Arab0161	*Boechera stricta*	Central and Southern Cascades Forest	az	AY165374	AH	AY257725		
Arab0162	*Boechera stricta*	Central and Southern Cascades Forest	t	AY165323	AH	AY257725		
Arab0163	*Boechera pendulocarpa*	Cascade Mountains Leeward Forests	h	AY165348	M	AY257704		
Arab0167	*Boechera retrofracta*	South Central Rockies Forest West	z	AY165325		AY257699		
Arab0168	*Boechera pendulocarpa*	South Central Rockies Forest West	bt	AY165331	M	AY257704		
Arab0169	*Boechera retrofracta*	South Central Rockies Forest West	h	AY165348	CV	AY257791		
Arab0173	*Boechera pendulocarpa*	Wasatch/Uinta Montane Forest North	bv	AY165332	CR	AY257789		
Arab0174	*Boechera pendulocarpa*	South Central Rockies Forest West	bt	AY165331	B	AY257693		
Arab0176	*Boechera pendulocarpa*	South Central Rockies Forest West	bw	AY165333	BN	AY257757		

Accession	Country	State/ Province	County	Herbarium	Herbarium number	Collector	Coll. number	X coordi-nate	Y coordi-nate
Arab0135	Canada	Quebec	Bonaventure	GH		J.F. Collins, M.L. Fernald			
Arab0136	Canada	Quebec		GH		L. Cinq-Mars, C. Rousseau		-71.28	46.75
Arab0137	Canada	Quebec	Matane	GH		M.L. Fernald et al.		-66.70	49.08
Arab0139	Canada	Quebec	Argenteuil	GH		F. Marie-Victorin, F. Rolland-Germain		-74.65	45.65
Arab0140	Canada	Manitoba	Springfield	GH		B. Boivin, E. Laishley			
Arab0141	Canada	Manitoba	Churchill	GH		W.B. Schofield			
Arab0142	Canada	Manitoba		GH		H.J. Scoggan	5300		
Arab0143	Canada	Manitoba		GH		H.J. Scoggan	8496	-97.18	50.08
Arab0144	Canada	Quebec	Matane	GH		M.L. Fernald, A.S. Pease		-69.76	48.23
Arab0146	USA	Utah	San Juan	GH		B. Maguire, J.D. Reed	1854	-109.83	37.66
Arab0152	USA	Utah	Sevier	GH		R.C. Rollins	57306	-111.45	38.75
Arab0156	USA	Vermont	Rutland	GH		W.W. Eggleston	1025	-72.88	43.81
Arab0158	USA	Washington	Whatcom	GH		W.C. Muenscher	7883	-121.88	48.78
Arab0160	USA	Washington	Whatcom	GH		W.C. Muenscher	7875	-121.13	48.75
Arab0161	USA	Washington		GH		G.N. Jones	10002	-121.66	46.88
Arab0162	USA	Washington		GH		L.J. Henderson		-121.50	46.25
Arab0163	Canada	British Columbia		GH		J.W. & E.M. Thompson		-121.55	50.38
Arab0167	USA	Idaho	Custer	GH		C.L. Hitchcock, C.V. Muhlick	9574	-114.85	44.50
Arab0168	USA	Idaho	Custer	GH		C.L. Hitchcock, C.V. Muhlick	9572	-114.85	44.50
Arab0169	USA	Idaho	Lemhi	GH		C.L. Hitchcock, C.V. Muhlick	9422	-114.28	44.85
Arab0173	USA	Idaho	Bear Lake	GH		R.C. & K.W. Rollins	8684	-111.58	42.23
Arab0174	USA	Idaho	Custer	GH		R.C. & K.W. Rollins	86163	-113.83	44.40
Arab0176	USA	Idaho	Blaine	GH		R.C. & K.W. Rollins	86120	-114.71	43.86

Accession	Taxon	wwf_ecoregion	ITS-type	ITS accession number	trnL/F type	trnLF accession numbers	***At2g25920 orthologue***	***At3g18900 orthologue***
Arab0179	*Boechera pendulocarpa*	Montana Valley Foothill Grasslands	bx	AY165334	M	AY257704		
Arab0180	*Boechera pendulocarpa*	Montana Valley Foothill Grasslands	h	AY165348	BU	AY257764		
Arab0181	*Boechera pendulocarpa*	Montana Valley Foothill Grasslands	bt	AY165331	BW	AY257766		
Arab0182	*Boechera pendulocarpa*	Montana Valley Foothill Grasslands	bx	AY165334	BV	AY257765		
Arab0183	*Boechera "divaricarpa"*	South Central Rockies Forest East	be	AY165377				
Arab0185	*Boechera "divaricarpa"*	South Central Rockies Forest East	l	AY165319	AS	AY257736		
Arab0186	*Boechera "divaricarpa"*	Colorado Rockies Forest	bw	AY165333	AS	AY257736		
Arab0187	*Boechera "divaricarpa"*	Western Great Lakes Forest	e	AY165316	AS	AY257736		
Arab0188	*Boechera "divaricarpa"*	Cascade Mountains Leeward Forests	az	AY165374	AH	AY257725		
Arab0189	*Boechera "divaricarpa"*	Klamath Siskiyou Forests	ad	AY165328	CI	AY257778		
Arab0190	*Boechera "divaricarpa"*	Klamath Siskiyou Forests	i	AY165317	CI	AY257778		
Arab0192	*Boechera "divaricarpa"*	Central and Southern Cascades Forest	ca	AY165323	CI	AY257778		
Arab0193	*Boechera "divaricarpa"*	Central and Southern Cascades Forest	ad	AY165328	CI	AY257778		
Arab0195	*Boechera cf. divaricarpa x B. holboellii var. retrofracta*	Great Basin Shrub Steppe East	h	AY165348	Y	AY257716		
Arab0196	*Boechera cf. divaricarpa x B. holboellii var. retrofracta*	Great Basin Shrub Steppe central East	cb	AY457928	CI	AY257778		
Arab0202	*Boechera pinetorum*	Colorado Rockies Forest	y	AY165355	AH	AY257725		
Arab0210	*Boechera pinetorum*	Eastern Cascades Forest	ar	AY165369	CI	AY257778		
Arab0213	*Boechera pinetorum*	Sierra Nevada North	i	AY165317				
Arab0218	*Boechera pinetorum*	Great Basin Shrub Steppe central West	ac	AY165327	N	AY257705		
Arab0219	*Boechera pinetorum*	Sierra Nevada North	ar	AY165369	CQ	AY257788		
Arab0221	*Boechera retrofracta*	Alberta/British Columbia Forest	h	AY165348	CS	AY257790		
Arab0222	*Boechera retrofracta*	Great Basin Shrub Steppe central West	h	AY165348	U	AY257712		
Arab0225	*Boechera retrofracta*	Great Basin Shrub Steppe West	au	AY165370	CJ	AY257779		
Arab0227	*Boechera retrofracta*	Great Basin Shrub Steppe central East	ac	AY165327	U	AY257712		
Arab0228	*Boechera retrofracta*	Great Basin Shrub Steppe central West	f	AY165346	O	AY257706		

Accession	Country	State/ Province	County	Herbarium	Herbarium number	Collector	Coll. number	X coordi- nate	Y coordi- nate
Arab0179	USA	Montana	Sanders						
Arab0180	USA	Montana	Missoula						
Arab0181	USA	Montana	Missoula	GH		J. Antonich	5	-113.98	46.87
Arab0182	USA	Montana	Sanders						
Arab0183	USA	Wyoming	Fremont						
Arab0185	USA	Wyoming	Park						
Arab0186	USA	Wyoming	Albany	GH		G.J. Goodman		-106.30	41.30
Arab0187	Canada	Manitoba	Provencher	GH		B. Boivin			
Arab0188	Canada	British Columbia		GH		J.A. Calder, D.B.O. Savile		-120.63	49.12
Arab0189	USA	California	Humboldt	GH		J.P. Tracy	10396	-123.41	41.03
Arab0190	USA	California	Siskiyou	GH		J.Th. Howell		-123.23	41.61
Arab0192	USA	Oregon	Klamath	GH		W.H. Baker	7197		
Arab0193	USA	Oregon	Klamath	GH		W.H. Baker	7289	-122.06	42.91
Arab0195	USA	Utah	Box Elder	GH		R.C. & K.W. Rollins	81257	-113.78	41.86
Arab0196	USA	Nevada	Elko	GH		R.C. Rollins	2546a, T.S. Chambers	-115.38	40.60
Arab0202	USA	Colorado	Costilla	GH		R.C. Rollins	12881	-105.23	37.60
Arab0210	USA	California	Modoc						
Arab0213	USA	California	Plumas	GH		J.Th. Howell, G.H. True		-120.85	39.86
Arab0218	USA	California	Inyo	GH		M. DeDecker	1905	-118.11	37.01
Arab0219	USA	California	El Dorado	GH		L.S. Rose	41301	-120.09	38.99
Arab0221	USA	Nevada	Elko	GH		R.C. Rollins	2579, T.S. Chamberas	-115.40	51.86
Arab0222	USA	Nevada	Nye						
Arab0225	USA	Nevada	Lyon	GH		I. Tidestrom	10204	-119.16	38.48
Arab0227	USA	Nevada	Elko	GH		P. Train	3659	-114.66	41.20
Arab0228	USA	Nevada	Nye						

Accession	Taxon	wwf_ecoregion	ITS-type	ITS accession number	trnL/F type	trnLF accession numbers	*At2g25920 orthologue*	*At3g18900 orthologue*
Arab0229	*Boechera retrofracta*	Great Basin Shrub Steppe central West	aa	AY165357	S	AY257710		
Arab0230	*Boechera retrofracta*	Great Basin Shrub Steppe central East	h	AY165348	U	AY257712		
Arab0237	*Boechera "holboellii"*	Central and Southern Cascades Forest	az	AY165374	CC	AY257772		
Arab0238	*Boechera stricta*	Colorado Rockies Forest	cm	AY165335	AI	AY257726		
Arab0239	*Boechera stricta*	Colorado Rockies Forest	l	AY165319	AS	AY257736		
Arab0240	*Boechera stricta*	Colorado Rockies Forest	l	AY165319	AP	AY257733		
Arab0241	*Boechera stricta*	Colorado Rockies Forest	d	AY165315	BB	AY257745		
Arab0242	*Boechera stricta*	Colorado Rockies Forest	v	AY165353	BF	AY257749		
Arab0243	*Boechera stricta*	Colorado Rockies Forest	l	AY165319	AS	AY257736		
Arab0244	*Boechera stricta*	Colorado Rockies Forest	l	AY165319	AS	AY257736		
Arab0245	*Boechera stricta*	Colorado Rockies Forest	v	AY165353	AS	AY257736		
Arab0246	*Boechera stricta*	Colorado Rockies Forest	v	AY165353	AS	AY257736		
Arab0248	*Boechera stricta*	Great Basin Shrub Steppe East	c	AY165314	AS	AY257736		
Arab0251	*Boechera stricta*	South Central Rockies Forest West	t	AY165323	AD	AY257721		
Arab0254_1	*Boechera stricta*	South Central Rockies Forest East	r	AY165321	AO	AY257732		
Arab0254_2	*Boechera stricta*	South Central Rockies Forest East	l	AY165319				
Arab0260	*Boechera stricta*	Colorado Rockies Forest	l	AY165319	BB	AY257745		
Arab0263	*Boechera stricta*	North Central Rockies Forest	az	AY165374	AH	AY257725		
Arab0266	*Boechera stricta*	Montana Valley Foothill Grasslands	t	AY165323	AH	AY257725		
Arab0272	*Boechera stricta*	Western Great Lakes Forest	e	AY165316	AS	AY257736		
Arab0275	*Boechera stricta*	South Central Rockies Forest East	az	AY165374	AH	AY257725		
Arab0277	*Boechera stricta*	Colorado Rockies Forest	l	AY165319	AK	AY257728		
Arab0284	*Boechera stricta*	South Central Rockies Forest East	r	AY165321	AS	AY257736		
Arab0287	*Boechera stricta*	South Central Rockies Forest East	v	AY165353	BJ	AY257753		
Arab0289	*Boechera stricta*	South Central Rockies Forest East	l	AY165319	BH			
Arab0292	*Boechera stricta*	Snake/Columbia Shrub Steppe	r	AY165321	AS	AY257736		
Arab0293	*Boechera stricta*	South Central Rockies Forest West	t	AY165323	AH	AY257725		

Accession	Country	State/ Province	County	Herbarium	Herbarium number	Collector	Coll. number	X coordi-nate	Y coordi-nate
Arab0229	USA	Nevada	Nye						
Arab0230	USA	Nevada	Elko						
Arab0237	USA	Washington		GH		G.N. Jones	9988	-121.66	46.88
Arab0238	USA	Colorado	Ouray	GH		U.T. Waterfall	11686	-107.68	37.91
Arab0239	USA	Colorado	Gunnison	GH		L.C. Higgins	1939	-106.98	38.95
Arab0240	USA	Colorado	Gunnison	GH		R.C. Rollins	1459	-106.48	38.68
Arab0241	USA	Colorado	San Juan	GH		A.R. Hodgdon, G.B. Rossbach		-107.70	37.88
Arab0242	USA	Colorado	Grand	GH		R.C. Rollins	1039	-106.08	40.25
Arab0243	USA	Colorado	Grand	GH		R.C. Rollins	1038	-105.84	40.31
Arab0244	USA	Colorado	Lake	GH		R.C. Rollins	1350	-106.56	39.10
Arab0245	USA	Colorado	Grand	GH		R.C. Rollins	1028	-106.08	40.25
Arab0246	USA	Colorado	Custer	GH		R.C. Rollins	1218	-105.06	37.98
Arab0248	USA	Idaho	Power	GH		R.C. & K.W. Rollins	81308	-112.97	42.38
Arab0251	USA	Idaho	Custer	GH		A. Cronquist	2814	-115.18	44.40
Arab0254_1	USA	Wyoming	Sublette	GH		R.C. & K.W. Rollins		-109.65	42.86
Arab0254_2	USA	Wyoming	Sublette	GH		R.C. Rollins	79302, K.W. Rollins	-109.65	42.86
Arab0260	USA	Wyoming	Albany	GH		R.C. Rollins	991a	-105.36	41.15
Arab0263	USA	Montana	Glacier	GH		R.C. Rollins	57214	-113.71	48.80
Arab0266	USA	Montana	Lake	GH		C.L. Hitchcock	18266	-113.98	47.41
Arab0272	USA	Michigan	Iosco	GH		M.L. Fernald, A.S. Pease	3330	-83.33	44.43
Arab0275	USA	Wyoming	Park						
Arab0277	USA	Wyoming	Carbon	GH		M. Ownbey	1077	-107.01	41.18
Arab0284	USA	Wyoming	Sublette	GH		C.L. Porter		-110.56	42.60
Arab0287	USA	Wyoming	Big Horn						
Arab0289	USA	Wyoming	Washakie						
Arab0292	USA	Idaho	Clark	GH		R.J. Davis	338	-112.30	44.26
Arab0293	USA	Idaho	Idaho	GH		R.C. & K.W. Rollins	83234	-115.70	45.26

Accession	Taxon	wwf_ecoregion	ITS-type	ITS accession number	trnL/F type	trnLF accession numbers	*At2g25920 orthologue*	*At3g18900 orthologue*
Arab0294	*Boechera stricta*	South Central Rockies Forest West	c	AY165314	AH	AY257725		
Arab0295	*Boechera stricta*	Snake/Columbia Shrub Steppe	c	AY165314	AS	AY257736		
Arab0296	*Boechera stricta*	South Central Rockies Forest West	ca	AY165323	AS	AY257736		
Arab0305	*Boechera stricta*	New England/Acadian Forest	e	AY165316	AS	AY257736		
Arab0311	*Boechera "holboellii"*	Cascade Mountains Leeward Forests	h	AY165348	CI	AY257778		
Arab0314	*Boechera "holboellii"*	Wasatch/Uinta Montane Forest	h	AY165348				
Arab0315	*Boechera "holboellii"*	Colorado Rockies Forest	bw, hb	AY165333, AY457932	AS	AY257736		
Arab0317	*Boechera collinsii*	Interior Yukon Alaska Lowland Taiga	l	AY165319	CG	AY257776		
Arab0318	*Boechera collinsii*	New England/Acadian Forest	h	AY165348				
Arab0319	*Boechera collinsii*	Canadian Aspen Forest and Parklands	h	AY165348				
Arab0320	*Boechera collinsii*	Canadian Aspen Forest and Parklands	h	AY165348	CG	AY257776		
Arab0322	*Boechera collinsii*	Northern Mixed Grasslands	h	AY165348	CF	AY257775		
Arab0326	*Boechera collinsii*	New England/Acadian Forest	h	AY165348	CG	AY257776		
Arab0329	*Boechera stricta*	Eastern Great Lakes Forest	e	AY165316	AS	AY257736		
Arab0330	*Boechera stricta*	Allegheny Highlands	e	AY165316	AS	AY257736		
Arab0332	*Boechera stricta*	Eastern Great Lakes Forest	e	AY165316	AS	AY257736		
Arab0333	*Boechera cf. stricta*	NE Coastal Forest	e	AY165316	AS	AY257736		
Arab0334	*Boechera stricta*	Allegheny Highlands	e	AY165316	AS	AY257736		
Arab0337	*Boechera "divaricarpa"*	Klamath Siskiyou Forests	cz	AY165394				
Arab0339	*Boechera stricta*	NE Coastal Forest	e	AY165316	AS	AY257736		
Arab0341	*Boechera stricta*	Wasatch/Uinta Montane Forest	v	AY165353	BD	AY257747		
Arab0343	*Boechera stricta*	Wasatch/Uinta Montane Forest	v	AY165353	AS	AY257736		
Arab0344	*Boechera stricta*	Colorado Plateau Shrub Steppe	c	AY165314	BK	AY257754		
Arab0346	*Boechera stricta*	Wasatch/Uinta Montane Forest	c	AY165314	AQ	AY257734		
Arab0347	*Boechera stricta*	Wasatch/Uinta Montane Forest	cq	AY165336	AS	AY257736		

Accession	Country	State/ Province	County	Herbarium	Herbarium number	Collector	Coll. number	X coordi-nate	Y coordi-nate
Arab0294	USA	Idaho	Custer	GH		R.C. & K.W. Rollins	83269	-114.33	44.51
Arab0295	USA	Idaho	Custer	GH		R.J. Davis	706	-113.43	44.28
Arab0296	USA	Idaho	Custer	GH		R.J. Davis	706		
Arab0305	USA	Maine	Oxford	GH		A.St. Pease		-70.68	44.51
Arab0311	USA	Washington	Kittitas	GH		C.L. Hitchcock, J.S. Martin	4731	-120.68	47.35
Arab0314	USA	Utah	Washington	GH		K. Thorne, J. Chandler. D. Nelson	4529	-111.71	41.08
Arab0315	USA	Wyoming	Albany	GH		B. Stein, W. Wagner, J. Miller, P. Lowry	1854	-106.25	41.35
Arab0317	Canada	Yukon		GH		C.C. Loan	441	-140.98	67.41
Arab0318	Canada	Quebec	Rimouski	GH		M.L. Fernald, J.F. Collins		-68.76	48.36
Arab0319	Canada	Manitoba		GH		H.J. Scoggan	11021	-100.68	49.39
Arab0320	Canada	Manitoba		GH		H.J. Scoggan, W.K.W. Baldwin	7203	-100.00	49.51
Arab0322	Canada	Alberta		GH		M.E. Moodie	823	-112.65	51.42
Arab0326	Canada	Quebec	Rimouski	GH		R.C. Rollins	55248, C.E. Wood Jr.	-68.76	48.36
Arab0329	USA	New York	Saratoga	GH		H.D. House	23457	-73.73	43.00
Arab0330	USA	New York	Tompkins	GH		K.M. Wiegand		-76.50	42.48
Arab0332	USA	New York	Saratoga	GH		H.D. House	23457	-73.73	43.00
Arab0333	USA	New York	Saratoga	GH		W.C. Muenscher, A.A. Lindsey	3336	-73.81	40.83
Arab0334	USA	New York	Tompkins	GH		K.M. Wiegand, F.P. Metcalf		-76.50	42.45
Arab0337	USA	Oregon	Jackson	GH				-122.72	42.08
Arab0339	USA	Rhode Island	Providence	GH		J.F. Collins		-71.46	41.96
Arab0341	USA	Utah	Duchesne						
Arab0343	USA	Utah	Daggett	GH		R.C. & K.W. Rollins	8655	-109.48	41.00
Arab0344	USA	Utah	Kane	GH		R.C. & K.W. Rollins	83185	-112.63	37.38
Arab0346	USA	Utah	Summit	GH		R.K. Vickery Jr.	654	-110.88	40.78
Arab0347	USA	Utah	Beaver	GH		S.L. Welsh, G. Moore	3288	-112.35	38.33

Accession	Taxon	wwf_ecoregion	ITS-type	ITS accession number	trnL/F type	trnLF accession numbers	*At2g25920 orthologue*	*At3g18900 orthologue*
Arab0349	*Boechera retrofracta*	Wyoming Basin Shrub Steppe	ac	AY165327	Y	AY257716		
Arab0350	*Boechera retrofracta*	South Central Rockies Forest East	h	AY165348	BY	AY257768		
Arab0355	*Boechera retrofracta*	South Central Rockies Forest East	ac	AY165327	U	AY257712		
Arab0356	*Boechera retrofracta*	South Central Rockies Forest East	h	AY165348	CI	AY257778		
Arab0358	*Boechera retrofracta*	South Central Rockies Forest East	h	AY165348	CY	AY257794		
Arab0359	*Boechera retrofracta*	Wasatch/Uinta Montane Forest	cs, ap	AY165365, AY165388	U	AY257712		
Arab0360	*Boechera retrofracta*	Wyoming Basin Shrub Steppe	r	AY165321	BJ	AY257753		
Arab0361	*Boechera retrofracta*	Wyoming Basin Shrub Steppe	h	AY165348	BX	AY257767		
Arab0363	*Boechera retrofracta*	Wyoming Basin Shrub Steppe	ct	AY165348	U	AY257712		
Arab0365	*Boechera retrofracta*	Colorado Rockies Forest	h	AY165348	BU	AY257764		
Arab0367	*Boechera "divaricarpa"*	Western Great Lakes Forest	cu	AY165355	AH	AY257725		
Arab0368	*Boechera "divaricarpa"*	Western Great Lakes Forest	cv	AY165323	AH	AY257725		
Arab0372	*Boechera "divaricarpa"*	Western Great Lakes Forest	cy, q, cx	AY165374, AY165392, AY165393	BY	AY257768		
Arab0376	*Boechera "divaricarpa"*	Upper Midwest Forest/Savanna Transition	y	AY165355	AS	AY257736		
Arab0378	*Boechera "divaricarpa"*	Central Tall Grasslands	e	AY165316	Z	AY257717		
Arab0380	*Boechera "divaricarpa"*	South Central Rockies Forest West	db	AY165319	BS	AY257762		
Arab0381	*Boechera "divaricarpa"*	South Central Rockies Forest East	dc, de, dd, df, dg	AY165396, AY165397, AY165398, AY165399, AY165400	BJ	AY257753		
Arab0384	*Boechera "divaricarpa"*	Great Basin Shrub Steppe West	dh	AY165353	AJ	AY257727		
Arab0385	*Boechera "divaricarpa"*	Eastern Great Lakes Forest	e	AY165316	AS	AY257736		
Arab0386	*Boechera "divaricarpa"*	Eastern Great Lakes Forest	e	AY165316	AS	AY257736		
Arab0387	*Boechera "divaricarpa"*	Eastern Great Lakes Forest	e	AY165316	AS	AY257736		
Arab0388	*Boechera "divaricarpa"*	Klamath Siskiyou Forests	di	AY165402	AH	AY257725		

Accession	Country	State/ Province	County	Herbarium	Herbarium number	Collector	Coll. number	X coordi-nate	Y coordi-nate
Arab0349	USA	Wyoming	Uinta	GH		R.C. & K.W. Rollins	79157	-110.43	41.31
Arab0350	USA	Wyoming	Fremont	GH		R.C. Rollins	79340, M.L. Rollins	-108.80	42.55
Arab0355	USA	Wyoming	Big Horn	GH		L.O. & R. Williams	3013	-107.90	44.85
Arab0356	USA	Wyoming	Park	GH		R.C. Rollins	57187, R.J. Davis	-110.63	44.95
Arab0358	USA	Wyoming	Sublette	GH		E.B. & L.B. Payson		-109.80	42.95
Arab0359	USA	Wyoming	Uinta	GH		R.C. Rollins	2305		
Arab0360	USA	Wyoming	Fremont-Hotsprings	GH		C.L. & M.W. Porter	8608	-108.06	43.46
Arab0361	USA	Wyoming	Big Horn						
Arab0363	USA	Wyoming	Lincoln	GH		E.B. Payson, G.M. Armstrong			
Arab0365	USA	Wyoming	Albany	GH		R.C. Rollins	1070	-106.16	41.33
Arab0367	USA	Michigan	Keweenaw	GH		A.S. Pease, E.C. Ogden			
Arab0368	USA	Michigan	Keweenaw	GH		M.L. Fernald, A.A. Pease			
Arab0372	USA	Michigan	Keweenaw	GH		A.S. Pease, E.C. Ogden			
Arab0376	USA	Minnesota	Wright	GH		J.W. Moore, B.O. Phennig			
Arab0378	USA	Minnesota	Rock	GH		Ph. Johnson			
Arab0380	USA	Montana	Beaverhead	GH		R.C. & K.W. Rollins	86183		
Arab0381	USA	Montana	Carbon						
Arab0384	USA	Nevada	Douglas	GH		M. Lavin	SW36		
Arab0385	USA	New York	Oswego	GH		N. Hotchkiss	3175	-76.15	43.65
Arab0386	USA	New York	Oswego	GH		N. Hotchkiss	2930	-76.18	43.60
Arab0387	USA	New York	Jefferson	GH		M.K. Hasbrouck			
Arab0388	USA	Oregon	Jackson	GH		R.C. & K.W. Rollins	81215		

Accession	Taxon	wwf_ecoregion	ITS-type	ITS accession number	trnL/F type	trnLF accession numbers	*At2g25920 orthologue*	*At3g18900 orthologue*
Arab0391	*Boechera "divaricarpa"*	South Central Rockies Forest East outpost I	dl, w	AY165355	CG	AY257776		
Arab0392	*Boechera "divaricarpa"*	Wasatch/Uinta Montane Forest	dm, dp, w, do, dn	AY165405, AY165406, AY165407, AY165408, AY165356	AS	AY257736		
Arab0395	*Boechera "divaricarpa"*	Wasatch/Uinta Montane Forest	n	AY165319	AW	AY257740		
Arab0395			dq	AY165409				
Arab0397	*Boechera "holboellii"*	Wasatch/Uinta Montane Forest North	h	AY165348	BU	AY257764		
Arab0398	*Boechera "divaricarpa"*	Wasatch/Uinta Montane Forest	r	AY165321	B	AY257693		
Arab0399	*Boechera "divaricarpa"*	Wasatch/Uinta Montane Forest	c	AY165314	AS	AY257736		
Arab0401	*Boechera "divaricarpa"*	Great Basin Shrub Steppe East	ds	AY165337	AS	AY257736		
Arab0402	*Boechera retrofracta*	Sierra Nevada North	h	AY165348	CQ	AY257788		
Arab0403	*Boechera retrofracta*	Sierra Nevada North	h	AY165348	T			
Arab0404	*Boechera retrofracta*	Klamath Siskiyou Forests	i	AY165317	I	AY257703		
Arab0405	*Boechera retrofracta*	Colorado Rockies Forest	cu	AY165355	AH	AY257725		
Arab0407	*Boechera retrofracta*	Colorado Plateau Shrub Steppe	dt	AY165410	U	AY257712		
Arab0409	*Boechera retrofracta*	Colorado Rockies Forest	h	AY165348	BU	AY257764		
Arab0412	*Boechera retrofracta*	Colorado Plateau Shrub Steppe	y	AY165355	AH	AY257725		
Arab0414	*Boechera retrofracta*	Colorado Rockies Forest	bt	AY165331	U	AY257712		
Arab0416	*Boechera retrofracta*	Colorado Rockies Forest	h	AY165348	BU	AY257764		
Arab0417	*Boechera retrofracta*	North Central Rockies Forest	h	AY165348	CX	AY257793		
Arab0418	*Boechera retrofracta*	South Central Rockies Forest West	h	AY165348	BU	AY257764		
Arab0421	*Boechera retrofracta*	Blue Mountain Forest	h	AY165348	CC	AY257772		
Arab0423	*Boechera "divaricarpa"*	South Central Rockies Forest West	t	AY165323	AH	AY257725		
Arab0424	*Boechera "divaricarpa"*	North Central Rockies Forest	az	AY165374	B	AY257693		
Arab0425	*Boechera "divaricarpa"*	North Central Rockies Forest	s	AY165322	BJ	AY257753		
Arab0426	*Boechera "divaricarpa"*	South Central Rockies Forest East	l	AY165319	AH	AY257725		
Arab0427	*Boechera "divaricarpa"*	South Central Rockies Forest West	t	AY165323	AH	AY257725		

Accession	Country	State/ Province	County	Herbarium	Herbarium number	Collector	Coll. number	X coordi-nate	Y coordi-nate
Arab0391	USA	South Dakota	Lawrence	GH		E.J. Palmer	37164		
Arab0392	USA	Utah	Wayne						
Arab0395	USA	Utah	Cache						
Arab0395									
Arab0397	USA	Utah	Daggett	GH		R.C. & K.W. Rollins	79141	-109.48	40.86
Arab0398	USA	Utah	Rich	GH		R.C. & K.W. Rollins	79321	-111.46	41.93
Arab0399	USA	Utah	Rich	GH		R.C. & K.W. Rollins	81332	-111.46	41.91
Arab0401	USA	Nevada	Nye						
Arab0402	USA	California	Placer	GH		L.C. Higgins	1743		
Arab0403	USA	California	Nevada	GH		L.C. Higgins	1742		
Arab0404	USA	California	Siskiyou						
Arab0405	USA	Colorado	Park	GH		R.C. & K.W. Rollins	8360		
Arab0407	USA	Colorado	Rio Blanco						
Arab0409	USA	Colorado	Routt	GH		W.A. Weber	6969	-106.83	40.75
Arab0412	USA	Colorado	Gunnison	GH		R.C. Rollins	2094a	-106.86	38.55
Arab0414	USA	Colorado	Gunnison	GH		R.C. Rollins	7957, K.W. Rollins	-106.60	38.55
Arab0416	USA	Colorado	Larimer	GH		R.C. Rollins	2402, T.S. Chambers	-105.76	40.38
Arab0417	USA	Idaho	Idaho						
Arab0418	USA	Idaho	Valley	GH		H.D. Ripley & C. Barneby	10695	-116.11	44.08
Arab0421	USA	Idaho	Idaho	GH		L. Constance	1854	-116.35	45.31
Arab0423	USA	Montana	Ravalli	GH		R.C. & K.W. Rollins	83297	-113.93	45.70
Arab0424	USA	Montana	Glacier	GH		A.St. Pease		-113.65	48.80
Arab0425	USA	Montana	Glacier	GH		L.C. Higgins	1677	-113.41	48.71
Arab0426	USA	Montana	Broadwater	GH		W.E. Booth	56381	-111.10	46.35
Arab0427	USA	Montana	Granite	GH		C.L. Hitchcock & C.V. Muhlick	14481	-113.76	46.25

Accession	Taxon	wwf_ecoregion	ITS-type	ITS accession number	trnL/F type	trnLF accession numbers	*At2g25920 orthologue*	*At3g18900 orthologue*
Arab0428	*Boechera "divaricarpa"*	Montana Valley Foothill Grasslands	ea	AY165411	AH	AY257725		
Arab0429	*Boechera "divaricarpa"*	South Central Rockies Forest East	r	AY165321	AV	AY257739		
Arab0431	*Boechera "divaricarpa"*	North Central Rockies Forest	az	AY165374	B	AY257693		
Arab0435	*Boechera "divaricarpa"*	Sierra Nevada	v	AY165353	AH	AY257725		
Arab0436	*Boechera "divaricarpa"*	Great Basin Shrub Steppe West	l	AY165319	BD	AY257747		
Arab0437	*Boechera "divaricarpa"*	Great Basin Shrub Steppe central East	ee	AY165338	CI	AY257778		
Arab0438	*Boechera "divaricarpa"*	Sierra Nevada	i	AY165317	AH	AY257725		
Arab0439	*Boechera "divaricarpa"*	Snake/Columbia Shrub Steppe	c	AY165314	BY	AY257768		
Arab0440	*Boechera "divaricarpa"*	Great Basin Shrub Steppe West	ef	EU275011	AJ	AY257727		
Arab0442	*Boechera "divaricarpa"*	Wasatch/Uinta Montane Forest	y	AY165355	AS	AY257736		
Arab0444	*Boechera "divaricarpa"*	Wasatch/Uinta Montane Forest	bx	AY165334	BO	AY257758		
Arab0445			eh	AY165412				
Arab0446	*Boechera "divaricarpa"*	Eastern Great Lakes Forest	e	AY165316	AS	AY257736		
Arab0447	*Boechera "divaricarpa"*	Eastern Great Lakes Forest	e	AY165316	AS	AY257736		
Arab0448	*Boechera "divaricarpa"*	Eastern Great Lakes Forest	e	AY165316	AS	AY257736		
Arab0449	*Boechera "divaricarpa"*	Cascade Mountains Leeward Forests	ei, ej, el, em	AY165413, AY165414, AY165416, AY165417	A	AY257692		
Arab0450	*Boechera "divaricarpa"*	Cascade Mountains Leeward Forests	h	AY165348	B	AY257693		
Arab0452	*Boechera "divaricarpa"*	Upper Midwest Forest/Savanna Transition	e	AY165316	AS	AY257736		
Arab0453	*Boechera "divaricarpa"*	South Central Rockies Forest East	s		BJ	AY257753		
Arab0454	*Boechera "divaricarpa"*	South Central Rockies Forest East	l	AY165319	AS	AY257736		
Arab0456	*Boechera "divaricarpa"*	South Central Rockies Forest East	k	AY165318	AS	AY257736		
Arab0457	*Boechera "divaricarpa"*	South Central Rockies Forest East	l	AY165319	AH	AY257725		
Arab0458	*Boechera "divaricarpa"*	South Central Rockies Forest East	k	AY165318	BS	AY257762		
Arab0461	*Boechera pendulocarpa*	Sierra Nevada	bt	AY165331				

Accession	Country	State/ Province	County	Herbarium	Herbarium number	Collector	Coll. number	X coordi-nate	Y coordi-nate
Arab0428	USA	Montana	Missoula						
Arab0429	USA	Montana	Gallatin	GH		E.B. & L.B. Payson		-111.10	44.66
Arab0431	USA	Montana	Lake	GH		H.D. Ripley & C. Barneby	10959	-114.10	48.38
Arab0435	USA	Nevada	Ormsby	GH		P. Train	3213	-119.90	39.15
Arab0436	USA	Nevada	Elko	GH		R.C. Rollins	2546, T.S. Chambers	-115.38	40.58
Arab0437	USA	Nevada	White Pine	GH		R.C. & K.W. Rollins	83202	-114.66	39.11
Arab0438	USA	Nevada	Douglas						
Arab0439	USA	Nevada	Humboldt	GH		A. Cronquist	8644	-118.71	41.68
Arab0440	USA	Nevada	Douglas						
Arab0442	USA	Utah	Garfield	GH		R.C. Rollins	57320	-111.60	38.00
Arab0444	USA	Utah	Daggett	GH		L.N. Goodding		-109.78	40.83
Arab0445									
Arab0446	USA	Vermont	Grand Isle	GH				-73.25	44.82
Arab0447	USA	Vermont		GH				-73.26	44.84
Arab0448	USA	Vermont	Chittenden	GH		C.G. Pringle		-73.18	44.50
Arab0449	USA	Washington	Kittitas	GH		B. Ertter	6534		
Arab0450	USA	Washington	Whatcom	GH		W.C. Muenscher	10089	-120.71	48.75
Arab0452	USA	Wisconsin	Door	GH		E.T. Kraus et al.		-87.16	45.15
Arab0453	USA	Wyoming	Sheridan						
Arab0454	USA	Wyoming	Teton	GH		A.A. Beetle	11895	-110.66	43.45
Arab0456	USA	Wyoming	Fremont	GH		Rollins	2046	-109.05	42.83
Arab0457	USA	Wyoming	Lincoln	GH		E.B. Payson, G.M. Armstrong		-110.83	42.71
Arab0458	USA	Wyoming	Park	GH		J.H. Beaman, K.J. Stone	1543	-110.07	44.40
Arab0461	USA	Nevada	Washoe	GH		A. Pinzl	1265	-119.91	39.35

Accession	Taxon	wwf_ecoregion	ITS-type	ITS accession number	trnL/F type	trnLF accession numbers	*At2g25920 orthologue*	*At3g18900 orthologue*
Arab0462	*Boechera pendulocarpa*	Great Basin Shrub Steppe central West	bt	AY165331	S	AY257710		
Arab0464	*Boechera pendulocarpa*	Great Basin Shrub Steppe central East	g	AY165347	CK	AY257780		
Arab0465	*Boechera pendulocarpa*	Wasatch/Uinta Montane Forest North	ab	AY165326	BU	AY257764		
Arab0466	*Boechera pendulocarpa*	Great Basin Shrub Steppe East	au	AY165370	S	AY257710		
Arab0469	*Boechera pendulocarpa*	Great Basin Shrub Steppe East	bt	AY165331	BP	AY257759		
Arab0470	*Boechera pendulocarpa*	Okanogan Forest	bt	AY165331	K	AY257702		
Arab0471	*Boechera pendulocarpa*	Wyoming Basin Shrub Steppe	ab	AY165326	CR	AY257789		
Arab0473	*Boechera pendulocarpa*	South Central Rockies Forest East	bt	AY165331	CL	AY257781		
Arab0474	*Boechera pendulocarpa*	South Central Rockies Forest East	bt	AY165331	M	AY257704		
Arab0475	*Boechera pendulocarpa*	South Central Rockies Forest East	bt	AY165331	CL	AY257781		
Arab0477	*Boechera retrofracta*	Sierra Nevada North	er	AY165339	S	AY257710		
Arab0478	*Boechera pinetorum*	Sierra Nevada South	z	AY165325	CQ	AY257788		
Arab0479	*Boechera pinetorum*	Sierra Nevada South	h	AY165348	CM	AY257782		
Arab0480	*Boechera retrofracta*	Great Basin Shrub Steppe East	h	AY165348				
Arab0481	*Boechera retrofracta*	South Central Rockies Forest West	h	AY165348	BU	AY257764		
Arab0482	*Boechera retrofracta*	North Central Rockies Forest	h	AY165348	BU	AY257764		
Arab0483	*Boechera retrofracta*	South Central Rockies Forest West	h	AY165348	CI	AY257778		
Arab0486	*Boechera retrofracta*	South Central Rockies Forest West	h	AY165348	CI	AY257778		
Arab0487a	*Boechera retrofracta*	South Central Rockies Forest West	h	AY165348	CI	AY257778		
Arab0487b	*Boechera cf. "macounii"*	South Central Rockies Forest West	h	AY165348				
Arab0488	*Boechera retrofracta*	Great Basin Shrub Steppe East	h	AY165348	U	AY257712		
Arab0490	*Boechera retrofracta*	Snake/Columbia Shrub Steppe	h	AY165348	CI	AY257778		
Arab0494	*Boechera retrofracta*	South Central Rockies Forest West	h	AY165348	U	AY257712		
Arab0496	*Boechera retrofracta*	Western Great Lakes Forest	h	AY165348	BY	AY257768		
Arab0499	*Boechera retrofracta*	Montana Valley Foothill Grasslands	h	AY165348	BU	AY257764		
Arab0500	*Boechera retrofracta*	Great Basin Shrub Steppe West	eu	AY165340	S	AY257710		
Arab0501	*Boechera retrofracta*	Great Basin Shrub Steppe West	ev	AY165341	CI	AY257778		
Arab0502	*Boechera retrofracta*	Great Basin Shrub Steppe West	h	AY165348	U	AY257712		

Accession	Country	State/ Province	County	Herbarium	Herbarium number	Collector	Coll. number	X coordi-nate	Y coordi-nate
Arab0462	USA	Nevada	Nye	GH		R.C. & K.W. Rollins	79225	-117.28	38.95
Arab0464	USA	Nevada	White Pine						
Arab0465	USA	Utah	Rich	GH		R.C. & K.W. Rollins	79310	-111.46	41.93
Arab0466	USA	Utah	Box Elder	GH		R.C. & K.W. Rollins	81260	-113.85	41.76
Arab0469	USA	Utah	Box Elder	GH		S.J. Preece Jr.	578	-113.51	41.93
Arab0470	USA	Washington	Stevens	GH		H.T. Rogers	268 et al.	-118.15	48.23
Arab0471	USA	Wyoming	Lincoln						
Arab0473	USA	Wyoming	Fremont						
Arab0474	USA	Wyoming	Fremont						
Arab0475	USA	Wyoming	Fremont						
Arab0477	USA	California	Nevada	GH		J.Th. Howell		-120.35	39.36
Arab0478	USA	California	Fresno	GH		T. Ross	3081	-118.74	36.86
Arab0479	USA	California	Tulare	GH		E.C. Twisselmann et al.	18095	-118.26	36.20
Arab0480	USA	Idaho	Caribou						
Arab0481	USA	Idaho	Lemhi	GH		C. L	3685		
Arab0482	USA	Idaho	Kootenai	GH		J.H. Christ	10261	-116.88	47.96
Arab0483	USA	Idaho	Blaine	GH		A. Cronquist	2612	-114.86	43.91
Arab0486	USA	Idaho	Valley	GH		J.W. Thompson	13741	-115.50	44.85
Arab0487a	USA	Idaho	Custer	GH		J.F. Macbride, E.B. Payson		-114.38	44.56
Arab0487b	USA	Idaho	Custer	GH		J.F. Macbride, E.B. Payson		-114.38	44.56
Arab0488	USA	Idaho	Cassia	GH		R.C. & K.W. Rollins	86189	-113.68	42.30
Arab0490	USA	Idaho	Clark	GH		R.J. Davis	339	-112.30	44.26
Arab0494	USA	Idaho	Custer	GH		C.L. Hitchcock & C.V. Muhlick	8998	-114.35	44.25
Arab0496	USA	Michigan	Keweenaw	GH		M.L. Fernald, A.A. Pease		-87.95	47.46
Arab0499	USA	Montana	Ravalli						
Arab0500	USA	Nevada	Humboldt						
Arab0501	USA	Nevada	Washoe						
Arab0502	USA	Nevada	Humboldt	GH		R.C. & K.W. Rollins	79246	-118.43	41.30

Accession	Taxon	wwf_ecoregion	ITS-type	ITS accession number	trnL/F type	trnLF accession numbers	*At2g25920 orthologue*	*At3g18900 orthologue*
Arab0503	*Boechera retrofracta*	Great Basin Shrub Steppe West	ew, ex, ag	AY165348, AY165419, AY165358	S	AY257710		
Arab0504	*Boechera retrofracta*	Great Basin Shrub Steppe West	ev	AY165341	U	AY257712		
Arab0510	*Boechera retrofracta*	Sierra Nevada North	z	AY165325	CQ	AY257788		
Arab0512	*Boechera retrofracta*	Great Basin Shrub Steppe central West	au	AY165370	N	AY257705		
Arab0514	*Boechera retrofracta*	Great Basin Shrub Steppe central East	fb	AY165342	BY	AY257768		
Arab0515	*Boechera retrofracta*	Snake/Columbia Shrub Steppe	h	AY165348	U	AY257712		
Arab0516	*Boechera retrofracta*	Snake/Columbia Shrub Steppe	ac	AY165327	U	AY257712		
Arab0526	*Boechera retrofracta*	South Central Rockies Forest East	h	AY165348	BU	AY257764		
Arab0527	*Boechera retrofracta*	South Central Rockies Forest West	h	AY165348	CX	AY257793		
Arab0532	*Boechera retrofracta*	Great Basin Shrub Steppe central West	au	AY165370	U	AY257712		
Arab0534	*Boechera retrofracta*	Great Basin Shrub Steppe West	h	AY165348	M	AY257704		
Arab0535	*Boechera retrofracta*	Sierra Nevada North	h	AY165348	CI	AY257778		
Arab0537	*Boechera retrofracta*	Great Basin Shrub Steppe central East	ac	AY165327				
Arab0538	*Boechera retrofracta*	Great Basin Shrub Steppe central East	h	AY165348	CS	AY257790		
Arab0539	*Boechera pendulocarpa*	South Central Rockies Forest East outpost II	ab	AY165326	CR	AY257789		
Arab0543	*Boechera pendulocarpa*	South Central Rockies Forest East	bt	AY165331	M	AY257704		
Arab0544	*Boechera pendulocarpa*	South Central Rockies Forest East	bt	AY165331	CR	AY257789		
Arab0545	*Boechera pendulocarpa*	South Central Rockies Forest East	bt	AY165331	M	AY257704	GQ166234	
Arab0547	*Boechera pendulocarpa*	South Central Rockies Forest East	bt	AY165331	Q	AY257708		
Arab0548	*Boechera "divaricarpa"*	South Central Rockies Forest East	fh, dn	AY165331				
Arab0549	*Boechera pendulocarpa*	South Central Rockies Forest East	bx	AY165334				
Arab0550	*Boechera pendulocarpa*	South Central Rockies Forest East	bx	AY165334	M	AY257704		
Arab0551	*Boechera pinetorum*	Colorado Plateau Shrub Steppe	fi, fj	AY165421	F	AY257697		
Arab0558a	*Boechera pinetorum*	Sierra Nevada South	z	AY165325	CQ	AY257788		

Accession	Country	State/ Province	County	Herbarium	Herbarium number	Collector	Coll. number	X coordi-nate	Y coordi-nate
Arab0503	USA	Nevada	Mineral	GH		G.T. Austin			
Arab0504	USA	Nevada	Churchill	GH		G.T. Austin		-117.86	39.58
Arab0510	USA	Nevada	Washoe						
Arab0512	USA	Nevada	Nye						
Arab0514	USA	Nevada	Nye						
Arab0515	USA	Nevada	Humboldt						
Arab0516	USA	Nevada	Humboldt						
Arab0526	USA	Montana	Park	GH		J.G. Witt	1687	-109.95	45.01
Arab0527	USA	Montana	Ravalli	GH		R.C. Rollins	57216	-113.93	45.73
Arab0532	USA	Nevada	Lander						
Arab0534	USA	Nevada	Mineral						
Arab0535	USA	Nevada	Washoe	GH		W.A. Archer	5992	-119.90	39.33
Arab0537	USA	Nevada	Nye	GH		A. Eastwood & J.Th. Howell		-115.26	38.81
Arab0538	USA	Nevada	Elko	GH		B. Maguire, A.H. Holmgren		-115.46	41.83
Arab0539	USA	Wyoming	Johnson						
Arab0543	USA	Wyoming	Sheridan	GH		A.A. Beetle	11882	-107.51	44.78
Arab0544	USA	Wyoming	Park	GH		L.O. & R.P. Williams		-109.75	44.93
Arab0545	USA	Wyoming	Park						
Arab0547	USA	Wyoming	Park	GH		R.C. Rollins	51284a, C.L. Porter	-110.63	44.95
Arab0548	USA	Wyoming	Park	GH		R.C. Rollins	51284b, C.L. Porter		
Arab0549	USA	Wyoming	Johnson	GH		R.C. & K.W. Rollins	81404	-106.98	43.96
Arab0550	USA	Wyoming	Fremont	GH		R.C. & K.W. Rollins	81380	-108.76	42.53
Arab0551	USA	Arizona	Navajo	GH		R.H. Peebles	13438		
Arab0558a	USA	California	Madera	GH		J.Th. Howell		-119.06	37.65

Accession	Taxon	wwf_ecoregion	ITS-type	ITS accession number	trnL/F type	trnLF accession numbers	*At2g25920 orthologue*	*At3g18900 orthologue*
Arab0561	*Boechera "divaricarpa"*	Colorado Rockies Forest	l	AY165319	AH	AY257725		
Arab0562	*Boechera "divaricarpa"*	Wyoming Basin Shrub Steppe	fl	AY165343	AO	AY257732		
Arab0564	*Boechera "divaricarpa"*	Colorado Rockies Forest	l	AY165319	CI	AY257778		
Arab0566	*Boechera "divaricarpa"*	Colorado Plateau Shrub Steppe West	t	AY165323	CI	AY257778		
Arab0568	*Boechera "divaricarpa"*	Colorado Rockies Forest	l	AY165319	AF	AY257723		
Arab0570	*Boechera "divaricarpa"*	South Central Rockies Forest West	fn	AY165423	AH	AY257725		
Arab0571	*Boechera "divaricarpa"*	South Central Rockies Forest West	bt	AY165331	AS	AY257736		
Arab0572	*Boechera "divaricarpa"*	South Central Rockies Forest West	fo	AY165424	AS	AY257736		
Arab0572	*Boechera "divaricarpa"*	South Central Rockies Forest West	n	AY165349	AS	AY257736		
Arab0573	*Boechera "divaricarpa"*	Colorado Rockies Forest	l	AY165319	BJ	AY257753		
Arab0576	*Boechera "divaricarpa"*	South Central Rockies Forest West	t	AY165323	AH	AY257725		
Arab0577	*Boechera "divaricarpa"*	Blue Mountain Forest	h	AY165348	AH	AY257725		
Arab0582	*Boechera retrofracta*	Okanogan Forest	h	AY165348	CU	AY257786		
Arab0585	*Boechera retrofracta*	Interior Yukon Alaska Tundra					GQ166235	
Arab0586	*Boechera retrofracta*	Alaska/St. Elias Range Tundra	h	AY165348	BY	AY257768		
Arab0588	*Boechera retrofracta*	Interior Yukon Alaska Tundra	h	AY165348	BY	AY257768		
Arab0590	*Boechera collinsii*	Yukon Interior Dry Forest	h	AY165348	BY	AY257768		
Arab0592	*Boechera retrofracta*	Interior Yukon Alaska Lowland Taiga	h	AY165348	BY	AY257768		
Arab0594	*Boechera retrofracta*	Alaska/St. Elias Range Tundra	h	AY165348	BY	AY257768		
Arab0603	*Boechera "divaricarpa"*	South Central Rockies Forest West	fq, fr	AY165425	BT	AY257763		
Arab0606	*Boechera "divaricarpa"*	South Central Rockies Forest West	t	AY165323	AH	AY257725		
Arab0607	*Boechera "divaricarpa"*	Wasatch/Uinta Montane Forest	l	AY165319	D	AY257695		
Arab0608	*Boechera "divaricarpa"*	Snake/Columbia Shrub Steppe	h	AY165348	AH	AY257725		
Arab0609	*Boechera "divaricarpa"*	South Central Rockies Forest West	c	AY165314	AS	AY257736		
Arab0611	*Boechera "divaricarpa"*	South Central Rockies Forest East	h	AY165348	BC	AY257746		
Arab0613	*Boechera "divaricarpa"*	Wasatch/Uinta Montane Forest	ft	AY165427	B	AY257693		
Arab0614	*Boechera "divaricarpa"*	Wasatch/Uinta Montane Forest	r	AY165321	D	AY257695		
Arab0621	*Boechera retrofracta*	Klamath Siskiyou Forests	i	AY165317				

Accession	Country	State/ Province	County	Herbarium	Herbarium number	Collector	Coll. number	X coordi-nate	Y coordi-nate
Arab0561	USA	Colorado	San Juan	GH		A.R. Hodgdon, G.B. Rossbach		-107.66	37.81
Arab0562	USA	Colorado	Moffat	GH		R.C. Rollins	2227	-107.58	40.46
Arab0564	USA	Colorado	Grand	GH		R.C. Rollins	1040	-106.08	40.25
Arab0566	USA	Colorado	La Plata	GH		J. Bequaert		-107.56	37.23
Arab0568	USA	Colorado	Hinsdale	GH		L.C. Higgins	2237	-107.28	38.00
Arab0570	USA	Idaho	Custer	GH		R.C. Rollins	86173	-114.84	44.36
Arab0571	USA	Idaho	Custer	GH		R.C. & K.W. Rollins	86166	-114.83	44.10
Arab0572	USA	Idaho	Custer	GH		R.C. Rollins	86159	-114.83	44.1
Arab0572	USA	Idaho	Custer	GH		R.C. & K.W. Rollins	86159		
Arab0573	USA	Wyoming	Albany	GH		R.C. & K.W. Rollins	8617	-105.45	41.31
Arab0576	USA	Idaho	Lemhi	GH		R.C. Rollins	57217	-113.96	45.63
Arab0577	USA	Idaho	Idaho						
Arab0582	Canada	British Columbia		GH		T.T. McCare	1891	-119.91	51.67
Arab0585	Canada	Yukon		GH		H.M. & L.C. Raup	12437	-138.51	61.05
Arab0586	Canada	Yukon		GH		H.M. & L.C. Raup	12199	-138.51	61.05
Arab0588	Canada	Yukon		GH		A.E. Porsild, A.J. Breitung		-135.00	64.00
Arab0590	Canada	Yukon		GH		J.M. Gillett	3176		
Arab0592	USA	Alaska		GH		W.J. Cody	5064	-146.91	64.38
Arab0594	USA	Alaska		GH		E. Scamman	5095	-150.81	63.36
Arab0603	USA	Idaho	Idaho	GH		P.A. Munz, C. Davidson	18216		
Arab0606	USA	Idaho	Lemhi	GH		R.C. & K.W. Rollins	86186	-113.45	45.00
Arab0607	USA	Idaho	Bear Lake	GH		R.J. Davis	395		
Arab0608	USA	Idaho	Lemhi	GH		C.L. Hitchcock, C.V. Muhlick	9277	-113.50	44.68
Arab0609	USA	Idaho	Blaine	GH		J.F. Macbride, E.B. Payson		-114.71	43.86
Arab0611	USA	Idaho	Bonneville	GH		R.C. & K.W. Rollins	79298	-111.25	43.56
Arab0613	USA	Idaho	Bear Lake	GH		R.C. & K.W. Rollins	81324		
Arab0614	USA	Idaho	Bear Lake	GH		R.C. & K.W. Rollins	81325	-111.58	42.21
Arab0621	USA	California	Siskiyou	GH		S.K. Harris, R. Leland	5483	-122.96	41.36

Accession	Taxon	wwf_ecoregion	ITS-type	ITS accession number	trnL/F type	trnLF accession numbers	*At2g25920 orthologue*	*At3g18900 orthologue*
Arab0624	*Boechera retrofracta*	Klamath Siskiyou Forests	bt	AY165331	S	AY257710		
Arab0626	*Boechera retrofracta*	Eastern Cascades Forest	h	AY165348	CI	AY257778		
Arab0627	*Boechera retrofracta*	Sierra Nevada North	h	AY165348	CI	AY257778		
Arab0628	*Boechera retrofracta*	Snake/Columbia Shrub Steppe	h	AY165348	CI	AY257778		
Arab0629	*Boechera retrofracta*	Klamath Siskiyou Forests	i	AY165317	CI	AY257778		
Arab0631	*Boechera retrofracta*	Klamath Siskiyou Forests	i	AY165317	CB	AY257771		
Arab0640	*Boechera stricta*	North Central Rockies Forest	t	AY165323	AH	AY257725		
Arab0641	*Boechera stricta*	Northern Transitional Alpine Forests	az	AY165374	AH	AY257725		
Arab0643	*Boechera stricta*	Cascade Mountains Leeward Forests	t	AY165323	AH	AY257725		
Arab0644	*Boechera stricta*	Northern Cordillera Forest	az	AY165374	AH	AY257725		
Arab0645	*Boechera stricta*	Muskwa/Slave Lakes Forest	az	AY165374	AH	AY257725		
Arab0646	*Boechera stricta*	Cascade Mountains Leeward Forests	az	AY165374	AH	AY257725		
Arab0647	*Boechera stricta*	Yukon Interior Dry Forest	az	AY165374	AH	AY257725		
Arab0648	*Boechera stricta*	Colorado Plateau Shrub Steppe	l	AY165319	AS	AY257736		
Arab0649	*Boechera stricta*	Great Basin Shrub Steppe West	az	AY165374	AJ	AY257727		
Arab0650	*Boechera stricta*	Great Basin Shrub Steppe West	r	AY165321	AS	AY257736		
Arab0652	*Boechera stricta*	Colorado Rockies Forest	l	AY165319	BD	AY257747		
Arab0656	*Boechera "divaricarpa"*	Eastern Great Lakes Forest	e	AY165316	AS	AY257736		
Arab0659	*Boechera "divaricarpa"*	Eastern Great Lakes Forest	e	AY165316	AS	AY257736		
Arab0661	*Boechera "divaricarpa"*	Cascade Mountains Leeward Forests	cy, fw	AY165428	BJ	AY257753		
Arab0663	*Boechera "divaricarpa"*	Muskwa/Slave Lakes Forest	az	AY165374	BY	AY257768		

Accession	Country	State/ Province	County	Herbarium	Herbarium number	Collector	Coll. number	X coordi-nate	Y coordi-nate
Arab0624	USA	California	Siskiyou	GH		C.L. Hitchcock, J.S. Martin	5310	-123.33	41.58
Arab0626	USA	California	Modoc	GH		B. Bartholomew	6347	-121.41	41.28
Arab0627	USA	California	Lassen	GH		R.C. & K.W. Rollins	81174	-120.70	40.48
Arab0628	USA	California	Lassen	GH		R.C. & K.W. Rollins	81177	-120.71	40.73
Arab0629	USA	California	Trinity	GH		A. Eastwood & J.Th. Howell		-122.70	40.96
Arab0631	USA	California	Siskiyou	GH		L. Constance, R.C. Rollins		-123.11	41.66
Arab0640	Canada	British Columbia		GH		R.L. Taylor, J.A. Calder, D.H. Ferguson	3408	-114.20	49.03
Arab0641	Canada	British Columbia		GH		J.A. Calder, D.B.O. Savile, J.M. Ferguson	14661	-127.42	54.76
Arab0643	Canada	British Columbia		GH		J.W. & E.M. Thompson	404	-121.70	50.60
Arab0644	Canada	British Columbia		GH		H.M. Raup & D.S. Correll	10813	-124.56	58.51
Arab0645	Canada	British Columbia		GH		H.M. Raup & D.S. Correll	10242	-121.40	57.37
Arab0646	Canada	British Columbia		GH		J.W. & E.M. Thompson	404	-121.70	50.60
Arab0647	Canada	Yukon		GH		A.E. Porsild, A.J. Breitung	10539	-133.00	61.00
Arab0648	USA	Arizona		GH		A. Eastwood & J.Th. Howell		-112.08	36.10
Arab0649	USA	California	Mono	GH		A.M. Alexander, L. Kellogg	4570	-119.35	38.43
Arab0650	USA	California	Mono						
Arab0652	USA	Colorado	Huerfano	GH		S.D. McKelvey	4821	-105.20	37.59
Arab0656	Canada	Ontario	Bruce	GH				-80.98	44.96
Arab0659	Canada	Ontario	Bruce	GH		J.R. Breed et al.	134	-81.18	44.96
Arab0661	Canada	British Columbia		GH		J.W. & E.M. Thompson			
Arab0663	Canada	British Columbia		GH		H.M. Raup, D.S. Correll	10208	-122.71	57.23

Accession	Taxon	wwf_ecoregion	ITS-type	ITS accession number	trnL/F type	trnLF accession numbers	*At2g25920 orthologue*	*At3g18900 orthologue*
Arab0664	*Boechera "divaricarpa"*	Northern Cordillera Forest	l	AY165319	CA	AY257770		
Arab0667	*Boechera "divaricarpa"*	Eastern Cascades Forest	t	AY165323	AJ	AY257727		
Arab0669	*Boechera "divaricarpa"*	Klamath Siskiyou Forests	fy	AY165344	CI	AY257778		
Arab0670	*Boechera "divaricarpa"*	Klamath Siskiyou Forests	fy	AY165344	CI	AY257778		
Arab0671	*Boechera pinetorum*	Colorado Plateau Shrub Steppe	fz, an	AY165327	AH	AY257725		
Arab0674	*Boechera pinetorum*	Great Basin Shrub Steppe East	f	AY165346	U	AY257712		
Arab0678	*Boechera pinetorum*	Colorado Plateau Shrub Steppe	gb	AY165430	AS	AY257736		
Arab0681	*Boechera "holboellii"*	Eastern Cascades Forest	bx	AY165334	M	AY257704		
Arab0682	*Boechera pinetorum*	Wyoming Basin Shrub Steppe	ap	AY165366	AH	AY257725		
Arab0683	*Boechera pinetorum*	South Central Rockies Forest East	gd	AY165431	B	AY257693		
Arab0684	*Boechera pinetorum*	South Central Rockies Forest East	gf	AY165432	AH	AY257725		
Arab0685	*Boechera cf. divaricarpa*	South Central Rockies Forest East outpost I	be	AY165377	CG	AY257776		
Arab0689	*Boechera pinetorum*	Great Basin Shrub Steppe East	aj	AY165361	AC	AY257720		
Arab0692	*Boechera pinetorum*	Great Basin Shrub Steppe central West	an, ag	AY165364, AY165358	S	AY257710		
Arab0694	*Boechera pinetorum*	Great Basin Shrub Steppe central West	ad	AY165328	U	AY257712		
Arab0695	*Boechera pinetorum*	Great Basin Shrub Steppe central East	gg, ap	AY165433, AY165366	BY	AY257768		
Arab0697	*Boechera pinetorum*	Great Basin Shrub Steppe central West	w	AY165347	S	AY257710		
Arab0701	*Boechera pinetorum*	Great Basin Shrub Steppe central West	h	AY165348				
Arab0703	*Boechera cf. "holboellii"*	Eastern Cascades Forest	gj, gk, gl	AY457929, AY457930, AY457931	L	AY257703		
Arab0704	*Boechera pinetorum*	Snake/Columbia Shrub Steppe	ap	AY165366	BY	AY257768		
Arab0707	*Boechera pinetorum*	Great Basin Shrub Steppe East	f	AY165346	U	AY257712		
Arab0711	*Boechera "divaricarpa"*	Sierra Nevada	gm	AY165345	AH	AY257725		
Arab0715	*Boechera "divaricarpa"*	Eastern Cascades Forest	ek	AY165415	A	AY257692		

Accession	Country	State/ Province	County	Herbarium	Herbarium number	Collector	Coll. number	X coordi-nate	Y coordi-nate
Arab0664	Canada	Yukon		GH		H.M. Raup, W.H. Drury, K.A. Raup	13105	-137.00	60.00
Arab0667	USA	California	Modoc	GH		M.J. Williams	84-79-2	-120.63	41.48
Arab0669	USA	California	Siskiyou						
Arab0670	USA	California	Siskiyou						
Arab0671	USA	Utah	Kane	GH		R.C. & K.W. Rollins	79182		
Arab0674	USA	Utah	Iron	GH		R.C. & K.W. Rollins	8193	-113.43	37.65
Arab0678	USA	Utah	Carbon	GH		R.C. & K.W. Rollins	83120		
Arab0681	USA	Washington	Kittitas	GH		J.W. Thompson	14773	-120.95	46.96
Arab0682	USA	Wyoming	Carbon	GH		R.C. Rollins	79346, M.L. Rollins		
Arab0683	USA	Wyoming	Sheridan						
Arab0684	USA	Wyoming	Fremont						
Arab0685	USA	Wyoming	Crook						
Arab0689	USA	Nevada	Clark	GH		A. Cronquist, P.V. Wells, J.C. Beatley		-115.10	36.66
Arab0692	USA	Nevada	Nye	GH		J. Beatley			
Arab0694	USA	Nevada	Nye	GH		J. Beatley		-116.11	37.46
Arab0695	USA	Nevada	White Pine	GH		A. Pinzl	3450		
Arab0697	USA	Nevada	Nye	GH		J. Beatley			
Arab0701	USA	Nevada	Elko						
Arab0703	USA	Oregon	Klamath						
Arab0704	USA	Oregon	Harney	GH		R.C. & K.W. Rollins	83231		
Arab0707	USA	Utah	Iron	GH		R.C. & K.W. Rollins	8195	-113.43	37.65
Arab0711	USA	Nevada	Douglas						
Arab0715	USA	California	Modoc	GH		A.M. Alexander, L. Kellogg	4880		

Accession	Taxon	wwf_ecoregion	ITS-type	ITS accession number	trnL/F type	trnLF accession numbers	*At2g25920 orthologue*	*At3g18900 orthologue*
Arab0716	*Boechera "divaricarpa"*	Klamath Siskiyou Forests	t	AY165323	AJ	AY257727		
Arab0718	*Boechera "divaricarpa"*	Sierra Nevada South	t	AY165323	CI	AY257778		
Arab0722	*Boechera "divaricarpa"*	Klamath Siskiyou Forests	t	AY165323	AJ	AY257727		
Arab0730	*Boechera stricta*	Upper Midwest Forest/Savanna Transition	s	AY165322	AS	AY257736		
Arab0735	*Boechera retrofracta*	Blue Mountain Forest	gq	AY165331	BU	AY257764		
Arab0736	*Boechera retrofracta*	Central and Southern Cascades Forest	h	AY165348	BU	AY257764		
Arab0741	*Boechera retrofracta*	Great Basin Shrub Steppe East	gr	AY165435	U	AY257712		
Arab0753	*Boechera retrofracta*	Eastern Great Lakes Forest	h	AY165348	BU	AY257764		
Arab0754	*Boechera retrofracta*	Yukon Interior Dry Forest	gs	AY165348	BY	AY257768		
Arab0755	*Boechera retrofracta*	Cascade Mountains Leeward Forests	gu	AY165348	BY	AY257768		
Arab0761	*Boechera retrofracta*	Alberta Mountain Forest	gv	AY165348	BY	AY257768		
Arab0762	*Boechera retrofracta*	North Central Rockies Forest	gw	AY165439	CG	AY257776		
Arab0765	*Boechera retrofracta*	Montana Valley Foothill Grasslands	gx	EU274871	CG	AY257776		
Arab0771	*Boechera retrofracta*	South Central Rockies Forest West	gy	EU274873				
Arab0774	*Boechera retrofracta*	South Central Rockies Forest West	h	AY165348	BU	AY257764		
Arab0775	*Boechera retrofracta*	South Central Rockies Forest West	h	AY165348	CD	AY257773		
Arab0779	*Boechera stricta*	Eastern Canadian Forests	e	AY165316	AS	AY257736		
Arab0781	*Boechera stricta*	Eastern Great Lakes Forest	gz	EU274878	AS	AY257736		
Arab0783	*Boechera retrofracta*	Wasatch/Uinta Montane Forest North	h	AY165348	BU	AY257764		
Arab0785	*Boechera retrofracta*	North Central Rockies Forest	ha	EU274883				
Arab0788	*Boechera retrofracta*	Snake/Columbia Shrub Steppe	bj	AY165329	R	AY257709		
Arab0789	*Boechera retrofracta*	Cascade Mountains Leeward Forests	h	AY165348	BU	AY257764		
Arab0792	*Boechera retrofracta*	Okanogan Forest	h	AY165348	BU	AY257764		
Arab0796	*Boechera stricta*	Great Basin Shrub Steppe East	v	AY165353	AH	AY257725		
Arab0797	*Boechera stricta*	Great Basin Shrub Steppe East	r	AY165321	AS	AY257736		

Accession	Country	State/ Province	County	Herbarium	Herbarium number	Collector	Coll. number	X coordi-nate	Y coordi-nate
Arab0716	USA	California	Siskiyou	GH		D. Parker	282		
Arab0718	USA	California	Tulare	GH		R.S. Ferris, L. Lorraine		-118.60	36.45
Arab0722	USA	California	Siskiyou	GH		W. Parker	282		
Arab0730	USA	Wisconsin	Calumet	GH		N.C. Fassett		-88.30	44.15
Arab0735	USA	Oregon	Baker	GH		R.C. Rollins	2632, T.S. Chambers		
Arab0736	USA	Oregon	Lane	GH		A. Eastwood & J.Th. Howell			
Arab0741	USA	Utah	Box Elder						
Arab0753	Canada	Ontario	Simcoe	GH		J.H. Soper, H.M. Dale	3816		
Arab0754	Canada	Yukon		GH		A.E. Porsild, A.J. Breitung			
Arab0755	Canada	British Columbia		GH		J.W. & E.M. Thompson			
Arab0761	Canada	Alberta		GH		E. Scamman	3054		
Arab0762	Canada	Alberta		GH		J. Macoun			
Arab0765	Canada	Alberta		GH		W.C. McCalla		-114.08	51.08
Arab0771	USA	Idaho	Custer	GH		R.C. & K.W. Rollins	79287	-113.83	44.21
Arab0774	USA	Idaho	Lemhi	GH		R.C. Rollins	57247b	-113.56	44.60
Arab0775	USA	Idaho	Custer	GH		R.C. & K.W. Rollins	83276	-113.76	44.65
Arab0779	Canada			GH		M.L. Fernald, B. Long, J.M. Fogg, Jr.		-57.91	49.50
Arab0781	Canada	Quebec	Ottawa	GH		B. Boivin, L. Jenkins, L.J. Van Rens	14293	-76.01	45.55
Arab0783	USA	Utah	Uintah	GH		R.C. Rollins	1765	-109.73	40.63
Arab0785	USA	Washington	Pend Oreille						
Arab0788	USA	Washington	Douglas	GH		J.W. Thompson	14620	-120.11	47.41
Arab0789	USA	Washington	Okanogan	GH		O.T. Edwards	226	-120.11	48.36
Arab0792	USA	Washington	Okanogan	GH		J.W. Thompson	7130	-119.10	48.70
Arab0796	USA	Nevada	Elko						
Arab0797	USA	Nevada	White Pine						

Accession	Taxon	wwf_ecoregion	ITS-type	ITS accession number	trnL/F type	trnLF accession numbers	*At2g25920 orthologue*	*At3g18900 orthologue*
Arab0798	*Boechera stricta*	Great Basin Shrub Steppe East	r	AY165321	BD	AY257747		
Arab0802	*Boechera stricta*	Great Basin Shrub Steppe East	c	AY165314	AS	AY257736		
Arab0803	*Boechera stricta*	Great Basin Shrub Steppe West	c	AY165314	BE	AY257748		
Arab0804	*Boechera stricta*	Great Basin Shrub Steppe East	r	AY165321	BD	AY257747		
Arab0806	*Boechera "divaricarpa"*	Sierra Nevada	az	AY165374	AH	AY257725		
Arab0807	*Boechera stricta*	Great Basin Shrub Steppe West	v	AY165353	AH	AY257725		
Arab0812	*Boechera stricta*	Colorado Rockies Forest	l	AY165319	AH	AY257725		
Arab0813	*Boechera stricta*	Colorado Rockies Forest	l	AY165319	BI	AY257752		
Arab0814	*Boechera stricta*	Colorado Rockies Forest	l	AY165319	AO	AY257732		
Arab0815	*Boechera stricta*	Colorado Rockies Forest	l	AY165319	BG	AY257750		
Arab0817	*Boechera stricta*	Colorado Rockies Forest	l	AY165319	BF	AY257749		
Arab0818	*Boechera stricta*	Colorado Rockies Forest	d	AY165315	BB	AY257745		
Arab0819	*Boechera stricta*	Cascade Mountains Leeward Forests	az	AY165374	AH	AY257725		
Arab0822	*Boechera stricta*	North Central Rockies Forest	az	AY165374	AH	AY257725		
Arab0823	*Boechera stricta*	North Central Rockies Forest	az	AY165374	AH	AY257725		
Arab0835	*Boechera cobrensis*	Snake/Columbia Shrub Steppe	ad	AY165328	ci	AY257778		
Arab0836	*Boechera cobrensis*	Great Basin Shrub Steppe West	ad	AY165328	ci	AY257778		
Arab0837	*Boechera cobrensis*	Great Basin Shrub Steppe central West	ad	AY165328	ci	AY257778		
Arab0838	*Boechera cobrensis*	Great Basin Shrub Steppe West	hk	EU274966	ci	AY257778		
Arab0839	*Boechera cobrensis*	Snake/Columbia Shrub Steppe	gy	EU274873	ci	AY257778		
Arab0841	*Boechera cobrensis*	Great Basin Shrub Steppe West	hl	EU274967				
Arab0842	*Boechera cobrensis*	Snake/Columbia Shrub Steppe	hm	EU274968	ig	EU154158, EU154296		
Arab0843	*Boechera cobrensis*	Snake/Columbia Shrub Steppe	ad	AY165328	ig	EU154158, EU154296		
Arab0845	*Boechera cobrensis*	Snake/Columbia Shrub Steppe	ad	AY165328	ci	AY257778		
Arab0849	*Boechera crandallii*	Colorado Plateau Shrub Steppe East	f	AY165346	ci	AY257778		
Arab0850	*Boechera crandallii*	Colorado Plateau Shrub Steppe East	hn	EU274969	ci	AY257778		

Accession	Country	State/ Province	County	Herbarium	Herbarium number	Collector	Coll. number	X coordi-nate	Y coordi-nate
Arab0798	USA	Nevada	Elko	GH		R.C. Rollins	2545, T.S. Chambers	-115.38	40.58
Arab0802	USA	Nevada	Elko	GH		A. Pinzl	4520	-115.47	41.80
Arab0803	USA	Nevada	Eureka						
Arab0804	USA	Nevada	Elko						
Arab0806	USA	Nevada	Douglas						
Arab0807	USA	Nevada	Elko						
Arab0812	USA	Colorado	Hinsdale	GH		R.C. Rollins	1506	-107.38	37.78
Arab0813	USA	Colorado	Saguache	GH		D. Neill, C. Hamilton	1779	-106.46	38.41
Arab0814	USA	Colorado	Saguache	GH		R.C. & K.W. Rollins	86226	-106.25	38.40
Arab0815	USA	Colorado	Pitkin	GH		C. Leostitch##	3963	-106.75	39.13
Arab0817	USA	Colorado	Clear Creek	GH		U.T. Waterfall	3423	-105.43	39.65
Arab0818	USA	Colorado	Rio Garnde	GH		R.C. Rollins	1484	-106.60	37.45
Arab0819	Canada	British Columbia		GH		J.A. Calder, D.B.O. Savile	11647	-120.65	49.13
Arab0822	Canada	Alberta		GH		A.J. Breitung	16765	-113.98	49.03
Arab0823	Canada	Alberta		GH		R. Spellenberg, R. Soreng		-114.53	50.26
Arab0835	USA	Nevada	Humboldt						
Arab0836	USA	Nevada	Mineral						
Arab0837	USA	Nevada	Nye						
Arab0838	USA	Nevada	Washoe						
Arab0839	USA	Oregon	Harney						
Arab0841	USA	Nevada	Lyon	GH		R.C. & K.W. Rollins	81143	-119.21	38.55
Arab0842	USA	Nevada	Washoe	GH		R.C. & K.W. Rollins	81222	-119.60	41.53
Arab0843	USA	Nevada	Humboldt	GH		R.C. & K.W. Rollins	81227		
Arab0845	USA	Nevada	Humboldt	GH		A. Pinzl	8362, T. Knight, D. Danley	-118.63	41.97
Arab0849	USA	Colorado	Gunnison						
Arab0850	USA	Colorado	Gunnison						

Accession	Taxon	wwf_ecoregion	ITS-type	ITS accession number	trnL/F type	trnLF accession numbers	*At2g25920 orthologue*	*At3g18900 orthologue*
Arab0855	*Bochera davidsonii*	Great Basin Shrub Steppe West	oq	EU274870	b	AY257693		
Arab0861	*Boechera fendleri*	Chihuahua Desert	ho	EU274970	cj	AY257779		
Arab0864	*Boechera fendleri*	Chihuahua Desert	hp	EU274971	ii	EU154159, EU154297		
Arab0868	*Boechera spatifolia*	Colorado Rockies Forest	hq	EU274972				
Arab0869	*Boechera spatifolia*	Colorado Rockies Forest	u	AY165324	il	EU154161, EU154299		
Arab0876	*Boechera fendleri*	Mojave Desert	hr	EU274973	ci	AY257778		
Arab0877	*Boechera fernaldiana var. fernaldiana*	Great Basin Shrub Steppe West	hs	EU274974	iq	EU154165, EU154303		
Arab0880	*Boechera fernaldiana var. fernaldiana*	Great Basin Shrub Steppe West	bt	AY165331	iq	EU154165, EU154303		
Arab0881	*Boechera fernaldiana var. fernaldiana*	Great Basin Shrub Steppe central West	op	EU274869	cj	AY257779		
Arab0885	*Boechera fernaldiana*	Great Basin Shrub Steppe central West	op	EU274869	iu	EU154168, EU154306		
Arab0886	*Boechera fernaldiana var. fernaldiana*	Great Basin Shrub Steppe central West	hu	EU274975	ci	AY257778		
Arab0887	*Boechera fernaldiana*	Great Basin Shrub Steppe West	hv	EU274976	ci	AY257778		
Arab0890	*Boechera fernaldiana var. fernaldiana*	Great Basin Shrub Steppe West	hw	EU274977	ci	AY257778		
Arab0892	*Boechera fernaldiana var. fernaldiana*	Great Basin Shrub Steppe central West	op	EU274869	hv	EU154169, EU154307		
Arab0895	*Boechera gunnisoniana*	Colorado Plateau Shrub Steppe East	os	EU274978	ci	AY257778		
Arab0898	*Boechera gunnisoniana*	Colorado Plateau Shrub Steppe East	hx	EU274891	ds	DQ013060		
Arab0903	*Boechera inyoensis*	Great Basin Shrub Steppe central West	hy	EU274979	in	EU154171, EU154309		
Arab0904	*Boechera koehleri*	Klamath Siskiyou Forests	hz	EU274980	o	AY257706		
Arab0909	*Boechera lasiocarpa*	Wasatch/Uinta Montane Forest	oh	EU274892	d	AY257695		
Arab0910	*Boechera lasiocarpa*	Wasatch/Uinta Montane Forest	oh	EU274892	d	AY257695		
Arab0915	*Boechera lemmonii*	Great Basin Shrub Steppe West	er	AY165339				

Accession	Country	State/ Province	County	Herbarium	Herbarium number	Collector	Coll. number	X coordi-nate	Y coordi-nate
Arab0855	USA	Nevada	Humboldt						
Arab0861	USA	New Mexico	Dona Ana						
Arab0864	USA	Texas	Brewster	GH		R.C. Rollins	6172, D.S. Correll	-103.20	29.83
Arab0868	USA	Colorado	Chaffee	GH		R.C. Rollins	8356, K.W. Rollins, A.G. Roads	-106.10	38.81
Arab0869	USA	Colorado	Saguache	GH		R.C. Rollins	8354, K.W. Rollins, A.G. Roads	-106.03	38.36
Arab0876	USA	Nevada	Nye	GH		J.Beatley, J.L. Reveal		-115.95	36.46
Arab0877	USA	Nevada	Humboldt						
Arab0880	USA	California	Mono	GH		R.S. Mitchell	2087	-118.30	37.79
Arab0881	USA	Nevada	Humboldt						
Arab0885	USA	Nevada	Nye						
Arab0886	USA	Nevada	Lander						
Arab0887	USA	Nevada	Mineral						
Arab0890	USA	Nevada	Douglas						
Arab0892	USA	Nevada	Nye						
Arab0895	USA	Colorado	Gunnison						
Arab0898	USA	Colorado	Gunnison	GH		R.C. & K.W. Rollins	7960, K.W. Rollins	-106.80	38.51
Arab0903	USA	Nevada	Nye						
Arab0904	USA	Oregon	Douglas	GH		J. & O. Fosback		-123.33	43.21
Arab0909	USA	Utah	Salt Lake						
Arab0910	USA	Utah	Cache						
Arab0915	USA	California	Mono	GH		V. Duran	2611	-118.25	37.63

Accession	Taxon	wwf_ecoregion	ITS-type	ITS accession number	trnL/F type	trnLF accession numbers	*At2g25920 orthologue*	*At3g18900 orthologue*
Arab0918	*Boechera lemmonii*	South Central Rockies Forest West	er	AY165339	m	AY257704		
Arab0923	*Boechera lemmonii*	Great Basin Shrub Steppe central West	er	AY165339	ci	AY257778		
Arab0927	*Boechera lignifera*	Colorado Plateau Shrub Steppe	ia	EU274981	y	AY257716		
Arab0928	*Boechera lignifera*	Great Basin Shrub Steppe central East	ad	AY165328	ci	AY257778		
Arab0930	*Boechera lignifera*	Colorado Plateau Shrub Steppe	er	AY165339	u	AY257712		
Arab0931	*Boechera lignifera*	Great Basin Shrub Steppe East	h	AY165348	by	AY257768		
Arab0933	*Boechera lignifera*	Great Basin Shrub Steppe West	ib	EU274982				
Arab0934	*Boechera lignifera*	Great Basin Shrub Steppe East	ic	EU274983	ab	AY257719	GQ166236	
Arab0935	*Boechera lignifera*	Great Basin Shrub Steppe East					GQ166237	
Arab0939	*Boechera lignifera*	Wyoming Basin Shrub Steppe	id	EU274984	u	AY257712		
Arab0942	*Boechera lignifera*	Great Basin Shrub Steppe East	ie	EU274985				
Arab0945	*Boechera lignifera*	Wasatch/Uinta Montane Forest	ac	AY165327				
Arab0946	*Boechera "pulchra"*	Great Basin Shrub Steppe West	eu	AY165340	s	AY257710		
Arab0953	*Boechera pauciflora*	Great Basin Shrub Steppe central East	h	AY165348	u	AY257712		
Arab0955	*Boechera pauciflora*	Eastern Cascades Forest	if	EU274986	L	AY257703		
Arab0959	*Boechera pauciflora*	Eastern Cascades Forest	ig	EU274987	ix	EU154176, EU154314		
Arab0960	*Boechera pauciflora*	Eastern Cascades Forest	ih	EU274988	L	AY257703		
Arab0965	*Boechera sparsiflora*	Great Basin Shrub Steppe West	gx	EU274871	ci	AY257778		
Arab0967	*Boechera sparsiflora*	Snake/Columbia Shrub Steppe	ii	EU274989	b	AY257693		
Arab0968	*Boechera "sparsiflora"*	Snake/Columbia Shrub Steppe	c	AY165314				
Arab0971	*Boechera pauciflora*	Snake/Columbia Shrub Steppe	h	AY165348	ci	AY257778		
Arab0972	*Boechera pauciflora*	Great Basin Shrub Steppe West	ar	AY165369	ci	AY257778		
Arab0977	*Boechera sparsiflora*	Great Basin Shrub Steppe West	gx	EU274871	ci	AY257778		
Arab0980	*Boechera sparsiflora*	Snake/Columbia Shrub Steppe	gx	EU274871	iz	EU154178, EU154316		
Arab0981	*Boechera sparsiflora*	Great Basin Shrub Steppe central West	h	AY165348	ci	AY257778		
Arab0982	*Boechera sparsiflora*	Snake/Columbia Shrub Steppe	gx	EU274871				
Arab0985	*Boechera pauciflora*	Great Basin Shrub Steppe West	ar	AY165369				

Accession	Country	State/ Province	County	Herbarium	Herbarium number	Collector	Coll. number	X coordi-nate	Y coordi-nate
Arab0918	USA	Idaho	Lemhi	GH		R.C. & K.W. Rollins	86175	-113.31	44.43
Arab0923	USA	Nevada	Nye						
Arab0927	USA	Colorado	Rio Blanco						
Arab0928	USA	Nevada	Lincoln						
Arab0930	USA	Colorado	Gunnison	GH		R.C. & K.W. Rollins	7972, K.W. Rollins	-106.61	38.45
Arab0931	USA	Idaho	Power	GH		R.C. & K.W. Rollins	81306	-112.90	42.27
Arab0933	USA	Nevada	Nye						
Arab0934	USA	Nevada	White Pine						
Arab0935	USA	Utah	Millard	GH		S. Goodrich	18696	-113.76	38.76
Arab0939	USA	Wyoming	Sweetwater	GH		R.C. & K.W. Rollins	81354		
Arab0942	USA	Utah	Box Elder	GH		R.C. & K.W. Rollins	81285	-113.75	41.81
Arab0945	USA	Utah	Uintah	GH		R.C. & K.W. Rollins	79138	-109.58	40.71
Arab0946	USA	Nevada	Humboldt						
Arab0953	USA	Nevada	Elko						
Arab0955	USA	California	Modoc						
Arab0959	USA	California	Modoc						
Arab0960	USA	California	Modoc						
Arab0965	USA	Nevada	Lyon						
Arab0967	USA	Idaho	Gem						
Arab0968	USA	Idaho	Cassia	GH		R.C. & K.W. Rollins	81280	-113.61	42.31
Arab0971	USA	California	Lassen	GH		R.C. & K.W. Rollins	81178	-120.71	40.73
Arab0972	USA	California	Mono	GH		R.C. & K.W. Rollins	81139	-119.18	38.36
Arab0977	USA	California	Mono	GH		R.C. & K.W. Rollins	81141	-119.18	38.41
Arab0980	USA	Nevada	Washoe	GH		R.C. & K.W. Rollins	81221	-119.60	41.53
Arab0981	USA	Nevada	Humboldt	GH		R.C. & K.W. Rollins	81238	-117.55	41.66
Arab0982	USA	Nevada	Humboldt	GH		R.C. & K.W. Rollins	81225	-119.28	41.80
Arab0985	USA	California	Mono	GH		R.C. & K.W. Rollins	81142	-119.18	38.41

Accession	Taxon	wwf_ecoregion	ITS-type	ITS accession number	trnL/F type	trnLF accession numbers	*At2g25920 orthologue*	*At3g18900 orthologue*
Arab0986	*Boechera pauciflora*	Eastern Cascades Forest	hf	EU274990	kb	EU154180, EU154318		
Arab0988	*Boechera californica*	Sierra Nevada South	ev	AY165341	ci	AY257778		
Arab0989	*Boechera californica*	California Coastal Sage and Chaparral	og	EU274884	bu	AY257764		
Arab0992	*Boechera sparsiflora*	Eastern Cascades Forest	gx	EU274871				
Arab0999	*Boechera sparsiflora*	Snake/Columbia Shrub Steppe	ou	EU274991	ke	EU154182, EU154320		
Arab1006	*Boechera pauciflora*	Great Basin Shrub Steppe West	h	AY165348				
Arab1009	*Boechera "microphylla"*	Great Basin Shrub Steppe West	h	AY165348	fo	EU154191, EU154329		
Arab1011	*Boechera "microphylla"*	Great Basin Shrub Steppe central East	ik	EU274894	fo	EU154191, EU154329		
Arab1013	*Boechera "microphylla"*	Great Basin Shrub Steppe East	h	AY165348	m	AY257704		
Arab1015	*Boechera "microphylla"*	Snake/Columbia Shrub Steppe	il	EU274866	fo	EU154191, EU154329		
Arab1016	*Boechera "microphylla"*	Great Basin Shrub Steppe West	h	AY165348	fo	EU154191, EU154329		
Arab1019	*Boechera "microphylla"*	Snake/Columbia Shrub Steppe	h	AY165348	fo	EU154191, EU154329		
Arab1020	*Boechera "microphylla"*	Great Basin Shrub Steppe central West	im	EU274895	ci	AY257778		
Arab1023	*Boechera "microphylla"*	South Central Rockies Forest West	in	EU274896	ci	AY257778		
Arab1025	*Boechera "microphylla"*	South Central Rockies Forest West	h	AY165348	b	AY257693		
Arab1027	*Boechera "microphylla"*	Great Basin Shrub Steppe East	h	AY165348	b	AY257693		
Arab1028	*Boechera "microphylla"*	Wasatch/Uinta Montane Forest	h	AY165348	fo	EU154191, EU154329		
Arab1030	*Boechera "microphylla"*	Wasatch/Uinta Montane Forest	h	AY165348	fo	EU154191, EU154329		
Arab1031	*Boechera "microphylla"*	South Central Rockies Forest East	h	AY165348				
Arab1035	*Boechera pallidifolia*	Colorado Plateau Shrub Steppe East	io	EU274897	cl	AY257781		
Arab1039	*Boechera pendulina*	Colorado Plateau Shrub Steppe	g	AY165347	c	AY257694		

Accession	Country	State/ Province	County	Herbarium	Herbarium number	Collector	Coll. number	X coordi-nate	Y coordi-nate
Arab0986	USA	California	Modoc	GH		R.C. & K.W. Rollins	81193	-120.28	41.65
Arab0988	USA	California	Tulare						
Arab0989	USA	California	Riverside	GH		R.L. Dressler	824	-116.71	33.66
Arab0992	USA	California	Modoc						
Arab0999	USA	Nevada	Washoe						
Arab1006	USA	Nevada	Mineral						
Arab1009	USA	Nevada	Humboldt						
Arab1011	USA	Nevada	Elko						
Arab1013	USA	Nevada	Elko						
Arab1015	USA	Nevada	Washoe						
Arab1016	USA	Nevada	Pershing						
Arab1019	USA	Nevada	Humboldt						
Arab1020	USA	Nevada	Humboldt	GH		R.C. & K.W. Rollins	81230	-117.53	41.66
Arab1023	USA	Idaho	Valley						
Arab1025	USA	Montana	Ravalli	GH		R.C. & K.W. Rollins	83299, A.G. Roads	-113.96	45.81
Arab1027	USA	Utah	Box Elder						
Arab1028	USA	Utah	Millard						
Arab1030	USA	Utah	Salt Lake						
Arab1031	USA	Wyoming	Fremont	GH		R.C. Rollins	79336, M.L. Rollins	-108.85	42.43
Arab1035	USA	Colorado	Gunnison	GH		R.C. & K.W. Rollins	7970, K.W. Rollins	-106.61	38.45
Arab1039	USA	Colorado	Rio Blanco						

Accession	Taxon	wwf_ecoregion	ITS-type	ITS accession number	trnL/F type	trnLF accession numbers	*At2g25920 orthologue*	*At3g18900 orthologue*
Arab1040	*Boechera pendulina*	Great Basin Shrub Steppe central East	ip	EU274869	en	EU154085, EU154223		
Arab1041	*Boechera pendulina*	Great Basin Shrub Steppe central East	g	AY165347	en	EU154085, EU154223		
Arab1042	*Boechera pendulina*	Great Basin Shrub Steppe central East	g	AY165347	ho	EU154142, EU154280		
Arab1045	*Boechera pendulina*	Colorado Plateau Shrub Steppe	g	AY165347	hp	EU154143, EU154281		
Arab1056	*Boechera perennans*	Wasatch/Uinta Montane Forest	f	AY165346				
Arab1060	*Boechera perennans*	Great Basin Shrub Steppe central East	ev	AY165341	ci	AY257778		
Arab1061	*Boechera perennans*	Mojave Desert	ev	AY165341	ci	AY257778		
Arab1062	*Boechera perennans*	Mojave Desert	ev	AY165341	ci	AY257778		
Arab1064	*Boechera perennans*	Great Basin Shrub Steppe East	pb	EU274899	ab	AY257719		
Arab1067	*Boechera perennans*	Mojave Desert	ev	AY165341	ci	AY257778		
Arab1068	*Boechera perennans*	Great Basin Shrub Steppe central East	ir	EU274900	ci	AY257778		
Arab1070	*Boechera cf. gracilipes*	Mojave Desert	is	EU274867	hr	EU154145, EU154283		
Arab1072	*Boechera puberula*	Klamath Siskiyou Forests	ol	EU274901	s	AY257710		
Arab1076	*Boechera puberula*	Eastern Cascades Forest	eu	AY165340	s	AY257710		
Arab1078	*Boechera puberula*	Eastern Cascades Forest	it	EU274902	s	AY257710		
Arab1080	*Boechera puberula*	Great Basin Shrub Steppe West	eu	AY165340	s	AY257710		
Arab1081	*Boechera puberula*	Great Basin Shrub Steppe central West	eu	AY165340	s	AY257710		
Arab1084	*Boechera puberula*	Snake/Columbia Shrub Steppe	eu	AY165340	s	AY257710		
Arab1088	*Boechera puberula*	Snake/Columbia Shrub Steppe	eu	AY165340	h	AY257699		
Arab1089	*Boechera puberula*	Great Basin Shrub Steppe central West	om	EU274903	o	AY257706		
Arab1091	*Boechera puberula*	Snake/Columbia Shrub Steppe	ac	AY165327	s	AY257710		
Arab1092	*Boechera puberula*	Great Basin Shrub Steppe West	eu	AY165340	s	AY257710		
Arab1095	*Boechera puberula*	Great Basin Shrub Steppe central West	on	EU274904	s	AY257710		
Arab1096	*Boechera puberula*	Great Basin Shrub Steppe West	au	AY165370	cj	AY257779		
Arab1097	*Boechera puberula*	Great Basin Shrub Steppe West	eu	AY165340	s	AY257710		

Accession	Country	State/ Province	County	Herbarium	Herbarium number	Collector	Coll. number	X coordi-nate	Y coordi-nate
Arab1040	USA	Nevada	White Pine						
Arab1041	USA	Nevada	White Pine						
Arab1042	USA	Nevada	White Pine						
Arab1045	USA	Utah	Duchesne						
Arab1056	USA	Utah	Iron	GH		R.C. & K.W. Rollins	81112	-113.40	37.56
Arab1060	USA	Nevada	Lincoln	GH		A. Pinzl	7003	-114.30	37.03
Arab1061	USA	Nevada	Clark	GH		A. Pinzl	6888	-114.11	36.28
Arab1062	USA	Nevada	Nye	GH		J. Beatley, J.L. Reveal		-117.06	37.54
Arab1064	USA	Utah	Washington	GH		L.C. Higgins	1229		
Arab1067	USA	Nevada	Lincoln						
Arab1068	USA	Nevada	Lincoln						
Arab1070	USA	Nevada	Clark	GH		J.Beatley, J.L. Reveal		-115.81	36.33
Arab1072	USA	Nevada		GH		R.C. & K.W. Rollins	83221 et al.	-123.65	42.58
Arab1076	USA	California	Modoc	GH		R.C. & K.W. Rollins	81195	-120.28	42.00
Arab1078	USA	California	Lassen	GH		R.C. & K.W. Rollins	81183	-120.95	40.96
Arab1080	USA	Nevada	Lyon	GH		R.C. & K.W. Rollins	81146	-119.21	38.58
Arab1081	USA	Nevada	Eureka	GH		R.C. & K.W. Rollins	81242	-116.26	40.65
Arab1084	USA	Nevada	Washoe	GH		R.C. & K.W. Rollins	81219	-119.90	41.53
Arab1088	USA	Idaho	Gooding						
Arab1089	USA	Nevada	Nye						
Arab1091	USA	Nevada	Humboldt	GH		A. Pinzl	3951	-119.23	41.31
Arab1092	USA	Nevada	Lyon						
Arab1095	USA	Nevada	Elko						
Arab1096	USA	Nevada	Douglas						
Arab1097	USA	Nevada	Humboldt	GH		A. Pinzl	8965	-117.55	41.58

Accession	Taxon	wwf_ecoregion	ITS-type	ITS accession number	trnL/F type	trnLF accession numbers	*At2g25920 orthologue*	*At3g18900 orthologue*
Arab1098	*Boechera puberula*	Great Basin Shrub Steppe West	au	AY165370	y	AY257716		
Arab1100	*Boechera puberula*	Great Basin Shrub Steppe central East	au	AY165370	s	AY257710		
Arab1101	*Boechera puberula*	Snake/Columbia Shrub Steppe	au	AY165370	s	AY257710		
Arab1103	*Boechera puberula*	Great Basin Shrub Steppe East	au	AY165370	hu	EU154148, EU154286		
Arab1104	*Boechera puberula*	Great Basin Shrub Steppe West	iu	EU274905	o	AY257706		
Arab1107	*Boechera puberula*	Great Basin Shrub Steppe central West	au	AY165370	s	AY257710		
Arab1109	*Boechera puberula*	Great Basin Shrub Steppe West	pa	EU274906	m	AY257704		
Arab1112	*Boechera tiehmii*	Sierra Nevada South	er	AY165339	hw	EU154149, EU154287		
Arab1113	*Boechera williamsii*	South Central Rockies Forest West	iv	EU274907	as	AY257736		
Arab1114	*Boechera williamsii*	South Central Rockies Forest East	ao	AY165365	hx	EU154150, EU154288		
Arab1300	*Boechera stricta*	Sierra Nevada			AH	AY257725	GQ166238	
Arab1315	*Boechera missouriensis*		sg	EU274863				HE589955
Arab1317	*Boechera macounii*	Eastern Canadian Forests	iw	EU274908			GQ166326	HE589956
Arab1318	*Boechera lignifera*	Great Basin Shrub Steppe central East	iw	EU274908				
Arab1319	*Boechera lignifera*	Sierra Nevada North	i	AY165317				
Arab1320	*Boechera lignifera*	Okanogan Forest	h	AY165348	dt	EU154066, EU154204		
Arab1321	*Boechera lignifera*	Okanogan Forest	ix	EU274909	bu	AY257764		
Arab1322	*Boechera lyallii*	Cascade Mountains Leeward Forests	iy	EU274868	dv	EU154068, EU154206		
Arab1325	*Boechera lyallii*	Okanogan Forest	iz	EU274910				
Arab1327	*Boechera lyallii*						GQ166239	
Arab1330	*Boechera lyallii*							HE589957

Accession	Country	State/ Province	County	Herbarium	Herbarium number	Collector	Coll. number	X coordi-nate	Y coordi-nate
Arab1098	USA	Nevada	Humboldt						
Arab1100	USA	Nevada	White Pine						
Arab1101	USA	Nevada	Humboldt						
Arab1103	USA	Nevada	Elko						
Arab1104	USA	Nevada	Churchill						
Arab1107	USA	Nevada	Nye						
Arab1109	USA	Nevada	Churchill						
Arab1112	USA	California	Mono						
Arab1113	USA	Idaho	Custer	GH		R.C. & K.W. Rollins	83255, A.G. Roads	-113.93	44.36
Arab1114	USA	Wyoming	Fremont						
Arab1300	USA	California	Inyo	CAS		M. DeDecker	3289	-118.34	36.77
Arab1315	USA	Massachusetts	Hampden	DAO	792294	Tad Zebryk	6688	-72.62	42.28
Arab1317	Canada	Québec		DAO	784288	N. Dignard, P. Petitclerc et J.-M. Hardy	Jan 37	-64.23	48.53
Arab1318	USA	Nevada	White Pine	DAO	543491	Noel H. Holmgren and James Reveal	929	-114.20	39.21
Arab1319	USA	California	Lassen	DAO	599199	T. Mosquin und P. H. Raven	4328	-120.43	40.21
Arab1320	Canada	British Columbia		DAO		J. A. Calder und D. B. O. Savile	10366	-120.33	50.66
Arab1321	Canada	Columbia		DAO	563888	J.A. Calder und D. B. O. Savile	10945		
Arab1322	Canada	British Columbia		DAO	135261	R. Hainault	1535	-120.18	49.03
Arab1325	Canada	British Columbia		DAO	564513	J. A. Calder und D. B. O. Savile	11686	-119.58	49.50
Arab1327	Canada	British Columbia		DAO	564527	M. Bell und J. Davidson	544	-114.61	49.35
Arab1330	Canada	Alberta		DAO	564510	J. A. Calder und K. T. Mac Kay	32722B	-116.38	51.66

Accession	Taxon	wwf_ecoregion	ITS-type	ITS accession number	trnL/F type	trnLF accession numbers	*At2g25920 orthologue*	*At3g18900 orthologue*
Arab1331	*Boechera calderi*	Alberta Mountain Forest	ab	AY165326				HE589958
Arab1345	*Boechera "microphylla"*	Snake/Columbia Shrub Steppe	h	AY165348	dw	EU154069, EU154207	GQ166240	
Arab1346	*Boechera microphylla*	Central and Southern Cascades Forest	ka	EU274911	dx	EU154070, EU154208	GQ166241	HE589959
Arab1349	*Boechera microphylla*	Great Basin Shrub Steppe West	h	AY165348				
Arab1350	*Boechera microphylla*	Blue Mountain Forest	h	AY165348				
Arab1351	*Boechera microphylla*	Great Basin Shrub Steppe central West	op	EU274869	dy	EU154071, EU154209	GQ166327	
Arab1353	*Boechera microphylla*	North Central Rockies Forest	kb	EU274912	dz	EU154072, EU154210		
Arab1358	*Bochera davidsonii*	Great Basin Shrub Steppe central West	oq	EU274870	KC	EU154186, EU154324		
Arab1360	*Boechera fendleri*	Colorado Plateau Shrub Steppe	kd	EU274913	eb	EU154074, EU154212		
Arab1363	*Boechera fendleri*	Mojave Desert	is	EU274867				
Arab1364	*Boechera fendleri*	Mojave Desert	ke	EU274914	ec	EU154075, EU154213		
Arab1367	*Boechera koehleri*	Klamath Siskiyou Forests					GQ166328	
Arab1369	*Boechera laevigata*	Eastern Great Lakes Forest	or	EU274839	ef	EU154078, EU154220		
Arab1371	*Boechera laevigata*	Eastern Great Lakes Forest	kh	EU274843	ef	EU154078, EU154217		
Arab1372	*Boechera laevigata*		ki	EU274844	ef	EU154078, EU154218		
Arab1374	*Boechera laevigata*	Eastern Great Lakes Forest	or	EU274839	ef	EU154078, EU154221		
Arab1376	*Boechera laevigata*		kk	EU274845	ef	EU154078, EU154219		
Arab1377	*Boechera laevigata*		or	EU274839	ef	EU154078, EU154222	GQ166329	
Arab1378	*Boechera lemmonii*	Sierra Nevada South	er	AY165339			GQ166330	
Arab1379	*Boechera lemmonii*	Sierra Nevada North	gx	EU274871	ci	AY257778	GQ166242	

Accession	Country	State/ Province	County	Herbarium	Herbarium number	Collector	Coll. number	X coordi- nate	Y coordi- nate
Arab1331	Canada	Alberta		DAO	564507	K. T. Mac Kay und J. A. Calder	32671	-117.08	52.18
Arab1345	USA	Oregon	Harney	DAO	564736	A. Cronquist	8258	-118.56	42.58
Arab1346	USA	Washington	Skamania	DAO	564735	J. A. Calder, D. B. O. Savile, R. L. Taylor		-122.01	45.63
Arab1349	USA	Nevada	Humboldt	DAO	575984	A. Tiehm	11100	-117.68	41.51
Arab1350	USA	Oregon	Baker	DAO	599422	J. M. Gillett und R. L. Taylor		-118.15	44.40
Arab1351	USA	Nevada	Lander	DAO	540938	A. Tiehm	10456	-116.86	40.45
Arab1353	Canada	British Columbia		DAO		J. A. Calder und D. B. O. Savile	7686	-115.86	49.41
Arab1358	USA	Nevada	Humbold	DAO	579108	A. Tiehm	9729	-117.63	41.45
Arab1360	USA	Utah	Garfield	DAO	391082	A. Cronquist and N. Holmgren	9292	-110.71	38.15
Arab1363	USA	Nevada	Clark	DAO	391077	I. W. Clokes	8369	-115.63	36.30
Arab1364	USA	Nevada	Clark	DAO	391309	I. W. Clokey	7538	-115.58	36.25
Arab1367	USA	Oregon	Douglas	DAO	565059	J. W. Thomson	10157	-123.33	43.21
Arab1369	Canada	Ontario	Leeds	DAO	128040	W. J. Cody and D. Munro	21773	-76.19	44.29
Arab1371	Canada	Ontario	Hastings	DAO	795203	W. G. Dore	20307	-77.35	44.16
Arab1372	Canada	Ontario	Stormont	DAO	795204	G. N. Gogo	335	0.00	0.00
Arab1374	Canada	Ontario	Leeds	DAO	129698	W. J. Cody and D. Munro	21701	-79.19	44.29
Arab1376	Canada	Quebec	Huntison	DAO	564454	G. N. Gogo	334	0.00	0.00
Arab1377	Canada	Quebec		DAO	564450	L. Cinq-Mars et J. Bonneau	63-495	0.00	0.00
Arab1378	USA	California	Tulare	DAO	564495	R. S. Ferris and L. Lorraine	11196	-118.56	36.40
Arab1379	USA	California	Tehama	DAO	564492	G. W. Gillett	262	-121.51	40.43

Accession	Taxon	wwf_ecoregion	ITS-type	ITS accession number	trnL/F type	trnLF accession numbers	*At2g25920 orthologue*	*At3g18900 orthologue*
Arab1380	*Boechera lemmonii*	Cascade Mountains Leeward Forests	er	AY165339	ci	AY257778	GQ166243	
Arab1381	*Boechera lemmonii*	North Central Rockies Forest	er	AY165339	eh	EU154080, EU154218	GQ166244	
Arab1382	*Boechera lemmonii*	Cascade Mountains Leeward Forests	kl	EU274915	eh	EU154080, EU154218		
Arab1384	*Boechera lemmonii*	North Central Rockies Forest	km	EU274916	eh	EU154080, EU154218		
Arab1385	*Boechera lemmonii*	North Central Rockies Forest	er	AY165339	m	AY257704		
Arab1407	*Boechera breweri*	Klamath Siskiyou Forests	kn	EU274872	ci	AY257778	GQ166245	HE589960
Arab1409	*Boechera canadensis*		ko	EU274840	ei	EU154081, EU154219	GQ166246	
Arab1410	*Boechera canadensis*		sc	EU274860	ek	EU154082, EU154220	GQ166247	HE589961
Arab1411	*Boechera canadensis*		kp	EU274841	ei	EU154081, EU154219	GQ166248	
Arab1413	*Boechera canadensis*		sd	EU274861	ei	EU154081, EU154219	GQ166249	HE589962
Arab1416	*Boechera canadensis*		sc	EU274860	ei	EU154081, EU154219		
Arab1417	*Boechera canadensis*		sc	EU274860	ei	EU154081, EU154219	GQ166250	HE589963
Arab1418	*Boechera canadensis*		sc	EU274860	ei	EU154081, EU154219		
Arab1419	*Boechera cobrensis*	Great Basin Shrub Steppe central West	ad	AY165328	ci	AY257778	GQ166251	
Arab1420	*Boechera cobrensis*	Great Basin Shrub Steppe central East					GQ166331	
Arab1421	*Boechera cobrensis*	Snake/Columbia Shrub Steppe	gy	EU274873			GQ166252	
Arab1422	*Boechera cobrensis*	Snake/Columbia Shrub Steppe	kr	EU274917	ci	AY257778	GQ166253	
Arab1423	*Boechera cobrensis*	Wyoming Basin Shrub Steppe	ks	EU274918	ci	AY257778	GQ166254	

Accession	Country	State/ Province	County	Herbarium	Herbarium number	Collector	Coll. number	X coordi-nate	Y coordi-nate
Arab1380	Canada	British Columbia		DAO	795197	J. A. Calder, J. A. Parmelee and R. L. Taylor	19633A	-120.21	49.06
Arab1381	Canada	Columbia		DAO	795198	J. A. Calder, D. B. O. Savile	11251	-115.96	50.46
Arab1382	Canada	British Columbia		DAO	795199	J. A. Calder, D. B. O. Savile and J. M. Ferguson	15561	-121.93	50.68
Arab1384	Canada	British Columbia		DAO	795201	R. L. Taylor, D. H. Ferguson	2779A	-115.60	49.71
Arab1385	Canada	British Columbia		DAO	700941	G. W. Duglas, J. L. Penny and D. Smith	13142	-115.68	50.65
Arab1407	USA	California	Shasta	DAO	563612	J. Langenheim	4337	-122.30	41.11
Arab1409	USA	Iowa	Decatur	DAO	563625	T. van Bruggen		-93.75	40.60
Arab1410	USA	Iowa	Fermont	DAO	563623	M. J. Fay		-95.68	40.68
Arab1411	Canada	Ontario	Wentworth	DAO	795206	A. Tamsalu		-63.55	45.63
Arab1413	Canada	Ontario	Elgin	DAO	795208	L. E. James		-81.20	42.78
Arab1416	Canada	Quebec	Gatineau	DAO	623011	W. G. Dore, R. J. Moore	14452	-75.93	45.58
Arab1417	Canada	Quebec		DAO	563618	B. Bovin, L. Jenkins and L. J. van Rens	14296	-76.10	45.55
Arab1418	Canada	Quebec		DAO	563617	B. Bovin et L. J. van Rens	14263	-75.70	45.46
Arab1419	USA	Nevada	Eureka	DAO	394484	A. Tiehm, M. Williams	7898	-116.40	39.56
Arab1420	USA	Nevada	Elko	DAO	579350	A. Tiehm, M. Williams	9630	-115.23	41.61
Arab1421	USA	Oregon	Harney	DAO	563653	C. L. Hitchcock, C. V. Muhlick	21169	-118.65	42.36
Arab1422	USA	Nevada	Humboldt	DAO	244742	J. L. Gentry jr., G. Davidse	1546	-117.38	41.86
Arab1423	USA	Wyoming	Uinta	DAO	563651	R. C. Rollins	1656	-110.30	41.36

Accession	Taxon	wwf_ecoregion	ITS-type	ITS accession number	trnL/F type	trnLF accession numbers	*At2g25920 orthologue*	*At3g18900 orthologue*
Arab1435	*Boechera pendulina*	Colorado Plateau Shrub Steppe West	kt	EU274874				
Arab1436	*Boechera pendulina*	Colorado Plateau Shrub Steppe	ev	AY165341	el	EU154083, EU154221	GQ166255	HE589964
Arab1437	*Boechera pendulina*	Colorado Plateau Shrub Steppe West	ev	AY165341			GQ166332	
Arab1440	*Boechera pendulina*	Great Basin Shrub Steppe central East	g	AY165347	em	EU154084, EU154222	GQ166256	
Arab1441	*Boechera pendulina*	Great Basin Shrub Steppe central East	g	AY165347	en	EU154085, EU154223	GQ166257	
Arab1442	*Boechera pendulina*	Wasatch/Uinta Montane Forest	ev	AY165341	bk	AY257754		HE589965
Arab1443b	*Boechera perennans*	Great Basin Shrub Steppe central East	f	AY165346				
Arab1444	*Boechera perennans*	Mojave Desert	ev	AY165341	ci	AY257778	GQ166258	
Arab1445	*Boechera perennans*	Colorado Plateau Shrub Steppe West	ku	EU274919	eq	EU154088, EU154226	GQ166259	
Arab1446	*Boechera perennans*							HE589966
Arab1446	*Boechera perennans*						GQ166260	
Arab1447	*Boechera platysperma*	Sierra Nevada North	kv	EU274876	er	EU154089, EU154227	GQ166261	
Arab1449	*Boechera pinzliae*	Sierra Nevada South	kw	EU274920	er	EU154089, EU154227		
Arab1452	*Boechera puberula*	Great Basin Shrub Steppe central West	au	AY165370	s	AY257710		
Arab1454	*Boechera puberula*	Snake/Columbia Shrub Steppe	au	AY165370	o	AY257706		
Arab1456	*Boechera puberula*	Snake/Columbia Shrub Steppe	kx	EU274921	s	AY257710		
Arab1460	*Boechera "pulchra"*	Great Basin Shrub Steppe central East	ky	EU274922	et	EU154091, EU154229		
Arab1461	*Boechera "pulchra"*	Colorado Plateau Shrub Steppe West	la	EU274923	eu	EU154092, EU154230		
Arab1462	*Boechera "pulchra"*	Colorado Plateau Shrub Steppe West	lb	EU274924	ev	EU154093, EU154231	GQ166262	
Arab1463	*Boechera "pulchra"*	Colorado Plateau Shrub Steppe West	lc	EU274877	ew	EU154094, EU154232	GQ166263	

Accession	Country	State/ Province	County	Herbarium	Herbarium number	Collector	Coll. number	X coordi-nate	Y coordi-nate
Arab1435	USA	Utah	Washington	DAO	564768	N. H. Holmgren, J. L. Reveal, C. la France	1986	-112.98	37.33
Arab1436	USA	Utah	Kane	DAO	625366	A. Cronquist	10080	-112.05	37.51
Arab1437	USA	Utah	Garfield	DAO	243185	J. L. Reveal, J. L. Gentry jr., G. Davidse	776	-112.11	37.53
Arab1440	USA	Nevada	White Pine	DAO	582035	A. Tiehm	9440	-115.01	39.41
Arab1441	USA	Nevada	White Pine	DAO	579046	A. Tiehm, B. Ertter, M. Williams	9390	-115.38	39.31
Arab1442	USA	Utah	Kane	DAO	402639	C. Reed, K. W. Rollins, A. G. Roads	83182	-112.56	37.50
Arab1443b	USA	Nevada	White Pine	DAO	564798	N. H. Holmgren, J. L. Reveal, C. La France	2178	-115.40	38.91
Arab1444	USA	Nevada	Clark	DAO	564800	A. Cronquist	9957	-115.23	35.58
Arab1445	USA	Colorado	Rio Blanco	DAO	320273	A. Cronquist	11455	-108.83	39.70
Arab1446	USA	[California]	San Diego	DAO	564801	Brown et al		-116.55	33.06
Arab1446	USA	California	San Diego	DAO	564801	Brown et al		-116.55	33.06
Arab1447	USA	Nevada	Washoe	DAO	540844	A. Tiehm	10834	-119.90	39.30
Arab1449	USA	California	Fresno	DAO	564827	C. H. Quibell	4036	-119.03	37.45
Arab1452	USA	Nevada	Lander	DAO	578992	A. Tiehm	9699	-116.88	40.43
Arab1454	USA	Oregon	Harney	DAO	599728	T. Mosquin, L. mosquin	4347	-119.61	43.41
Arab1456	USA	Oregon	Harney	DAO	599716	T. Mosquin, L. Mosquin	4349	-119.61	43.41
Arab1460	USA	Nevada	White Pine	DAO	564843	N. H. Holgren, J. L. Reveal	988	-114.35	38.83
Arab1461	USA	Colorado	Mesa	DAO	564844	W. A. Weber, D. Murray		-108.18	39.35
Arab1462	USA	Utah	Uintah						
Arab1463	USA	Utah	Kane	DAO	625593	A. Cronquist	10019	-111.43	37.18

Accession	Taxon	wwf_ecoregion	ITS-type	ITS accession number	trnL/F type	trnLF accession numbers	*At2g25920 orthologue*	*At3g18900 orthologue*
Arab1464	*Boechera "pulchra"*	California Interior Chaparral and Woodlands	ld	EU274925	ev	EU154093, EU154231	GQ166264	
Arab1468	*Boechera pygmaea*	Sierra Nevada South					GQ166333	
Arab1469	*Boechera rectissima*	Sierra Nevada South	le	EU274926	ey	EU154096, EU154234	GQ166265	
Arab1473	*Boechera schistacea*	Colorado Plateau Shrub Steppe	sh	EU275061	fa	EU154098, EU154236	GQ166334	
Arab1474	*Boechera gracilenta*	Colorado Plateau Shrub Steppe West	gz	EU274878	ci	AY257778	GQ166266	
Arab1475	*Boechera gracilenta*	Colorado Plateau Shrub Steppe West	ad	AY165328	ci	AY257778	GQ166267	
Arab1477	*Boechera gracilenta*	Colorado Plateau Shrub Steppe West	lf	EU274927	fb	EU154099, EU154237	GQ166268	
Arab1478	*Boechera gracilenta*	Colorado Plateau Shrub Steppe West	ad	AY165328	bw	AY257766	GQ166335	
Arab1480	*Boechera dentata*		lg	EU274846	z	AY257717		
Arab1492	*Boechera sparsiflora*	Snake/Columbia Shrub Steppe	gx	EU274871	ci	AY257778		
Arab1493	*Boechera sparsiflora*	Great Basin Shrub Steppe West	gx	EU274871	b	AY257693		
Arab1495	*Boechera subpinnatifida*	Klamath Siskiyou Forests	li	EU274928	fe	EU154102, EU154240	GQ166269	
Arab1496	*Boechera suffrutescens*	South Central Rockies Forest West	lk	EU274879	ff	EU154103, EU154241	GQ166270	
Arab1499	*Halimolobos perplexa*			EU275062			GQ166271	HE589967
Arab1500	*Polyctenium fremontii*			EU275063				
Arab1501	*Polyctenium fremontii*			EU275064				
Arab1502	*Polyctenium fremontii*			EU275065				
Arab1513	*Boechera missouriensis*	Appalachian/Blue Ridge Forest	ll	EU274929	fg	EU154104, EU154242		
Arab1514	*Cusickiella douglasii*			EU275066				
Arab1515	*Cusickiella douglasii*			EU275067				

Accession	Country	State/ Province	County	Herbarium	Herbarium number	Collector	Coll. number	X coordi-nate	Y coordi-nate
Arab1464	USA	California	San Luis Obispo	DAO	634475	R. F. Hoover	8203	-119.75	35.03
Arab1468	USA	California	Tulare	DAO	564849	J. T. Howell		-118.25	36.56
Arab1469	USA	California	Tulare	DAO	231531	P. Kenny, Connrad and Dunn	549	0.00	0.00
Arab1473	USA	Utah	Garfield	DAO	402637	C. Reed, K. W. Rollins, A. G. Roads	83161	-112.35	37.93
Arab1474	USA	Colorado	Mesa	DAO	564986	W. A. Weber		-108.75	39.10
Arab1475	USA	Colorado	Monterey						
Arab1477	USA	Utah	Uintah	DAO	564984	N. H. Holgern, J. L. Reveal, C. La France	1801	-109.20	39.85
Arab1478	USA	Colorado	Delta	DAO	564985	W. A. Weber	7565	-107.95	38.93
Arab1480	Canada	Ontario	Essex	DAO	661120	M. J. Oldham, G. M. Allen, J. R. Brown, A. A. Reznicek et al	7169	-82.85	41.85
Arab1492	USA	Idaho	Twin Falls	DAO	599252	T. Mosquin, L. Mosquin	4338	-114.46	42.55
Arab1493	USA	Nevada	Washoe	DAO	599255	T. Mosquin, J. M. Gillett	5308	-119.80	39.56
Arab1495	USA	California	Humboldt	DAO	565065	J. P. Tracy	18389	0.00	0.00
Arab1496	USA	Idaho	Camas	DAO	565066	C. L. Hitchcock		-114.80	43.50
Arab1499	USA	Idaho	Payette	DAO	567408	C. L. Hichcock, C. V. Muhlick	21789	-116.28	45.18
Arab1500	USA	Oregon	Klamath	DAO	96547	A. Cronquist	8207	-121.5	42.23
Arab1501	USA	Oregon	Malheur	DAO	96545	C. L. Hitchcock, C.V. Muhlick	21035	-117.25	43.05
Arab1502	USA	California	Lassen	DAO	662324	A. Tiehm, J. Nachlinger, M. Williams	11703	-120.25	40.53
Arab1513	USA	North Carolina	Burke	GH		R. L. Wilbur	7035	-81.88	35.88
Arab1514	USA	Nevada	Elko	GH		A. Tiehm, M. Williams	10554	-116.06	41.5
Arab1515	USA	Utah	Box Elder	GH		S. Goodrich, D. Atwood	17127	-113.73	41.68

Accession	Taxon	wwf_ecoregion	ITS-type	ITS accession number	trnL/F type	trnLF accession numbers	*At2g25920 orthologue*	*At3g18900 orthologue*
Arab1517	*Boechera lyallii*	North Central Rockies Forest	ab	AY165326	fh	EU154105, EU154243		HE589968
Arab1520	*Boechera sparsiflora*	Great Basin Shrub Steppe central West	gx	EU274871	ci	AY257778		
Arab1525	*Boechera sparsiflora*	Montana Valley Foothill Grasslands	lm	EU274930	fi	EU154187, EU154325		
Arab1526	*Boechera "sparsiflora"*	Great Basin Shrub Steppe central West	gx	EU274871	cy	AY257794		
Arab1527	*Boechera sparsiflora*	Snake/Columbia Shrub Steppe	gx	EU274871	fk	EU154188, EU154326		
Arab1529	*Boechera suffrutescens*	South Central Rockies Forest West	lk	EU274879	ff	EU154103, EU154241		
Arab1535	*Boechera crandallii*	Colorado Rockies Forest	ln	EU274931	ci	AY257778	GQ166272	HE589969
Arab1537	*Boechera crandallii*	Colorado Rockies Forest	lo	EU274880	ci	AY257778	GQ166273	HE589970
Arab1539	*Boechera crandallii*	Colorado Plateau Shrub Steppe West	lp	EU274932	ci	AY257778		HE589971
Arab1540	*Boechera crandallii*	Colorado Rockies Forest	sl	EU275068	cl	AY257781	GQ166274	HE589972
Arab1542	*Boechera lyallii*	Sierra Nevada South	v	AY165353	bt	AY257763		HE589973
Arab1554	*Boechera missouriensis*	Southeastern Mixed Forest	lr	EU274847	fl	EU154189, EU154327		
Arab1555	*Boechera missouriensis*	Southeastern Mixed Forest	ls	EU274842	fl	EU154189, EU154327		
Arab1556	*Boechera missouriensis*	Southeastern Mixed Forest	ls	EU274842	fl	EU154189, EU154327		
Arab1566	*Boechera macounii*	South Central Rockies Forest East	h	AY165348	m	AY257704	GQ166275	
Arab1569	*Boechera macounii*	Colorado Plateau Shrub Steppe West	ad	AY165328	fm	EU154106, EU154244		
Arab1571	*Boechera macounii*	South Central Rockies Forest West	lt	EU274933	m	AY257704		
Arab1572	*Boechera macounii*	Snake/Columbia Shrub Steppe	h	AY165348	fn	EU154190, EU154328		
Arab1573	*Boechera macounii*	Great Basin Shrub Steppe central East	il	EU274866	ci	AY257778	GQ166336	
Arab1574	*Boechera macounii*	Great Basin Shrub Steppe West	h	AY165348	fo	EU154191, EU154329		

Accession	Country	State/ Province	County	Herbarium	Herbarium number	Collector	Coll. number	X coordi-nate	Y coordi-nate
Arab1517	Canada	British Columbia		GH	6641	E. Scamman		-115.65	50.91
Arab1520	USA	Nevada	Washoe	GH		M. J. Williams, A. Thiem	80-15-4	-117.41	38.31
Arab1525	USA	Idaho	Power	GH		N. H. Holmgren, P. K. Holmgren	4818	-113.12	45.86
Arab1526	USA	Nevada	Nye	GH		S. K. Harris	21283	-117.43	38.85
Arab1527	USA	Nevada	Humboldt	GH		A. Tiehm, S. Tucker	7259	-118.68	41.73
Arab1529	USA	Idaho	Blaine	GH		R. C. Rollins, K. W. Rollins	79276	-114.30	43.66
Arab1535	USA	Colorado	Gunnison	GH	10206	H. D. Ripley, R. C. Barneby		-107.30	38.30
Arab1537	USA	Colorado	Gunnison	GH		R. C. Rollins	2084	-106.41	38.45
Arab1539	USA	Colorado	Montrose	GH		W. A. Weber, P. Miller		-107.65	38.53
Arab1540	USA	Colorado	Gunnison						
Arab1542	USA	California	Tuolumne	GH		R. F. Hoover	4487	-119.65	38.20
Arab1554	USA	North Carolina	Stanly	GH		S. w. Leonard, A. E. Radforf		-80.26	35.23
Arab1555	USA	South Carolina	Lancaster	GH		H. E. Ahles, J. G. Haesloop	23095	-80.51	34.65
Arab1556	USA	South Carolina	Lancaster	GH		J. R. Bozeman, J. Logue, S. Spongberg, D. Culwell	8817	-80.51	34.66
Arab1566	USA	Wyoming	Fremont	GH		H. Marriott	10274	-108.75	42.50
Arab1569	USA	Utah	Uintah	GH		S. Welsh, N. Neese	18341	-109.16	40.48
Arab1571	USA	Idaho	Idaho						
Arab1572	USA	Oregon	Harney						
Arab1573	USA	Nevada	White Pine	GH		A. Tiehm, S. Crisafulli	11798	-114.65	39.16
Arab1574	USA	Nevada	Humboldt	GH		A. Tiehm	11041	-118.08	41.71

Accession	Taxon	wwf_ecoregion	ITS-type	ITS accession number	trnL/F type	trnLF accession numbers	*At2g25920 orthologue*	*At3g18900 orthologue*
Arab1577	*Boechera macounii*	South Central Rockies Forest West	h	AY165348	b	AY257693	GQ166276	HE589974
Arab1580	*Boechera pallidifolia*	Colorado Plateau Shrub Steppe East	lu	EU274934	cl	AY257781		HE589975
Arab1591	*Cusickiella douglasii*			EU275069				
Arab1592	*Cusickiella douglasii*			EU275070				
Arab1593	*Cusickiella douglasii*			EU275071				HE589976
Arab1598	*Halimolobos perplexa var. lemhiensis*			EU275072			GQ166277	HE589977
Arab1599	*Polyctenium fremontii*			EU275009			GQ166278	
Arab1600	*Polyctenium fremontii*			EU275010			GQ166279	
Arab1601	*Boechera "sparsiflora"*	California Coastal Sage and Chaparral	lv	EU274935	bu	AY257764		
Arab1604	*Boechera williamsii*	South Central Rockies Forest East	bt	AY165331	as	AY257736		
Arab1606	*Boechera subpinnatifida*	Eastern Cascades Forest	lw	EU274936	m	AY257704		
Arab1607	*Boechera subpinnatifida*	Great Basin Shrub Steppe East	lx	EU274937				
Arab1608	*Boechera subpinnatifida*	Klamath Siskiyou Forests	ac	AY165327	m	AY257704		
Arab1609	*Boechera suffrutescens*	Sierra Nevada North	gx	EU274871	fq	EU154108, EU154246	GQ166280	
Arab1610	*Boechera suffrutescens*	Sierra Nevada North	lk	EU274879	fe	EU154102, EU154240	GQ166337	
Arab1611	*Boechera lemmonii*	South Central Rockies Forest West	er	AY165339	m	AY257704	GQ166281	HE589978
Arab1612	*Boechera lemmonii*	Cascade Mountains Leeward Forests	er	AY165339	ci	AY257778		
Arab1613	*Boechera lemmonii*	Colorado Rockies Forest	ly	EU274882	fs	EU154109, EU154247	GQ166338	
Arab1614	*Boechera lemmonii*	Sierra Nevada South	er	AY165339	ci	AY257778		HE589979
Arab1615	*Boechera lemmonii*	North Central Rockies Forest	er	AY165339	m	AY257704		HE589980
Arab1617	*Boechera lemmonii*	Central and Southern Cascades Forest	er	AY165339	ci	AY257778	GQ166282	
Arab1618	*Boechera lemmonii*	Wasatch/Uinta Montane Forest	lz	EU274938				HE589981
Arab1619	*Boechera lemmonii*	Central and Southern Cascades Forest	er	AY165339	ci	AY257778		

Accession	Country	State/ Province	County	Herbarium	Herbarium number	Collector	Coll. number	X coordi-nate	Y coordi-nate
Arab1577	USA	Idaho	Lemhi	GH		R. C. Rollins	57248	-113.56	44.61
Arab1580	USA	Colorado	Gunnison	GH		B. C. Johnston	2400	-106.86	38.60
Arab1591	USA	Idaho	Elmore	GH		D. Atwood	12442	-115.73	43.36
Arab1592	USA	Nevada	Humboldt	GH		N. H. Holmgren, P. K. Holmgren	4097	-118.46	41.32
Arab1593	USA	Nevada	Humboldt	GH		A. Tiehm, J. Nachlinger	12971	-117.3	41.8
Arab1598	USA	Idaho	Lemhi	GH		P. Lesica, A. Garde	5288	-114.68	45.36
Arab1599	USA	Nevada	Washoe	GH		A. Tiehm, J. Nachlinger, G. Schoolcraft	8820	-119.9	40.91
Arab1600	USA	Idaho	Lincoln	GH				-114.56	43.15
Arab1601	USA	California	San Diego	GH		B. Anderson	2537	-116.68	33.36
Arab1604	USA	Wyoming	Fremont	GH		D. Atwood, H. Marriott	12775	-109.43	43.70
Arab1606	USA	California	Modoc	GH		B. Bartholomew, B. Anderson	4177	-120.38	41.23
Arab1607	USA	Utah	Millard	GH		W. P. Cottam	7123	-113.45	38.63
Arab1608	USA	Oregon	Josephine	GH		L. Constance, R. C. Rollins		-123.48	42.51
Arab1609	USA	California	Plumas						
Arab1610	USA	California	Plumas	GH		J. T. Howell, G. H. True		-120.88	40.05
Arab1611	USA	Idaho	Blaine	GH		J. W. Thompson		-114.51	43.85
Arab1612	Canada	British Columbia		GH		J. A. Calder, J. A. Parmelee, r. l. Taylor	19633A	-120.21	49.06
Arab1613	USA	Colorado	Gunnison						
Arab1614	USA	California	Mono	GH		M. DeDecker	4781	-118.88	37.58
Arab1615	USA	Montana	Glacier	GH		A. DeBolt, P. Lesica	3495	-113.63	48.86
Arab1617	USA	Oregon	Deschutes						
Arab1618	USA	Utah	Duchesne	GH		S. L. Welsh, E. Neese	19962	-110.40	40.71
Arab1619	USA	Washington		GH		J. William Thompson	15045b	-121.26	46.80

**Accession**	**Taxon**	**wwf_ecoregion**	**ITS-type**	**ITS accession number**	**trnL/F type**	**trnLF accession numbers**	***At2g25920 orthologue***	***At3g18900 orthologue***
Arab1620	*Boechera lemmonii*	South Central Rockies Forest East	ly	EU274882	ci	AY257778		
Arab1621	*Boechera lignifera*	Colorado Plateau Shrub Steppe East	gz	EU274878				
Arab1622	*Boechera lignifera*	South Central Rockies Forest West	h	AY165348				
Arab1624	*Boechera lignifera*	Wasatch/Uinta Montane Forest	ac	AY165327				
Arab1626	*Boechera lyallii*	South Central Rockies Forest East	l	AY165319	ft	EU154110, EU154248		
Arab1627	*Boechera lyallii*	Eastern Cascades Forest	ad	AY165328	ah	AY257725		
Arab1628	*Boechera lyallii*	North Central Rockies Forest	ab	AY165326	m	AY257704	GQ166283	
Arab1629	*Boechera lyallii*	Great Basin Shrub Steppe central East	l	AY165319	bt	AY257763		
Arab1630	*Boechera lyallii*	South Central Rockies Forest West	ad	AY165328	as	AY257736		
Arab1631	*Boechera canadensis*		sc	EU274860	ei	EU154081, EU154219		
Arab1632	*Boechera canadensis*	Ozark Mountain Forests	ma	EU274848	fu	EU154111, EU154249	GQ166339	HE589982
Arab1633	*Boechera canadensis*	Southeastern Mixed Forest	mb	EU274849	fv	EU154112, EU154250		
Arab1634	*Boechera canadensis*		sd	EU274861	ei	EU154081, EU154219	GQ166340	HE589983
Arab1635	*Boechera canadensis*	Southeastern Mixed Forest	mc	EU274850	fw	EU154192, EU154330		HE589984
Arab1638	*Boechera canadensis*		md	EU274851	fx	EU154193, EU154331	GQ166284	HE589985
Arab1640	*Boechera breweri*	California Interior Chaparral and Woodlands	ha	EU274883	fz	EU154195, EU154333	GQ166285	HE589986
Arab1641	*Boechera suffrutescens*	South Central Rockies Forest West	me	EU274939				
Arab1642	*Boechera suffrutescens*						GQ166286	
Arab1643	*Boechera "sparsiflora"*	California Montane Chaparral and Woodlands	og	EU274884	ci	AY257778		

Accession	Country	State/ Province	County	Herbarium	Herbarium number	Collector	Coll. number	X coordi-nate	Y coordi-nate
Arab1620	USA	Wyoming	Lincoln	GH		L. M. Shultz, J. S. Shultz	3616	-110.83	42.75
Arab1621	USA	Colorado	Rio Blanko	GH		R. C. Rollins, K. W. Rollins, S. Peterson, K. Wiley-Eberle, A. Roads, D. Wilken	8397	-108.43	39.95
Arab1622	USA	Montana	Beaverhead	GH		P. Lesica	3099	-112.88	45.30
Arab1624	USA	Utah	Uintah	GH		N. H. Holmgren, P. K. Holmgren	5104	-109.48	40.63
Arab1626	USA	Wyoming	Fremont	GH		R. W. Scott	853	-110.03	43.78
Arab1627	USA	Washington State	Yakima	GH		J. W. Thompson	15032	-121.26	46.80
Arab1628	USA	Montana		GH		R. C. Rollins	57213	-113.66	48.78
Arab1629	USA	Nevada	Elko	GH		D. Charlet	381		
Arab1630	USA	Idaho	Custer	GH		R. C. Rollins, K. W. Rollins	86170	-114.35	44.36
Arab1631	Canada	Quebec	Ottawa	GH		B. Boivin, L. Jenkins, L. J. Van Rens	14296	-75.93	45.58
Arab1632	USA	Arkansas	Montgomery			D. E. Boufford, V. M. Bates, E. W. Wood	25488	-93.91	34.41
Arab1633	USA	Mississippi	Jefferson	GH		J. D. Ray, Jr.	8341	-91.20	31.58
Arab1634	USA	New York	Columbia	GH		H. D. House	22690	-73.81	42.18
Arab1635	USA	North Carolina	Stanly			D. E. Boufford, H E. Ahles	9830	-80.25	35.50
Arab1638	USA	Georgia	Randolph	GH		R. M. Harper	2229	-84.75	31.78
Arab1640	USA	California	Contra Costa	GH		L. Constance, J. L. Morrison	2192	-121.93	37.90
Arab1641	USA	Idaho	Blaine	GH		R. C. Rollins, K. W. Rollins	86133	-114.71	43.88
Arab1642	USA	Nevada	Douglas	GH		A. Pinz	7440	-119.9	39.1
Arab1643	USA	California	Santa Barbara						

Accession	Taxon	wwf_ecoregion	ITS-type	ITS accession number	trnL/F type	trnLF accession numbers	*At2g25920 orthologue*	*At3g18900 orthologue*
Arab1644	*Boechera "sparsiflora"*	California Montane Chaparral and Woodlands	og	EU274884	ci	AY257778		
Arab1646	*Boechera "sparsiflora"*	Snake/Columbia Shrub Steppe	mf	EU274940	as	AY257736		
Arab1647	*Boechera "sparsiflora"*	Great Basin Shrub Steppe West	h	AY165348				
Arab1648	*Boechera "sparsiflora"*	Great Basin Shrub Steppe central East	f	AY165346	ci	AY257778		
Arab1649	*Boechera "sparsiflora"*	Snake/Columbia Shrub Steppe	h	AY165348	fz	EU154195, EU154333		
Arab1651	*Polyctenium fremontii*			EU275073			GQ166287	
Arab1652	*Polyctenium fremontii*			EU275074				
Arab1653	*Polyctenium fremontii*			EU275075				
Arab1654	*Polyctenium fremontii*			EU275076				
Arab1656	*Polyctenium williamsiae*			EU275077				
Arab1657	*Polyctenium williamsiae*			EU275078				
Arab1661	*Boechera cobrensis*	Great Basin Shrub Steppe West	ad	AY165328			GQ166288	HE589987
Arab1662	*Boechera cusickii*	Palouse Grasslands	h	AY165348	m	AY257704		
Arab1663	*Boechera cusickii*	Palouse Grasslands	h	AY165348				
Arab1664	*Bochera davidsonii*	Sierra Nevada North	mg	EU274941	KF	EU154183, EU154321		
Arab1665	*Bochera davidsonii*	Sierra Nevada	mh	EU274942	kg	EU154184, EU154322		
Arab1666	*Bochera davidsonii*	Great Basin Shrub Steppe West	oq	EU274870	kh	EU154185, EU154323		
Arab1667	*Bochera davidsonii*	Great Basin Shrub Steppe West	oq	EU274870				
Arab1668	*Boechera cobrensis*	Great Basin Shrub Steppe central West	gy	EU274873	ci	AY257778	GQ166289	HE589988
Arab1669	*Boechera cusickii*	Snake/Columbia Shrub Steppe	mi	EU274943				
Arab1670	*Boechera cusickii*	Palouse Grasslands	b	AY165313				

Accession	Country	State/ Province	County	Herbarium	Herbarium number	Collector	Coll. number	X coordi-nate	Y coordi-nate
Arab1644	USA	California	Los Angeles	GH		S. Boyd, L. Raz	9647	-118.30	34.56
Arab1646	USA	Idaho	Twin Falls	GH		N. H. Holmgren, P. K.Holmgren	5781	-114.83	42.08
Arab1647	USA	Nevada	Douglas	GH		N. H. Holmgren, P. K. Holmgren	5690		
Arab1648	USA	Utah	Box Elder	GH		N. H. Holmgren, P. K. Holmgren	4844		
Arab1649	USA	Washington	Grant	GH		X. M. Gaines, T. H. Scheffer	353	-119.33	47.60
Arab1651	USA	Idaho	Blaine	GH		N. D. Atwood	10124	-114.26	43.3
Arab1652	USA	California	Lassen	GH		A. Tiehm, J. Nachlinger, G. SChoolcraft	8825	-120.05	40.88
Arab1653	USA	Nevada	Washoe	GH		A. Tiehm	8091	-119.31	41.31
Arab1654	USA	Oregon	Harney	GH		B. Ertter	5740	-119.63	43.4
Arab1656	USA	Nevada	Lyon	GH		A. Tiehm	12806	-119.41	39.2
Arab1657	USA	Nevada	Washoe	GH		R. C. Rollins, K. W. Rollins, A. G. Roads, A. Tiehm, M. Williams	83219	-119.7	39.28
Arab1661	USA	Nevada	Esmeralda	GH		J. D. Morefield, R. Price	4625	-118.26	37.85
Arab1662	USA	Washington	Whitman	GH		R. C. Rollins, L. Constance	1098	-117.63	47.23
Arab1663	USA	Washington	Whitman	GH		R. C. Rollins, L. Constance	1104	-117.75	47.15
Arab1664	USA	California	Plumas	GH		L. Ahart	5355	-120.86	39.78
Arab1665	USA	California	Plumas	GH		B. Anderson	2804	-120.70	39.76
Arab1666	USA	Nevada	Humboldt	GH		A. Tiehm	9729	-117.65	41.55
Arab1667	USA	Nevada	Humboldt	GH		A. Tiehm	9091	-117.78	41.41
Arab1668	USA	Nevada	Humboldt	GH		A. Tiehm	11088	-118.11	41.10
Arab1669	USA	Idaho	Gooding	GH		B. Ertter	4212	-114.61	43.20
Arab1670	USA	Oregon	Gilliam	GH		A. N. Steward	6874	-119.95	45.11

Accession	Taxon	wwf_ecoregion	ITS-type	ITS accession number	trnL/F type	trnLF accession numbers	*At2g25920 orthologue*	*At3g18900 orthologue*
Arab1671	*Boechera inyoensis*	California Montane Chaparral and Woodlands	mk	EU274944	ci	AY257778		
Arab1672	*Boechera inyoensis*	Sierra Nevada South	i	AY165317	ci	AY257778		
Arab1673	*Boechera inyoensis*	Great Basin Shrub Steppe West	ad	AY165328				HE589989
Arab1674	*Boechera inyoensis*	Great Basin Shrub Steppe West	ml	EU274945	aa	AY257718	GQ166290	HE589990
Arab1675	*Boechera koehleri*	Klamath Siskiyou Forests	ac	AY165327	ci	AY257778		
Arab1677	*Boechera koehleri*	Klamath Siskiyou Forests	mm	EU274885	gc	EU154198, EU154336	GQ166291	HE589991
Arab1678	*Boechera koehleri*	Klamath Siskiyou Forests	sm	EU275079	gc	EU154198, EU154336	GQ166292	HE589992
Arab1681	*Boechera gracilenta*	Wasatch/Uinta Montane Forest North	ad	AY165328	ci	AY257778		
Arab1682	*Boechera gracilenta*	Colorado Plateau Shrub Steppe West	ad	AY165328	gd	EU154113, EU154251	GQ166293	
Arab1683	*Boechera gracilenta*	Wasatch/Uinta Montane Forest	ad	AY165328				
Arab1684	*Boechera shockleyi*	Mojave Desert	mn	EU274946	ge	EU154114, EU154252		
Arab1685	*Boechera shockleyi*	Great Basin Shrub Steppe West	mo	EU274886	ab	AY257719		
Arab1686	*Boechera fendleri*	Great Basin Shrub Steppe East	sn	EU275080	gf	EU154115, EU154253	GQ166294	HE589993
Arab1687	*Boechera "sparsiflora"*	Sierra Nevada South	i	AY165317	ci	AY257778		
Arab1688	*Boechera "sparsiflora"*	California Coastal Sage and Chaparral	og	EU274884	bu	AY257764		
Arab1692	*Boechera rectissima*	Sierra Nevada North	z	AY165325	gi	EU154117, EU154255		
Arab1693	*Boechera rectissima*	Sierra Nevada North	z	AY165325	gk	EU154118, EU154256		
Arab1694	*Boechera rectissima*	Sierra Nevada North	mp	EU274947	gl	EU154119, EU154257	GQ166295	HE589994
Arab1695	*Boechera repanda*	California Montane Chaparral and Woodlands	mr	EU274948	gm	EU154120, EU154258		HE589995

Accession	Country	State/ Province	County	Herbarium	Herbarium number	Collector	Coll. number	X coordi-nate	Y coordi-nate
Arab1671	USA	California	San Bernardino	GH		C. Davidson, W. Wisura, L. Goodman, T. Krantz	49917	-116.91	34.13
Arab1672	USA	California	Inyo	GH		M. Z. Thorne, M. DeDecker	38791	-118.33	36.76
Arab1673	USA	California	Mono	GH		J. D. Morefield	4381	-118.13	37.68
Arab1674	USA	California	Mono	GH		J. D. Morefield	3628	-118.11	37.63
Arab1675	USA	California	Siskiyou	GH		C. A. Ground, G. J. Muth	1789	-123.63	41.85
Arab1677	USA	California	Siskiyou	GH		C. A. Ground	871	-123.61	41.83
Arab1678	USA	California	Siskiyou	GH		C. A. Ground	635	-123.61	41.83
Arab1681	USA	Utah	Daggett	GH		R. C. Rollins, K. W. Rollins	8660	-109.71	40.93
Arab1682	USA	Utah	Kane	GH		R. C. Rollins, K. W. Rollins	8179	-112.63	37.28
Arab1683	USA	Utah	Uintah	GH		R. C. Rollins, K. W. Rollins	79131	-109.53	40.61
Arab1684	USA	Nevada	Lincoln	GH		P. V. Wells		-115.80	36.90
Arab1685	USA	Nevada	Nye						
Arab1686	USA	Nevada	Nye	GH		K. H. Thorne, B. T. Welsh	884	-115.03	38.65
Arab1687	USA	Nevada	Tulare	GH		J. R. Shevock	1596	-118.41	36.23
Arab1688	USA	California	San Diego	GH		R. M. Beauchamp, C. Harbison, B. Mackintosh	2642	-116.61	32.96
Arab1692	USA	California	Plumas	GH		R. C. Rollins, K. W. Rollins	81163	-120.46	39.88
Arab1693	USA	California	Lassen	GH		R. C. Rollins, K. W. Rollins	81172	-120.70	40.45
Arab1694	USA	California	El Dorado	GH		B. Brown	38	-120.30	38.76
Arab1695	USA	California	San Bernardino						

Accession	Taxon	wwf_ecoregion	ITS-type	ITS accession number	trnL/F type	trnLF accession numbers	***At2g25920* orthologue**	***At3g18900* orthologue**
Arab1696	*Boechera repanda*	California Central Valley Grasslands	ms	EU274949	gn	EU154121, EU154259		
Arab1697	*Boechera repanda*	Sierra Nevada South			go	EU154199, EU154337	GQ166296	HE589996
Arab1698	*Boechera rigidissima*	Sierra Nevada	kv	EU274876	bd	AY257747		
Arab1699	*Boechera pallidifolia*	Colorado Plateau Shrub Steppe East	ad	AY165328	gp	EU154200, EU154338		HE589997
Arab1701	*Boechera xylopoda*	Mojave Desert	mt	EU274950			GQ166297	
Arab1702	*Boechera lincolnensis*	Mojave Desert	mu	EU274951	gq	EU154201, EU154339		
Arab1703	*Boechera lincolnensis*	Mojave Desert	so	EU275081	gr	EU154202, EU154340		
Arab1704	*Boechera formosa*	Colorado Plateau Shrub Steppe	lc	EU274877	gs	EU154203, EU154341		
Arab1705	*Boechera breweri*	Klamath Siskiyou Forests	mv	EU274952			GQ166298	
Arab1706	*Boechera breweri*	Klamath Siskiyou Forests	ha	EU274883	bu	AY257764	GQ166299	
Arab1707	*Boechera breweri*	Klamath Siskiyou Forests	gx	EU274871	gt	EU154122, EU154260		
Arab1708	*Boechera xylopoda*	Great Basin Shrub Steppe West	mw	EU274887	gq	EU154201, EU154339		
Arab1709	*Boechera xylopoda*	Great Basin Shrub Steppe central West	mw	EU274887	gu	EU154123, EU154261		
Arab1710	*Boechera lincolnensis*	Mojave Desert	mx	EU274953	gq	EU154201, EU154339		
Arab1713	*Boechera platysperma*	Sierra Nevada North	my	EU274954	gv	EU154124, EU154262		
Arab1714	*Boechera platysperma*	California Montane Chaparral and Woodlands	kv	EU274876	ci	AY257778		
Arab1715	*Boechera platysperma*	Eastern Cascades Forest	mz	EU274955	gw	EU154125, EU154263		
Arab1716	*Boechera platysperma*	Sierra Nevada North	na	EU274956	gx	EU154126, EU154264		

Accession	Country	State/ Province	County	Herbarium	Herbarium number	Collector	Coll. number	X coordi-nate	Y coordi-nate
Arab1696	USA	California	Tulare	GH		J. R. Shevock	2010	-120.33	36.31
Arab1697	USA	California	Tulare	GH		J. T. Howell		-118.11	36.26
Arab1698	USA	Nevada	Washoe	GH		A. Tiehm, B. Ertter, J. Strachan	8268	-119.88	39.20
Arab1699	USA	Colorado	Montezuma	GH		R. C. Rollins, F. A. Stafleu	7135	-108.68	37.35
Arab1701	USA	California	Inyo	GH		R. C. Rollins, K. W. Rollins	7865	-117.13	36.40
Arab1702	USA	Nevada	Clark	GH		R. C. Rollins, K. W. Rollins	7832	-115.90	36.20
Arab1703	USA	Nevada	Esmeralda	GH		A. Tiehm, M. Williams	11690	-117.55	37.93
Arab1704	USA	Colorado	Montezuma	GH		R. C. Rollins, F. A. Stafleu	7150	-109.03	37.26
Arab1705	USA	California	Siskiyou	GH			15177	-122.56	41.85
Arab1706	USA	California	Siskiyou	GH	2901	L. Constance, R. C. Rollins		-123.03	41.76
Arab1707	USA	California	Siskiyou	GH		L. Constance, R. C. Rollins		-123.43	41.67
Arab1708	USA	Nevada	Nye	GH		A. Tiehm	6932a	-116.68	38.28
Arab1709	USA	Nevada	Nye	GH		S. Goodrich		-116.68	38.28
Arab1710	USA	Nevada	Nye						
Arab1713	USA	California	Nevada	GH		G. H. True, J. T. Howell	5595	-120.31	39.41
Arab1714	USA	California	San Bernardino	GH		C. Davidson, W. Wisura, L. Goodman, T. Krantz	49840	-116.80	34.11
Arab1715	USA	California	Modoc	GH		D. Charlet	532	-120.21	41.30
Arab1716	USA	California	Nevada	GH		L. C. Higgins	1739	-120.33	39.31

**Accession**	**Taxon**	**wwf_ecoregion**	**ITS-type**	**ITS accession number**	**trnL/F type**	**trnLF accession numbers**	***At2g25920 orthologue***	***At3g18900 orthologue***
Arab1717	*Boechera platysperma*	Sierra Nevada North	nb	EU274957	gy	EU154127, EU154265	GQ166341	HE589998
Arab1718	*Boechera platysperma*	Sierra Nevada North	nc	EU274958	cy	AY257794		
Arab1720	*Boechera platysperma*	Sierra Nevada South	kv	EU274876	gz	EU154128, EU154266	GQ166300	
Arab1722	*Boechera perennans*	Sonora Desert	nd	EU274959	ci	AY257778	GQ166301	
Arab1723	*Boechera perennans*	Sonora Desert	ev	AY165341	ci	AY257778	GQ166302	
Arab1724	*Boechera perennans*		ne	EU274960				
Arab1726	*Boechera perennans*	Mojave Desert	ev	AY165341	ha	EU154129, EU154267		
Arab1727	*Boechera perennans*	Sierra Nevada South	ng	EU274961				
Arab1728	*Boechera perennans*	Great Basin Shrub Steppe central East			c	AY257694	GQ166303	
Arab1729	*Boechera perennans*	Mojave Desert	ev	AY165341	ci	AY257778	GQ166304	
Arab1730	*Boechera perennans*	Colorado Plateau Shrub Steppe West	ev	AY165341	ci	AY257778		
Arab1731	*Boechera inyoensis*	Sierra Nevada South	ar	AY165369	ci	AY257778	GQ166305	
Arab1732	*Boechera glaucovalvula*	Mojave Desert	nh	EU274888	hb	EU154130, EU154268	GQ166342	
Arab1733	*Boechera glaucovalvula*	Sierra Nevada South					GQ166343	
Arab1734	*Boechera glaucovalvula*	Mojave Desert	sp	EU275082	hc	EU154131, EU154269	GQ166344	
Arab1735	*Boechera glaucovalvula*	Mojave Desert	sp	EU275082	hc	EU154131, EU154269		
Arab1737	*Boechera gracilipes*	Colorado Plateau Shrub Steppe West	f	AY165346				
Arab1738	*Boechera gunnisoniana*	Colorado Plateau Shrub Steppe East	ad	AY165328	ds	DQ013060		
Arab1741	*Boechera pendulina*	Colorado Plateau Shrub Steppe	ev	AY165341	hd	EU154132, EU154270		
Arab1742	*Boechera pendulina*	Mojave Desert	f	AY165346	ci	AY257778		

Accession	Country	State/ Province	County	Herbarium	Herbarium number	Collector	Coll. number	X coordi-nate	Y coordi-nate
Arab1717	USA	California	Alpine	GH		C. B. Hardham	23796	-119.86	38.61
Arab1718	USA	Nevada	Washoe	GH		A. Tiehm	12034	-119.88	39.18
Arab1720	USA	California	Inyo	GH		G. L. Stebbins	6495	-118.65	37.33
Arab1722	USA	Arizona	Pima	GH		A. C. Gibson, L. C. Gibson	2925	-111.50	31.98
Arab1723	USA	Arizona	Pima	GH		T. Plowman	10413	-110.85	32.26
Arab1724	USA	Arizona		GH		A. Nelson, R. A. Nelson	1992	0.00	0.00
Arab1726	USA	California	San Bernardino	GH		R. F. Thorne, B. Prigge, D. Michener, S. Meyer, W. Wisura	51711	-115.53	35.03
Arab1727	USA	California	Inyo	GH		J. D. Morefield, D. H. McCarty	3372	-118.36	36.80
Arab1728	USA	Nevada	White Pine	GH		R. C. Rollins, K. W.Rollins, A. G. Roads	83191	-114.15	39.2
Arab1729	USA	Nevada	Nye	GH		A. Tiehm	10965	-117.06	37.53
Arab1730	USA	Nevada	Clark	GH		A. Pinzl	7023	-114.05	36.53
Arab1731	USA	California	Mono	GH		J. T. Howell, T. C. Fuller, G. D. Barbe		-119.16	37.71
Arab1732	USA	California	Riverside	GH		P. H. Raven	12900	-116.08	34.01
Arab1733	USA	California	Mono	GH		J. D. Morefield, D. H. McCarty	3461	-118.36	37.71
Arab1734	USA	Nevada	Nye	GH		N. H. Holmgren, P. K. Holmgren	6335	-116.86	36.85
Arab1735	USA	Nevada	Nye	GH		M. DeDecker	816	-116.88	35.83
Arab1737	USA	Arizona	Mohave	GH		F. W. Gould	1711	-113.91	35.08
Arab1738	USA	Colorado	Gunnison	GH		R. C. Rollins	2014	-106.78	38.50
Arab1741	USA	Utah	Kane	GH		R. C. Rollins, K. W. Rollins	8182	-112.55	37.33
Arab1742	USA	Nevada	Clark	GH		J. Beatley, T. Ackerman, S. Bamberg		-115.75	36.30

Accession	Taxon	wwf_ecoregion	ITS-type	ITS accession number	trnL/F type	trnLF accession numbers	*At2g25920 orthologue*	*At3g18900 orthologue*
Arab1744	*Boechera pendulina*	Wasatch/Uinta Montane Forest	g	AY165347	c	AY257694	GQ166306	
Arab1745	*Boechera pendulina*	Great Basin Shrub Steppe central East	ni	EU274962			GQ166345	
Arab1747	*Boechera pendulina*	Wasatch/Uinta Montane Forest	g	AY165347	c	AY257694	GQ166346	
Arab1748	*Boechera pendulina*	Colorado Plateau Shrub Steppe	ev	AY165341	c	AY257694		
Arab1749	*Boechera pendulina*	Wasatch/Uinta Montane Forest	ev	AY165341	he	EU154133, EU154271		
Arab1750	*Boechera pendulina*	Colorado Plateau Shrub Steppe West	kt	EU274874				
Arab1751	*Boechera perstellata*		se	EU274862	fl	EU154189, EU154327		
Arab1753	*Boechera spec.*		sr	EU275083				
Arab1754	*Boechera perennans*	Colorado Plateau Shrub Steppe West	ad	AY165328	ci	AY257778		
Arab1755	*Boechera perstellata*		se	EU274862	fl	EU154189, EU154327	GQ166347	
Arab1756	*Boechera parishii*	California Montane Chaparral and Woodlands	f	AY165346	hf	EU154134, EU154272	GQ166348	
Arab1757	*Boechera parishii*	California Montane Chaparral and Woodlands	f	AY165346	ci	AY257778	GQ166349	
Arab1758	*Boechera patens*	Appalachian/Blue Ridge Forest	st	EU275084				
Arab1759	*Boechera patens*	Appalachian/Blue Ridge Forest	st	EU275084				
Arab1761	*Boechera "pulchra"*	Great Basin Shrub Steppe West	h	AY165348	hg	EU154135, EU154273		
Arab1763	*Boechera "pulchra"*	Mojave Desert	nk	EU274963	ci	AY257778	GQ166350	
Arab1764	*Boechera "pulchra"*	Mojave Desert	nl	EU274889	ci	AY257778		
Arab1765	*Boechera "pulchra"*	California Montane Chaparral and Woodlands	su	EU275085	ci	AY257778	GQ166307	
Arab1767	*Boechera puberula*	Great Basin Shrub Steppe West	sv	EU275086	s	AY257710	GQ166351	

Accession	Country	State/ Province	County	Herbarium	Herbarium number	Collector	Coll. number	X coordi-nate	Y coordi-nate
Arab1744	USA	Utah	Sevier	GH		R. C. Rollins, K. W. Rollins, A. G. Roads	83135	-112.21	38.76
Arab1745	USA	Nevada	Elko	GH		A. Tiehm, M. Williams	6684	-114.81	40.55
Arab1747	USA	Utah	Garfield	GH		R. C. Rollins, K. W. Rollins, A. G. Roads	83518	-112.33	37.98
Arab1748	USA	Utah	Garfield	GH		J. L. Reveal, J. L. Gentry, G. Davidse	776	-112.08	37.55
Arab1749	USA	Utah	Kane	GH		R. C. Rollins, K. W. Rollins, A. G. Roads	83182	-112.56	37.48
Arab1750	USA	Utah	Washington	GH		N. H. Holmgren, J. L. Reval, C. La France	1986	-113.00	37.33
Arab1751	USA	Tennessee	Davidson	GH		K. a. Wilson, R. B. Channell	709	-86.41	35.88
Arab1753	USA	Texas	Burnet	GH		R. C. Rollins, D. S. Correll, K. L. Chambers	5792	-98.23	30.70
Arab1754	USA	Utah	San Juan	GH		R. Kass, K. Preston	2187	-109.70	37.20
Arab1755	USA	Tennessee	Davidson	GH		R. B. Channell	7996	-86.63	36.11
Arab1756	USA	California	San Bernardino	GH		R. F. Thorne, C. W. Tilforth, J. Little	53158	-116.90	34.30
Arab1757	USA	California	San Bernardino	GH		R. F. Thorne, B. Prigge, C. W. Tilforth	54321	-116.91	34.30
Arab1758	USA	Pennsylvania	Franklin	GH		J. W. Adams, E. T. Wherry	4691	-77.88	39.85
Arab1759	USA	Pennsylvania	Berks	GH		H. Wilkens	2743	-75.91	40.30
Arab1761	USA	Nevada	Douglas	GH				-119.63	39.03
Arab1763	USA	California	San Bernardino						
Arab1764	USA	California	Los Angeles	GH		R. F. Thorne, G. Wallace	43415	-117.90	34.45
Arab1765	USA	California	Los Angeles						
Arab1767	USA	Nevada	Lyon	GH		A. Tiehm	7723	-119.43	39.48

Accession	Taxon	wwf_ecoregion	ITS-type	ITS accession number	trnL/F type	trnLF accession numbers	*At2g25920 orthologue*	*At3g18900 orthologue*
Arab1768	*Boechera puberula*	Great Basin Shrub Steppe West	sf	EU275087	s	AY257710		
Arab1769	*Boechera puberula*	Great Basin Shrub Steppe central West	sw	EU275088	u	AY257712		
Arab1771	*Boechera fendleri*	Great Basin Shrub Steppe central East	ev	AY165341	en	EU154085, EU154223		
Arab1773	*Boechera fendleri*	Chihuahua desert	f	AY165346				
Arab1776	*Boechera fernaldiana*	Great Basin Shrub Steppe central West	op	EU274869	hk	EU154138, EU154276		
Arab1777	*Boechera fernaldiana*	Great Basin Shrub Steppe central West	op	EU274869	ci	AY257778		
Arab1778	*Boechera fernaldiana*	Great Basin Shrub Steppe central West	op	EU274869	hi	EU154137, EU154275		
Arab1780	*Boechera "holboellii"*	South Central Rockies Forest East	bw	AY165333	hl	EU154139, EU154277		
Arab1781	*Bochera davidsonii*	Great Basin Shrub Steppe West	oq	EU274870				
Arab1782	*Boechera oxylobula*	Colorado Plateau Shrub Steppe East	hc	AY165408	ci	AY257778	GQ166352	
Arab1784	*Boechera oxylobula*	Wasatch/Uinta Montane Forest	g	AY165347	c	AY257694	GQ166308	
Arab1785	*Boechera oxylobula*	Colorado Plateau Shrub Steppe West	hc	AY165408	ds	DQ013060	GQ166309	
Arab1786	*Boechera oxylobula*	Colorado Plateau Shrub Steppe East	hc	AY165408	ci	AY257778		
Arab1788	*Boechera fendleri*	Mojave Desert	f	AY165346	f	AY257697		
Arab1789	*Boechera fendleri*	Colorado Rockies Forest	nm	EU274964	ci	AY257778		
Arab1790	*Boechera fendleri*						GQ166310	
Arab1800	*Boechera missouriensis*		nn	EU274852	hm	EU154140, EU154278		HE589999
Arab1802	*Boechera laevigata*		or	EU274839	fg	EU154104, EU154242		HE590000
Arab1804	*Boechera laevigata*		no	EU274853	fg	EU154104, EU154242		
Arab1805	*Boechera laevigata*		np	EU274854	fg	EU154104, EU154242		HE590001

Accession	Country	State/ Province	County	Herbarium	Herbarium number	Collector	Coll. number	X coordi-nate	Y coordi-nate
Arab1768	USA	Nevada	Washoe	GH		M. J. Williams, R. Holcomb	8643	-119.88	39.55
Arab1769	USA	Nevada	Elko	GH		N. H. Holmgren, P. K. Holmgren	4891	-115.96	41.21
Arab1771	USA	Nevada	Lincoln	GH		P. Train	2664	-114.56	37.96
Arab1773	USA	New Mexico	Dona Ana						
Arab1776	USA	Nevada	Nye	GH		S. Goodrich, F. Smith	13274	-116.98	38.65
Arab1777	USA	Nevada	Nye	GH		S. K. Harris	21282	-117.45	38.83
Arab1778	USA	Nevada	Nye	GH		S. K. Harris	21307	-117.46	38.99
Arab1780	USA	Wyoming	Sublette	GH		C. L. Porter, M. W. Porter	10438	-109.70	42.95
Arab1781	USA	Nevada	Humboldt	GH		M. P. Yoder Williams	1208	-117.53	41.65
Arab1782	USA	Colorado	Gunnison	GH		B. C. Johnston	2393	-106.96	38.43
Arab1784	USA	Utah	Sevier	GH		R. Kass, E. Neese, B. Neely	2349	-112.03	38.66
Arab1785	USA	Colorado	Gunnison	GH		R. C. Rollins, K. W. Rollins, A. G. Roads	8375	-107.30	38.46
Arab1786	USA	Colorado	Gunnison	GH		R. C. Rollins, K. W. Rollins	7953	-106.61	38.50
Arab1788	USA	Arizona	Mohave	GH		C. W. Tilforth	545	-113.91	35.08
Arab1789	USA	Colorado	Hinsdale	GH		J. H. Beaman, F. H. Erbisch	1118	-107.31	38.03
Arab1790	USA	Nevada	Clark	GH		I. W. Clokey	7538	-115.65	36.25
Arab1800	USA	West Virginia	Kanawha	GH		G. C. Tucker	3306	-81.83	38.40
Arab1802	USA	Wisconsin	Jackson	GH		V. E. McNeilus	99236	-90.83	44.30
Arab1804	USA	North Carolina	Mitchell	GH		E. W. Wood, D. E. Boufford	2208	-82.11	36.10
Arab1805	USA	New York	Greene						

Accession	Taxon	wwf_ecoregion	ITS-type	ITS accession number	trnL/F type	trnLF accession numbers	*At2g25920 orthologue*	*At3g18900 orthologue*
Arab1806	*Boechera laevigata*		ot	EU274855	fg	EU154104, EU154242		
Arab1807	*Boechera laevigata*		nr	EU274856	fg	EU154104, EU154242		
Arab1808	*Boechera laevigata*		ns	EU274857	fg	EU154104, EU154242		
Arab1809	*Boechera laevigata*		nt	EU274858	fl	EU154189, EU154327		
Arab1810	*Boechera laevigata*		nu	EU274859	fg	EU154104, EU154242		
Arab1811	*Boechera arcuata*	Sierra Nevada South	sx	EU275089			GQ166353	
Arab1813	*Boechera arcuata*	Sierra Nevada South	og	EU274884				
Arab1814	*Boechera arcuata*	Sierra Nevada South	og	EU274884				
Arab1815	*Boechera atrorubens*	Cascade Mountains Leeward Forests	gx	EU274871				
Arab1816	*Boechera atrorubens*						GQ166354	
Arab1817	*Boechera bodiensis*	Great Basin Shrub Steppe West	pe	EU275012				
Arab1818	*Boechera breweri*						GQ166355	
Arab1819	*Boechera breweri*	California Central Valley Grasslands	kn	EU274872			GQ166356	
Arab1820	*Boechera breweri var. shastaensis*	Klamath Siskiyou Forests	pf	EU275013			GQ166357	HE590002
Arab1821	*Boechera breweri var. shastaensis*	Klamath Siskiyou Forests	ha	EU274883				
Arab1823	*Boechera cobrensis*	Wyoming Basin Shrub Steppe	ks	EU274918			GQ166358	
Arab1824	*Boechera cobrensis*	Wyoming Basin Shrub Steppe	ks	EU274918				HE590003
Arab1825	*Boechera constancei*	Sierra Nevada North	pg	EU275014			GQ166359	
Arab1826	*Boechera constancei*	Sierra Nevada North	ph	EU275015				HE590004
Arab1827	*Boechera constancei*	Sierra Nevada North	pi	EU275016				

Accession	Country	State/ Province	County	Herbarium	Herbarium number	Collector	Coll. number	X coordi-nate	Y coordi-nate
Arab1806	USA	Ohio	Londonville	GH		E. B. Harger	8258	-82.21	40.63
Arab1807	USA	Tennessee	Unicoi	GH		E. W. Wood, D. E. Boufford	4803	-82.33	36.11
Arab1808	USA	Tennessee	Wilson	GH		R. C. Rollins, K. W. Rollins	8723	-86.28	36.18
Arab1809	USA	Tennessee	Marshall	GH		V. E. McNeilus	98210	-86.71	35.60
Arab1810	USA	Virginia	Patrick	GH		D. E. Boufford	10096	-80.31	36.83
Arab1811	USA	California	Kern	MO	5577109	Barbara Britter, James R. Shevock	5957	-118.40	35.56
Arab1813	USA	California	Tulare County	MO	2610649	Ernest C. Twisselmann	13299	-118.48	35.96
Arab1814	USA	California	Tulare	MO	1200331	Arthur Cronquist		-118.54	36.54
Arab1815	USA	Washington	Kittitas County	MO	1202749	J. William Thompson	14511	-120.93	47.19
Arab1816	USA	Washington	Kittitas County	MO	5434440	Walter J. Eyerdam	1475	-120.41	46.98
Arab1817	USA	Nevada	Lyon County	MO	3024099	Arnold Thiem, Margaret Williams	7411	-119.48	39.19
Arab1818	USA	California	Tehema County	MO	5720918	B. A. Smith	904	-122.64	39.81
Arab1819	USA	California	Sutter County						
Arab1820	USA	California	Shasta County	MO	5720919	B. A. Smith	920	-122.04	40.75
Arab1821	USA	California	Sskiyou County						
Arab1823	USA	Wyoming	Sweetwater County	MO	4865425	M. D. Windham	3034	-109.63	41.20
Arab1824	USA	Wyoming	Uinta County						
Arab1825	USA	Californa	Plumas County	MO	3209857	M. S. Taylor, M. Foster	3827	-120.78	39.89
Arab1826	USA	California	Plumas County	MO	3834668	M. S. Taylor	4471	-121.00	40.12
Arab1827	USA	California	Plumas County	MO	5071933	John Thomas Howell, T. C. Fuller, G. D. Barbe	54150	-120.96	40.11

Accession	Taxon	wwf_ecoregion	ITS-type	ITS accession number	trnL/F type	trnLF accession numbers	*At2g25920 orthologue*	*At3g18900 orthologue*
Arab1828	*Boechera constancei*	Sierra Nevada North	pi	EU275016				
Arab1829	*Boechera crandallii*	Colorado Plateau Shrub Steppe	sb	EU275017				HE590005
Arab1830	*Boechera crandallii*	Colorado Plateau Shrub Steppe East	pk	EU275026				HE590006
Arab1832	*Boechera cusickii*	Palouse Grasslands	h	AY165348				
Arab1834	*Boechera davidsonii*	Great Basin Shrub Steppe West	pl	EU275018				
Arab1837	*Boechera "divaricarpa"*	South Central Rockies Forest East	l	AY165319				
Arab1838	*Boechera "divaricarpa"*	South Central Rockies Forest East	h	AY165348				
Arab1839	*Boechera "divaricarpa"*	Great basin Shrub Steppe central East	c	AY165314				
Arab1840	*Boechera falcatoria*	Great Basin Shrub Steppe East	ee	AY165338				
Arab1842	*Boechera falcifructa*	Great basin Shrub Steppe central East	pm	EU275019			GQ166360	
Arab1843	*Boechera fendleri*	Arizona Mountains Forest	g	AY165347			GQ166361	HE590007
Arab1844	*Boechera fendleri*	Chihuahua Desert	g	AY165347			GQ166362	HE590008
Arab1845	*Boechera fendleri*	Wasatch/Uinta Montane Forest	g	AY165347				
Arab1846	*Boechera fendleri*	Chihuahua Desert	g	AY165347				
Arab1847	*Boechera fendleri*						GQ166363	HE590009
Arab1848	*Boechera fendleri*	Western Short Grasslands	g	AY165347				
Arab1849	*Boechera fernaldiana*	Snake/Columbia Shrub Steppe	pd	EU275020				
Arab1850	*Boechera fernaldiana*	Great Basin Shrub Steppe central West	op	EU274869				
Arab1851	*Boechera fernaldiana*	Great Basin Shrub Steppe central West	pc	EU275021				

Accession	Country	State/ Province	County	Herbarium	Herbarium number	Collector	Coll. number	X coordi-nate	Y coordi-nate
Arab1828	USA	California	Plumas County	MO	3835569	M. S. Taylor	4582	-120.98	40.12
Arab1829	USA	Colorado	Montose County	MO	5879799	M. D. Windham	3046	-107.54	38.45
Arab1830	USA	Colorado	Gunnison County	MO	5762584	M. D. Windham	2102	-106.81	38.52
Arab1832	USA	Washington	Whitman County	MO	1198505	Lincoln Constance, Reed Rollins	1101	-117.68	47.18
Arab1834	USA	Nevada	Humboldt County	MO	5188405	M. D. Windham	99-223	-117.55	41.66
Arab1837	USA	Wyoming	Lincoln County	MO		Edwin B. Payson, George M. Armstrong	3367	-110.93	42.72
Arab1838	USA	Wyoming	Park County	MO	1124218	Louis O. Williams, Rua P. Williams	3683	-109.50	44.00
Arab1839	USA	Nevada	Elko County	MO	715443	P. B. Kennedy	4485	-115.70	41.70
Arab1840	USA	Utah	Box Elder County	MO	5720857	M. D. Windham	99-100	-113.78	41.86
Arab1842	USA	Nevada	Elko County	MO	5188404	M. D. Windham	99-229	-114.79	41.59
Arab1843	USA	Arizona	Apache County	MO	5879789	M. D. Windham	3073	-109.10	36.47
Arab1844	USA	New Mexico	Catron County						
Arab1845	USA	Utah	Washington County	MO	240322	S. Welsh, K. Taylor, F. Peabody	13175	-113.28	37.53
Arab1846	USA	New Mexico	Catron County						
Arab1847	USA	Utah	San Juan County	MO	5879796	M. D. Windham, L. Allphin, J. Frnsworth	3077	-109.76	37.67
Arab1848	USA	New Mexico	San Miguel County						
Arab1849	USA	Nevada	Elko County	MO	3397047	Reed C. and Kathryn W. Rollins	79273	-114.67	41.98
Arab1850	USA	Nevada	Nye County	MO	3398901	Reed C. and Kathryn W. Rollins	79224	-117.35	38.96
Arab1851	USA	Nevada	Nye County	MO	5720854	M. D. Windham	99-210	-117.35	38.99

Accession	Taxon	wwf_ecoregion	ITS-type	ITS accession number	trnL/F type	trnLF accession numbers	***At2g25920* orthologue**	***At3g18900* orthologue**
Arab1852	*Boechera fernaldiana*	Great Basin Shrub Steppe central West	il	EU274866			GQ166364	
Arab1853	*Boechera fernaldiana var. vivariensis*	Colorado Plateau Shrub Steppe East	f	AY165346				
Arab1854	*Boechera fernaldiana var. vivariensis*	Colorado Plateau Shrub Steppe East	pn	EU275022				
Arab1856	*Boechera formosa*	Colorado Plateau Shrub Steppe East	lc	EU274877			GQ166365	HE590010
Arab1857	*Boechera formosa*	Colorado Plateau Shrub Steppe West	po	EU275023				
Arab1858	*Boechera formosa*	Colorado Plateau Shrub Steppe West	po	EU275023			GQ166366	HE590011
Arab1859	*Boechera formosa*						GQ166367	HE590012
Arab1860	*Boechera formosa*	Colorado Plateau Shrub Steppe East	pp	EU275024			GQ166368	HE590013
Arab1861	*Boechera formosa*	Colorado Plateau Shrub Steppe East	ad	AY165328			GQ166369	HE590014
Arab1862	*Boechera formosa*	Colorado Plateau Shrub Steppe East	pq	EU275025			GQ166370	HE590015
Arab1863	*Boechera glareosa*	Wasatch/Uinta Montane Forest	g	AY165347			GQ166371	
Arab1865	*Boechera glaucovalvula*	Sonora Desert	nh	EU274888				
Arab1867	*Boechera glaucovalvula*	Sierra Nevada South	nh	EU274888				
Arab1868	*Boechera gracilipes*	Arizona Mountains Forest	f	AY165346				
Arab1870	*Boechera gracilipes*	Great Basin Shrub Steppe East	f	AY165346			GQ166372	
Arab1871	*Boechera gracilipes*	Colorado Plateau Shrub Steppe West	f	AY165346			GQ166373	
Arab1872	*Boechera gunnisonana*	Colorado Plateau Shrub Steppe East	pk	EU275026				
Arab1873	*Boechera hastatula*						GQ166374	
Arab1875	*Boechera holboellii*	Kalaalit Nunaat Low Arctic Tundra	h	AY165348				
Arab1876	*Boechera holboellii*	Kalaalit Nunaat Low Arctic Tundra	h	AY165348				
Arab1881	*Boechera howellii*	Central and Southern Cascades Forest	pr	EU275027				

Accession	Country	State/ Province	County	Herbarium	Herbarium number	Collector	Coll. number	X coordi-nate	Y coordi-nate
Arab1852	USA	Nevada	Nye County	MO	5720854	M. D. Windham	99-210	-117.35	38.99
Arab1853	USA	Utah	Uintah County	MO	5188413	M. D. Windham	99-079	-109.17	40.35
Arab1854	USA	Colorado	Moffat County	MO	1738661	C. L. Porter		-108.88	40.47
Arab1856	USA	Utah	San Juan County	MO	5879751	M. D. Windham, M. J. Windham, L. Funes	2994	-109.66	37.24
Arab1857	USA	Utah	Duchesne County	MO	5598887	M. D. Windham	2402	-110.49	40.17
Arab1858	USA	Utah	Carbon County	MO	4865514	M. D. Windham	3363	-110.64	39.54
Arab1859	USA	Wyoming	Sweetwater County	MO	5879752	M. D. Windham	3031	-109.52	41.55
Arab1860	USA	Colorado	Montrose County	MO	4865466	M. D. Windham, M. J. Windham, L. Funes	2991	-108.60	38.21
Arab1861	USA	Colorado	Mesa County	MO	4865470	M. D. Windham, L. Funes	2981	-109.02	39.17
Arab1862	USA	Utah	Grand County	MO	5577782	M. D. Windham, M. K. Windham, L. Fawcett	99-051	-109.38	38.70
Arab1863	USA	Utah	Summit County	MO	keine MO Nummer	M. D. Windham	99-251	-110.16	40.94
Arab1865	USA	California	Riverside County	MO	1032395	Philip A. Munz, C. L. Hitchcock	12231	-115.80	33.73
Arab1867	USA	California	Mono County	MO	3439716	James D. Morefield, Douglas McCarty	3461	-118.93	37.75
Arab1868	USA	Arizona		MO	3398902			-112.21	36.71
Arab1870	USA	Utah	Millard County	MO	5720943	M. D. Windham	99-134	-112.26	39.34
Arab1871	USA	Arizona	Mohave County	MO	5762608	M. D. Windham	99-193	-113.14	36.38
Arab1872	USA	Colorado	Gunnison County	MO	3398881	Reed C. Rollins and Kathryn W. Rollins	7973	-106.92	38.54
Arab1873	USA	Oregon	Baker County	MO	5720851	M. E. Vining	A-29	-116.66	45.43
Arab1875	Greenland			MO	3691687	Minna Moller	1692	-50.46	69.66
Arab1876	Greenland	West Greenland		MO	5577479	Bant Fradskild, Vilhelm Daigaard	87553	-50.55	67.95
Arab1881	USA	Oregon	Klamath County	MO	1206165	J. William Thompson	12246	-122.11	42.93

**Accession**	**Taxon**	**wwf_ecoregion**	**ITS-type**	**ITS accession number**	**trnL/F type**	**trnLF accession numbers**	***At2g25920 orthologue***	***At3g18900 orthologue***
Arab1882	*Boechera lasiocarpa*						GQ166375	
Arab1885	*Boechera inyoensis*	Great Basin Shrub Steppe West	i	AY165317				
Arab1886	*Boechera inyoensis*						GQ166376	
Arab1887	*Boechera johnstonii*	California Montane Chaparral and Woodlands	ev	AY165341			GQ166377	
Arab1888	*Boechera koehleri*	Klamath Siskiyou Forests	sa	EU275060				
Arab1890	*Boechera koehleri*	Klamath Siskiyou Forests	mm	EU274885				
Arab1891	*Boechera koehleri*	Klamath Siskiyou Forests	kn	EU274872				
Arab1892	*Boechera lasiocarpa*	Great Basin Shrub Steppe East	ad	AY165328				
Arab1893	*Boechera lemmonii*	Great Basin Shrub Steppe West	ps	EU275028			GQ166378	HE590016
Arab1894	*Boechera lemmonii*	Sierra Nevada North	pt	EU275029			GQ166379	
Arab1895	*Boechera lignifera*	Colorado Plateau Shrub Steppe East	ee	AY165338			GQ166380	HE590017
Arab1896	*Boechera lignifera*						GQ166381	HE590018
Arab1897	*Boechera lignifera*	Wasatch/Uinta Montane Forest	ad	AY165328			GQ166382	
Arab1898	*Boechera lignifera*	Arizona Mountains Forest	pu	EU275030				HE590019
Arab1899	*Boechera lignifera*	Wyoming Basin Shrub Steppe	pv	EU275031				
Arab1900	*Boechera lincolnensis*	Mojave Desert	pw	EU275032			GQ166383	HE590020
Arab1901	*Boechera lincolnensis*	Mojave Desert	ev	AY165341			GQ166384	
Arab1902	*Boechera nevadensis*	Mojave Desert	px	EU275033			GQ166385	
Arab1903	*Boechera lyallii*	Central and Southern Cascades Forest	ad	AY165328				
Arab1905	*Boechera lyallii*	Cascade Mountains Leeward Forests	py	EU275034			GQ166386	

Accession	Country	State/ Province	County	Herbarium	Herbarium number	Collector	Coll. number	X coordi-nate	Y coordi-nate
Arab1882	USA	Utah	Cache County	MO	5879782	M. D. Windham, L. Allphin	2195b	-111.78	41.39
Arab1885	USA	California	Inyo County	MO	5577983	Mary DeDecker	2718	-118.19	36.80
Arab1886	USA	California	Inyo County	MO	5582506	Mary DeDecker	5809	-118.08	37.07
Arab1887	USA	California	Riverside County	MO	5879785	M. D. Windham	2954	-116.60	33.63
Arab1888	USA	Oregon	Douglas County	MO	1080832	J. William Thompson	10157	-123.34	43.21
Arab1890	USA	Oregon		MO	924653	L. F. Henderson	5720	-123.64	42.06
Arab1891	USA	Oregon	Douglas County	MO	1289309	Lincoln Constance, Reed C. Rollins	2954	-123.34	43.21
Arab1892	USA	Utah	Cache County	MO	3397057	Reed C. and Kathryn W. Rollins	79316	-111.79	41.74
Arab1893	USA	California	Mono County	MO	5577779	M. D. Windham, M. Beilstein	2483	-119.32	38.44
Arab1894	USA	California	Tuolumne County						
Arab1895	USA	Arizona	Apache County	MO	4780147	M. D. Windham, D. Roth	2320	-109.43	35.88
Arab1896	USA	Wyoming	Sweetwater County	MO	5598889	M. D. Windham	00-081	-109.22	41.01
Arab1897	USA	Utah	Summit County	MO	5720831	M. D. Windham	99-076	-111.41	40.96
Arab1898	USA	New Mexico	McKinley County	MO	4780150	M. D. Windham	3370	-109.01	35.92
Arab1899	USA	Wyoming	Sweetwater County	MO	5879803	M. D. Windham	3032	-109.52	41.55
Arab1900	USA	California	San Bernardino County	MO	4865424	M. D. Windham	2956	-116.46	34.13
Arab1901	USA	Nevada	Nye County	MO	4924766	Ann Pinzl	11205	-116.77	36.93
Arab1902	USA	Nevada	Clark County	MO	5762591	M. D. Windham	2159	-115.65	36.29
Arab1903	USA	Washington	Yakima County	MO	1202111	J. William Thompson	15139	-121.51	46.87
Arab1905	USA	Washington	Ohnlan County	MO	1081616	J. William Thompson	10789	-120.75	47.43

Accession	Taxon	wwf_ecoregion	ITS-type	ITS accession number	trnL/F type	trnLF accession numbers	*At2g25920 orthologue*	*At3g18900 orthologue*
Arab1908	*Boechera microphylla*	South Central Rockies Forest West	h	AY165348			GQ166387	HE590021
Arab1909	*Boechera microphylla*	Great Basin Shrub Steppe East	h	AY165348			GQ166388	
Arab1911	*Boechera oxylobula*	Colorado Plateau Shrub Steppe East	hc	AY165408				
Arab1912	*Boechera oxylobula*	Colorado Plateau Shrub Steppe East	hc	AY165408			GQ166389	
Arab1913	*Boechera oxylobula*	Colorado Rockies Forest	pz	EU275035			GQ166390	
Arab1914	*Boechera oxylobula*	Colorado Rockies Forest	ra	EU275036				
Arab1915	*Boechera oxylobula*						GQ166391	
Arab1916	*Boechera pallidifolia*	Colorado Plateau Shrub Steppe East	ad	AY165328			GQ166392	
Arab1917	*Boechera pallidifolia*	Wasatch/Uinta Montane Forest	ad	AY165328			GQ166393	HE590022
Arab1918	*Boechera pallidifolia*	Colorado Plateau Shrub Steppe West	ad	AY165328				HE590023
Arab1919	*Boechera pallidifolia*	Colorado Plateau Shrub Steppe East	ad	AY165328			GQ166394	HE590024
Arab1920	*Boechera pallidifolia*	Colorado Plateau Shrub Steppe East	ad	AY165328				HE590025
Arab1921	*Boechera pallidifolia*	Wyoming Basin Shrub Steppe	rb	EU275037			GQ166395	
Arab1924	*Boechera paupercula*	Great Basin Shrub Steppe West	v	AY165353				
Arab1925	*Boechera paupercula*	South Central Rockies Forest West	rc	EU275038				
Arab1926	*Boechera pendulina*						GQ166396	
Arab1927	*Boechera pendulina*	Great Basin Shrub Steppe central East	g	AY165347			GQ166397	HE590026
Arab1928	*Boechera pendulina*	Arizona Mountains Forest	rd	EU275039				HE590027
Arab1929	*Boechera pendulina*	Colorado Plateau Shrub Steppe West	ev	AY165341				HE590028
Arab1932	*Boechera perennans*	Mojave Desert	ev	AY165341			GQ166398	
Arab1933	*Boechera perennans*	Mojave Desert	re	EU275040				HE590029

Accession	Country	State/ Province	County	Herbarium	Herbarium number	Collector	Coll. number	X coordi-nate	Y coordi-nate
Arab1908	USA	Idaho	Elmore County	MO	5598937	M. D. Windham	2376	-115.47	43.77
Arab1909	USA	Utah	Millard County	MO	5188401	M. D. Windham	99-133	-112.26	39.34
Arab1911	USA	Colorado	Gunnison County	MO	3398764	Reed C. Rollins and Kathryn W. Rollins	7953	-106.72	38.50
Arab1912	USA	Colorado	Gunnison County	MO	3472806	Reed C. Rollins and Kathryn W. Rollins	8375	-107.30	38.45
Arab1913	USA			MO	5720869			-106.00	39.16
Arab1914	USA	Colorado	Hinsdale County	MO	5762588	M. D. Windham	2098	-107.35	38.01
Arab1915	USA	Colorado	Montrose County	MO	4865461	M. D. Windham	3052	-107.54	36.45
Arab1916	USA	New Mexico	Taos County	MO	4865439	M. D. Windham	3059	-105.68	36.67
Arab1917	USA	Utah	Wayne County	MO	5188414	M. D. Windham, C.J. Hansen, M. K. Windham	97-057	-111.48	38.28
Arab1918	USA	Utah	Kane County	MO	5563087	M. D. Windham	2395	-112.18	37.30
Arab1919	USA	Utah	Uintah County	MO	5762583	M. D. Windham	2643	-109.54	39.75
Arab1920	USA	Colorado	Gunnison County	MO	5762601	M. D. Windham	2103b	-106.81	38.52
Arab1921	USA	Colorado	Moffat County	MO	5690788	M. D. Windham	00-089	-108.74	40.84
Arab1924	USA	California	Mono County	MO	3500067	James D. Morefield, Timothy S. Ross	4709	-118.22	37.64
Arab1925	USA	Wyoming	Teton	MO	3178471		4288	-110.70	43.83
Arab1926	USA	Utah	Kane County	MO	5598884	M. D. Windham	2023	-112.50	37.50
Arab1927	USA	Nevada	White Pine County	MO	5598876	M. D. Windham	2050	-114.69	39.24
Arab1928	USA	Arizona	Coconino County	MO	5598888	M. D. Windham	2349	-112.15	36.73
Arab1929	USA	Utah	Kane County	MO	5598883	M. D. Windham	2025	-112.56	37.49
Arab1932	USA	Utah	Washington County	MO	5879788	M. D. Windham	2971	-113.85	37.01
Arab1933	USA	California	San Brnardino County	MO	5879794	M. D. Windham	2965	-115.88	35.78

Accession	Taxon	wwf_ecoregion	ITS-type	ITS accession number	trnL/F type	trnLF accession numbers	*At2g25920 orthologue*	*At3g18900 orthologue*
Arab1934	*Boechera perennans*	Chihuahua Desert	rf	EU275041			GQ166399	HE590030
Arab1936	*Boechera pinzliae*	Great Basin Shrub Steppe West	rg	EU275042				
Arab1938	*Boechera platysperma*	Sierra Nevada South	rh	EU275043				
Arab1941	*Boechera polyantha*	Cascade Mountains Leeward Forests	h	AY165348				
Arab1942	*Boechera polyantha*	Palouse Grasslands	ri	EU275044				
Arab1944	*Boechera polyantha*	North Central Rockies Forest	h	AY165348				
Arab1945	*Boechera polyantha*	Okanogan Forest	h	AY165348				
Arab1946	*Boechera puberula*	Snake/Columbia Shrub Steppe	rj	EU275045			GQ166400	
Arab1947	*Boechera puberula*	Great Basin Shrub Steppe West	rk	EU275046				
Arab1948	*Boechera puberula*	Snake/Columbia Shrub Steppe	ol	EU274901				
Arab1949	*Boechera pulchra*	California Montane Chaparral and Woodlands	rl	EU275047			GQ166401	
Arab1950	*Boechera pulchra*	California Montane Chaparral and Woodlands	rl	EU275047			GQ166402	HE590031
Arab1951	*Boechera pusilla*	South Central Rockies Forest East	f	AY165346				
Arab1952	*Boechera pusilla*	South Central Rockies Forest East	f	AY165346				
Arab1956	*Boechera rectissima*	Sierra Nevada South	z	AY165325				
Arab1957	*Boechera rectissima*	Klamath Siskiyou Forests	z	AY165325				
Arab1959	*Boechera repanda*	Great basin Shrub Steppe West	rm	EU275048				
Arab1960	*Boechera repanda*						GQ166403	
Arab1961	*Boechera repanda*	Mojave Desert	rn	EU275049				HE590032
Arab1962	*Boechera rigidissima*	Klamath Siskiyou Forests	kv	EU274876			GQ166404	HE590033
Arab1963	*Boechera schistacea*	Colorado Plateau Shrub Steppe West	ro	EU275050				

Accession	Country	State/ Province	County	Herbarium	Herbarium number	Collector	Coll. number	X coordi-nate	Y coordi-nate
Arab1934	USA	Arizona	Pima County	MO	5879760	M. D. Windham	2933	-110.76	31.80
Arab1936	USA	Nevada	Esmeralda County	MO	3497189	James D. Morefield	4656	-118.33	37.86
Arab1938	USA	California	Tuolumne County	MO	1192007	R. F. Hoover	2514	-120.13	38.09
Arab1941	USA	Washington	Okanogan County	MO	1012653	J. William Thompson	7130	-119.43	48.70
Arab1942	USA	Washington	Spokane County	MO	952200	Wilhelm Sucksdorf		-118.34	47.90
Arab1944	USA	Montana	Flathead County	MO	1249363	H. T. Rogers, J. M. Rogers	899	-114.17	48.38
Arab1945	USA	Washington	Ferry County	MO	1259582	H. T. Rogers	336	-118.13	48.71
Arab1946	USA	Nevada	Washoe County	MO	3391137	Reed C. and Kathryn W. Rollins	81219	-119.85	41.59
Arab1947	USA	Nevada	Nye County	MO	5690753	M. D. Windham	99-115	-117.52	38.96
Arab1948	USA	Nevada	Elko County	MO	5188398	M. D. Windham	99-101	-114.76	41.74
Arab1949	USA	California	Los Angeles County	MO	5613050	Steve Boyd, Lauren Raz	9428	-118.66	34.63
Arab1950	USA	California	Los Angeles County	MO	4917151	Steve Boyd, Timothy S. Ross	9233	-118.48	34.70
Arab1951	USA	Wyoming	Fremont County	MO	5720935	M. D. Windham	99-140	-108.87	42.45
Arab1952	USA	Wyoming	Fremont County	MO	3862055	R. Dorn	5092	-108.86	42.45
Arab1956	USA	California	Maders County	MO	1150103	L. Constance	2393	-119.43	37.24
Arab1957	USA	California	Siskiyou County						
Arab1959	USA	California	Alpine County						
Arab1960	USA	California	Fresno County	MO	4996393	Barbara Ertter, Jane Shewock et al.	9204	-118.77	36.84
Arab1961	USA	California	Tulare County						
Arab1962	USA	California	Trinity County	MO	5720933	B. A. Smith	1073	-123.22	40.95
Arab1963	USA	Utah	Garfield County	MO	3472790	Reed C. and Kathryn W. Rollins with Aileen G. Roads	83161	-112.43	37.82

Accession	Taxon	wwf_ecoregion	ITS-type	ITS accession number	trnL/F type	trnLF accession numbers	*At2g25920 orthologue*	*At3g18900 orthologue*
Arab1964	*Boechera schistacea*	Great Basin Shrub Steppe central West					GQ166405	
Arab1965	*Boechera schistacea*	Great Basin Shrub Steppe central West	rp	EU275051			GQ166406	HE590034
Arab1966	*Boechera shockleyi*	Great Basin Shrub Steppe East	mo	EU274886			GQ166407	HE590035
Arab1968	*Boechera sparsiflora*	Colorado Rockies Forest	u	AY165324			GQ166408	
Arab1969	*Boechera sparsiflora*	Colorado Rockies Forest	rr	EU275052			GQ166409	HE590036
Arab1970	*Boechera stricta*	Sierra Nevada	dj	AY165403				
Arab1971	*Boechera stricta*	Colorado Plateau Shrub Steppe	rs	EU275053			GQ166410	HE590037
Arab1972	*Boechera stricta*	Great Basin Shrub Steppe East	r	AY165321				HE590038
Arab1973	*Boechera stricta*	Colorado Rockies Forest	rt	EU275054				HE590039
Arab1974	*Boechera sparsiflora*	Great Basin Shrub Steppe central West	h	AY165348				
Arab1975	*Boechera sparsiflora*	South Central Rockies Forest West	h	AY165348				HE590040
Arab1976	*Boechera subpinnatifida*	Great Basin Shrub Steppe West	rj	EU275045				
Arab1977	*Boechera subpinnatifida*	Klamath Siskiyou Forests	ac	AY165327				
Arab1979	*Boechera subpinnatifida*	Klamath Siskiyou Forests	ru	EU275055				
Arab1980	*Boechera suffrutescens*	Sierra Nevada North	lk	EU274879				HE590041
Arab1982	*Boechera suffrutescens*	South central Rockies Forest West	me	EU274939			GQ166411	
Arab1984	*Boechera suffrutescens*	Blue Mountain Forest	me	EU274939			GQ166412	
Arab1985	*Boechera texana*	Chihuahua Desert	rv	EU275056				
Arab1987	*Boechera texana*	Chihuahua Desert	rw	EU275057				
Arab1990	*Boechera texana*						GQ166413	
Arab1991	*Boechera williamsii*	Wyoming Basin Shrub Steppe	rx	EU275058				

Accession	Country	State/ Province	County	Herbarium	Herbarium number	Collector	Coll. number	X coordi-nate	Y coordi-nate
Arab1964	USA	Nevada	Nye County	MO	5720932	M. D. Windham, M. K. Windham	99-284	-117.33	38.95
Arab1965	USA	Nevada	Nye County	MO	4865458	M. D. Windham	3104	-117.32	38.95
Arab1966	USA	Utah	Millard County	MO	5188400	M. D. Windham	99-122	-113.62	38.92
Arab1968	USA	Colorado	Saguache County	MO	3446232	Reed C. Rollins	8354	-105.94	38.24
Arab1969	USA		Gilpin County						
Arab1970	USA	California	Inyo County	MO	5690795	M. D. Windham	2572	-118.25	36.57
Arab1971	USA	Utah	Grand County	MO	5879770	M. D. Windham	3167	-109.22	38.50
Arab1972	USA	Nevada	White Pine County	MO	5577321	M. D. Windham, E. Rickart	2165	-114.30	39.01
Arab1973	USA	Colorado	San Miguel County	MO	5879772	M. D. Windham	3174	-107.89	37.81
Arab1974	USA	Nevada	Lander County	MO	5188433	M. D. Windham	99-213	-116.86	40.43
Arab1975	USA	Idaho	Boise County	MO	5762614	M. D. Windham	2379	-115.66	43.69
Arab1976	USA	Nevada	Humboldt County	MO	5879787	M. D. Windham	3127	-117.54	41.66
Arab1977	USA	Oregon	Jackson County						
Arab1979	USA	California	Tehama County	MO	5087944	Vernon H. Oswald, Lowell Ahart	7029	-122.90	40.25
Arab1980	USA	California	El Dorado County	MO	5690755	M. D. Windham	2579	-120.09	38.82
Arab1982	USA	Idaho	Blaine County	MO	5188408	M. D. Windham	99-299	-114.71	43.87
Arab1984	USA	Oregon	Baker County	MO	5720926	M.E. Vining	A-31	-116.72	45.37
Arab1985	USA	Texas	Presidio County	MO	860281	Herbert C. Hanson	640	-104.01	30.30
Arab1987	USA	Texas		MO	995489	Marcus E. Jones	25824	-105.35	31.17
Arab1990	USA	New Mexico	Torrance County	MO	2871434	W. L. Wagner, D. Sabo	3056	-105.53	34.66
Arab1991	USA	Wyoming	Fremont County	MO	3391066	Reed C. and Kathryn W. Rollins	81369	-108.95	42.34

**Accession**	**Taxon**	**wwf_ecoregion**	**ITS-type**	**ITS accession number**	**trnL/F type**	**trnLF accession numbers**	***At2g25920 orthologue***	***At3g18900 orthologue***
Arab1992	*Boechera falcata*	East Siberian Taiga	ry	EU275059				
Arab1993	*Borodinia tilingii*		rz	EU274864				
ES044	*Borodinia baicalensis*		rz	EU274865				
BH115_442	*Boechera "holboellii"*						GQ166311	
ES001	*Boechera stricta*		he	EU274893				HE590042
ES002	*Boechera stricta*		he	EU274893				HE590043
ES003	*Boechera stricta*	Colorado Rockies Forest						HE590044
ES004	*Boechera stricta*		ov	EU274993				HE590045
ES005	*Boechera stricta*	Colorado Rockies Forest						HE590046
ES007	*Boechera stricta*		nv	EU274994				HE590047
ES010	*Boechera stricta*	South Central Rockies Forest West						HE590048
ES013	*Boechera stricta*							HE590049
ES015	*Boechera lyallii*						GQ166312	HE590050
ES018	*Boechera stricta*	Colorado Plateau Shrub Steppe						HE590051
ES027	*Boechera "divaricarpa"*		ow	EU274995				
ES030	*Boechera stricta*		nw	EU274996				
ES031	*Boechera "divaricarpa"*		nx	EU274997				
ES033	*Boechera "divaricarpa"*		ny	EU274998				
ES037	*Boechera stricta*		nz	EU274999				
ES038	*Boechera stricta*	Wasatch/Uinta Montane Forest	c		ah		GQ166313	HE590052
ES040	*Boechera stricta*	Colorado Plateau Shrub Steppe	l	AY165319	dd	DQ013047	GQ166314	
ES042	*Boechera stricta*		oa	EU275000				
ES044	*Boechera stricta*							HE590053
ES046	*Boechera "divaricarpa"*		oc	EU275001				
ES047	*Boechera "divaricarpa"*		od	EU275002				
ES048	*Boechera stricta*		ox	EU275003				
ES050	*Boechera stricta*		oy	EU275004				
ES053	*Boechera stricta*		oi	EU275005				

Accession	Country	State/ Province	County	Herbarium	Herbarium number	Collector	Coll. number	X coordi-nate	Y coordi-nate
Arab1992	Russia	Siberia	Ayano-Mayskiy District	MO	5739836	S. Kharkevich, T. Buch	274	137.43	57.58
Arab1993	Russia	Siberia	Ayano-Mayskiy District	MO	5100619	S. Kharkevich, I. Vyshin	277b	135.11	48.42
ES044	Russia								
BH115_442	USA	Montana	Ravalli						
ES001	USA	Colorado	Gunnison	HEID	500201			-106.6	38.57
ES002	USA	Colorado	Gunnison	HEID	500201			-106.6	38.57
ES003	USA	Colorado	Gunnison	HEID	500201			-106.6	38.56
ES004	USA	Colorado	Gunnison	HEID	500201			-106.6	38.57
ES005	USA	Colorado	Gunnison	HEID	500201			-106.83	38.65
ES007	USA	Montana	Beaverhead	HEID	500207			-112.86	45.68
ES010	USA	Montana	Beaverhead	HEID	500210			-112.86	45.68
ES013	USA	California	Tulare	HEID	500213			-118.21	36.43
ES015	USA	Idaho	Idaho	HEID	500215			-114.71	46.43
ES018	USA	Colorado	Gunnison	HEID	500218			-106.83	38,65
ES027	USA	California	Alpine	HEID	500227			-119.93	38.5
ES030	USA	California	Mono	HEID	500230			-119.58	38.3
ES031	USA	California	Mono	HEID	500231			-119.58	38.3
ES033	USA	California	Alpine	HEID	500233			-119.92	38.7
ES037	USA	Oregon	Harney	HEID	500237			-118.57	42.7
ES038	USA	Utah	Cane	HEID	500238			-112.7	37.5
ES040	USA	Utah	San Juan	HEID	500240			-110.8	38.8
ES042	USA	Colorado	Gunnison	HEID	500242			-106.83	38.65
ES044	USA	Montana	Carbon County	HEID	500244				
ES046	USA	Montana	Beaverhead	HEID	500246			-112.47	44.55
ES047	USA	Montana	Beaverhead	HEID	500247			-112.62	44.55
ES048	USA	Montana	Beaverhead	HEID	500248			-112.65	44.55
ES050	USA	Colorado	Park County	HEID	500250			-106.05	39.35
ES053	USA	Colorado	Clear Creek	HEID	500253			-105.6	39.68

Accession	Taxon	wwf_ecoregion	ITS-type	ITS accession number	trnL/F type	trnLF accession numbers	*At2g25920 orthologue*	*At3g18900 orthologue*
ES072	*Boechera stricta*		of	EU275006				
ES075	*Boechera crandallii*		hg	EU275007				HE590054
ES077	*Boechera gracilenta*	Colorado Plateau Shrub Steppe West	ad		do	DQ013056	GQ166315	HE590055
ES079	*Boechera pygmaea*		oz	EU275008			GQ166316	HE590056
ES080	*Boechera lemmonii*	Colorado Rockies Forest			dm	DQ013054	GQ166317	HE590057
ES081	*Boechera fendleri*	Arizona Mountains Forest						HE590058
ES082	*Boechera crandallii*							HE590059
ES083	*Boechera pendulina*	Wyoming Basin Shrub Steppe			ci	AY257778	GQ166318	HE590060
ES085	*Boechera fendleri*	Colorado Rockies Forest South						HE590061
ES089	*Boechera lasiocarpa*	Wasatch/Uinta Montane Forest			d	AY257695	GQ166319	
ES090	*Boechera suffrutescens*	Blue Mountain Forest			db	DQ013046	GQ166320	
ES092	*Boechera "holboellii"*	South Central Rockies Forest West			dn	DQ013055	GQ166321	
ES093	*Boechera "holboellii"*	South Central Rockies Forest West	h		ci	AY257778	GQ166322	
ES095	*Boechera "holboellii"*	South Central Rockies Forest West	bt		ci	AY257778	GQ166323	
ES096	*Boechera "holboellii"*	South Central Rockies Forest West	h		dl	DQ013053	GQ166324	
ES097	*Boechera "holboellii"*	South Central Rockies Forest West	h		ci	AY257778	GQ166325	HE590062
TS206	*Boechera "holboellii"*						HE585211	
TS287	*Boechera "holboellii"*						HE585212	
	Anelsonia eurycarpa			DQ452059				
	Boechera serotina			DQ452062				
	Capsella rubella			AJ232913				
	Cusickiella douglasii			AF146515				
	Cusickiella quadricostata			DQ452066				
	Cusickiella quadricostata			AF146514				
	Halimolobos perplexa var. lemhiensis			AJ232927				
	Halimolobos perplexa var. perplexa			AJ232926				
	Pennellia longifolia			AF307627				
	Pennellia micrantha			AF307629				

Accession	Country	State/ Province	County	Herbarium	Herbarium number	Collector	Coll. number	X coordi-nate	Y coordi-nate
ES072	USA	Idaho	Blaine	HEID	500272			-114.7	43.25
ES075	USA	Colorado	Gunnison	HEID	500275			-106.7	38.5
ES077	USA	Utah	Wayne	HEID	500277			-111.48	38.26
ES079	USA	California	Tulare	HEID	500279			-118.23	36.43
ES080	USA	Colorado	Park	HEID	500280			-106.01	39.35
ES081	USA	New Mexiko	Grant	HEID	500281			-107.96	33.03
ES082	USA	Colorado	Gunnison	HEID	500282			-107.68	38.56
ES083	USA	Colorado	Moffat	HEID	500283			-108.7	40.85
ES085	USA	New Mexiko	Santa Fe	HEID	500285			-105.76	35.58
ES089	USA	Utah	Salt Lake	HEID	500289			-111.75	40.78
ES090	USA	Oregon	Baker	HEID	500290			-116.73	45.36
ES092	USA	Idaho	Custer	HEID	500292			-114.25	44.81
ES093	USA	Idaho	Lemhi	HEID	500293			-114.58	45.35
ES095	USA	Idaho	Lemhi	HEID	500295			-114.33	45.3
ES096	USA	Idaho	Lemhi	HEID	500296			-114.28	45.25
ES097	USA	Idaho	Custer	HEID	500297			-114.51	44.61
TS206	USA	Montana	Ravalli			obtained from T. F. Sharbel, T. Mitchell-Olds			
TS287	USA	California	Stoery			obtained from T. F. Sharbel, T. Mitchell-Olds			
