# Supplementary material for: A Continental-Wide Perspective: The Genepool of Nuclear Encoded Ribosomal DNA and Single-Copy Gene Sequences in North American Boechera (Brassicaceae)
Source: PLoS One. 2012 May 14;7(5):e36491. doi: 10.1371/journal.pone.0036491 (PMC3351400; doi:10.1371/journal.pone.0036491)
Supplement: Table S4 — ITS types which were found to have ambiguous sites and were hence assumed to be found in hybrid individuals; given are the accession number, taxon as on herbarium voucher, ITS type, chloroplast DNA lineage according to [8] and [7] and the main representative taxon which shared this cpDNA type; also it is indicated if hybrids of this taxon have been identified previously according to [14]; ITS types from Table S4 are shaded gray in the ITS phylogeny (Figure S1). (DOC) [file pone.0036491.s004.doc]

| **Accession Number** | **Taxon** | **ambgs ITS type** | **cpDNA lineage (type)** | **cpDNA type mainly found in** | **main cpDNA type in taxon given on voucher** | **described as apomict in FNA** |
| --- | --- | --- | --- | --- | --- | --- |
| Arab 1322 | *Boechera lyallii* | iy | 2 (DV) | derived from S9 (mainly stricta) | mainly lineage II | can be apomictic |
| Arab 1346 | *Boechera microphylla* | ka | 1 (DX) | B. microphylla; with FO in S20 | S20 and derived haplotypes in lin I. and CI in lin. III | two apomict hybrids known |
| Arab 1353 | *Boechera microphylla* | kb | 3 (DZ) | see Kiefer et al. 2009; derived from S14 | S20 and derived haplotypes in lin I. and CI in lin. III | two apomict hybrids known |
| Arab 1382 | *Boechera lemmonii* | kl | 3 (CH) | see Kiefer et al. 2009; derived from CI | mainly CI and derived and a group with lin. I haplotype M | indication for three apomictic hybrids |
| Arab 1384 | *Boechera lemmonii* | km | 3 (CH) | see Kiefer et al. 2009; derived from CI | mainly CI and derived and a group with lin. I haplotype M | indication for three apomictic hybrids |
| Arab 1445 | *Boechera perennans* | ku | 3 (EQ) |  | mainly S16 | B. gracilenta as apomict has been excluded from B. perennans |
| Arab 1449 | *Boechera howellii* | kw | 3 (ER) | B. platysperma | own lineage derived from S14 which includes ER | no hybrid described |
| Arab 1456 | *Boechera puberula* | kx | 1 (S) | B. puberula | mainly S | serves as parent for apomictic hybrids |
| Arab 1535 | *Boechera crandallii* | ln | 3 (CI) | see Kiefer et al. 2009; among them B. crandallii | mainly CI or related haplotypes | hybridises with B. pallidifolia |
| Arab 1539 | *Boechera crandallii* | lp | 3 (CI) | see Kiefer et al. 2009; among them B. crandallii | mainly CI or related haplotypes | hybridises with B. pallidifolia |
| Arab 1580 | *Boechera pallidifolia* | lu | 3 (CL) | see Kiefer et al. 2009; derived from CI | CL and DS | prone to hybridisation giving rise to several apomictic species |
| Arab 1646 | *Boechera sparsiflora* | mf | 2 (AS) | “B. divaricarpa” | lin I S1 and S6, lin III S16 | parent in at least four apomictic hybrids |
| Arab 1671 | *Boechera inyoensis* | mk | 3 (CI) | see Kiefer et al. 2009; among them B. inyoensis | mixed haplotypes, but mainly CI and derived | apomictic hybrid related to B. shockleyi |
| Arab 1702 | *Boechera lincolnensis* | mu | 3 (GQ) | B. pulchra | mainly lin, VI | now B. lincolnensis; no hybrids described |
| Arab 1708 | *Boechera xylopoda* | mw | 3 (GQ) | B. pulchra | mainly CI and derived | apomict included in B. xylopoda |
| Arab 1709 | *Boechera* *xylopoda* | mw | 3 (GU) | B. pulchra | mainly CI and derived | apomict included in B. xylopoda |
| Arab 1722 | *Boechera perennans* | nd | 3 (CI) | one of the main species sharing CI | mainly S16 | B. gracilenta as apomict has been excluded from B. perennans |
| Arab 1789 | *Boechera fendleri* | nm | 3 (CI) | see Kiefer et al. 2009; | mainly S16 (CI and CJ), some lin:I haplotypes | hybrids segregated today into different taxa |
| Arab0105 | *Boechera pinetorum* | hi | (C) |  |  |  |
| Arab0123 | *Boechera “divaricarpa”* | bj | 1 (B) | B. davidsonii, B. divaricarpa, “B. holboellii”, B. microphylla | AS and AH main types but B also frequent | apomictic hybrid |
| Arab0139 | *Boechera “divaricarpa”* | bn | 2 (AS) | B. stricta | AS one of the main haplotypes | apomictic hybrid |
| Arab0788 | *Boechera retrofracta* | bj | 1 (R) | see Kiefer et al. 2009; derived from S1 | BU, BY, CI and other; lineage III | hybrids with at least 12 other species |
| Arab0850 | *Boechera crandallii* | hn | 3 (CI) | see Kiefer et al. 2009; among them B. crandallii | mainly CI or related haplotypes | hybridises with B. pallidifolia |
| Arab0864 | *Boechera fendleri* | hp | Ii |  |  |  |
| Arab0876 | *Boechera fendleri* | hr | 3 (CI) | see Kiefer et al. 2009; | mainly S16 (CI and CJ), some lin:I haplotypes | hybrids segregated today into different taxa |
| Arab0877 | *Boechera fernaldiana var. stylosa* | hs | 3 (IQ) | see Kiefer et al. 2009; derived from S25 | mainly S16 (CI and CJ) and related haplotypes | no hybrid described |
| Arab0886 | *Boechera fernaldiana var. fernaldiana* | hu | 3 (CI) | see Kiefer et al. 2009; among them B. fernaldiana | mainly S16 (CI and CJ) and related haplotypes | no hybrid described |
| Arab0887 | *Boechera fernaldiana* | hv | 3 (CI) | see Kiefer et al. 2009; among them B. fernaldiana | mainly S16 (CI and CJ) and related haplotypes | no hybrid described |
| Arab0890 | *Boechera fernaldiana var. stylosa* | hw | 3 (CI) | see Kiefer et al. 2009; among them B. fernaldiana | mainly S16 (CI and CJ) and related haplotypes | no hybrid described |
| Arab0898 | *Boechera gunnisoniana* | hx | 3 (DS) | Unique to B. gunnisoniana | mainly DS, derived from S16 (CI) | no hybrid described |
| Arab0903 | *Boechera inyoensis* | hy | 3 (IN) | see Kiefer et al. 2009; derived from CI | mixed haplotypes but mainly CI and derived | apomictic hybrid related to B. shockleyi |
| Arab0904 | *Boechera koehleri* | hz | 1 (O) | mainly B. puberula | mainly O and derived | no hybrid described |
| Arab0927 | *Boechera lignifera* | ia | 1 (Y) | see Kiefer et al. 2009; included in S7; shared with B. puberula | S7 and S14 | apomictic hybrids with B. cobrensis known |
| Arab0933 | *Boechera lignifera* | ib | none |  |  |  |
| Arab0934 | *Boechera lignifera* | ic | 2 (AB) | see Kiefer et al. 2009; central haplotype; shared with B. perennans and B. shockleyi | S7 and S14 | apomictic hybrids with B. cobrensis known |
| Arab0939 | *Boechera lignifera* | id | 1 (U) | B. retrofracta, B. pinetorum, B. lignifera | S7 and S14 | apomictic hybrids with B. cobrensis known |
| Arab0955 | *Boechera pauciflora* | if | 1 (L) | B. sparsiflora; derived from S1 where B. microphylla and B. sparsiflora are included | lin I S1 and S6, lin III S16 | apomict now included in B. pauciflora |
| Arab0959 | *Boechera pauciflora* | ig | 3 (IX) | derived from S16 which also includes B. sparsiflora | lin I S1 and S6, lin III S16 | apomict now included in B. pauciflora |
| Arab0960 | *Boechera pauciflora* | ih | 1 (L) | B. sparsiflora | lin I S1 and S6, lin III S16 | apomict now included in B. pauciflora |
| Arab0967 | *Boechera sparsiflora* | ii | 1 (B) | B. microphylla, B. sparsiflora and others | lin I S1 and S6, lin III S16 | parent in at least four apomictic hybrids |
| Arab0999 | *Boechera sparsiflora* | ou | 1 (KE) | derived from S7; B. retrofracta, B. pinetorum | lin I S1 and S6, lin III S16 | parent in at least four apomictic hybrids |
| Arab1011 | *Boechera “microphylla”* | ik | 1 (FO) | B. microphylla; in S20 (S20; Kiefer et al. 2009) | S20 and derived haplotypes in lin I. and CI in lin. III | two apomict hybrids known |
| Arab1020 | *Boechera “microphylla”* | im | 3 (CI) | see Kiefer et al. 2009; among them B. microphylla | S20 and derived haplotypes in lin I. and CI in lin. III | two apomict hybrids known |
| Arab1023 | *Boechera “microphylla”* | in | 3 (CI) | see Kiefer et al. 2009; among them B. microphylla | S20 and derived haplotypes in lin I. and CI in lin. III | two apomict hybrids known |
| Arab1035 | *Boechera pallidifolia* | io | 3 (CL) | see Kiefer et al. 2009; derived from CI | CL and DS | prone to hybridisation giving rise to several apomictic species |
| Arab1040 | *Boechera pendulina* | ip | 1 (EN) | see Kiefer et al. 2009; included in S2; B. pendulina | lin. I, S2 and derived | apomictic hybrids known |
| Arab1064 | *Boechera perennans* | pb | 2 (AB) | included in S8, the central cpDNA type of the network; mixed species | mainly S16 | B. gracilenta as apomict has been excluded from B. perennans |
| Arab1068 | *Boechera perennans* | ir | 3 (CI) | one of the main species sharing CI | mainly S16 | apomictic hybrids known |
| Arab1078 | *Boechera puberula* | it | 1 (S) | B. puberula | mainly S | serves as parent for apomictic hybrids |
| Arab1095 | *Boechera puberula* | on | 1 (S) | B. puberula | mainly S | serves as parent for apomictic hybrids |
| Arab1104 | *Boechera puberula* | iu | 1 (O) | B. puberula | mainly S | serves as parent for apomictic hybrids |
| Arab1113 | *Boechera williamsii* | iv | 2 (AS) | “B. divaricarpa | AS | parent of apomictic B. saxomontana |
| Arab1435 | *Boechera pendulina* | kt | None |  |  |  |
| Arab1665 | *Boechera davidsonii* | mh | (KG) |  |  |  |
| Arab1701 | *Boechera xylopoda* | mt | none |  |  |  |
| Arab1713 | *Boechera platysperma* | my | (GV) |  |  |  |
| Arab1724 | *Boechera perennans* | ne | none |  |  |  |
| Arab1769 | *Boechera puberula* | sw | (U) |  |  |  |
| Arab1786 | *Boechera puberula* | sf | (S) |  |  |  |
| Arab1849 | *Boechera fernaldiana* | pd | none |  |  |  |
| Arab1851 | *Boechera fernaldiana* | pc | none |  |  |  |
| Arab1872 | *Boechera gunnisoniana* | pk | none |  |  |  |
| Arab1985 | *Boechera texana* | rv | none |  |  |  |
| ES030 | *Boechera stricta* | nw | none |  |  |  |
| ES031 | *Boechera “divaricarpa”* | nx | none |  |  |  |
| ES037 | *Boechera stricta* | nz | none |  |  |  |
| ES046 | *Boechera “divaricarpa”* | oc | none |  |  |  |
| ES047 | *Boechera “divaricarpa”* | od | none |  |  |  |
| ES050 | *Boechera stricta* | oy | none |  |  |  |
