# Supplementary material for: A Continental-Wide Perspective: The Genepool of Nuclear Encoded Ribosomal DNA and Single-Copy Gene Sequences in North American Boechera (Brassicaceae)
Source: PLoS One. 2012 May 14;7(5):e36491. doi: 10.1371/journal.pone.0036491 (PMC3351400; doi:10.1371/journal.pone.0036491)
Supplement: Table S8 — Summary of the phylogenetic tree and network reconstructions for all taxa included in the study; based on the data conclusions were drawn on lineage sorting and hybridisation which are stated in the last column. (DOC) [file pone.0036491.s008.doc]

| **Taxon** | **ITS types within a lineage** | **ITS types outside this lineage** | **ITS types unresolved on polytomy** | **Position in ITS network** | **Geographic distribution ITS types within lineage** | **Geographic distribution ITS types outside lineage** | **Lineage in *At2g25920 (ELF3)*** | **Lineage in *At3g18900 (VRN1)*** | **Lineage sorting** | **Conclusion** |
| --- | --- | --- | --- | --- | --- | --- | --- | --- | --- | --- |
| *B. arcuata* | SX, OG |  |  | connected to Z and H, reticulate | Sierra Nevada South |  | unresolved | missing |  | with *B. rectissima* and *B. sparsiflora* (putative hybrids?) in reticulate relationship |
| *B. atrorubens* | GX |  |  |  | Cascade Mountains Leeward Forests |  | missing | missing |  | only one accession; this within GX genepool |
| *B. bodiensis* | PE |  |  |  | Great Basin Shrub Steppe West |  | missing | missing |  | only one accession; this one derived from GX genepool |
| *B. breweri* | MV, HA, GX, KN |  |  | GX derived from rare ITS type EI, others derived from GX | Klamath/Siskiyou Forests, California Interior Chaparral, California Valley |  |  | clade 1; with *B. koehleri*, *B. sparsiflora* and *B. microphylla* | incomplete, but complete separation from ancestral genepool H | ITS lineage unresolved with *B. koehleri* and *B. sparsiflora*, but separated from ancestral genepool H; evolved from GX genepool |
| *B. breweri var. shastaensis* | PF, HA |  |  |  | Klamath Siskiyou Forests |  | with *B. breweri* | clade 1; with *B. koehleri*, *B. sparsiflora* and *B. microphylla* |  | Endemic which occurs with *B. breweri* and is in the same ITS lineage |
| *B. californica* |  | EV with *B. perennans* and *B. pendulina*, OG with *B. sparsiflora* and *B. arcuata* |  | OG has reticulate connections, EV is derived from F which has reticulate connections | Sierra Nevada South,  California Coastal Sage and Chaparral |  | missing | missing |  | only two accessions; possible hybrid origin |
| *B. canadensis* |  |  |  |  | Eastern North America |  |  |  |  | not discussed here, see Kiefer et al. 2009 |
| *B. cobrensis* | GY, KR, KS | HM with *B. paupercula* | AD, HK, HL | lineage derived from AD | Great Basin, Snake/Columbia, Wyoming Shrub Steppe | Snake/Columbia Shrub Steppe | separate lineage | clade 1; unresolved | incomplete | young taxon that evolved from the AD genepool |
| *B. collinsii* |  | R, L in *B. stricta* lineage | H, AE, AF, CO | H is ancestral ITS type | Montana Valley Foothill Grasslands,  NW Mixed Grasslands,  South Central Rockies, Interior Yukon Alaska Lowland Taiga, New England/Acadian Forest, Canadian Aspen Forest and Parklands,  Northern Mixed Grasslands,  Yukon Interior Dry Forest |  | missing | missing |  | evolved from ancestral genepool H; shows signs of hybridisation with *B. stricta* |
| *B. constancei* | PG, PH, PI |  |  | derived from AD by three steps | Sierra Nevada North |  | one accession in one lineage with *B. suffrutescens*, *B. paupercula*, *B. formosa* | clade 2; one accession together with one *B. suffrutescens* and one *B. rigidissima* | sample number too low; could be complete | separated lineage which includes one *B. suffrutescens* accession, from AD genepool |
| *B. crandallii* |  |  | LN, LO, LP, SL, SB, PK, F, HN | SL derived from F; F also contains *B. crandallii* | Colorado Rockies, Colorado Plateau |  | separate lineage with four accessions | clade 1; eight accessions unresolved on polytomy | incomplete | young taxon with beginning separation (own ITS types, own lineage in one single copy gene tree); several ITS types with ambiguous sites and hence putative hybridogenous background |
| *B. cusickii* | H | B from *B. stricta* lineage | MI | Mi Derived from H | Palouse Grasslands, Snake Columbia Shrub Steppe |  | missing | missing | incomplete | five accessions, three in H genepool, B may indicate hybridisation with *B. stricta* |
| *B. davidsonii* | MG, MH, OQ, PL |  |  | derived from gw by several mutational steps | Great Basin, Sierra Nevada |  | missing | missing | complete | well separated lineage from gw |
| *B. falcata* |  |  | RY | derived from gw | Russia, Siberia |  | missing | missing |  | only one accession |
| *B. falcatoria* | with fi and fj |  | EE | derived from AD | Great Basin Shrub Steppe East |  | missing | missing |  | only one accession; located in putative hybridogenous lineage |
| *B. falcifructa* |  |  | PM | from gw by several mutational steps | Great Basin Shrub Steppe central East |  | unresolved | missing |  | only one accession; long branch on polytomy in ITS phylogeny |
| *B. fendleri* |  | KD with GZ,  IS with KE  SN in mixed species group 1,  HP with *B. texana* lineage | EV, F, NM, HO, HR, G | GZ connected to AD and several other ITS types;  IS-KE directly derived from G; SN in mixed species group derived from AD by reticulate ITS type HY | Colorado Plateau, Great Basin, Mojave Desert, Chihuahua Desert, Colorado Rockies Forest, Arizona Mountains, Wasatch Uinta, Western Short Grasslands |  | in mixed species group with same individual as in ITS; with *B. gracilipes*; unresolved on the polytomy with individuals carrying ITS type G, | clade 2; lineage with *B. pendulina* and *B. perennans* |  | seems to mainly come out of G genepool but also shares the connected ITS types EV and F; large potential for hybridisation with AD genepool; NM has ambiguous sites ans also indicates hybridisation |
| *B. fernaldiana var. fernaldiana* |  |  | HU |  | Great Basin |  |  |  |  | only one accession; HU has ambiguous sites |
| *B. fernaldiana var. fernaldiana* |  | HS and OP with mixed species group; BT with *B. pendulocarpa*, with IL | OK | HW derived from H; mixed species group derived from AD through reticulate ITS type HY | Great Basin |  | missing | missing |  | possibly of hybrid origin indicated by presence in several lineages |
| *B. fernaldiana var. vivariensis* |  | PN with PV from *B. lignifera* | F | F is derived from H through reticulate connections | Colorado Plateau |  | missing | missing |  | only two accessions |
| *B. formosa* | LC, PO with *B. pulchra* |  | AD, PP, PQ | LC, PO lineage derived from AD by reticulate connections | Colorado Plateau |  | separate lineage with B. pulchra; also with *B. suffrutescens* and *B. constancei* | clade 2; with *B. lincolnensis* and *B. pulchra* |  | possible hybrid origin in which *B. pulchra* and a taxon from the AD genepool may have been involved |
| *B. formosa* | LC |  |  | in *B. pulchra*/*B. formosa* luineage which is derived from AD | Colorado Plateau Shrub Steppe |  | missing | missing |  | onyl one accession; goes together with *B. pulchra*/*B. formosa* |
| *B. glareosa* |  |  | G | G derived from H; reticulate connections | Wasatch/Uinta |  | with *B. lignifera* | missing |  | only one accession |
| *B. glaucovalvula* | NH, SP; sister of *B. pulchra* |  |  | together with *B. pulchra* in derived position | Mojave Desert, Sonora Desert, Sierra Nevada |  | separate lineage | missing | complete | clearly separate lineage which evolved out of the H genepool together with *B. pulchra* |
| *B. gracilenta* | AD with *B. pallidifolia* and others | GZ | LF | GZ has reticulate connections; LF derived from AD | Colorado Plateau Shrub Steppe, Wasatch/Uinta |  | with *B. pallidifolia* | clade 1; unresolved | incomplete | individuals with GZ and LF maybe be of hybrid origin; *B.gracilenta* may be a young species from the AD genepool |
| *B. gracilipes* |  |  | F | F is derived from H through reticulate connections | Colorado Plateau, Great Basin, Arizona Mountains |  | separate lineage with *B. fendleri* |  | incomplete | lineage which evolved from the hybridogenous genepool F |
| *B. gunnisoniana* |  | OS with *B. oxylobula* | PK, AD, HX | AD is derived from H | Colorado Plateau East |  | missing | missing |  | PK, HX have both ambiguous sites; differing positions and ambiguous sites suggest hybrid origin |
| *B. holboellii sensu Al-Shehbaz* |  |  | H | H is ancestral ITS type with biggest distribution range | Greenland |  | missing | missing |  | only two accessions; possibly evolved from ITS type H which has the largest distribution range |
| *B. howellii* | KW |  |  | in lineage derived from H | Sierra Nevada South |  | missing | missing |  | only one accession; together with *B. platysperma* accessions derived from genepool H |
| *B. howellii* |  |  | PR |  | Cascades |  | missing | missing |  | only one accession |
| *B. inyoensis* |  | ML and HY in mixed species group | MK, I, AD, AR | I derived from H, AD derived from H; AR derived from reticulate EI | California Montane Chaparral, Sierra Nevada, Great Basin West |  | mixed species group and with *B. breweri* | clade 1; with *B. lignifera;* clade 2 mixed species |  | either misidentified because taxon that forms mixed species is unknown or of hybrid origin so it ends up with a hybrid swarm |
| *B. johnstonii* |  | HY in mixed species group |  | HY | Great Basin |  | unresolved on polytomy | missing |  | only one accession; this has ambiguous sites |
| *B. koehleri* | MM, SM, SA, KN |  | AC, HZ | AC derived from H, GX derived from H | Klamath/Siskiyou |  | with *B. breweri*, *B. lemmonii*, *B. suffrutescens* | clade 1; together with *B. breweri* and *B. sparsiflora* and *B. microphylla* | incomplete | very closely related to *B. breweri* and *B. sparsiflora*; HZ has ambiguous sites |
| *B. laevigata* |  |  |  |  | Eastern North America |  |  |  |  | not discussed here, see Kiefer et al. 2009 |
| *B. lasiocarpa* |  |  | AD, OH | AD derived from H | Great basin East, Wasatch/Uinta |  | missing | missing |  | derived from AD genepool |
| *B. lemmonii* | ER, KL, LY, LZ, PS, PT | GX with *B. sparsiflora* | KM | ER derived from ITS type AD | Sierra Nevada, cascade Mountains, South Central Rockies, North Central Rockies, Colorado Rockies, Wasatch/Uinta |  | own lineage; unresolved on polytomy; with B*. breweri* and *B. koehleri*, *B. suffrutescens* | clade 1; separate lineage | complete | separate lineage which evolved from AD genepool; some indications for hybridisation |
| *B. lignifera* | EE with IE (and FI, FJ) | I with DI; ER as typical *B. lemmonii* ITS type; IW also in *B. lemmonii* lineage; GZ with KD; PV with PN; | H, AC, AD, IX, PU, IA, IB, IC, ID | H ancestral, AC derived from H, AD derived from H | Great Basin, Sierra Nevada, Okanogan Forest, Colorado Plateau, South Central Rockies, Wasatch/Uinta, Arizona Mountains, Wyoming Basin |  | with *B. pallidifolia* and *B. gracilentai*, with *B. glareosa* and unresolved on polytomy | clade 1; on polytomy and lineage with *B. inyoensis* | incomplete | either of hybrid origin or still hybridising frequently |
| *B. lincolnensis* |  |  |  | PW, EV | Mojave Desert |  | unresolved on polytomy | clade 2; one group with B*. formosa* and *B. pulchra* |  | only two accessions |
| *B. lincolnensis* | MU, SO, MX in typical *B. pulchra* lineage |  |  | lineage derived from ITS type H by three steps | Mojave Desert |  | missing | missing | complete in respect to ancestral ITS type H; incomplete in respect to *B. glaucovalvula* | part of well separated lineage in which sublineages are not separated |
| *B. lyallii* |  | AD, AB in one lineage; L, V in one lienage;  PY in one lineage with FQ and FR | IY, IZ, | IZ derived from AZ | Cascade Mountains, Okanogan Forest, Alberta Mountains, North Central Rockies, Sierra Nevada, South central Rockies, Great Basin |  | separate lineage | clade 1; sister to *B. stricta* | incomplete | the occurence in separate lineages in the network indicates a possible hybridogenous background; IY has ambiguous sites |
| *B. macounii* |  | IL with *B. fernaldiana* , IW with *B. lemmonii* | AD, LT, H | AD derived from H; IL derived from H; LT derived from H | South Central Rockies, Great Basin, Colorado Plateau, Snake/Columbia Shrub Steppe, Eastern Canadian Forest | Great Basin Shrub Steppe Central East, Eastern Canadian Forest | within *B. microphylla* lineage and unresolved on polytomy | clade 1; with *B. microphylla* and *B. koehleri*; clade 2; sister to *B. pendulina*, *B. fendleri* and *B. perennans* group | incomplete | evolved from H genepool into AD; not separable from *B. microphylla* |
| *B. microphylla* |  | OP in mixed species group | H, KA, KB |  | Central Southern Cascades, Great Basin, Blue Mountains, North Central Rockies | Great Basin Shrub Steppe Central West | separate lineage | with *B. microphylla* and *B. koehleri* | incomplete | evolved from H genepool; presence in mixed species group may show tendency for hybridisation; KA and KB have ambiguous sites |
| *B. missouriensis* |  |  |  |  | Eastern North America |  |  |  |  | not discussed here, see Kiefer et al. 2009 |
| *B. nevadensis* |  |  | PX |  | Mojave Desert |  | unresolved on polytomy | missing |  | only one accession |
| *B. oxylobula* | HC with *B. oxylobula* |  | G | HC is derived from F | Colorado Plateau, Wasatch/Uinta |  | occurs with *B. pendulina in* one lineage and a separate *B. oxylobula* lineage | missing |  | taxon which has ITS type G is also misplaced in single copy marker; may be hybrid; hybridogenous origin also suggested for other accessions due to being derived from F genepool |
| *B. oxylobula* | HC, PZ, RA; lineage with *B. gunnisoniana* and *B. demissa* |  |  | derived from ITS type F by one missing ITS type | Colorado Plateau, Colorado Rockies |  |  |  |  | evolved from genepool F; putative hybridogenous origin |
| *B. pallidifolia* |  |  | AD, LU, RB, IO | AD derived from H | Colorado Plateau, Wasatch/Uinta, Wyoming Basin |  | together with *B. gracilenta* | clade 1; unresolved on polytomy |  | evolved from AD genepool |
| *B. parishii* |  |  | F | reticulate ITS type in network | California Montane Chaparral |  | unresolved on polytomy | missing |  | only two accessions; hybrid origin? |
| *B. patens* | ST |  |  |  | Appalachian blue Ridge Forest |  | missing | missing |  | probably no *Boechera* |
| *B. pauciflora* | H, IF, IG, IH, HF |  | AR, sharing with *B. pinetorum* and *B. inyoensis* | AR connected to the same missing ITS type as GX; the ITS types in the lineage derived from GX which itself was derived from H by two steps | Eastern Cascades Forest, Great Basin Shrub Steppe West |  | missing | missing |  | the connection with AR indicates a possible hybrid line; otherwise derived from the GX genepool which is the typical *B. sparsiflora* genepool |
| *B. paupercula* | RC with one *B. cobrensis* accession |  | V | RC derived from AD; V derived from W | Great Basin West, South Central Rockies |  | missing | missing |  | only two accessions; occurence in far separated ITS types suggests hybrid origin |
| *B. pendulina* | EV, G, F |  | NI, IP, KT | NI derived from F | Colorado Plateau, Great Basin East, Wasatch/Uinta, Mojave Desert, Arizona Mountains Forest |  | lineage with *B. demissa* and unresolved on polytomy | clade 2; lineage together with *B. fendleri* and *B. perennans* | incomplete | evolved from G genepool but is also present in ev genepool; IP and KT have ambiguous sites |
| *B. pendulina* |  |  | EV, KT |  | Colorado Plateau, Wasatch/Uinta |  | lineage with *B. demissa* and unresolved on polytomy | lineage together with *B. fendleri* and *B. perennans* | incomplete | evolved from G genepool but is also present in evgenepool |
| *B. pendulocarpa* | BT, BX, BW;  AB, BV | AU with *B. puberula*, G with *B. pendulina* and *B. fendleri*, | BU, EO, EP , EQ , FF , FG , H | BT/BX/BW reticulate connection to Z, H and G; AB/BV derived from AD | Cascade Mountains, Great Basin Shrub Steppe, Montana Valley Foothill Grasslands, Okanogan Forest, Sierra Nevada, South Central Rockies, Wasatch/Uinta, Wyoming Basin |  | with B. platysperma | missing |  | found in two independent ITS lineages; may indicate hybrid origin of the taxon and subsequent split of genepool into one following one parent and the other one following the other parent or that *B. pendulocarpa* is polyphyletic |
| *B. perennans* |  |  | f, ev, ku, nd, ne, nf, ng, ad, re, rf, pb, ir, RD | F and EV are connected, AD is in a different part of the network; NG reticulate with ni and ev; re and rf derived from ev | Great Basin, Mojave Deesert, Colorado Plateau, Sonora Desert, Sierra Nevada, Chihuahua Desert, Wasatch Uinta |  | unresolved on polytomy and with *B. gracilenta*with one accession | clade 1; together with *B. pendulina* and unresolved on polytomy; clade 2; lineage with *B. fendleri* and *B. pendulina* |  | KU, ND, PB, NE, IR with ambiguous sites; evolved from EV genepool; potential for hybridisation |
| *B. perstellata* |  |  |  |  | Eastern North America |  |  |  |  | not discussed here, see Kiefer et al. 2009 |
| *B. pinetorum* |  | AJ with mixed species group; Y with BO, BP and others; Z with *B. rectissima* and *B. sparsiflora* | AC, AD, AL , AR, AU, CD, CF, CG, CH, CI, CK, DY, F, FK, GA, GH, H, I | AC, AD derived from H; F with reticulate connections, | California Montane Chaparral, Colorado Plateau,  Colorado Rockies,  Eastern Cascades, Great Basin, Mojave Desert, Sierra Nevada, Snake/Columbia Shrub Steppe, South Central Rockies, Wasatch/Uinta, Wyoming Basin |  | missing | missing |  | highly hybridogenous taxon |
| *B. pinzliae* | RG |  |  |  | Great Basin Shrub Steppe |  | missing | missing |  | only one accession |
| *B. platysperma* | kv, my, mz, na, nb, nc, rh; well supported separate lineage |  |  | derived from ITS type H by three mutational steps | Sierra Nevada, Eastern Cascades, California Montane Chaparral |  | well supported lineage together with *B. pendulocarpa* accessions | missing |  | well separated old lineage which evolved from genepool H |
| *B. polyantha* | RI, H |  |  | RI derived from EV; reticulate connection to rf | Cascade Mountains Leeward Forests, Palouse Grasslands, North Central Rockies, Okanogan Forest |  |  |  |  | presence in these two different lineages suggests hybrid origin |
| *B. puberula* | KX, SW, RJ, EU, ON is the lineage derived from EU |  | AU, AC, IU, PA, SV, SF, RK, OL, IT, OM | AU, AC, EU connected; sharing in decreasing proportion with *B. retrofracta* | Great Basin and Snake/Columbia Shrub Steppe, Eastern Cascades, Klamath Siskiyou |  | missing | missing | incomplete | taxon evolved out of a separate *B. retrofracta* genepool (AC) |
| *B. pulchra* | KY, LD, NK, NL, SU, RL, EU | LA, LB, LC with *B. formosa;*  EU with *B. puberula* | H | lineage derived from ITS type H by three steps | Great Basin Shrub Steppe, Colorado Plateau Shrub Steppe, California Interior Chaparral, Mojave Desert |  | with *B. formosa* and separate lineage | clade 2; with *B. formosa* and *B. lincolnensis* | complete in respect to ancestral ITS type H; incomplete in respect to *B. glaucovalvula* | part of well separated lineage in which sublineages are not separated; misidentified *B. formosa* included |
| *B. pusilla* |  |  | F | reticulate ITS type | Southern central Rockies |  | missing | missing | incomplete | may be of hybrid origen |
| *B. pygmaea* |  |  |  | derived from ao or fq by several steps |  |  |  | clade 2; own lienage |  | only one accession |
| *B. rectissima* | Z, LE, MP |  |  | LE and MP connected to Z; reticulate relationships with BT, H and OG | Sierra Nevada North and South, Klamath/Siskiyou |  | separate lineage | clade 1; on polytomy | incomplete | may be of hybrid origin |
| *B. repanda* | MR, MS, RM, RN |  |  | not connected to network | California Valley, California Montane Chaparral, Great Basin West, Mojave |  |  |  | complete | placed outside of Western North American *Boechera* |
| *B. retrofracta* |  | BT with BX; ER in *B. lemmonii* lineage; EU with *B. puberula*, GX with *B. sparsiflora*, GY with *B. cobrensis*, HA with *B. breweri*, Z with *B. rectissima*, I with DI | AA, AC, AD, AU, CE, CK, CL, CR, DU, DX, DY, DZ, ET, EV, EY, EZ, F, FA, FB, FC, FD, FE, FK, GY, H, HA, R, Y, Z |  | Alaska/St. Elias Range,Alberta Mountain Forest, Blue Mountains, Cascade Mountains, Colorado Plateau,  Colorado Rockies, Great Basin, Interior Yukon Alaska Lowland, Klamath/Siskiyou,  Montana Valley Foothill Grasslands,  North Central Rockies,NW Mixed Grasslands, South  Okanogan Forest, Sierra Nevada, Snake/Columbia Shrub Steppe, South Central Rockies, Wasatch/Uinta, Western Canadian Forests, Western Great Lakes Forest, Wyoming Basin Shrub Steppe, Yukon Interior Dry Forest |  | missing | missing |  | found allover the network but mostly nodes which contain *B. retrofracta* are connected to eachother; *B. retrofracta* may be the background from which the other taxa evolved |
| *B. rigidissima* | KV with *B. platysperma* |  |  | lineage containing KV derived from H in three steps | Klamath/Siskiyou, Sierra Nevada |  | missing | clade 2; with *B. suffrutescens* and *B. constancei* | complete in respect to ancestral ITS type H | only two accessions; both within B. platysperma; young taxon derived from this lieage? |
| *B. schistacea* | SH, RO, RP |  |  | derived from highly reticulate ITS type W | Colorado Plateau Shrub Steppe, Great Basin West |  | with *B. sparsiflora* and unresolved | clade 1; unresolved |  | putative hybrid origin |
| *B. shockleyi* | MN, MO in mixed species group 1 |  |  | derived from AD through the reticulate ITS type HY | Mojave Desert, Great Basin Shrub Steppe |  | mixed species group | clade 2; mixed species group |  | only two accessions; may be of hybrid origen |
| *B. shortii* |  |  |  |  | Eastern North America |  |  |  |  | not discussed here, see Kiefer et al. 2009 |
| *B. sparsiflora* | GX, LM, H, OU |  | II | GX is connected to H by two steps, LM derived from GX | Great Basin West, Snake Columbia Shrub Steppe West, Eastern Cascades Forest, Montana Valley Foothill Grasslands |  | missing | missing |  | OU with ambiguous sites; same as for other *B. sparsiflora* |
| *B. spatifolia* | HQ |  | U | HQ is with *B. texana*; reticulate connections too HH or W; U has a reticulate connection to F and G | Colorado Rockies Forest |  | missing | missing |  | putative hybrid |
| *B. stricta* | own ITS lineage |  |  |  | North America apart from South-East |  | separate lineage | clade 1; separate lineage together with *B. lyallii* |  | well separated sexual lineage which is parent in hybridisation events |
| *B. subpinnatifida* | LW and RJ together with *B. puberula* in AC lineage | LX with *B. patens* on polytomy with outgroup; LI and RU form separate lineage |  | LI and RU connected to AC | Klamath/Siskiyou, Eastern Cascades, Great Basin Shrub Steppe West |  | unresolved on polytomy | missing |  | LX seems to be a misidentification; *B. subpinnatifida* evolved from AC genepool |
| *B. suffrutescens* |  | GX with *B. sparsiflora*  LK with *B. constancei* | ME | LK derived from AD by three steps; ME derived from AC | Southern Central Rockies, Sierra Nevada North, Blue Mountain Forest |  | with *B. constancei* and *B. formosa*, with *B. breweri* and with ES090 | clade 2; with *B. constancei* and *B. rigidissima* |  | LK and ME are the most frequent ITS types; the individual carrying GX seems to be a misidentification or a hybrid; questionably if the individuals carrying LK are actually *B. constancei* or if *B. suffrutescens* is actually split into two lineages |
| *B. texana* | RV, RW with *B. fendleri* |  |  | reticulate connections too HH or W | Chihuahua Desert |  | missing | missing |  | probably of hybrid origin; the two B. fendleri accessions clustering with it may bis misdetermined |
| *B. tiehmii* | ER |  |  |  | Sierra Nevada South |  | missing | missing |  | only one accession; this clusters with *B. lemmonii* |
| *B. williamsii* | BT |  | RX, IV, AO | RX and AO have a connection to FQ; AO is internal with reticulate connections | Southern Central Rockies Forest, Wyoming Basin Shrub Steppe |  | missing | missing |  | probably hybridogenous origin |
| *B. xylopoda* | MT, MW |  |  | lineage derived from ITS type H by three steps | Mojave Desert, Great Basin |  | unresolved | missing | complete in respect to ancestral ITS type H; incomplete in respect to *B. glaucovalvula* | part of well separated lineage in whichsublineages are not separated |
| *Borodinia tilingii* | RZ; sister to *B. laevigata*/*B. missouriensis* lineage |  |  |  | Russia |  |  |  |  | not discussed here, see Kiefer et al. 2009 |
| *B. ”divaricarpa”* | multipe of *B. stricta* lineage | BT, BX | H | scattered across network, mainly *B. stricta* lineage but also H, BT, BX | complete distribution range of *Boechera* because determined according to Rollins, 1993 |  | missing | missing |  | accessions identified according to taxonomy by Rollins, 1993; whatever hybridogenous group this is, *B. stricta* is one parent and *B. pendulocarpa* or *B. retrofracta* may also be involved |
| *B. ”fernaldiana”* |  | OP with mixed species group; IL with HW | PD, PC, HV | HW derived from H; mixed species group derived from AD through | Great Basin, Snake/Columbia Shrub Steppe |  | unresolved | missing |  | PD, PC and HV have ambiguous sites; presence of taxon with different unrelated ITS types suggests hybridisation |
| *B. „microphylla“* |  | IL with *B. fernaldiana* , IM in mixed species group | H, IK, IL, IM, IN | IL derived from H | Snake/Columbia, South central Rockies, Great basin, Wasatch/Uinta | Snake/Columbia Shrub Steppe, Great Basin Shrub Steppe Central West | separate lineage | clade 1; with *B. microphylla* and *B. koehleri* | incomplete | evolved from H genepool; presence in mixed species group may show tendency for hybridisation; IK, IM, IN have ambiguous sites |
| *B. „sparsiflora“* | GX | LV and OG with mixed species group,  C in *B. stricta* lineage, RR with BO/BP,  I with di | F, H, U, MF | RR in group with BO/BP derived from H; I derived from H or AS; U in reticulate connection between F and G | Great Basin Shrub Steppe West, California Coastal Sage and Chaparral, California montane Chaparral, Snake/Columbia Shrub Steppe, Sierra Nevada South, Colorado Rockies, South Central Rockies |  | with *B. schistacea* and with mixed species group | clade 1; with *B. breweri* and in mixed species group; clade 2; mixed species group |  | includes many potential hybrids, maybe therefore not determined further; hybrids may be individual with C, RR, F; individuals with LV and OG may be *B. sparsiflora var. californica*; individual with GX may be *B. sparsiflora var. sparsiflora* |
