# Supplementary material for: A Continental-Wide Perspective: The Genepool of Nuclear Encoded Ribosomal DNA and Single-Copy Gene Sequences in North American Boechera (Brassicaceae)
Source: PLoS One. 2012 May 14;7(5):e36491. doi: 10.1371/journal.pone.0036491 (PMC3351400; doi:10.1371/journal.pone.0036491)
Supplement: Table S10 — List of apomictic taxa according to [14] included in this study and ITS types shared by these accessions. (DOC) [file pone.0036491.s010.doc]

| **Taxon** | **ITS type** | **Apomictic or apomictic + sexual according to Al-Shehbaz and Windham 2010** |
| --- | --- | --- |
| *B. bodiensis* | PE | apomictic |
| *B. “divaricarpa”* | ad, ay, az, be, bd, bc, bf, bg, bh, bi, bj  bl, bn, bo, bp, br  bt, bw, bx, c, ca, cu  cv, cy, fw, cy, q, cx  db, dc, de, dd, df, dg, dh, di, dl, w, dm, dp, w, do, dn, ds, e, ea, ee, ef, ei, ej, el, em, ek, fh, dn, fl, fn, fo, fq, fr,  ft, fy, gm, h, i, k, l, n, o, p, q, r, s, t, u, v, y | apomictic |
| *B. falcatoria* | EE | apomictic |
| *B. falcifructa* | PM | apomictic |
| *B. gracilenta* | gz, ad, lf, ad, ad, ad, ad, ad, ad | apomictic |
| *B. holboellii* | H | apomictic + sexual |
| *B. inyoensis* | hy, mk, i, ad, ml, ar | apomictic |
| *B. lemmonii* | er, gx, kl, km, ly, lz, ps, pt | apomictic + sexual |
| *B. lignifera* | ac, ad, ee, er, gz, h, i, ia, ib, ic, id, ie, iw, ix, pu, pv | apomictic + sexual |
| *B. lyallii* | ab, ad, iy, iz, l, py, v | apomictic + sexual |
| *B. macounii* | ad, h, il, iw, lt | apomictic |
| *B. microphylla* | H, IM, IN, IK, IL, KA, KB, OP | apomictic + sexual |
| *B. pauciflora* | if, ig, ih, h, ar, hf | apomictic |
| *B. pendulina* | ev, f, g, ip, kt, ni, rd | apomictic + sexual |
| *B. pinetorum* | ac, ad, aj, ak, ag, ai, ah, an, ag, ap, ar, as, au, aw, an, ax, hh, ag, f, fi, fj, fz, an, gb, gd, gf, gg, ap, h, hi, an, ap, am, ag, i, w,x, w, y, z | apomictic + sexual |
| *B. pinzliae* | rg | apomictic + sexual |
| *B. pusilla* | f | apomictic |
| *B. rigidissima* | kv | apomictic |
| *B. suffrutescens* | lk, gx, me | apomictic + sexual |
| *B. xylopoda* | mt, mw | apomictic |
