# Supplementary figures and images for: A Continental-Wide Perspective: The Genepool of Nuclear Encoded Ribosomal DNA and Single-Copy Gene Sequences in North American Boechera (Brassicaceae)
Source: PLoS One. 2012 May 14;7(5):e36491. doi: 10.1371/journal.pone.0036491 (PMC3351400; doi:10.1371/journal.pone.0036491)

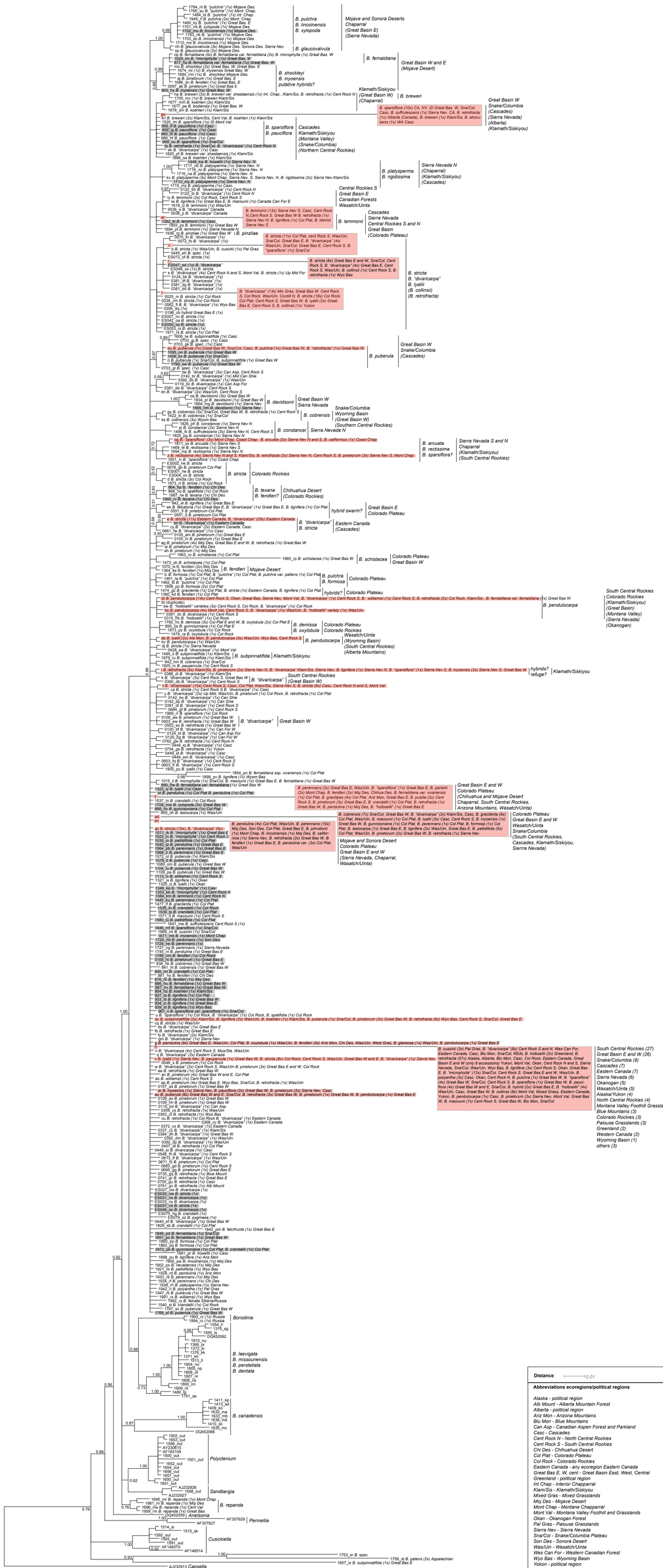

Supplement: Figure S1 — Phylogenetic reconstruction by Bayesian analysis based on ITS types of Boechera and several Boechereae taxa and Capsella rubella as outgroup; ITS types are labelled with their letter code; if they occur only once the accession number of the taxon carrying the ITS type is given; if the ITS type is shared among several accessions only the taxa are given including the frequency of the ITS type; geographical origin is indicated according to the names of the ecoregions given in [72]; for abbreviations of ecoregion names refer to the table given in the Figure; ITS types shaded in grey include ambiguous sites; ITS types shaded in red were used in the reduced network analysis (Figure 1). (PDF) [file pone.0036491.s011.pdf]

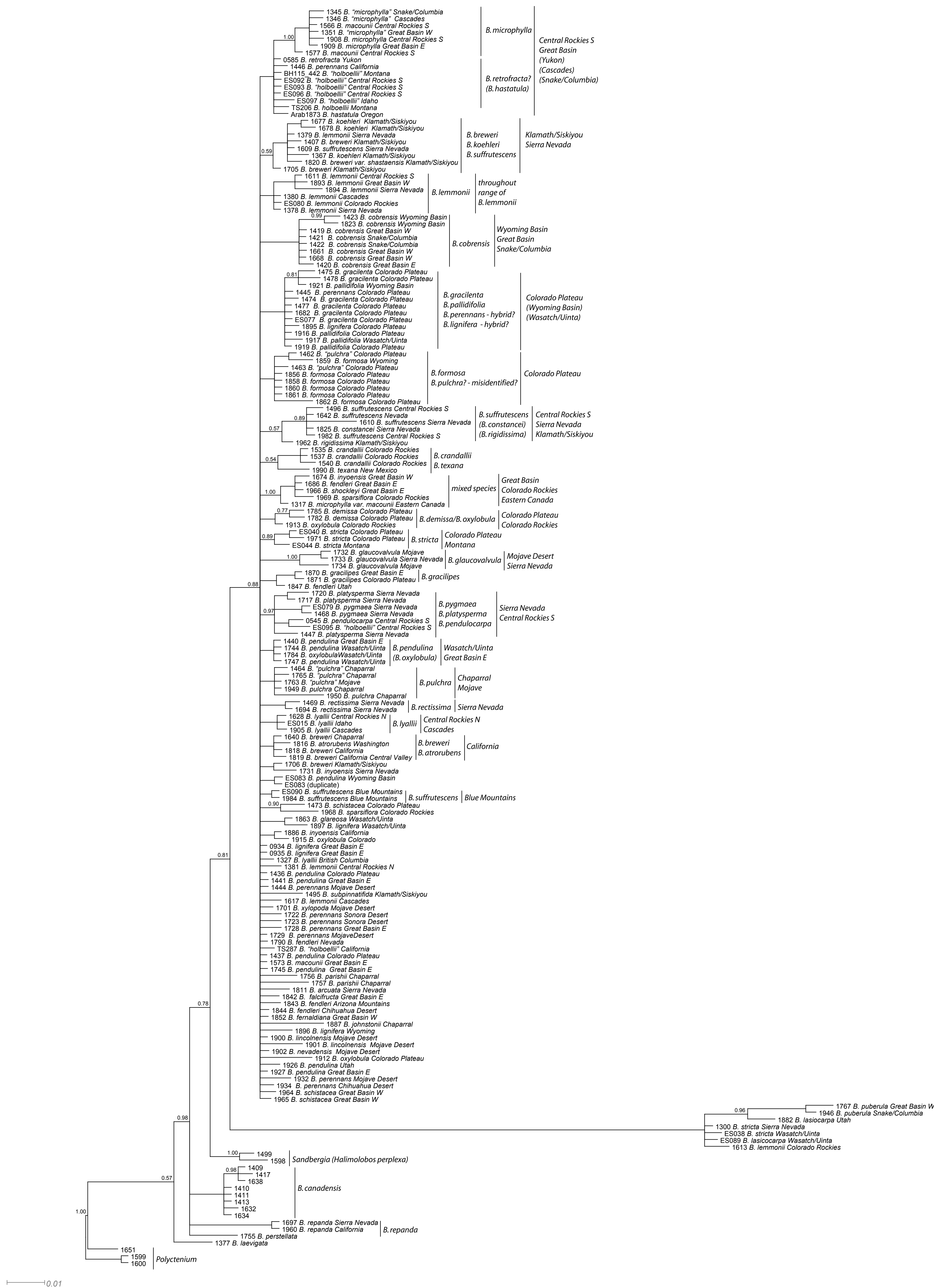

Supplement: Figure S2 — Bayesian analysis of the At2g25920 dataset; number, taxon identity and geographic origin of the accessions are given. (PDF) [file pone.0036491.s012.pdf]

0.01

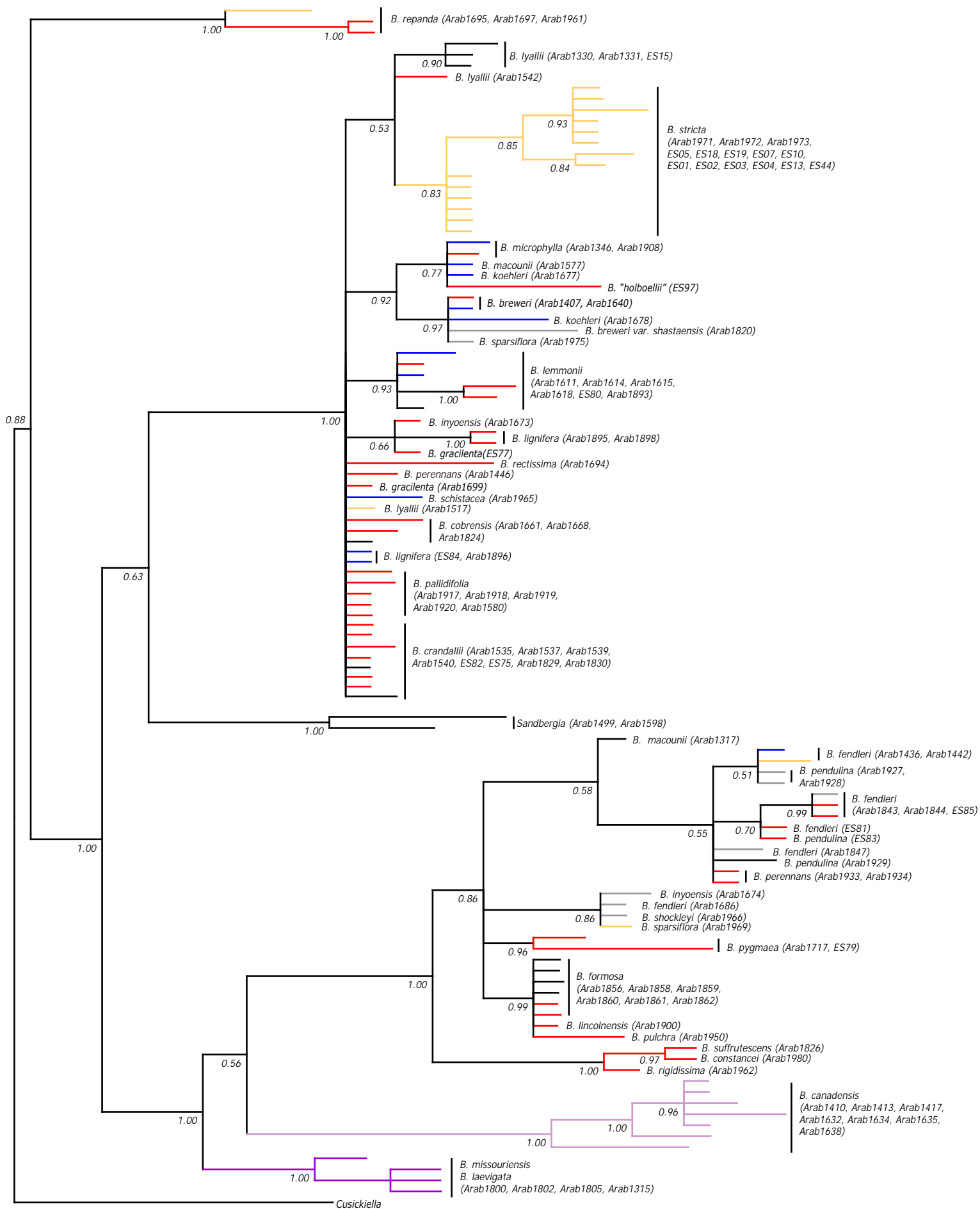

Supplement: Figure S3 — Bayesian analysis of the At3g18900 dataset; number, taxon identity and geographic origin of the accessions are given; in this phylogeny it is also indicated in which cpDNA lineage the accessions occurred in a previous study ([6]; blue = lineage 1, yellow = lineage 2, red = lineage 3, pink/lilac = lineage 5 and 6, grey = central cpDNA type). (PDF) [file pone.0036491.s013.pdf]
